# Neuropilin‐1 (NRP1) expression distinguishes self‐reactive helper T cells in systemic autoimmune disease

**DOI:** 10.15252/emmm.202215864

**Published:** 2022-09-07

**Authors:** Ben JE Raveney, Yosif El‐Darawish, Wakiro Sato, Yoshiyuki Arinuma, Kunihiro Yamaoka, Shohei Hori, Takashi Yamamura, Shinji Oki

**Affiliations:** ^1^ Department of Immunology National Institute of Neuroscience Tokyo Japan; ^2^ Department of Rheumatology and Infectious Diseases Kitasato University School of Medicine Sagamihara Japan; ^3^ Laboratory for Immunology and Microbiology Graduate School of Pharmaceutical Sciences, The University of Tokyo Tokyo Japan

**Keywords:** neuropilin‐1, NR4A2, self‐reactive T helper cells, SLE, systemic autoimmune disease, Immunology

## Abstract

Pathogenic T helper cells (Th cells) that respond to self‐antigen cannot be easily distinguished from beneficial Th cells. These cells can generate systemic autoimmune disease in response to widely expressed self‐antigens. In this study, we have identified neuropilin‐1 (NRP1) as a cell surface marker of self‐reactive Th cells. NRP1^+^ Th cells, absent in non‐regulatory T cell subsets in normal mice, appeared in models of systemic autoimmune disease and strongly correlated with disease symptoms. NRP1^+^ Th cells were greatly reduced in *Nr4a2* cKO mice, which have reduced self‐reactive responses but showed normal responses against exogenous antigens. Transfer of NRP1^+^ Th cells was sufficient to initiate or accelerate systemic autoimmune disease, and targeting NRP1‐expressing Th cells therapeutically ameliorated SLE‐like autoimmune symptoms in BXSB‐Yaa mice. Peripheral NRP1^+^ Th cells were significantly increased in human SLE patients. Our data suggest that self‐reactive Th cells can be phenotypically distinguished within the Th cell pool. These findings offer a novel approach to identify self‐reactive Th cells and target them to treat systemic autoimmune disease.

## Graphical abstract

In systemic autoimmune disease, self‐reactive Th cells support B cells to make pathogenic autoantibodies against a range of self‐antigens. Finding a marker of pathogenic self‐reactive Th cells allows targeting of these responses to treat disease regardless of antigen.

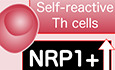

**The paper explained**

**Problem**

Autoimmune disease results from self‐attack by one's own immune system. In a systemic autoimmune disease such as Lupus, B cells make antibodies that target one's own tissues and organs and cause chronic damage. These responses are controlled by T helper cells that have developed to be specific for self rather than making protective responses against invading pathogens. As these dangerous cells hijack normally beneficial processes and look like helpful T cells, they are hard to destroy without harming important mechanisms that defend human health.

**Results**

Using a mutant mouse strain that develops a similar autoimmune disease to SLE, we investigated the properties of these self‐reactive T cells. We blocked a gene that was previously associated with dangerous responses and found the self‐reactive T cells were decreased and the autoimmune symptoms were decreased but defense against foreign pathogens was unaffected. This allowed us to compare normal and self‐reactive T cells directly and we found that they unusually expressed a protein called neuropilin‐1 on their cell surface. By targeting this receptor to deliver a payload that kills cells, we could destroy these pathogenic T cells and treat disease. We also found there was an increase in T cells with this neuropilin‐1 receptor in the blood of human patients with Lupus disease.

**Impact**

Testing blood for these neuropilin‐1‐expressing T cells could provide early information in patients to diagnose an autoimmune disease caused by self‐reactive T cells. The ability to identify this type of cell from similar helpful T cells is key to being able to make specific drugs to treat patients. Current treatments rely on blocking the function of part of the immune system or generally reducing the immune system activity, which can reduce the effectiveness of defense against common infectious diseases. If self‐reactive T cells causing autoimmune disease can be specifically targeted, it would allow a “silver bullet” therapy to effectively help sufferers whilst reducing potentially dangerous side effects.

## Introduction

Systemic lupus erythematosus (SLE), the archetypal systemic autoimmune disorder, is characterized by chronic inflammation accompanied by fever, malaise, joint and muscle pain, and fatigue (Lisnevskaia *et al*, 2014). SLE pathogenesis is associated with autoantibody production against a wide variety of endogenous antigens (Yaniv *et al*, 2015; Tsokos *et al*, 2016). Most autoantibodies in SLE are high‐affinity immunoglobulin G class (IgG) indicating production by self‐reactive B cells that have undergone somatic hypermutation and class switching driven by self‐reactive T helper (Th) cell interactions (Baudino *et al*, 2006; Suurmond & Diamond, 2015). Th cells reacting to self‐antigens have been directly implicated in SLE and SLE‐like models and thus are a potential target for SLE treatments (Crispin *et al*, 2010; Dong *et al*, 2011). However, therapeutically targeting autoreactive Th cells is challenged by difficulties in discriminating pathogenic self‐reactive from non‐self‐reactive Th cells (Comte *et al*, 2015).

B cell activation and maturation are spurred by interactions with follicular helper T (Tfh) cells in the germinal centers (GC) of lymphoid organs and Tfh cell dysregulation is associated with the development of aberrant B cell responses and systemic autoimmunity (Vinuesa *et al*, 2009; Craft, 2012; Ueno *et al*, 2015; Ueno, 2016). Tfh cells are identified by co‐expression of PD‐1 and CXCR5; PD‐1 plays a key role in Tfh cell activation (Shi *et al*, 2018) and CXCR5 allows Tfh cells to migrate to B cell follicles in lymphoid tissue (Crotty, 2019). Tfh cells secrete interleukin (IL)‐21, a critical cytokine controlling B cell maturation and efficient antibody production (Ozaki *et al*, 2002). In addition to Tfh cells, populations of PD‐1^+^ Th cells lacking CXCR5 expression may also contribute to systemic autoimmunity (Rao *et al*, 2017). These cells, termed peripheral helper T (Tph) cells, are expanded in SLE and share features with Tfh cells including *maf* expression and IL‐21 production (Bocharnikov *et al*, 2019). Some reports have shown that Tph are more important for driving autoantibody production than classical Tfh cells (Makiyama *et al*, 2019), although their relative contributions to pathogenicity in SLE remain unclear.

Male BXSB.Yaa mice develop a severe spontaneous SLE‐like disease characterized by lymphoid hyperplasia, monocytosis, hyper antibody production, including autoantibodies such as anti‐nuclear antibodies (ANA), and immune complex‐mediated glomerulonephritis. As this disease is enhanced by the presence of the Yaa locus (Y chromosome‐associated autoimmune acceleration locus that includes duplication of *tlr7*), pathogenic self‐responses predominately occur in male mice, although mild disease develops later with age in female mice (Murphy & Roths, 1979). It is known that BXSB.Yaa disease requires IL‐21 and Th cells, in particular ICOS‐expressing Tfh cells that produce IL‐21, and are critical for the development of SLE‐like disease in BXSB mice (Chu *et al*, 1996; Bubier *et al*, 2007, 2009; Li *et al*, 2011).

Our previous studies have associated the expression of *Nr4a2* with autoreactive Th cells in patients with the autoimmune disease multiple sclerosis and its mouse model (Doi *et al*, 2008; Raveney *et al*, 2013). Using RNA interference treatment and generating transgenic *Nr4a2* cKO mice with a T‐cell‐specific *Nr4a2* deficiency, we reported that NR4A2 was required for Th17 cell secretion of IL‐21, which in turn promoted autoimmune disease (Raveney *et al*, 2013, 2015). Furthermore, only priming with self‐peptide and not with foreign antigens induced aberrant expression of *Nr4a2* in Th cells hinting at a specific association between NR4A2 and autoreactive Th responses (Raveney *et al*, 2013).

In this study, we have shown that male BXSB.Yaa mice lacking *Nr4a2* in T cells were protected from SLE‐like disease with reduced self‐reactive Th cells. Systemic responses to foreign antigens remained intact in the absence of *Nr4a2*, suggesting that NR4A2 may preferentially control self‐reactive immune responses. Research into Th cells in SLE to date focused on Tfh and Tph as pathogenic subsets, which both express PD‐1; we now reveal a new cell subset expressing NRP1 as well as PD‐1 that transcends these classic subsets. These non‐regulatory (conventional) NRP1‐expressing Th cells intrinsically bear self‐reactive TcRs and act to generate SLE‐like disease. Therapeutic targeting of NRP1‐expressing cells ameliorated disease indicators, giving future hope for a new approach in treating Th‐cell‐mediated systemic autoimmune disease, especially as human SLE patients were found to also have increased NRP1‐expressing Th cells.

## Results

### T‐cell‐specific deletion of the *Nr4a2* gene abrogates systemic autoimmunity in BXSB.Yaa mice

Male BXSB.Yaa mice develop a spontaneous SLE‐like disease, resulting from responses against a range of self‐antigens. Thus, this model may be more representative of the natural disease course of systemic autoimmunity than many artificial autoimmune models induced against a single antigen. The disease manifests with extreme lymphocyte expansion and enlarged secondary lymphoid organs as well as high levels of pathogenic self‐reactive antibodies (Andrews *et al*, 1978; Merino *et al*, 1992). Development of systemic autoimmunity in BXSB.Yaa mice requires IL‐21 produced by Th cells to influence self‐reactive B cell differentiation (Bubier *et al*, 2007, 2009).

**Figure 1:**
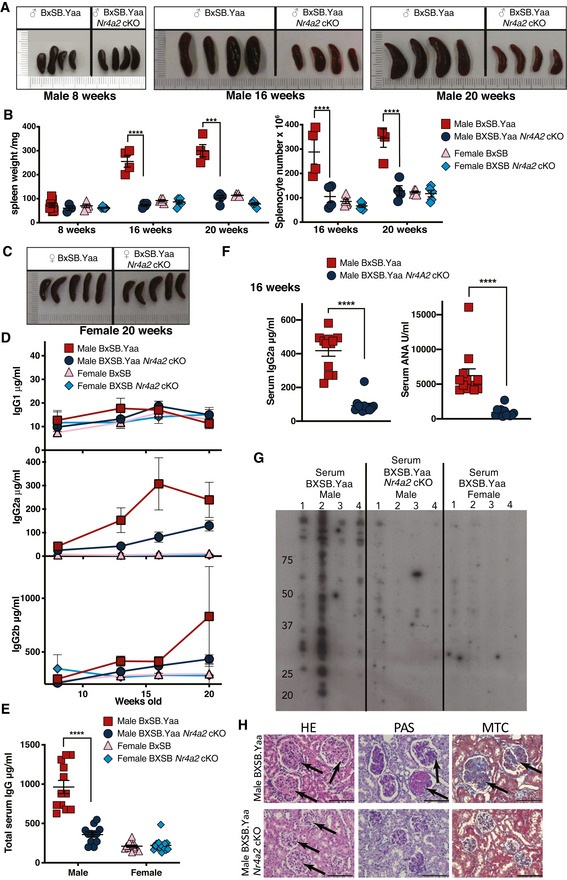
T‐cell‐specific deletion of the Nr4a2 gene abrogates systemic autoimmunity in BXSB mice Cre‐CD4.*Nr4a2* ^fl/fl^ mice backcrossed with BXSB.Yaa mice were maintained under SPF conditions until the time point indicated.
A, BSpleens from co‐housed littermate male *Nr4a2* ^fl/fl^ BXSB.Yaa and Cre‐CD4 *Nr4a2* ^fl/fl^ BXSB.Yaa (BXSB.Yaa *Nr4a2* cKO) mice were excised, photographed (A), and size measured by weight and total cell number (B). Error bars, SEM for individual mice (*n* = 4); representative of at least 10 independent experiments, 4–10 per group; ****P < 0.0001, ***P < 0.001. Two‐way ANOVA test with Bonferroni's multiple comparison test.CRepresentative photographs from 20‐week‐old female BXSB.Yaa mice are shown.D–FSerum prepared from 16‐week‐old male and female BXSB.Yaa or BXSB.Yaa *Nr4a2* cKO mice was assessed by ELISA at the indicated time points for IgG1, IgG2a, and IgG2b (D), comparing total IgG levels from male and female mice aged 16 weeks (E) and IgG2a and IgG anti‐nuclear antibody (ANA) for male mice at 16 weeks (F). Time courses in (D) show a representative experiment, *n* = 4 per group and individual mice from three pooled independent experiments in (E, F), *n* = 12 per group; (E) ****P < 0.0001, Two‐tailed unpaired *t*‐test with Welch's correction; (F) ****P < 0.0001, Two‐tailed Mann–Whitney U test. Error bars, SEM.GLiver proteins were resolved by PAGE and the binding of serum antibodies from four individual mice from each group (numbered 1–4) was detected by anti‐IgG secondary antibody.HKidney histology of male BXSB.Yaa mice and BXSB.Yaa *Nr4a2* cKO mice aged 24 weeks for H&E, black arrows indicate glomeruli which are more enlarged and inflamed in male BXSB.Yaa mice; periodic acid/Schiff reagent (PAS), arrows indicate pink staining showing glomerular deposition of extracellular matrix components; or Masson's trichrome (MTC), arrows indicate blue staining showing collagen deposition. The black scale bar represents 100 μm, data are representative of at least six individual experiments.
Source data are available online for this figure.

**Figure EV1:**
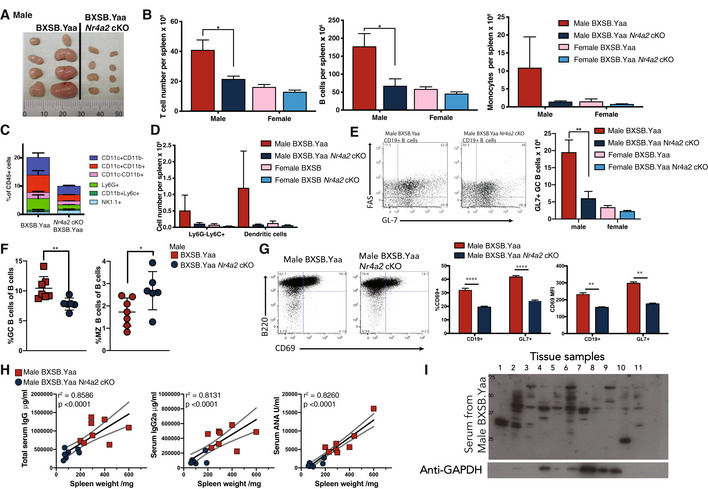
T‐cell‐specific deletion of the *Nr4a2* gene abrogates systemic autoimmunity in BXSB mice AInguinal lymph nodes from 16‐week‐old male BXSB.Yaa or BXSB.Yaa *Nr4a2* cKO mice were photographed.B–GSpleens from male and female mice were disrupted and cell numbers were counted by flow cytometry: TcRb^+^ T cells (B, left), CD19^+^ B cells (B, center), and scatter gated CD45^+^CD19^−^TcR^−^ NK1.1^−^ monocytes (B, right). Non‐T and ‐B cells from male mice were further fractionated by staining with CD11b, CD11c, Ly6G, Ly6C, as well as NK1.1 (C). CD45^+^CD11b^+^Ly6G‐Ly6C^+^ cells (Ly6G‐Ly6C^+^) and CD45^+^CD11c^+^ (dendritic cells) were also assessed from male and female mice (D). Germinal center B cell (CD19^+^B220^+^ FAS^+^GL7^+^, GC B cells) numbers per spleen were also assessed: representative flow cytometry staining is shown (E, left), GC cell numbers for individual mice (E, Right) and GC B cells and CD19^+^B220^+^CD21^−^CD23^+^ marginal zone (MZ) B cells measured as a proportion of total CD19^+^ B cells (F). B cell activation as measured by CD69 flow cytometry in total CD45^+^TcR^−^CD19^+^ B cells (CD19^+^) and CD45^+^TcR^−^CD19^+^GL7^+^ B cells (GL7^+^), representative flow cytometry staining is shown (G, left), CD69^+^ cell populations as a percentage of parent cells (G, center), and CD69 mean fluorescent intensity (MFI) of cell populations (G, right). *n* = 4–8 mice per group and data represent 3–6 similar experiments. Error bars represent SEM for individual mice; *n* = 4–7; *P < 0.05, **P < 0.01, ***P < 0.001, ****P < 0.0001 unpaired two‐tailed Student's *t*‐test with Welch's correction. Data are representative of at least 10 independent experiments with 4–10 per group.HSerum concentrations of antibodies (Y‐axis) was compared with spleen weight (X‐axis) from 16‐week‐old male BXSB.Yaa and male BXSB.Yaa *Nr4a2* cKO mice; for total IgG (left), IgG2a (center), and ANA (right). *n* = 8 mice per group; linear Spearman *R* correlation test; regression analysis (solid line) shown with 95% confidence intervals (dotted lines).IIgG serum antibody binding to PAGE‐resolved proteins isolated from 11 tissues from 16‐week‐old male BXSB.Yaa mice. Tissues are as follows: 1: salivary glands, 2: liver, 3: lung, 4: heart, 5: stomach, 6: kidney, 7: musele, 8: brain, 9: spinal cord, 10: small intestine, 11: large intestine.

NR4A2 controls IL‐21 secretion by Th cells in a model of organ‐specific autoimmunity (Raveney *et al*, 2013). To determine whether or not NR4A2 also plays an essential role in IL‐21‐dependent systemic autoimmune disease, we generated BXSB.Yaa mice with a *Nr4a2* gene deletion in T cells. Backcrossing our previously reported CD4‐Cre^Tg/+^ *Nr4a2* ^fl/fl^ mice (Raveney *et al*, 2015) to the BXSB.Yaa background provided BXSB.Yaa mice both with a T‐specific *Nr4a2* deficiency (Cre‐CD4^+/−^ *Nr4a2* ^fl/fl^ BXSB.Yaa, BXSB.Yaa *Nr4a2* cKO) and *Nr4a2*‐sufficient littermates (*Nr4a2* ^fl/fl^ BXSB.Yaa, BXSB.Yaa mice). Male BXSB.Yaa mice developed severe splenomegaly by 16–20 weeks of age similar to wild‐type male BXSB.Yaa mice, while BXSB.Yaa *Nr4a2* cKO littermates were significantly protected from lymphoid expansion (Figs 1A–C and EV1A). Male mice lacking NR4A2 were also similar to female BXSB mice that lack the Yaa locus and do not develop the disease (Murphy & Roths, 1979; Merino *et al*, 1992), maintaining normal spleen weight and cellularity (Fig 1B and C).

Splenomegaly in Male BXSB.Yaa mice over 16 weeks of age with intact NR4A2 was associated with increased numbers of a variety of major immune cell populations, including T cells, B cells, and monocyte populations (Fig EV1B–D). This cell expansion was significantly suppressed in Male BXSB.Yaa *Nr4a2* cKO mice, with cell populations remaining similar to pre‐disease levels and to female BXSB mice. Not only were B cells generally expanded in Male BXSB.Yaa mice, but GL7^+^ germinal center (GC) B cells were specifically expanded and the proportion of CD21^+^CD23^low^ marginal zone (MZ) B cells was reciprocally decreased (Fig EV1E and F). These changes that are indicative of disease induction in SLE‐like disease were blocked in male BXSB.Yaa *Nr4a2* cKO mice. Instead, male BXSB.Yaa *Nr4a2* cKO mice maintained pre‐disease B cell proportions with significantly lower levels of B cell activation than diseased mice as indicated using CD69 level (Fig EV1G). The differences observed in non‐T cells, despite the *Nr4a2* deletion being T‐cell‐specific, suggest that the expansion of these populations results from T‐cell‐related changes.

Autoimmune pathology in male BXSB.Yaa mice is generated by the production of autoantibodies in a similar manner to human SLE (Izui *et al*, 2000; Arbuckle *et al*, 2003). As B cell expansion, activation, and switching to GC B cells were blocked by T‐cell‐specific deletion of *Nr4a2*, we wished to measure the effects on autoantibody production. Male BXSB.Yaa mice had high IgG serum levels after disease onset, consisting mostly of IgG2a and IgG2b, but no such antibody response developed in Male BXSB.Yaa *Nr4a2* cKO mice or littermate female BXSB mice (Fig 1D and E). In particular, IgG2a and ANA, key pathogenic effector autoantibodies in SLE, were strongly accumulated in Male BXSB.Yaa mice with the disease, but not in Male BXSB.Yaa *Nr4a2* cKO mice (Fig 1F). Interestingly, these serum levels of ANA, IgG2a, and total IgG correlated significantly with spleen size (Fig EV1H).

Although ANA are representative autoantibodies, SLE involves diverse autoantibody responses against a wide variety of self‐antigens. To explore this further, we measured the reactivity of serum IgG from 16‐week‐old male BXSB.Yaa mice by immunoblotting against protein lysates extracted from 11 different male BXSB.Yaa self‐organs (Fig EV1I). In particular, serum from male BXSB.Yaa mice bound to multiple liver proteins, while serum derived from male BXSB.Yaa *Nr4a2* cKO mice or female mice was less reactive (Fig 1G). Progressive glomerulonephritis caused by the renal accumulation of immune complexes is a typical feature of the disease in BXSB.Yaa mice (Andrews *et al*, 1978). Multiple pathologies were observed in the kidney of 16‐week‐old male BXSB.Yaa mice, including glomerular basement membrane hyperplasia, strong perivascular inflammation, focal glomerular fibrosis, and glycoprotein deposition, while these features were milder in male BXSB.Yaa *Nr4a2* cKO littermates (Fig 1H).

Together our data show a T‐cell‐specific deletion of the *Nr4a2* gene thoroughly blocked onset of SLE‐like disease hallmarks in Male BXSB.Yaa mice, suggesting that NR4A2‐expressing Th cells are intrinsically involved in the development of autoreactive responses in this disease.

### Rapid expansion of diverse Th cell subsets in male BXSB.Yaa mice is NR4A2 dependent

**Figure 2:**
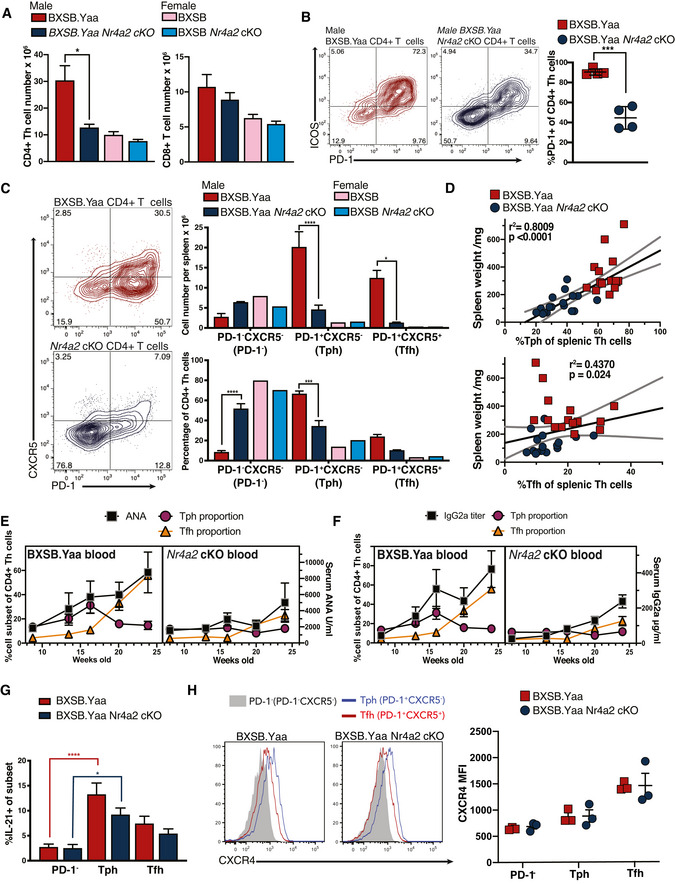
Rapid expansion of diverse Th cell subsets in BXSB.Yaa mice is Nr4a2 dependent A–CSplenocytes from 16‐week‐old male and female BXSB.Yaa or BXSB.Yaa *Nr4a2* cKO littermate mice were assessed by flow cytometry for CD4^+^ Th (TcRβ^+^CD4^+^CD8^−^) and CD8^+^ Tc (TcRβ^+^CD4^−^CD8^+^) cell number per spleen (A); PD‐1/ICOS among TcRβ^+^CD4^+^ cells (representative staining and PD‐1^+^ proportion, male mice, B), representative PD‐1/CXCR5 staining for TcRβ^+^CD4^+^ cells (C, left) to determine PD‐1^−^ (PD‐1^−^CXCR5^−^), Tph (PD‐1^+^CXCR5^−^), and Tfh (PD‐1^+^CXCR5^+^) cell subsets and measure mean cell number of each subset per spleen (C, right upper) and proportion among TcRβ^+^CD4^+^ T cells (C, right lower). Error bars, SEM for individual mice, *n* = 4. Data are representative of at least five independent experiments with 4–10 mice per group; ****P < 0.0001, ***P < 0.001, and *P < 0.05 two‐way ANOVA test with Bonferroni's multiple‐comparison test comparing *Nr4a2* cKO effect for each subset.DFor individual mice, Tph and Tfh cells' cell percentage in splenic Th cells was compared with spleen weight; linear regression lines shown with 95% CI; *n* = 15 per group across three independent experiments; Spearman R correlation statistics indicated.E, FSerum antibody levels for male BXSB.Yaa and male BXSB.Yaa *Nr4a2* cKO at the indicated times were compared with Th cell subsets. The left axes indicate Tph or Tfh cell percentage; the right axes indicate serum ANA (E) or IgG (F) level. *n* = 8 per group, representative of three independent experiments; error bars, SEM.GIL‐21 was measured in splenocytes from 16‐week‐old male mice by intracellular flow cytometry following restimulation PD‐1^−^, Tph, and Tfh subsets *n* = 5; data are representative of two independent experiments; error bars, SEM, ****P < 0.0001, *P < 0.05, two‐way ANOVA test with Bonferroni's multiple‐comparison test.HCXCR4 expression was measured by flow cytometry for each subset. Representative CXCR4 staining (left) and geometric mean fluorescent intensity (MFI) for each group (right) are shown. *n* = 3 individual mice; data are representative of three similar experiments, error bars, SEM.

**Figure EV2:**
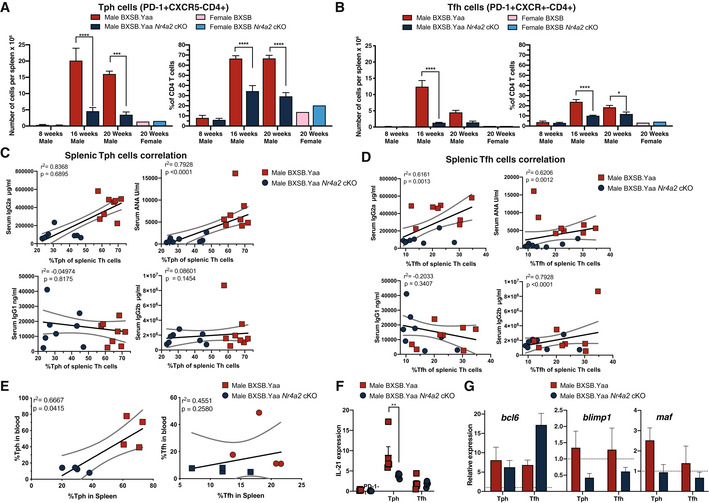
Rapid expansion of diverse Th cell subsets in BXSB mice is NR4A2 dependent A–ESpleen cell suspensions from 8‐ to 20‐week‐old male and 16‐week‐old female BXSB.Yaa and BXSB.Yaa *Nr4a2* cKO mice were evaluated for populations within TcRβ^+^CD4^+^ Th cells by cell number per spleen and proportion among Th cells for Tph (PD‐1^+^CXCR5^−^) cells (A) and Tfh (PD‐ 1^+^CXCR5^+^) cells (B). Data are representative of at least 4–10 independent experiments with 4–10 mice per group; error bars, mean with SEM; *P < 0.05, ***P < 0.001, ***P < 0.001 two‐way ANOVA with Bonferroni's multiple‐comparison test. For individual mouse, serum IgG2a, ANA, IgG1, and IgG2b level was compared with subset percentage in splenic Th cells for Tph cells (C) and Tfh cells (D). Linear regression lines shown with 95% CI; statistical testing by Spearman *R* test indicated; *n* = 8 per group across 2 independent experiments. For 16‐week‐old male mice, Th cell subset proportions in spleens (X‐axis) were compared with subset proportion in blood (Y‐axis) among whole Th cells for Tph cells (E, left) and Tfh cells (E, right). *n* = 4; Spearman *R* correlation test; linear regression analysis lines shown with 95% confidence intervals.F, GSplenic Th cells from 16‐week‐old male BXSB.Yaa and BXSB.Yaa *Nr4a2* cKO mice purified by flow cytometric cell sorting into PD‐1^−^, PD‐1^+^CXCR5^−^ (Tph), and PD‐1^+^CXCR5^+^ (Tfh) cells and gene transcription for *il21* (F); **P < 0.01 two way ANOVA with Bonferroni's multiple‐comparison test, and *bcl6*, *blimp1*, and *maf* (G) were assessed by real‐time qPCR. Relative transcript levels were normalized to PD‐1^−^ cells (PD‐1^−^ cells were accorded level 1.0, indicated by dotted lines), *n* = 4 mice per group; error bars, mean with SEM; data are representative of at least three similar experiments.

SLE‐like disease in male BXSB.Yaa mice has been associated with Tfh cells (Bubier *et al*, 2009), therefore we further scrutinized T cells to determine if NR4A2 had effects on particular subsets. NR4A2‐dependent T cell expansion was mainly attributed to CD4^+^ Th cells, with little increase in CD8^+^ Tc cells (Fig 2A). Ninety percent of Th cells in 16‐week‐old male BXSB.Yaa mice expressed PD‐1, a surface receptor found on Tfh cells, versus < 50% in male BXSB.Yaa *Nr4a2* cKO mice (Fig 2B). The number of cells in this fraction co‐expressing the Tfh‐related chemokine receptor CXCR5 was also greater in male BXSB.Yaa mice. However, PD‐1^+^ Th cells lacking CXCR5 co‐expression were also significantly increased in male BXSB.Yaa spleens (Fig 2C). A similar population of PD‐1^+^CXCR5^−^ extrafollicular Th cells, termed Tph cells, have been reported as driving autoantibody production in a mouse model of rheumatoid arthritis and in human SLE (Odegard *et al*, 2008; Rao *et al*, 2017; Lin *et al*, 2019; Makiyama *et al*, 2019). In male BXSB.Yaa, we find that such Tph cells predominate over Tfh cells at the onset of systemic autoimmunity and are maintained throughout the disease, yet are virtually absent in young male BXSB.Yaa mice and female BXSB mice (Fig EV2A and B). Interestingly, the splenic Tph cell level correlated with splenomegaly more significantly than Tfh cells (Fig 2D) and a similar finding for Tph correlation with serum levels of ANA and IgG2a, but IgG1 and IgG2b had no correlation with either cell subset (Fig EV2C and D). Th cell subsets in blood had different kinetics with blood Tfh cells increasing at a later time point, whereas the increase in blood Tph cells occurred in early disease and mirrored increases in serum ANA and total IgG2a levels (Fig 2E and F). In male BXSB.Yaa *Nr4a2* cKO mice, circulating Tfh and Tph cell expansion was abrogated and ANA production and increases in IgG2a titer were suppressed at all time points. The blood level of Tph cells correlated well with the level in the spleen (Fig EV2E), but for Tfh cells, correlation was lower between sites, presumably as CXCR5 expression targets Tfh cells to the spleen. Splenic Tph and Tfh cells both produced IL‐21 on stimulation, but Tph had a significantly higher proportion of IL‐21^+^ cells (Fig 2G). IL‐21 producers were significantly reduced in male BXSB.Yaa *Nr4a2* cKO mice, particularly in the Tph subset. Similar findings were noted at the mRNA level for *Il21* (Fig EV2F). Transcripts of Tfh‐related markers, including the key transcription factor *Maf* and also *Blimp1*, were similar for Tfh and Tph cells, but with high expression compared with PD‐1^−^ cells and reduced level in male BXSB.Yaa *Nr4a2* cKO mice (Fig EV2G).

It is somewhat surprising that disease manifestations were more closely linked to Tph cells rather than Tfh cells, as previous reports highlighted a close link between ICOS/CXCR5 expression in Th cells and spleen cell number in male BXSB.Yaa mice. It has been reported that high‐affinity autoantibody production can be activated extrafollicularly, with B cells interacting with CXCR4^−^‐expressing Th cells rather than Tfh cells in GC (Odegard *et al*, 2008). Both Tfh cells and Tph cells expressed CXCR4 to a similar level indicating that CXCR4 expression was independent of NR4A2 (Fig 2H).

### Humoral responses against exogenous antigens are independent of NR4A2

**Figure 3:**
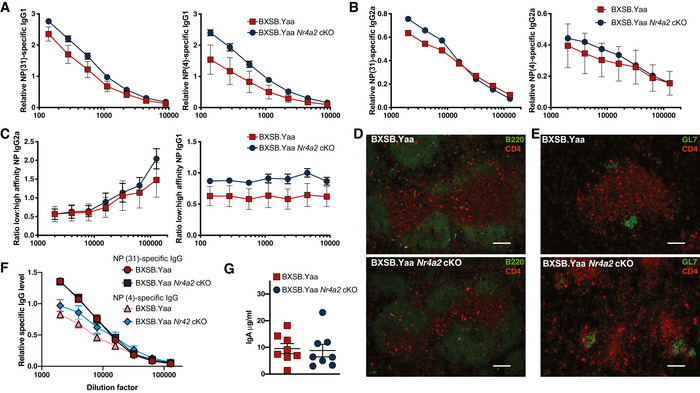
Humoral responses against exogenous antigens are independent of Nr4a2 A–CYoung male BXSB.Yaa and BXSB.Yaa *Nr4a2* cKO mice were immunized twice with NP‐KLH emulsified in alum. Serum was assessed for the level of antibodies binding NP‐31 (left) and NP‐4 (right) epitopes by ELISA across a range of diluted serum samples with IgG1‐specific (A) and IgG2a‐specific antibodies (B). The ratio for low‐ to high‐affinity (NP‐4:NP‐31) antibodies was calculated for IgG2a (left) and IgG1 (right) antibodies (C). Error bars, SEM, *n* = 5 mice per group; *P* > 0.1 two‐way ANOVA.D–FMale C57BL/6 Nr4a2 cKO or wild‐type littermate C57BL/6 mice were immunized twice with NP‐KLH emulsified in alum. After 2 weeks, 7 μm spleen sections were stained with Red:Alexa‐Fluor‐594‐CD4 and Green: FITC‐B220 (D) or FITC‐GL7 (E). The scale bar represents 100 nm. The serum was assessed for the level of IgG antibodies binding NP‐31 and NP‐4 (F).GStool samples from young male BXSB.Yaa and BXSB.Yaa *Nr4a2* cKO mice were measured for IgA concentration by ELISA. Points are samples from individual mice, error bars, SEM, *n* = 8 from two independent experiments; *P* > 0.1 unpaired Student's *t*‐test.

Reduced autoantibody production in male BXSB.Yaa *Nr4a2* cKO mice might result from a global defect in Th cell responses which in turn limits B cell help. It has been reported that IgG responses against the exogenous antigen can be stimulated separately to autoantibody production (Niu *et al*, 2008). Therefore, we tested if the defective T‐cell‐dependent antibody production we observed in spontaneous SLE‐like disease was also reconstituted during T‐cell‐mediated priming of antibody responses to exogenous antigens in the absence of NR4A2. High‐ and low‐affinity NP‐specific IgG1and IgG2a responses resulting from NP‐KLH immunization were NR4A2 independent (Fig 3A and B). Furthermore, there was no difference in ratio between high‐ and low‐affinity antibodies against NP moiety with/without *Nr4a2* indicating somatic hypermutation occurred to the same extent (Fig 3C). NP‐KLH immunization of mice on a C57BL/6 background, which do not develop spontaneous autoimmune disease, led to similar levels of Th cells (red) entering splenic B cell follicles (green, Fig 3D; Appendix Fig S1A) with/without *Nr4a2* in T cells and similar formation of germinal centers within lymphoid follicle structures (Green, Fig 3E; Appendix Fig S1B). Furthermore, anti‐NP antibody levels induced by the NP‐KHL immunization were not reduced in the absence of *Nr4a2* (Fig 3F). As another measure of Th‐dependent humoral responses to exogenous antigen, we measured fecal IgA levels, which are produced in responses to bacterial antigens (Kawamoto *et al*, 2012; Cao *et al*, 2015); these did not differ between C57BL/6 WT and C57BL/6 *Nr4a2* cKO mice (Fig 3G).

These data indicating normal antibody production in the absence of NR4A2 contrasts with our findings showing reduced autoantibody production in male BXSB.Yaa *Nr4a2* cKO mice. Taken together, T‐cell‐dependent humoral responses against exogenous antigens may be intrinsically distinct from autoantibody production and with NR4A2 exclusively required for T‐cell‐dependent IgG production against self‐antigen.

### Self‐reactive T cell pool is associated with NR4A2‐dependent Th cells

Our data suggest that NR4A2 influences pathogenic responses to self‐antigen in male BXSB.Yaa mice by controlling self‐reactive Th cells. T cells escaping central tolerance can allow peripheral expression of T cell receptors (TcR) specific for self‐antigen that are potentially autoreactive (Yu *et al*, 2015). To date, research into T cells that respond to self‐antigens has been hampered by an inability to discern such self‐reactive T cells from the useful T cell pool. However, a recent study highlighted a TcR feature linked to self‐reactivity: hydrophobic amino acid doublets in positions 6 and 7 (P6‐7) in the CDR3 region of a TcR were associated with promoting self‐reactivity, while hydrophilic P6‐7 doublets limit self‐reactivity (Stadinski *et al*, 2016). Hydrophobic TcR CDR3 apexes were also associated with self‐reactive TcRs in mouse regulatory cells and TcRs with this attribute were also higher in human subjects with RAG mutations with resulting increase in self‐reactive T cells (Daley *et al*, 2019). As we wished to examine potential self‐reactive T cells, we performed TcR CDR3 repertoire sequencing of memory Th cells from male BXSB.Yaa mice to identify T cell clones with these hallmarks of self‐reactivity.

**Figure 4:**
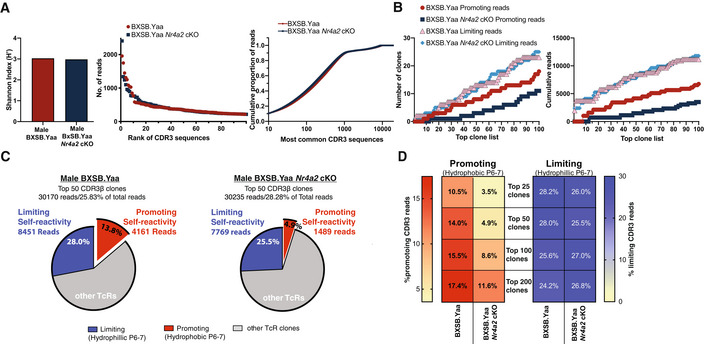
Expanded Th cell clones with hallmarks of TcRs associated with promoting self‐reactivity are reduced by *Nr4a2* deletion ASplenic memory T cells (TcRb^+^CD4^+^CD44^hi^CD62L^lo^) sorted from 20‐week‐old male BXSB.Yaa and BXSB.Yaa *Nr4a2* cKO were sequenced for unbiased TCR repertoire analysis. Unique TcR sequences were ordered by most common clones with summary diversity statistics calculated and the cumulative read proportion across all unique sequences ranked by most common CDR3 is shown for each genotype.BSequences were categorized based on residues CDR3 positions 6 and 7: hydrophobic P6/7 doublets (promoting self‐reactivity) and hydrophilic doublets (limiting self‐reactivity). Unique TcR sequences were ordered by most common clones based on read number, and the proportion of promoting and limiting self‐reactivity clones for the top 100 clones of each genotype is shown by the number of clones (left) and cumulative reads (right).C, DThe charts in (C) show the proportion of promoting and limiting self‐reactivity clones for each genotype for the top 50 clones, and the proportion of promoting and limiting self‐reactivity clones for the top 25, 50, 100, and 200 most common sequences by read number is shown by the heat map in (D). Source data are available online for this figure.

Male BXSB.Yaa and BXSB.Yaa *Nr4a2* cKO mice had extensive and similar TcR diversity with a similar distribution of TcR clones (Fig 4A). Clones were ranked by read number for each genotype and the CDR3 sequence was assessed for self‐reactivity limiting/promoting hallmarks (Appendix Table S1). The most common TcRβ CDR3 sequences with the highest read numbers represent the most expanded Th clones, therefore our investigation focused on such clones as these would represent the most relevant Th cell repertoire for ongoing disease. For example, the top 50 clones, represented at least 25% of the memory cell pool (Fig 4B). Self‐reactive promoting TcRs among these 50 most common clones were greatly reduced in male BXSB.Yaa *Nr4a2* cKO mice (Fig 4C). Similar results were observed across the top 25, 100, and 200 clones (covering ~ 15, 40, and 50% of repertoire reads, respectively), with reduced self‐reactive promoting clones in BXSB.Yaa *Nr4a2* cKO mice, but similar levels of limiting clones between genotypes (Fig 4D). These data indicate a selective loss of self‐reactive T cells from the periphery in the absence of NR4A2.

### Disease‐associated PD‐1^+^ Th cells share markers with regulatory T cells

The detection of self‐reactive cells by cell‐intrinsic hallmarks is somewhat of a “holy grail” in autoimmune research and using the properties of TcR CDR3 residues gives tantalizing hints at resolving this issue. To corroborate our finding that self‐reactive Th cells were reduced in male BXSB.Yaa *Nr4a2* cKO mice, we looked to another T cell population that responds to self‐peptides for potential self‐reactive T cell hallmarks: regulatory T (Treg) cells (Hsieh *et al*, 2006). Interestingly, Treg cells also upregulate PD‐1 (Francisco *et al*, 2009), thus we investigated if other surface markers were also shared between Treg and pathogenic PD‐1^+^ Th cells.

**Figure EV3:**
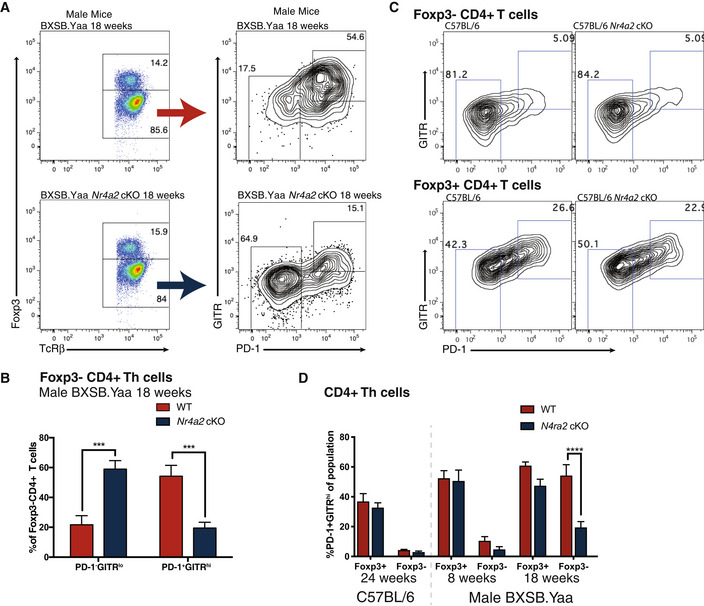
Foxp3^−^ non‐Treg Th cells subset expanded in diseased male BXSB.Yaa mice shares markers with Foxp3^+^ Treg cells A, BSingle‐cell suspensions from spleens of male BXSB.Yaa or male BXSB.Yaa Nr4a2 cKO mice aged 18 weeks were stained with surface antibodies against TcRβ, CD3, PD‐1, and GITR before intracellular staining with anti‐Foxp3. Foxp3 staining was used to select Foxp3^−^ conventional Th cells and GITR/PD‐1 levels were assessed. Representative staining is shown (A) with the percentage of conventional PD‐1^−^GITR^lo^ and PD‐1^+^GITR^hi^ Th cells shown for Foxp3^−^ cells (B).***P < 0.001; Two‐way ANOVA with Bonferroni's multiple comparisons test; *n* = 3–5 mice per group; error bars are SEM. These data are representative of three independent experiments.CSingle ‐ell suspensions from spleens of wild‐type C57BL/6 or *Nr4a2* cKO C57BL/6 mice aged 24 weeks were stained and examined by flow cytometry and Foxp3 was used to gate T cells into Foxp3^+^ and Foxp3^−^. GITR vs. PD‐1 expression in CD4^+^TcRβ^+^ T cells is shown for Foxp3^−^ and Foxp3^+^ These data are representative of three independent experiments (*n* = 2–4 mice per group);DSingle‐cell suspensions from spleens of wild‐type C57BL/6 or *Nr4a2* cKO C57BL/6 aged 24 weeks or male BXSB.Yaa or male BXSB.Yaa Nr4a2 cKO aged either 8 or18 weeks were stained with surface antibodies against, TcRβ, CD3, PD‐1, and GITR, before intracellular staining with anti‐Foxp3. Foxp3 staining was used to select conventional Th cells and Treg cells. The percentage PD‐1^+^GITR^hi^ Th cells is shown for Foxp3^−^ conventional Th cells and Foxp3^+^ Treg cells. *n* = 4–6 mice per group error bars, mean with SEM; ****P < 0.0001 two‐way ANOVA with Bonferroni's multiple‐comparison test.

Glucocorticoid‐induced TNFR‐related protein (GITR, also known as CD357 and TNFRSF18) has previously been reported as a marker of Treg cells (McHugh *et al*, 2002; Shimizu *et al*, 2002) and in particular, PD‐1^+^GITR^hi^ Treg subsets have been reported to include T cell populations with self‐reactive TcRs (Wyss *et al*, 2016). We measured GITR expression in Th cells subsets in male BXSB.Yaa mice after disease onset using the hallmark Treg transcription factor Foxp3 to separate non‐Treg conventional Th cells from Treg cells. We observed high numbers of PD‐1^+^GITR^hi^ cells among conventional non‐Treg cells, as defined by lack of Foxp3 expression (Fig EV3A and B). This expansion of Foxp3^−^PD‐1^+^GITR^hi^ Th cells was NR4A2 dependent and appeared to be associated with the development of self‐reactive responses as few PD‐1^+^GITR^hi^ were observed among conventional non‐Treg Th cells in *Nr4a2* cKO littermates (Fig EV3B), or in mice on a background that do not develop spontaneous autoimmune disease (Fig EV3C and D). This expansion of PD‐1^+^GITR^hi^ in male BXSB.Yaa mice was confined to conventional cells as Treg cell numbers and Foxp3^+^ PD‐1^+^GITR^hi^ Treg subsets were similar between genotypes (Fig EV3D).

### NRP1 co‐expression with PD‐1 identifies a Th cell subset that correlates with SLE‐like BXSB.YAA autoimmune disease

**Figure 5:**
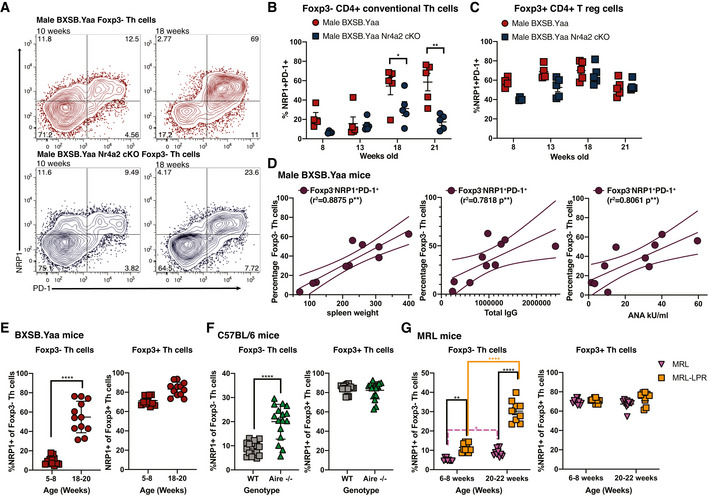
NRP1 expression identifies a Nr4a2‐dependent self‐reactive Th cell subset expanded in SLE A–CSplenocytes from male BXSB.Yaa and BXSB.Yaa *Nr4a2* cKO were assessed by flow cytometry for NRP1 and PD‐1 expression in Foxp3^+^ or Foxp3^−^ CD4^+^TcRβ^+^ subsets: representative staining at 10 and 18 weeks old (A), proportions of NRP1^+^PD‐1^+^ cells at 8–21 weeks of age in Foxp3^−^ Th cells (B) or Fxop3^+^ Th cells (C). *n* = 5 per group from at least two independent experiments; error bars, SEM; **P < 0.01; *P < 0.05 two‐tailed Mann–Whitney U test.DThe proportion of NRP1^+^PD‐1^+^ cells (Y‐Axis) from splenic TcRb^+^CD4^+^Foxp3^−^ of 18‐week‐old male BXSB.Yaa and BXSB.Yaa *Nr4a2* cKO was compared with (X‐axis) spleen weight (left), and serum levels of IgG (center) and ANA (right). *n* = 8, linear regression line with 95% CVs shown; data are representative of two independent experiments.E–GNRP1‐expressing proportion was measured by flow cytometry among TcRβ^+^CD4^+^Foxp3^−^ Th cells and TcRβ^+^CD4^+^Foxp3^+^ Th cells in mouse strains prone to systemic autoimmunity. Male BXSB.Yaa mice with SLE‐like disease aged 18–20 weeks (*n* = 12) were compared with pre‐disease 5‐ to 8‐week‐old male BXSB.Yaa mice (*n* = 12) (E); Male Aire knock‐out mice (Aire^−/−^; *n* = 16) were compared with male wild‐type mice (WT; *n* = 16), both on a C57BL/6 background and 20–24 weeks (F); Lupus‐prone female MRL‐LPR mice (*n* = 10) were compared with female MRL mice (*n* = 10) at early disease 6–8 weeks and established disease 20‐ to 22‐week‐old MRL.LPR (*n* = 10) with mild disease 20‐ to 22‐week‐old MRL (*n* = 10) (G). For BXSB.Yaa and C57BL/6 background mice, the Foxp3^−^ subset was determined by surface staining of a transgenic reporter, Foxp3^hCD2^; for MRL background mice, the Foxp3^−^ subset was determined by intracellular Foxp3 staining. Error bars, SD ****P < 0.0001, **P < 0.01, *P < 0.05, Two‐tailed unpaired *t*‐test with Welch's correction for BXSB.Yaa and C57BL/6 background plots, and two‐way ANOVA analysis with Bonferroni's multiple‐comparison test. Data are pooled from individual mice from at least two independent experiments.

While our observation of GITR expression on disease‐associated conventional Th cells confirmed our hypothesis that self‐reactive pathogenic and Tregs may share markers, GITR was detected as a shift and so not suitable as a marker of potential self‐reactive Th cells. Therefore, we sought additional receptors usually expressed by Treg cells that might prove more appropriate. Neuropilin‐1 (NRP1; CD304) is a receptor expressed on Treg cells (Bruder *et al*, 2004) that has also been linked with anergic T cells responding to self‐peptide (Kalekar *et al*, 2016). However, NRP1 expression on non‐Treg conventional Th cells has not been extensively studied, but one report links NRP1 with Tfh cells (Renand *et al*, 2013). We now observe that the onset of disease in male BXSB.Yaa mice led to a massive expansion of NRP1^+^ PD‐1^+^ Foxp3^−^ conventional Th cells in the spleen (Fig 5A and B). This expansion of NRP1^+^PD‐1^+^ Th cells was suppressed in the absence of NR4A2 in male BXSB.Yaa mice (Fig 5B), whereas no changes were seen in NRP1/PD‐1 levels among Treg cells in male BXSB.Yaa spleens during disease onset (Fig 5C).

**Figure EV4:**
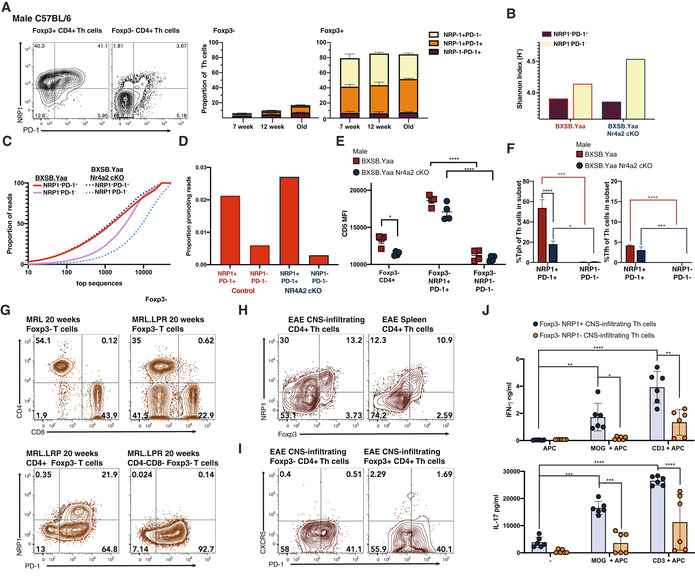
NRP1 expression identifies a *Nr4a2*‐dependent self‐reactive Th cell subset expanded in SLE ASpleens from wild‐type C57BL/6 mice were stained for NRP1 and PD‐1 among TcRβ^+^CD4^+^Foxp3^+^ T cells and TcRβ^+^CD4^+^Foxp3^+^ T cells for flow cytometry. Representative staining from 12‐week‐old mice and proportions of NRP1/PD‐1 subsets from mice aged 7, 12, and over 24 weeks old are shown (A), *n* = 3–8 per representative of least two independent litters; error bars, mean with SD.B–DTcR repertoires were analyzed among NRP1^+^PD1^+^ and NRP1^−^PD‐1^−^ ‐sorted Th subsets from 16‐week‐old male BXSB.Yaa and BXSB.Yaa *Nr4a2* cKO mice. Repertoire diversity was indicated by Shannon index (B) and by calculating the cumulative proportion of the total TcR sequence reads against decreasingly common unique sequences to show distribution skewing (C) and the number of reads for clones with self‐reactivity promoting TcRs was calculated for the top 1,000 clones (D).ECD5 expression was measured by flow cytometry among Foxp3^−^ Th subsets from male BXSB.Yaa and BXSB.Yaa *Nr4a2* cKO mice aged 18 weeks. CD5 MFI is shown for the whole Foxp3^−^CD4^+^ T cells, and NRP1^+^PD1^+^ and NRP1^−^PD‐1^−^ subsets of Foxp3^−^ Th cells; *n* = 5 mice per group, error bars show SEM, *P < 0.05, ****P < 0.0001 two‐way ANOVA with Bonferroni's multiple‐comparison test; data are representative of at least three independent experiments.FFoxp3^−^CD4^+^TcRβ^+^ splenocytes from 18‐week‐old Male BXSB.Yaa and BXSB.Yaa *Nr4a2* cKO mice divided into NRP1^+^PD‐1^+^ and NRP1^−^PD‐1^−^ subsets were assessed for proportions of Tph (left) and Tfh (right) cells based on CXCR5/PD‐1 flow cytometry; *n* = 5 mice per group; data are representative of three independent experiments; error bars show SEM; ****P < 0.0001, ***P < 0.001, *P < 0.05, two‐way ANOVA with Bonferroni's multiple‐comparison test.GSplenocytes from 20‐week‐old female MRL or MRL.LPR mice were stained for flow cytometry and NRP1 and PD‐1 expression was measured for TcRβ^+^CD4^−^CD8^−^ cells. Data are representative of five individual mice per group.H–JEAE was induced in male C57BL/6 Foxp3^hCD2^ reporter mice. On day 17 peak disease, splenocytes and CNS‐infiltrating T cells were isolated and stained for flow cytometry. Representative FACS staining shown for NRP1 versus Foxp3 reporter for CD45^+^CD11b^−^TcRb^+^ Th cells (H) and for CXCR5 and PD‐1 for Foxp3^−^ or Foxp3^+^ NRP1^+^CD45^+^CD11b^−^TcRb^+^ CNS Th cells (I). Pools of three mice were combined and NRP1^+^ and NRP1^−^ Foxp3^−^ Th cells were purified by FACS sorting. Non‐T‐cell fractions were irradiated and used as APC. Th cells and APC were co‐cultured in the presence or absence of 100 μg/ml MOG or 2 μg/ml anti‐CD3. After 96 h, supernatants were collected and cytokine concentrations were measured by ELISA (J). Error bars, SD ****P < 0.0001, **P < 0.01, *P < 0.05 two‐way ANOVA analysis with Bonferroni's multiple‐comparison test. Points show triplicates for two pools of three individual mice. Data are representative of at least two independent experiments with six mice per experiment.

This increase in NRP1 expression in non‐Treg Th cells is not observed in standard mice that are not prone to autoimmunity when the steady state is maintained during aging. In C57BL/6 background mice, NRP1^+^PD‐1^+^ Th cells remained confined to Treg populations with few conventional Th cells expressing NRP1 (Fig EV4A).

Together, these data suggest that the expansion of NRP1^+^ conventional Th cells in male BXSB.Yaa mice is related to the onset of systemic autoimmunity rather than aging and implicates NRP1^+^ Th cells as pathogenic. This is further supported by the level of NRP1^+^PD‐1^+^ conventional Th cells in male BXSB.Yaa, which is strongly correlated with systemic autoimmunity as indicated by splenomegaly and autoantibody production (Fig 5D).

NRP1^+^PD‐1^+^ conventional Foxp3^−^ Th cells from male BXSB.Yaa mice had more restricted repertoires than Th cells that did not express PD‐1, with lower‐sequence diversity and higher coverage ratios of the most frequent TcR CDR3 sequences (Fig EV4B and C). The proportion of self‐reactivity promoting P6‐7 doublets of TcRβ CDR3 was somewhat increased in NRP1/PD‐1^−^‐expressing Th cells over PD‐1^−^ Th cells (Fig EV4D). These differences were apparent in both male BXSB.Yaa and Male BXSB.Yaa *Nr4a2* cKO, thus the reduced self‐reactive T cell population observed in the absence of NR4A2 was due to a differential expansion of self‐reactive Th cells rather than a skewing of TcR receptor selection away from self‐reactive TcRs. The increase in oligoclonality and self‐reactivity promoting clones supports the concept that NRP1^+^PD‐1^+^ cell populations result from expanded self‐reactive cells in disease.

In addition, CD5 expression upregulation, which has been associated with T cells expressing TcRs specific for self (Henderson *et al*, 2015; Zinzow‐Kramer *et al*, 2019), was increased in diseased male BXSB.Yaa mice. With NRP1^+^PD‐1^+^ conventional Th cells having significantly higher CD5 levels as compared with PD‐1^−^ Th cells (Fig EV4E). We also noted that most NRP1^+^ Foxp3^−^ Th cells in male BXSB.Yaa Mice were CXCR5^−^ and so found among the Tph fraction, but some Tfh cells also co‐expressed NRP1 (Fig EV4F); these proportions were decreased in the absence of *Nr4a2*.

Collectively, these data suggested that NR4A2 in T cells allows the expansion of Th cells bearing potentially self‐reactive TcRs, and this expanded subset is linked to NRP1 upregulation.

In addition to NRP1^+^ PD‐1^+^ Th cells, there was also a minor increase in NRP1^−^PD‐1^+^ Th cells in the Foxp3^−^ subset, with slightly higher levels in diseased male BXSB.Yaa mice versus BXSB.Yaa *Nr4a2* cKO mice (Appendix Fig S2A). Whereas NRP1^+^ Th cells correlate well with measures of SLE‐like disease in male BXSB.Yaa mice (Fig 5D), these NRP1^−^PD‐1^+^ Th cells only weakly correlate (Appendix Fig S2B). Over time, there was little expansion of these cells within the Foxp3^−^ fraction (Appendix Fig S2A) versus NRP1^+^PD‐1^+^ conventional Th cells, which were increasing (Fig 5B), supporting the concept that it is NRP1 rather than PD‐1 which is the key marker for pathogenic self‐reactive Th cell in this disease model.

### NRP1‐expressing conventional Th cells are expanded in a range of SLE mouse models

The unusual increase in conventional Th cells expressing NRP1 seen in male BXSB.Yaa mice developing SLE‐like disease was also apparent in other models that are prone to systemic autoimmune diseases (Fig 5E–G). On the C57BL/6 background, NRP1 expression was mostly confined to Treg cells at 20–24 weeks of age in wild‐type mice (Fig 5F). Aire knockout mice (Aire^−/−^), which were developed to study the disease autoimmune polyendocrinopathy‐candidiasis‐ectodermal dystrophy that results from a mutation in AIRE, are prone to mild systemic autoimmune disease symptoms on the C57BL/6 background (Anderson *et al*, 2002). Aire^−/−^ mice had a significant increase in NRP1 expression in conventional Foxp3^−^ Th cells compared with age‐matched wild‐type (WT) C57BL/6 mice (Fig 5F).

Mice on a MRL background develop SLE‐like autoimmune disease with splenomegaly, production of autoantibodies, and increased immune complex formation, leading to severe proliferative glomerulonephritis. A mutation in the Fas gene in MRL.LPR mice leads to lymphoproliferation which greatly accelerates disease, but a slowly developing autoimmune disease also develops in MRL mice without the LRP mutation (Andrews *et al*, 1978; Izui *et al*, 1984; Theofilopoulos & Dixon, 1985). Furthermore, this model has more accelerated disease in females over males (Izui *et al*, 1984) and thus is often used to model human SLE which has a strong female sex bias (Pons‐Estel *et al*, 2010). We observed such a disease in MRL.LPR mice was associated with an early increase in NRP1^+^ Foxp3^−^ Th cells at 6–8 weeks over MRL mice which was greatly enhanced by 20–22 weeks of age (Fig 5G), whereas NRP1 expression in Tregs remained stable. We also observed a slight increase in NRP1^+^ cells among Foxp3^−^ Th cells in MRL mice at 20–22 weeks, which may relate to the onset of the weaker systemic autoimmunity associated with non‐LPR MRL mice (Fig 5G). Interestingly, disease in MRL.LPR is also associated with an increase in unusual CD4^−^CD8^−^ T cells, a feature which leads to the severe lymphadenopathy in this model, but is not observed in human SLE (Morse *et al*, 1982; Cohen & Eisenberg, 1991); we observed that these cells were mostly PD‐1^+^, but only marginal NRP1 upregulation (Fig EV4G).

Overall, it appears that intrinsically self‐reactive Th cells in systemic autoimmune disease may have shared phenotypes with Treg cells, with NRP1^+^ conventional Th cells highly associated with autoimmune disease induction in NR4A2‐dependent manner.

Interestingly, we also detected Foxp3^−^ Th cells that expressed NRP1 in the target organ during experimental autoimmune encephalomyelitis (EAE), an induced mouse model of autoimmune neuroinflammatory disease that is used as an analog of human multiple sclerosis. Here, Th responses are induced by immunization of C57BL/6 mice with the CNS antigen MOG (myelin oligodendrocyte glycoprotein), leading to infiltration of the CNS with pathogenic Th cells that generate local damage. At peak disease, a significant proportion of CNS‐infiltrating Foxp3‐ Th cells had acquired NRP1 expression, making up a third of all CNS‐infiltrating Th cells (Fig EV4H). Unlike in systemic autoimmune disease, these NRP1^+^ Th cells did not express PD‐1 (Fig EV4I), suggesting NRP1 expression in autoimmune disease is not only limited to Tph and Tfh cells. Indeed, B cell responses are not required in EAE pathogenesis (Lyons *et al*, 1999), which instead requires pathogenic responses by populations of Th1 and Th17 cells responding to CNS antigens, infiltrating the CNS, and generating damage in this target organ (Jager *et al*, 2009; Loos *et al*, 2020).

When CNS‐infiltrating Th cells were restimulated with the immunizing peptide, these CNS Foxp3^−^ NRP1^+^ Th cells elaborated IFN‐γ and IL‐17, the hallmark cytokines produced by Th1 and Th17 cells (Fig EV4J). These data indicate that peptide‐specific NRP1^+^ Th1 and Th17 are induced in EAE and support the concept that self‐reactive NRP1^+^ Th cells may be pathogenic irrelevant to PD‐1 expression.

Overall, these data indicate that NRP1 expression by non‐Treg Th cells in autoimmunity may extend further than PD‐1^+^ Tfh/Tph cells in systemic autoimmune disease. Furthermore, such data provide indications that these cells are induced and respond to self‐peptides. However, an outstanding question remains—are these NRP1^+^ Th cells that develop in the context of systemic autoimmune disease involved in pathogenesis?

### NRP1/PD‐1‐expressing Th cells are pathogenic

**Figure 6:**
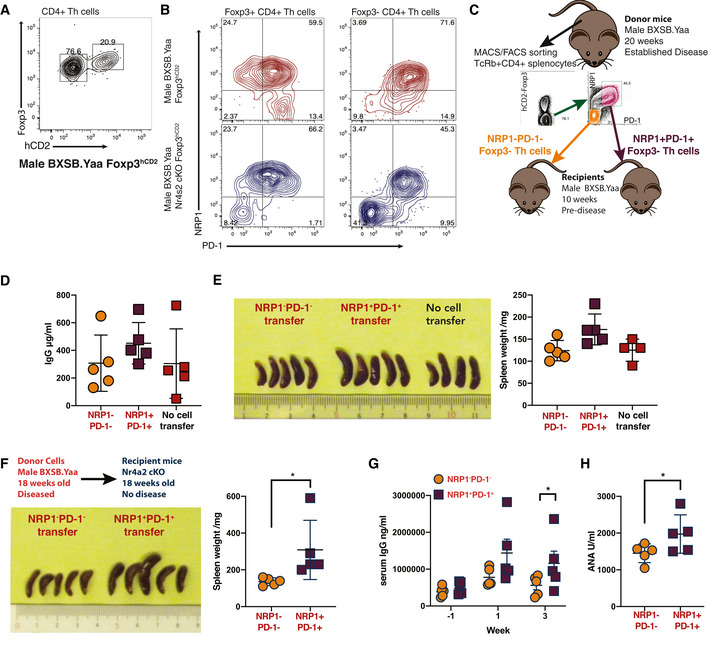
NRP1/PD‐1‐expressing Th cells are pathogenic A, BSpleens from male Foxp3^hCD2^ x BXSB.Yaa mice aged 20 weeks were stained for human CD2 as a Foxp3 surface reporter and intracellularly for Foxp3 among TcRβ^+^CD4^+^ T cells (A) and NRP1/PD‐1 among hCD2^+^ (Foxp3^+^) and hCD2^−^(Foxp3^−^) TcRβ^+^CD4^+^ T cells (B). Data are representative of three independent experiments.C–EAs indicated in (C) splenic NRP1^+^PD‐1^+^ and NRP1^−^PD‐1^−^ Foxp3^−^CD4^+^ conventional T cells sorted from 20‐week‐old male Foxp3^hCD2^ BXSB.Yaa mice and 250,000 cells were transferred into 10‐week‐old male BXSB.Yaa recipient mice. Four weeks after transfer, serum IgG levels were compared with littermate mice receiving no transfer (D), and spleen sizes were measured (E); *n* = 4 mice per group; error bars, mean with SD; representative of three independent experiments.F–HA total of 250,000 NRP1^+^PD‐1^+^ or NRP1^−^PD‐1^−^ Foxp3^−^CD4^+^ T cells were transferred into 18‐week‐old male BXSB.Yaa *Nr4a2* cKO recipient mice. Four weeks after transfer, spleen sizes were measured (F). Serum IgG (G) and ANA (H) concentrations were assessed 1 week before transfer and 1 and 3 weeks after transfer. *n* = 4 mice per group; error bars, mean with SD; representative of five experiments, (F, H) *P < 0.05 unpaired two‐tailed Student's *t*‐test, (G) *P < 0.05 group factor two‐way ANOVA analysis with Bonferroni's multiple‐comparison test.

As Foxp3^−^ Th cells expressing both NRP1 and PD‐1 were associated with autoimmune disease, we next tested whether or not NRP1/PD‐1‐expressing conventional Th cells were directly pathogenic. To discriminate live non‐Treg cell subsets from Treg, we backcrossed a reporter mouse that expresses surface human CD2 under the control of Foxp3 to a BXSB.Yaa background; identifying Foxp3^+^ Tregs more clearly than intracellular Foxp3 staining (Fig 6A). Using this reporter, we flow cytometrically sorted NRP1/PD‐1 and PD‐1^−^ conventional (Foxp3^−^) Th cells and tested them for pathogenicity by transfer into young male BXSB.Yaa mice prior to usual disease onset (Fig 6B and C). Transfer of NRP1^+^ Th cells generated a rapid increase in serum antibodies (Fig 6D) and increased splenomegaly (Fig 6E), whereas disease markers for recipients of PD‐1^−^ conventional Th cells remained equivalent to littermate mice without cell transfer.

**Figure EV5:**
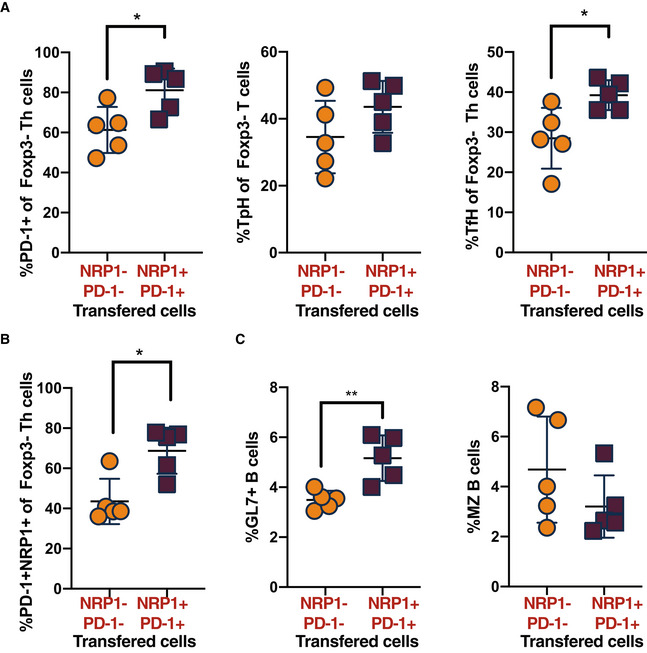
NRP1/PD‐1‐expressing Th cells are pathogenic A–CGroups of male BXSB.Yaa *Nr4a2* cKO mice aged 18 weeks received either NRP1^+^PD‐1^+^ CD4^+^ Foxp3^−^ T cells or NRP1^−^PD‐1^−^ CD4^+^ Foxp3^−^ T cells from 20‐week‐old donor Male Foxp3^hCD2^ × BXSB.Yaa mice. Four weeks after transfer, spleen cells were analyzed by flow cytometry for PD‐1^+^ T cells among Foxp3^−^CD4^+^ Th cells (A), *P < 0.05 two‐tailed unpaired Student's *t*‐test. NRP1^+^PD‐1^+^ cells among Foxp3^−^CD4^+^ Th cells (B), *P < 0.05 two‐tailed Mann–Whitney U test, and GL7^+^ GC B cells and CD21^+^CD23^−^ marginal zone B cells (C), **P < 0.01 two‐tailed unpaired Student's *t*‐test. *n* = 5 mice per group; bars show mean with SD; data are representative of two independent experiments.

Next, we then assessed if these cells were sufficient to generate autoimmunity where disease onset is blocked by a lack of NR4A2 in T cells. We transferred NRP1^+^ conventional Th cells or PD‐1^−^ conventional Th cells into 18‐week‐old male BXSB.Yaa *Nr4a2* cKO mice and measured disease signs induced by transfer of potentially pathogenic cells. Transfer of NRP1^+^PD‐1^+^ Foxp3^−^ Th cell elicited disease progression in protected male BXSB.Yaa *Nr4a2* cKO mice, with splenomegaly (Fig 6F and G) and increases in serum antibodies, including ANA (Fig 6H). These disease signs were accompanied by an expansion of NRP1^+^PD‐1^+^ Th cells in the conventional Th cell pool (Fig EV5A and B). Furthermore, this transfer of T cells also led to increased B cell activation and an increase in GC B cells with a reciprocal decrease in MZ B cells (Fig EV5C).

**Figure 7:**
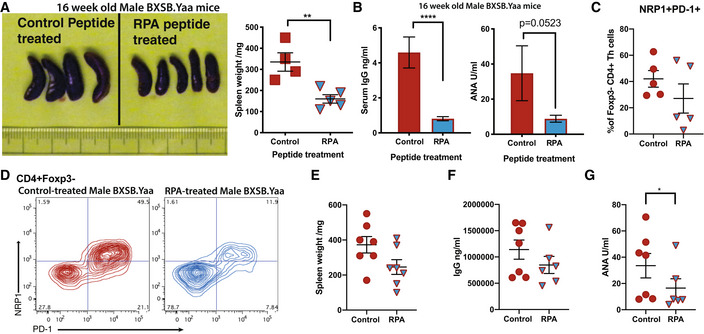
Therapeutic intervention targeting NRP1 ameliorates systemic autoimmunity A–DMale BXSB.Yaa mice were treated i.p. with 200 μM of control peptide (Control) or RPA peptide (RPA) in PBS at 10, 12, and 14 weeks of age. At 16 weeks, spleens were excised, photographed, and weighed (A), **P < 0.01 unpaired two‐tailed Student's *t*‐test and total IgG and ANA concentrations were measured in serum (B), ***P < 0.001, two‐tailed Mann–Whitney U test. Foxp3^−^ splenic Th cells were assessed by flow cytometry for NRP1/PD‐1 sub‐populations, summary data (C) and representative staining (D); *n* = 4 mice per group; error bars, mean with SEM; representative of seven similar independent experiments.E–GFor treatment of established disease, male BXSB.Yaa were injected i.p. with 500 μM control peptide (Control) or RPA peptide (RPA) in PBS at 14, 16, and 18 weeks of age, and mice were sacrificed at 19 weeks old. Spleens were weighed (E) and serum assessed for total IgG (F) and ANA concentration (G), *P < 0.05 two‐tailed Mann–Whitney U test. *n* = 4 mice per group; error bars, mean with SEM; representative of four independent experiments.

These data show a surprising expansion of non‐regulatory T cells co‐expressing NRP1^+^PD‐1^+^ associated with systemic autoimmune disease. These NRP1^+^PD‐1^+^ Th cells from diseased donor mice generated disease in both young male BXSB.Yaa and male BXSB.Yaa *Nr4a2* cKO recipient mice upon transfer. Taken together, we conclude that these NRP1^+^PD‐1^+^ conventional Th cells comprise pathogenic subsets with an implicit self‐reactivity that drives systemic autoimmune disease.

### Therapeutic intervention targeting NRP1 ameliorates systemic autoimmunity

As we have identified a pathogenic population of Th cells that can induce SLE‐like disease, we aimed to explore if targeting these cells could provide a new avenue for the treatment of systemic autoimmunity. Targeting PD‐1 has been used in cancer immunotherapy by enhancing anti‐tumor immune responses (Zou *et al*, 2016), and ligation of PD‐1 with PD‐L1Ig inhibits T cell activation (Freeman *et al*, 2000). We also observed that PD‐L1Ig treatment inhibited *in vitro* proliferation of PD‐1^+^ Th cells in a dose‐dependent manner, but this was only effective on cells from young mice, while similar treatment of Th cells from male BXSB.Yaa mice with established disease enhanced PD‐1^+^ Th cell division (Appendix Fig S3A). As these data indicated that the inhibitory functions of PD‐1 were impaired in mice with established disease, we set out to treat the disease in male BXSB.Yaa mice by targeting pathogenic Th cells via their expression of NRP1.

NRP1 is a functional receptor that offers a route to enter cells; for example, interactions between SARS‐CoV2 spike proteins with NRP1 have been reported to directly allow the SARS‐CoV2 virus to penetrate the cell membrane leading to infection (Cantuti‐Castelvetri *et al*, 2020; Daly *et al*, 2020). This route has been exploited artificially to deliver a drug payload to NRP1‐expressing cancer cells by attachment of an iRGD peptide to NRP1 and so initiate tumor cell killing (Teesalu *et al*, 2009; Kim *et al*, 2016). These works inspired us to design a NRP1‐targeting RPA peptide combined with proapoptotic peptide moiety (Sugahara *et al*, 2010). When this peptide was administered before disease onset, male BXSB.Yaa mice that received RPA treatment (RPA) were protected from splenomegaly (Fig 7A) and lymphadenopathy (Appendix Fig S3B), with a reduced serum secretion of IgG and ANA compared with male BXSB. Yaa mice receiving a control peptide (Control; Fig 7B). RPA treatment reduced NRP1^+^PD^−^1^+^ Th cell expansion (Fig 7C and D) and Tph and Tfh cells were both reduced by RPA peptide treatment (Appendix Fig S3C), while Foxp3^+^ Treg cells were unaffected by treatment targeting NRP1, including those that express NRP1 (Appendix Fig S3D), implying that different NRP1^+^‐expressing subsets have differential susceptibility to the intervention. To test this possibility, we sorted populations of NRP1^+^ Th from spleens of diseased male BXSB.Yaa mice, using our Foxp3 reporter to divide them into Foxp3^+^ Treg cells and Foxp3^−^ conventional Th cells and measured apoptosis induced by *in vitro* treatment with RPA peptide. We found that RPA peptide treatment of NRP1^+^ Th cells led to reduced cells, but NRP1^−^ cells were unaffected (Appendix Fig S3E). Cell death was greater in Foxp3^−^NRP1^+^ Th cells at mid‐to‐high RPA concentration over Foxp3^+^ NRP1 Th cells. Taken together with our *in vivo* data, these findings suggest that Treg cells may be more resistant to apoptosis induction via the NRP1‐targeting suicide peptide RPA.

In addition to prophylactic treatment, treatment after the disease established was still effective at reducing the key indicators of disease (Fig 7E–G), although the reduction in pathogenic T cell subsets was modest (Appendix Fig S3F).

To further test the efficacy of RPA peptide in treating systemic autoimmunity, we also examined the efficacy of NRP1‐targeted treatment in the MRL.LPR mouse model of systemic autoimmunity. Mutation in CD95 gene *Fas* ^*lpr*^ generates spontaneous systemic autoimmune responses leading to massive lymphadenopathy, loss of body weight, glomerulonephritis, and the induction of anti‐dsDNA antibodies (Andrews *et al*, 1978; Cohen & Eisenberg, 1991). MRL.LPR mice treated with RPA peptide showed increased body weight and reduced splenomegaly versus control‐treated MRL.LPR mice (Appendix Fig S3G and H). Lower levels of serum antibodies including anti‐dsDNA autoantibodies (Appendix Fig S3I) also resulted from RPA treatment and splenic NRP1^+^PD‐1^+^ conventional Th cells were reduced (Appendix Fig S3J) along with the reduction in disease indicators, suggesting a link between this subset and pathogenic mechanisms also in the MRL.LPR mice.

### NRP1‐expressing Th cells are upregulated in human SLE patients

Our data from mouse models highlight a pivotal role for NRP1^+^PD‐1^+^ non‐Treg Th cells in systemic autoimmune disease. As targeting NRP1 may provide a new direction for the treatment of systemic autoimmune disease, we tested the relevance of these cells in human SLE disease.

**Figure 8:**
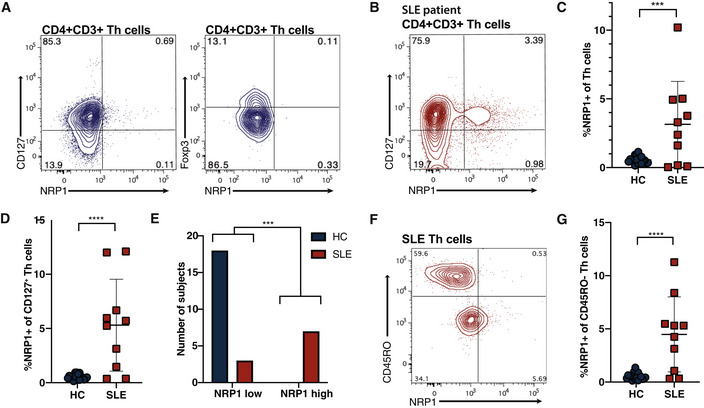
NRP1‐expressing Th cells are upregulated in human SLE patients APBMC were isolated from healthy volunteers and stained for flow cytometry to identify NRP1 expression in CD3^+^CD4^+^ Th cells against CD127 (left) and intracellular Foxp3 (right).B–DNRP1‐expressing cells among CD4^+^CD3^+^ Th cells were measured by flow cytometry in PBMC from 18 healthy volunteers (healthy controls, HC) and 10 SLE patients. Representative staining is shown for CD4^+^CD3^+^ Th cells (B), and summary data for CD3^+^CD4^+^ Th cells (C) and CD3^+^CD4^+^CD127^hi^ conventional cells (D). Plots show individual subjects, HC, *n* = 18; SLE, *n* = 10; with mean ± SD indicated by error bars; ***P < 0.001, ****P < 0.0001 unpaired Student's *t*‐test.EIndividuals were divided into NRP1‐high subjects (NRP1^+^ cells > 1.3% in Th cells) and NRP1‐low subjects (NRP1^+^ cells ≤ 1.35% in Th cells). Numbers of HC and SLE falling into each group are shown. ***P < 0.001 Chi‐squared test with Yates' correction.F, GNRP1‐expressing cells among CD3^+^CD4^+^CD45RO^−^ cells were measured, representative staining (F), and summary data (G). The plot shows individual subjects, HC, *n* = 18; SLE, *n* = 10; with mean ± SD indicated by error bars; ****P < 0.0001 unpaired Student's *t*‐test.

Unlike in mice, human regulatory T cells marked by CD127 downregulation or Foxp3 expression do not express high levels of NRP1 (Fig 8A). This finding is in accordance with a previous study (Milpied *et al*, 2009).

NRP1 levels in circulating Th cells from a cohort of 10 patients with active SLE disease (Appendix Table S2) were assessed and compared with a population of 18 healthy controls (HC). We observed a significant increase in NRP1^+^ Th cells in SLE (Fig 8B and C) and this difference was maintained when only conventional non‐Treg cells were considered (Fig 8D). To account for the uneven gender distribution in SLE, we compared NRP1^+^ Th cell data between female cases and female controls and found the significant difference remained extant (Appendix Fig S4A). Furthermore, the difference was not related to age or disease duration (Appendix Fig S4B and C) and patients in the SLE cohort were selected to have some level of active disease, regardless of treatment and there was no link between NRP1^+^ Th cell level and the level of disease activity (Appendix Fig S4D). While NRP1^+^ Th cell level was somewhat higher in the untreated SLE cohort as compared with the subjects under treatment at the time of sampling, this difference was not significant (Appendix Fig S4E).

As the level of NRP1^+^ Th cells in the HC cohort was normally distributed (D'Agostino & Pearson normality test alpha = 0.05), we applied a cut‐off value of 1.346% (calculated from HC population mean plus 3 standard deviations covering 99.7% of all predicted values in this population) to discern individuals that fall outside the HC population. At this cut‐off level, 7 of 10 SLE patients were defined as NRP1 high with all HC as NRP1 low (Fig 8E); giving this cut‐off a positive predictor value (PPV) of 0.8421 and a false discovery rate (FDR) of 0.

We further investigated the phenotype of NRP1^+^ Th cells in SLE, and observed that the majority were CD45RO‐ (Fig 8F). Indeed, the increase in NRP1^+^ Th cells among SLE patients was even more apparent when only CD45RO‐ Th cells were considered (Fig 8G).

The finding that NRP1^+^ Th cells were increased in SLE patients confirm the utility of our mouse model experiments. This preliminary human study not only indicates that similar pathogenic Th cell mechanisms are likely in play but also that targeting these cells via NRP1 could form a promising treatment, particularly as the lack of NRP1 on human Tregs limits potential side effects.

## Discussion

In this study, we demonstrated that T‐cell‐specific deletion of the *Nr4a2* gene prevents the induction of autoantibodies and SLE‐like systemic disease in male BXSB.Yaa mice. In contrast, antibody production against exogenous antigens was unaltered in the absence of *Nr4a2*, suggesting that NR4A2 expression in Th cells may be an intrinsic requirement for immune responses against self. Furthermore, the NR4A2‐dependent Th cell subsets expanded in disease and expressed a surface phenotype reminiscent of self‐reactive Treg cells which were pathogenic on transfer, and targeting these cells abrogated disease.

Th cells have a critical role in SLE, driving the development of pathogenic B cells producing autoantibodies and subsequent pathology (Craft, 2012; Tellier & Nutt, 2013; Crotty, 2014; Ueno, 2016) and treatments targeting T cells can reduce SLE‐like disease (Chu *et al*, 1996; Yin *et al*, 2015). However, immune targeting approaches without the ability to discriminate self‐ from non‐self‐responses carry a potential risk of side effects due to global immune deficits. Therefore, our new findings that *Nr4a2* deficiency compromises autoreactive T cell responses in systemic autoimmunity, while responses against foreign antigens remain intact, yield a tantalizing prospect for the specific identification of self‐reactive T cells.

NR4A2 and other NR4A family members have been previously linked to circulating autoreactive T cells in type 1 diabetes (Durinovic‐Bello *et al*, 2014). Furthermore, an intrinsic association between NR4A family members and the quality of T cell activation response has been highlighted: *Nr4a1* reporter mice are widely used to estimate antigenic stimulation in thymocytes (Moran *et al*, 2011; Stadinski *et al*, 2016), and TcR avidity in Treg cells is controlled by the presence/absence of *Nr4a2* (Sekiya *et al*, 2013). Therefore, NR4A family members may be crucially required for high‐affinity T cell responses to self‐antigens, irrelevant of their effector or regulatory functional properties. It is this apparent NR4A‐dependent ability to mount high avidity responses to stimulation by low self‐antigen levels or with lower‐affinity self‐reactive TcRs that may be shared between pathogenic autoreactive subsets and Treg cells (Hirota *et al*, 2007; Martin *et al*, 2013).

While Treg cells can have high affinity for self‐antigen, earlier studies suggested that negative selection in the thymus cells permits the release of conventional T cells only with TcRs that have low affinity for self‐antigen (Lee *et al*, 2012). As Treg cells can downregulate Foxp3 and become effector cells under certain conditions (Komatsu *et al*, 2014), it is possible that NR4A2‐dependent pathogenic Th cells with high‐affinity TcR for self‐antigens may be converted from common Treg precursor in the peripheral pool or switch directly from Treg cells during autoimmune disease development.

We show that NRP1 expression refines the identification of NR4A2‐dependent pathogenic Th cells in diseased male BXSB.Yaa mice and, furthermore, self‐reactive NRP1^+^ Th1/Th17 cells are induced in CNS autoimmune disease and trafficking into the target organ. Indeed, it has been previously reported that NRP1 deficiency reduces EAE symptoms, but the mechanism remains unknown (Solomon *et al*, 2011). The unusual NRP1^+^ conventional Th cells sharing a surface phenotype with Treg have not been previously reported in systemic autoimmune disease or target organ autoimmunity. Similar NRP1^+^ Th cells also expand in autoimmune‐prone MRL.LPR mice and *Aire* ^−/−^ mice, but not in older C57BL/6 mice. Slight increases in NRP1^+^ conventional Th cells are also observed in MRL mice by 20 weeks that may occur at the same time as early autoantibody increase seen even without the LPR mutation, although full autoimmunity does not become established in this strain until 1–2 years of age (Izui *et al*, 1984). Transfer of conventional NRP1^+^ Th cells enhances autoimmune disease and NRP1‐targeting treatment (RPA peptide) significantly ameliorated autoimmune indicators, thus not only are NRP1‐expressing conventional Th cells pathogenic and sufficient to induce disease but also are required for the full establishment of disease.

A number of studies highlighted NRP1 expression on subsets of Treg cells (Delgoffe *et al*, 2013; Overacre‐Delgoffe *et al*, 2017), whereas reports indicated NRP1 expression only on a small population of non‐Treg conventional cells (Milpied *et al*, 2009; Solomon *et al*, 2011). It was suggested that NRP1 expression was confined to natural Treg cells and thus could be used to distinguish them from induced Treg (Yadav *et al*, 2012), although later studies conflict with this finding (Singh *et al*, 2015). Our data connecting NRP1 with pathogenic Th cells are interesting when considering that both autoreactive T cells and many Treg cells are specific for self‐peptides and NRP1 may constitute part of a phenotype associated with self‐specific T cells. It is possible that NRP1^+^PD‐1^+^ pathogenic Th cells develop from Treg cells switching to an autoreactive phenotype or from *de novo* NRP1 expression of lower avidity self‐reactive peripheral Th cells. Further investigation into the ontogeny of these cells is required to understand this process.

Tfh and Tph cells have been previously implicated in driving B cell activation in systemic autoimmune disease, but these populations are characterized by markers shared with normal Th cells. Our data indicate that Th cells driving SLE‐like disease may be spread between Tph and Tfh cells and instead depends on NR4A2. Thus, T‐cell‐specific deletion of *Nr4a2* in male BXSB.Yaa mice enabled us to capture self‐reactive T cell subsets as a whole, resulting in the identification of NRP1 on conventional Th cells as a potential biomarker for specifying *bona fide* self‐reactive Th cells. However, without knowledge of all the self‐antigens involved in SLE, it is possible that NRP1^+^ pathogenic cells may only represent a subset of self‐reactive Th cells in SLE‐related diseases. A further caveat to our works is that NRP1 expression on human cells appears more controversial. An early study demonstrated a link between NRP1 and Treg cells in humans (Battaglia *et al*, 2008), but this was disputed by work showing that human Treg cells do not express NRP1 (Milpied *et al*, 2009). Therefore, further NRP1 studies in human disease are highly desirable. Furthermore, NRP1 is a complex receptor with a range of possible functions and a number of different binding domains (Pellet‐Many *et al*, 2008). It interacts immunologically relevant molecules and influences immune responses in a number of potential ways: semaphorins/plexin (Gu *et al*, 2002; Lu *et al*, 2021) and VEGF (Hansen *et al*, 2012) are ligands for different NRP1 domains, and NRP1 has been linked to T cell–dendritic cell interactions (Mizui & Kikutani, 2008) and cytokine signaling (Glinka *et al*, 2011). Thus, the mechanism of action for NRP1 in pathogenic Th cells driving autoreactive B cell responses remains to be understood.

The key challenge in controlling autoreactive responses is to disrupt Th cells responding inappropriately to self‐peptide while leaving beneficial T cell responses intact. However, autoreactive T cell targeting therapy has been limited by the ability to identify and target only self‐reactive T cells, while maintaining essential immune responses. Currently, treatments rely on blocking whole types of immune response/cells or through detailed knowledge of autoantigen(s) to induce tolerance. Most animal research into autoimmune disease focuses on models using defined antigens often with peptides/proteins at non‐physiologic levels or utilizing TcR transgenic mice with an unrealistic amount of self‐specific T cells. Thus, these models may not be suitable for a comprehensive understanding of key hallmarks of autoreactive cells in complex autoimmune diseases such as SLE. Our study approaches the question from a different direction, examining molecules that may be shared by Th cells generally that respond to self‐antigens, NR4A2 and NRP1. NRP1^+^ Th cells are associated with self‐reactive responses in autoimmune neuroinflammation and are pathogenic in SLE‐like disease, with increased NRP1^+^ non‐Treg Th cells in human SLE patients, and NRP1 can be targeted therapeutically to reduce disease. These findings hint at a wide application of NRP1 to detect and target self‐reactive Th cells in a range of autoimmune diseases: however, further study is needed to see how universal this marker is in other diseases and if these cells are the main pathogenic component. These findings potentially open a new approach to treat a wide variety of autoimmune diseases.

## Materials and Methods

### Study design

The aim of this study was to explore the role of NR4A2 in T cell activity in systemic autoimmune diseases; as NR4A2 was previously shown to affect IL‐21 in a target organ‐mediated autoimmune disease, this study focused on an IL‐21‐dependent standard mouse model of SLE. Mice lacking *Nr4a2* in T cells were generated on a background prone to a spontaneous SLE‐like disease, BXSB.Yaa. Breeding was established using a heterozygous system to generate age‐matched littermate groups that either lacked *Nr4a2* in T cells or had intact *Nr4a2* as controls. As the disease develops in male BXSB.Yaa mice, using female mice allowed matched controls with no disease. Preliminary experiments conducted during the backcrossing process confirmed key timepoint for disease induction in this well‐described disease model and required experimental group size. For treatment and transfer experiments, control or experiment groups were assigned randomly; where possible researchers were blinded to the treatment group, but blinding was not possible for mouse groups due to institutional requirements for labeling LMO animals. The number of experimental replicates and group sizes is indicated in the individual figure legends.

### Human subjects

The Ethics Committee of the National Center of Neurology and Psychiatry (NCNP) on human experimentation (A2019‐061) and the Kitasato University School of Medicine and Hospital (B19‐223) approved the study according to Declaration of Helsinki guidelines and Department of Health and Human Services Belmont Report. Written informed consent was obtained from all participants. The subjects donated blood samples for flow cytometric measurement and cohorts are listed in Appendix Table S2. SLE patients were recruited at Kitasato University Hospital, and diagnosis was confirmed according to the 1997 American College of Rheumatology revised classification criteria for SLE (Hochberg, 1997).

### Mice

All mice used were maintained in specific pathogen‐free conditions in accordance with institutional guidelines and used at the ages indicated. This study was approved by the Committee for Small Animal Research and Animal Welfare (National Center of Neurology and Psychiatry). All efforts were made to minimize animal suffering in clinical disease experiments where 5–10 mice were used for scoring in each group. BXSB.Yaa were purchased from Japan SLC, Inc. (Shizuoka, Japan) and were backcrossed 10 generations with C57BL/6 CD4‐Cre/*Nr4a2* ^fl/fl^ (*Nr4a2* cKO) mice (Raveney *et al*, 2015). Foxp3^hCD2^ mice of C57BL/6 background (Komatsu *et al*, 2009) were backcrossed 10 generations with BXSB‐Yaa CD4‐Cre/*Nr4a2* ^fl/fl^ (Nr4a2 cKO) mice. *Aire* ^*tm1*/*1Doi*/*J*^ (*Aire* KO) mice on a C57BL/6 background were purchased from Jackson Laboratory (Bar Harbor, ME, USA) and female MRL and MRL.LPR mice were purchased from Japan SLC, Inc. (Shizuoka, Japan).

For serum antibody measurements, blood was withdrawn by retro‐orbital bleed, cells were removed by centrifugation, and serum was harvested and stored at –80°C until assayed.

For anti‐NP antibody induction in *Nr4a2* cKO mice or control mice with intact *Nr4a2* on BXSB.Yaa or C57BL/6 background, mice were injected subcutaneously with 100 μg NP‐KLH (Biosearch Technologies, CA, USA) emulsified in complete Freund's adjuvant (Difco, KS, USA).

For treatment, NRP1‐targeting peptide (Sequence RPARPAR) or control peptide (sequence GAGAGAG) conjugated to a cell death‐inducing C‐GG‐_D_(KLAKLAK)2 domain (synthesized by Toray, Kanagawa, Japan) was administered, either 200 μM or 500 μM was given per mouse in 200 μl PBS.

EAE induction was carried out using our standard protocol (Raveney *et al*, 2015). Briefly, mice were injected subcutaneously with 100 μg MOG_35–55_ peptide (synthesized by Toray Research Center, Chūō‐ku, Tokyo, Japan) and 1 mg heat‐killed mycobacterium tuberculosis H37RA emulsified in complete Freund's adjuvant (Difco, KS, USA). One hundred nanogram Pertussis toxin (List Biological Laboratories, CA, USA) was injected intraperitoneally (i.p.) on days 0 and 2 after immunization. All peptides were synthesized by Toray Research Center (Chūō‐ku, Tokyo, Japan).

### Cell isolation, staining, and purification

Spleens were excised after mice were humanely euthanized, connective tissues were removed, and organs dried with an absorbent towel before weighing and photography. Single‐cell suspensions were then generated by mechanical disruption of tissues through 70 mm cell strainers (BD Biosciences) and red blood cells were lysed with ACK lysing solution as previously described (Raveney *et al*, 2015). CNS‐infiltrating lymphocytes were isolated from spinal cords and brains as previously described (Klemann *et al*, 2009). Briefly, the tissue was cut into small pieces and digested for 40 min at 37°C in RPMI 1640 media (Invitrogen, Tokyo, Japan) supplemented with 1.4 mg/ml collagenase H and 100 μg/ml DNase I (Roche Diagnostics, Tokyo, Japan). The resulting tissue homogenates were forced through a 70 μm cell strainer and leukocytes were enriched using a discontinuous 37%/80% Percoll density gradient centrifugation (GE Healthcare Life Sciences, Tokyo, Japan).

### Assessment of cell function

For stimulation experiments, culture media used were DMEM supplemented with 10% FCS, 2 mM l‐glutamine, 100 U/ml of penicillin–streptomycin, and 50 μM 2‐mercaptoethanol (all Invitrogen). Serum antibody levels were measured using Mouse IgG1, IgG2a, and IgG2b Ready‐set‐Go! ELISA kits (eBioscience, San Diego, CA, USA) according to manufacturer's instructions or standard ELISA using NP‐CGG (with conjugation ratio of either 4 or 31, Biosearch Technologies, CA, USA) and secondary antibodies. Fecal IgA antibodies were measured by ELISA (Affymetrix) according to the manufacturer's instructions. Anti‐nuclear antibodies were measured using ANA ELISA kits (USBiological, MA, USA) according to the manufacturer's instructions. Mouse IL‐17A and IFN‐γ from supernatants were measured using paired antibody kits according to manufacturers' instructions and were supplied by R&D Systems (Minneapolis, USA) and BD Biosciences, respectively.

For intracellular staining, cells were restimulated with 5 ng/ml PMA/ PMA, 500 ng/ml ionomycin (both Sigma‐Aldrich) in the presence of Golgi Stop (BD Biosciences) for 5 h, before surface staining and fixing/intracellular staining using an eBioscience Foxp3 staining kit according to manufacturer's instructions. For IL‐21 measurement, an IL‐21‐receptor IgG fusion protein (mouse IL‐21R Fc Chimera, R&D systems, MN, USA) was used after fixing cells with an eBioscience Foxp3 staining kit. Cells were then stained for surface antigens. Flow cytometry data were acquired using a FACS Canto II or FACS ARIA II (BD cytometry systems) with a FACS DIVA software. Antibodies for flow cytometry were sourced from BioLegend and are listed in Appendix Table S3.

### Real‐time qPCR

For quantification of mRNA transcripts, total RNA was extracted from flow cytometrically sorted spleen cell populations using a RNeasy Mini Kit (Qiagen, Tokyo, Japan) according to the manufacturer's instructions and cDNA was then generated using a first‐strand cDNA Kit (Takara, Shiga, Japan). Quantified real‐time PCR using a Power SYBR Green master mix (Applied Biosystems, Warrington, UK) with a LightCycler96 instrument (Roche Diagnostics) was performed using commercial primers (Quantitech primers, Qiagen), and gene expression values were normalized to the expression of the GAPDH, HPRT1, or b2M housekeeping genes.

### Histology

Kidney sections obtained from 24‐week‐old *Nr4a2*(fl/fl) or CD4‐Cre/*Nr4a2*(fl/fl) mice at necropsy were fixed with formalin, paraffin embedded, and stained with hematoxylin & eosin, periodic acid/Schiff reagent (PAS), or Masson's trichrome. At least 10 glomeruli were examined for each animal to obtain representative pictures.

### Fluorescence microscopy

Spleens were frozen in OCT at −80°C before sectioning in a Microm‐HM‐525 cryostat. Tissue sections were fixed in acetone for 5 min and blocked with 3% BSA/PBS. Sections were stained overnight with conjugated antibodies: FITC‐conjugated anti‐B220 or anti‐GL7, and biotinylated anti‐CD4 (all Biolegend) with a secondary stain of AlexaFluor‐594‐conjugated streptavidin (Thermo Life Technologies, MA, USA). Sections were mounted with Fluoromount (Southern Biotech, AL, USA) and imaged with a fluorescence BZ‐X850 microscope with a BZ‐X software (Keyence, Osaka, Japan).

### Immunoblotting

Various tissues were dissected from 20‐week‐old BXSB‐Yaa mice and tissue proteins were extracted by using Gentle Macs (Miltenyi Biotech K.K., Tokyo, Japan) according to the manufacturer's instructions.

### Unbiased amplification of TCR genes, sequencing, and data analysis

Next‐generation sequencing analysis was performed with an unbiased TCR repertoire analysis technology (Repertoire Genesis Inc., Osaka, Japan). Unbiased adaptor‐ligation PCR was performed according to the previous reports (Yoshida *et al*, 2000). After PCR amplification, index (barcode) sequences were added by amplification with Nextera XT index kit v2 setA (illumina, San Diego, CA). The indexed amplicon products were mixed in an equal molar concentration and quantified by a Qubit 2.0 Fluorometer (Thermo Fisher Scientific, Waltham, MA). Sequencing was carried out with the Illumina Miseq paired‐end platform (2 × 300 bp). All the paired‐end reads were classified by index sequences. Assignment of sequences was performed by determining sequences with the highest identity in a data set of reference sequences from the international ImMunoGeneTics information system^®^ (IMGT) database (http://www.imgt.org). Data processing, assignment, and data aggregation were automatically performed using a repertoire analysis software originally developed by Repertoire Genesis Inc. Nucleotide sequences of CDR3 regions ranged from conserved cysteine at position 104 (Cys104) of IMGT nomenclature to conserved phenylalanine at position 118 (Phe118) and the following glycine (Gly119) was translated to deduce amino acid sequences. A unique sequence read (USR) was defined as a sequence read having no identity in TRV, TRJ, and deduced amino acid sequence of CDR3 with the other sequence reads. The copy number of identical USR was automatically counted by RG software (Repertoire Genesis Inc, Japan) in each sample and then ranked in order of the copy number.

### Statistical analysis

Data were analyzed using Prism software (GraphPad, CA, USA) and presented as individual points with mean values ± SD. Normality testing was carried out using a Shapiro–Wilk test with a 5% alpha considered normal. Data sets were considered to have an equal variance when standard deviations were within a ratio of 2 and then confirmed with an *F* test. For bivariate hypothesis testing, a non‐parametric Mann–Whitney U test for comparing two sets of non‐Gaussian data and a Student's *t*‐test was used for normally distributed data, with Welch's correction when variances between data sets were unequal. For more than two data sets, a one‐way ANOVA was used for data with a Gaussian distribution with Bonferroni's *post hoc* test for multiple comparisons; when data set did not have equal variances, a Brown–Forsythe one‐way ANOVA test with Dunnett's T3 multiple comparisons test was substituted. For analysis of two independent variables, two‐way ANOVA Bonferroni's *post hoc* test for multiple comparisons. *P*‐values and *n* numbers are stated in the figure legends.

## Author contributions

**Ben JE Raveney:** Conceptualization; data curation; formal analysis; investigation; methodology; writing – original draft; writing – review and editing. **Yosif El‐Darawish:** Data curation; formal analysis; investigation. **Wakiro Sato:** Resources; data curation; project administration. **Yoshiyuki Arinuma:** Resources; formal analysis. **Kunihiro Yamaoka:** Resources; formal analysis. **Shohei Hori:** Resources. **Takashi Yamamura:** Conceptualization; supervision; funding acquisition; writing – review and editing. **Shinji Oki:** Formal analysis; supervision; writing – original draft; project administration; writing – review and editing.

In addition to the CRediT author contributions listed above, the contributions in detail are:

BR and YMED performed experiments; BR and SO designed experiments; BR, TY, and SO analyzed data; WS, YA, and KY provided human samples and analyzed data; SH provided C57BL/6 Foxp3^hCD2^ mice; BR, TY, and SO wrote the manuscript; and SO conceived the study and supervised the project.

## Disclosure and competing interests statement

The authors declare that they have no conflict of interest.

**For more information**

- OMIM: https://omim.org/
- GenBank: https://www.ncbi.nlm.nih.gov/gene/
- Lupus Foundation of America: https://www.lupus.org
- National Center for Neurology and Psychiatry: https://www.ncnp.go.jp/en/

## Supporting information



Appendix

Expanded View Figures PDF

PDF+

Source Data for Figure 1

Source Data for Figure 4

## Data Availability

The raw TcR sequencing data from this publication (Fig 4) have been deposited to the DNA DataBank of Japan (DDBJ) database and registered with BioProject as BioProject ID PRJNA856453; BioSample IDs: SAMN29553878 and SAMN29553879; data available from SRA http://www.ncbi.nlm.nih.gov/bioproject/856453. Aligned TcR vβ/jβ chains with CDR3β sequences are available in the extended data (Dataset Fig 4) with read counts for each sample.
