# Supplementary material for: Neuropilin‐1 (NRP1) expression distinguishes self‐reactive helper T cells in systemic autoimmune disease
Source: EMBO Mol Med. 2022 Sep 7;14(10):e15864. doi: 10.15252/emmm.202215864 (PMC9549730; doi:10.15252/emmm.202215864)
Supplement: Supplementary file 1 — Appendix [file EMMM-14-e15864-s005.pdf]

Appendix

Table of contents

Appendix Fig. S1
Appendix Fig. S2
Appendix Fig. S3
Appendix Fig. S4
Appendix Table S1 Most Common TcR Clones
Appendix Table S2. Characteristics of Human subjects
Appendix Table S3 List of antibodies

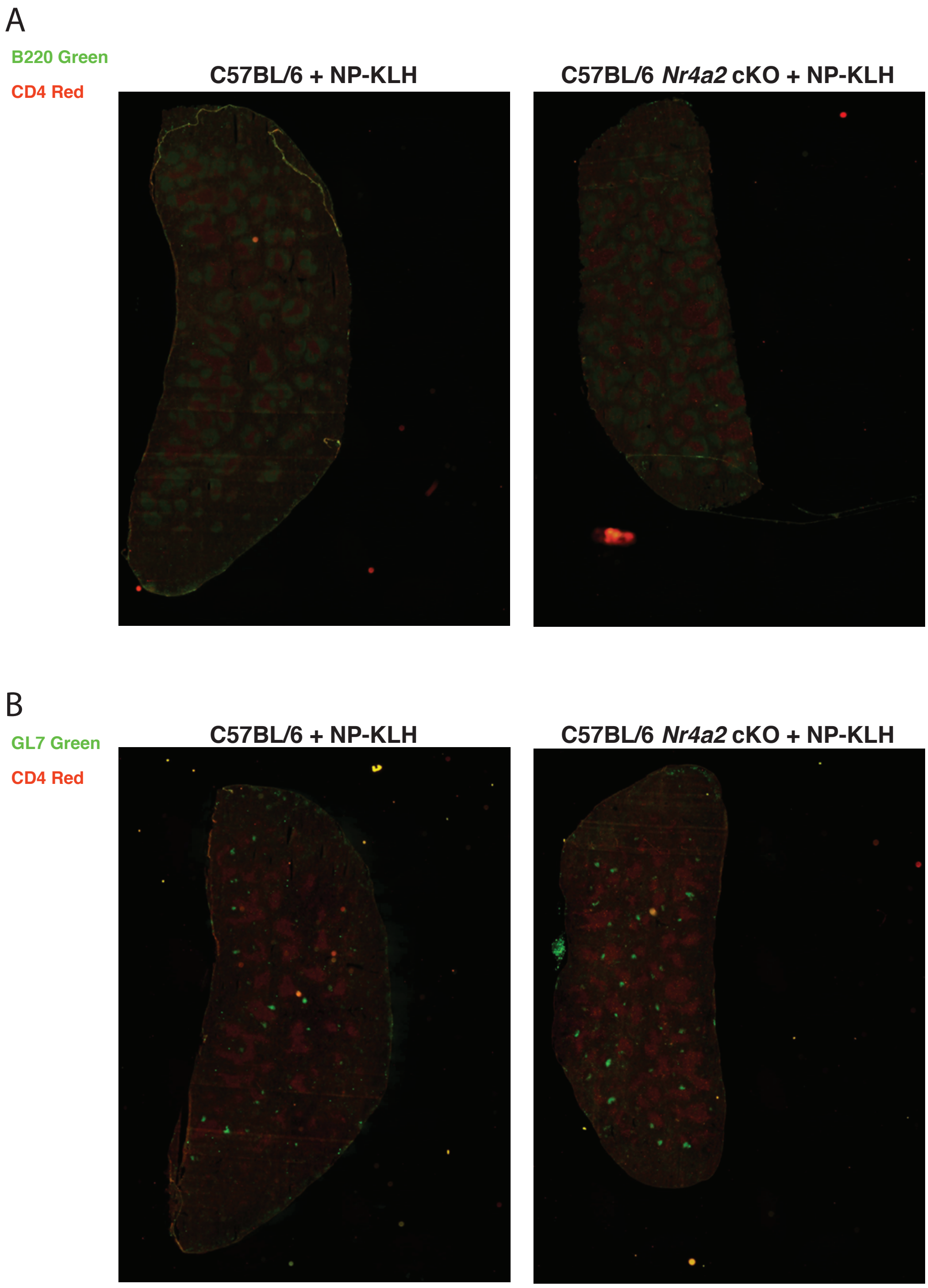

Appendix Fig. S1 – Normal germinal center formation induced by foreign antigen in absence of Nr4a2

Male C57BL/6 *Nr4a2* cKO mice (Right) or wildtype littermate C57BL/6 mice (left) were immunised twice with NP-KLH emulsified in alum. After 2 weeks, 7µm spleen sections were stained with Red:Alexa-Fluor-594-CD4 and Green: FITC-B220 (**A**) to visualise Th cells (red) entering B cell zones (green) or FITC-GL7 (**B**) to visualise Germinal Center formation (green). Representative photomicrographs show whole spleens at 2x magnification.

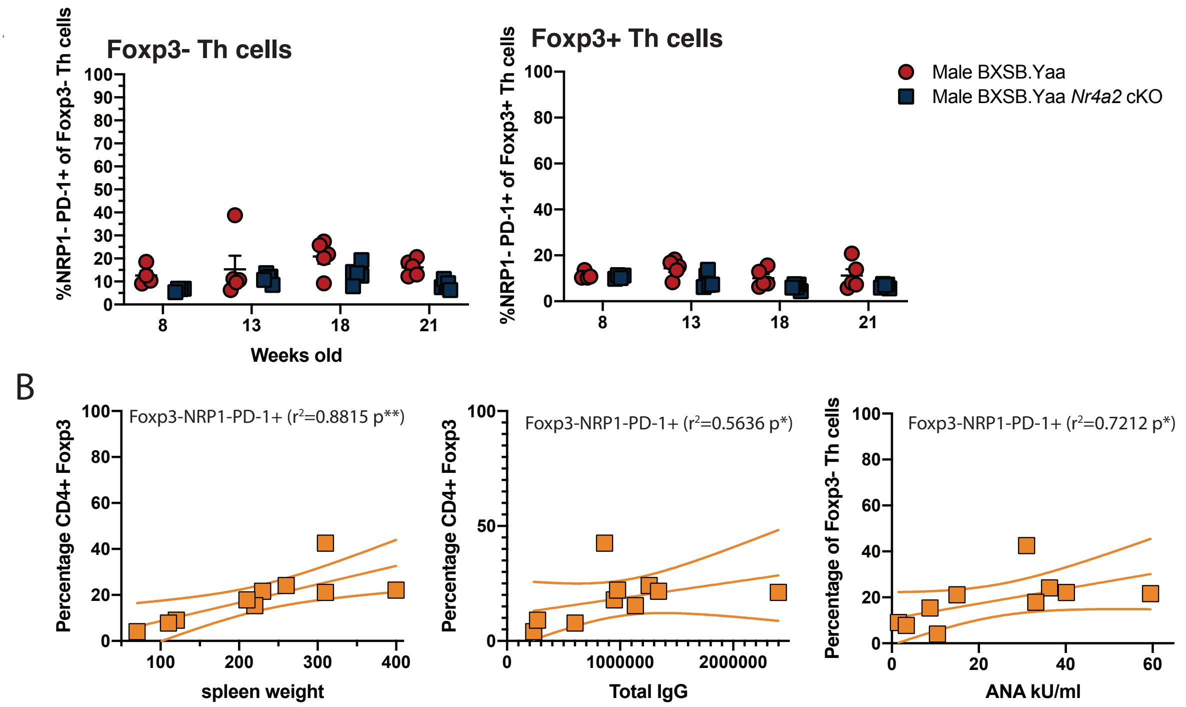

Appendix Fig. S2 NRP1$^-$ PD-1$^+$ Foxp3$^-$ Th cells can upregulate NRP1

Groups of Male BXSB.Yaa and Male BXSB.Yaa *Nr4a2* cKO mice were sacrificed at the indicated ages and splenic TcRβ$^+$CD4$^+$Foxp3- (**A**, left) and TcRβ$^+$CD4$^+$Foxp3$^+$ (**A**, right) were assessed for proportion of NRP1-PD-1$^+$ cells. n=4, Data is representative of at least 2 independent experiments, error bars are SD.

Proportion of NRP1-PD-1$^+$ and NRP1$^+$PD-1$^+$ cells (Y-Axis) from splenic TcRβ$^+$CD4$^+$Foxp3- of 18 week old Male BXSB.Yaa and Male BXSB.Yaa *Nr4a2* cKO was compared with (X-axis) spleen weight (**B**, left), and serum levels of IgG (**B**, center) and ANA (**B**, right). n=8, linear regression line with 95% CVs shown; data is representative of 2 independent experiments.

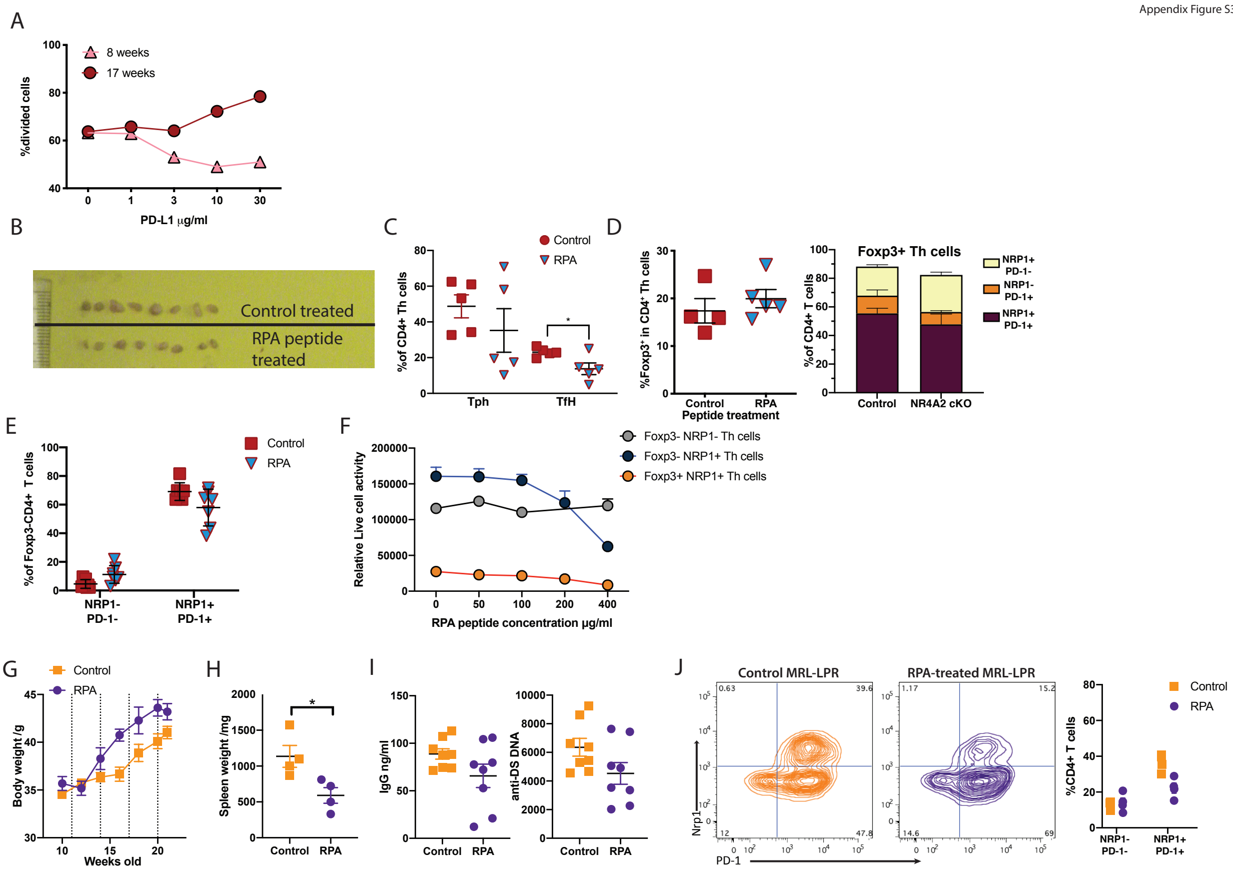

Appendix Fig. S3 Therapeutic intervention targeting NRP1 ameliorates systemic autoimmunity

Spleen cells from Male BxSB.Yaa mice at 8 weeks or 17 weeks of age were sorted by flow cytometry into TcRb$^+$CD4$^+$PD-1$^+$ populations and labelled with Cell trace Violet. Cells were restimulated *in vitro* using plate-bound anti-CD3 (10μg/ml) in the presence of recombinant PD-L1 at the indicated concentrations for 72 hours. Proliferation was calculated by Cell trace violet dilution to indicate the proportion of divided cells for PD-1$^+$ Th cells (**A**). n=4 mice pooled from 1 of 2 independent experiments.

Male BXSB.Yaa mice were treated with an i.p. administration of 200 μM of RPA peptide in PBS (RPA) or PBS alone (Control) at 10, 12, and 14 weeks of age. Mice were sacrificed at 16 weeks' old and inguinal lymph nodes were photographed (**B**). Spleen cells were stained for surface and intracellular markers and analysed by flow cytometry. Proportion of Tph (CXCR5-PD-1$^+$) and Tfh (CXCR5$^+$PD-1$^+$) of TcRβ$^+$CD4$^+$ cells was measured (**C**), and Foxp3$^+$ of TcRβ$^+$CD4$^+$ (**D**, left), and NRP1$^+$PD-1$^-$ and NRP1$^+$PD-1$^+$ of TcRβ$^+$CD4$^+$Foxp3$^+$ was calculated (**D**, right). n=4-5; data representative of 5 independent experiments. Proportion of sub-populations in splenic TcRb$^+$CD4$^+$ conventional Th cells was measured by NRP1/PD-1 staining (**E**). * P<0.05 Two tailed unpaired t-test with Welch's correction. Populations of Foxp3- Th cells were sorted by flow cytometry from spleens of 18 week old Male BXSB.Yaa mice, using the Foxp3^{hCD2} reporter system based on NRP1 and PD-1 expression to yield 3 subsets: TcRb$^+$CD4$^+$Foxp3-NRP1-PD-1-, TcRb$^+$CD4$^+$Foxp3-NRP1-PD-1$^+$, and TcRb$^+$CD4$^+$Foxp3-NRP1$^+$PD-1$^+$. They were then stimulated *in vitro* with pb-anti-CD3 and s-anti-CD28 antibodies with RPA peptide added at the indicated concentrations. On day 6, relative live cell numbers were measured by Cell Titer Glo 2 assay (**F**). These data are representative of 4 independent experiments.

MRL.LPR mice were treated with 200µM of RPA peptide in PBS (RPA) or PBS alone (Control), 11, 14, 17, and 20 weeks of age and mice were weighted fortnightly from week 10. Body weight is shown for each group by age with dotted lines indicating treatments (**G**). Mice were sacrificed at 21 weeks old and spleens weighed (**H**) and serum assessed for total IgG (**I, left**) and anti-DS DNA (**I, right**) concentration. n=10, statistical testing by Two-tailed unpaired t-test with Welch's correction * $P<0.05$. Data is representative of 4 independent experiments.
MRL.LPR mice were treated with 500 µM of RPA peptide in PBS (RPA) or PBS alone (Control), 11, 14, 17, and 20 weeks of age and mice were sacrificed at 21 weeks old. Spleen cells were stained for flow cytometric analysis for NRP1/PD-1 amongst TcRβ^{+}CD4^{+}Foxp3- Th cells; representative staining shown and collective data (**J**). n =5; data is representative of 4 independent experiments.

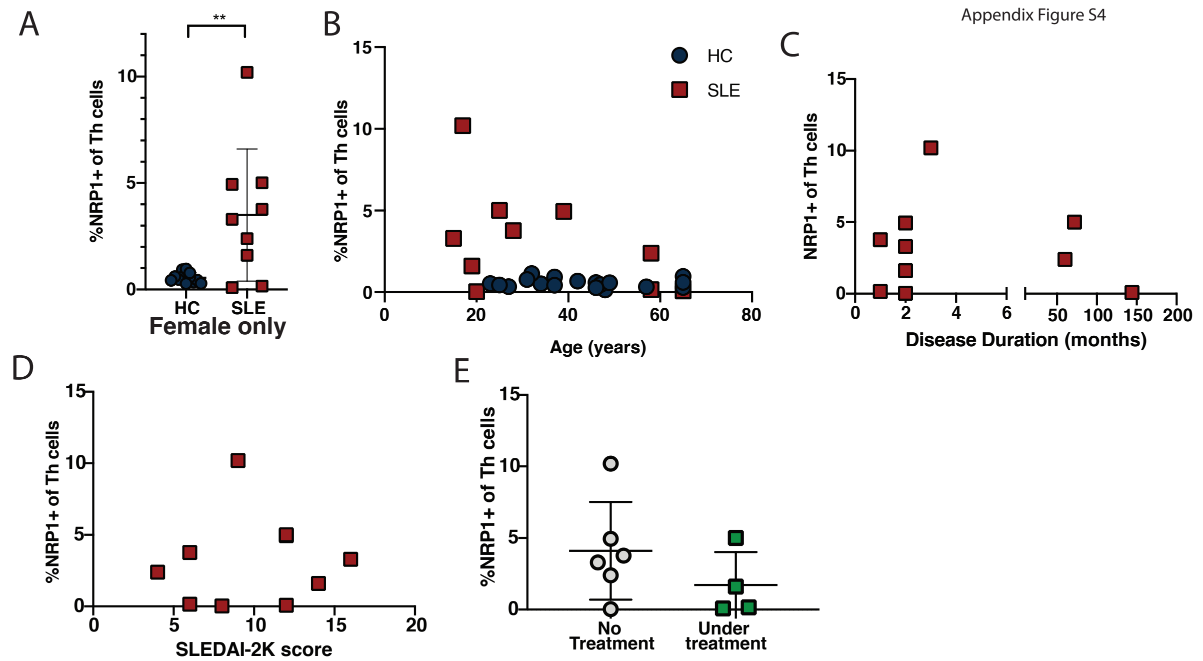

Appendix Fig. S4 NRP1-expressing Th cells are upregulated in human SLE patients

NRP1 expression in CD3$^+$CD4$^+$ Th cells was measured in 12 female healthy control (HC) and 9 female SLE subjects amongst PBMC (**A**) **p<0.001Unpaired Student's T test.
The level of NRP1-expressing cells amongst CD4$^+$CD3$^+$ Th cells was compared with subject age for 18 HC and 10 SLE subjects (**B**) and disease duration for 10 SLE subjects (**C**) No correlation with age for HC group $r^2 = 0.005474$, p =0.7894; weak negative correlation for SLE group $r^2 = 0.2184$, p =0.173 , Two-tailed Pearson correlation coefficient tests

The level of NRP1-expressing cells amongst CD4$^+$CD3$^+$ Th cells was compared with SLE disease activity (SLE Disease Activity Index 2000; SLE-DAI-2K score)for 10 SLE subjects (**D**) Not significant, $r^2 = 0.00695$, p =0.8189, Two-tailed Pearson correlation coefficient test
10 SLE patients were divided into 2 groups based on no current treatment (n=6) or currently under treatment (either MTX or PSL; n=4) and NRP1$^+$ Th cell level was compared between groups (**E**). Not significant, >0.05 Unpaired Student's T test.

Hydrophilic residue 6-7 (Limiting doublet)

Hydrophobic residue 6-7 (Promoting doublet)

Top 30 reads of control CD4+ T cells

TRBV	TRBJ	CDR3β sequence	No.of reads		P6-P7 doublet
			Control	NR4A2cKO	
TRBV29	TRBJ1-1	CASSLGQNTEVFF	1957	13	GQ
TRBV13-3	TRBJ1-6	CASSFRDSYNSPLYF	1748	0	RD
TRBV3	TRBJ2-2	CASSLGLSNTGQLYF	1449	0	GL
TRBV12-2	TRBJ2-5	CASSLELGGQDTQYF	1313	0	EL
TRBV13-3	TRBJ2-2	CASGGGLTGQLYF	1252	0	GL
TRBV13-3	TRBJ2-1	CASSDTGGAYNYAEQFF	1087	0	TG
TRBV12-2	TRBJ2-5	CASSTTGNQDTQYF	1044	0	TG
TRBV13-3	TRBJ2-5	CASSYWGGKDTQYF	1028	0	WG
TRBV13-3	TRBJ1-1	CASSGGSNTEVFF	849	0	GS
TRBV3	TRBJ1-3	CASSSTGDGNTLYF	769	0	TG
TRBV5	TRBJ2-2	CASSQPGTANTGQLYF	585	0	PG
TRBV13-2	TRBJ2-1	CASRGNYAEQFF	566	0	NY
TRBV15	TRBJ1-1	CASSFAGAEVFF	564	0	AG
TRBV5	TRBJ1-1	CASSDGAEVFF	555	0	GA
TRBV13-3	TRBJ1-1	CASSQPGQTEVFF	555	0	PG
TRBV12-2	TRBJ2-5	CASSLDWGDTQYF	531	10	DW
TRBV20	TRBJ2-3	CGARAQSAETLYF	523	0	QS
TRBV3	TRBJ1-2	CASSLVANSDYTF	522	0	VA
TRBV5	TRBJ2-1	CASSQKGGNYAEQFF	507	0	KG
TRBV5	TRBJ2-7	CASSQEDWGGYEQYF	494	0	ED
TRBV16	TRBJ2-7	CASSLETGGYEQYF	492	0	ET
TRBV5	TRBJ1-4	CASSQPNERLFF	490	0	PN
TRBV1	TRBJ1-5	CTCSADNGHNNQAPLF	469	0	DN
TRBV29	TRBJ2-2	CASSRQGASGQLYF	456	0	QG
TRBV5	TRBJ2-5	CASSQDGGADTQYF	449	0	DG
TRBV5	TRBJ1-5	CASSQDYNNQAPLF	438	0	DY
TRBV5	TRBJ1-6	CASSGTTSYNSPLYF	435	0	TT
TRBV5	TRBJ1-1	CASSPHRDAEQFF	432	0	HR
TRBV2	TRBJ2-1	CASSQPGQNTEVFF	432	0	PG
TRBV13-3	TRBJ2-1	CASGGLGGSALYF	431	0	LG

Top 30 reads of NR4A2 cKO CD4+ T cells

TRBV	TRBJ	CDR3β sequence	No.of reads		P6-P7 doublet
			Control	NR4A2cKO	
TRBV15	TRBJ1-1	CTCSGGQNSDYTF	0	2407	GQ
TRBV13-2	TRBJ2-5	CASSLRGDQDTQYF	0	1341	RG
TRBV12-2	TRBJ2-7	CASSGGGKNAEQFF	0	1242	GG
TRBV1	TRBJ2-7	CASGDPGGNERLFF	0	1102	PG
TRBV31	TRBJ1-3	CSSRLGGRDAETLYF	0	960	GG
TRBV15	TRBJ2-5	CASGDEGGNTLYF	0	933	EG
TRBV13-3	TRBJ2-2	CTCSAGPRDRAAEQFF	0	896	GP
TRBV13-2	TRBJ2-5	CASSYRRDPYSGNTLYF	0	777	RR
TRBV15	TRBJ2-2	CASSLETGGYAEQFF	0	766	ET
TRBV13-2	TRBJ2-4	CATRDRGHERLFF	0	764	RG
TRBV1	TRBJ2-7	CTCSGQIQDTQYF	0	759	QI
TRBV13-2	TRBJ2-4	CASSHDRNQDTQYF	0	727	DR
TRBV13-2	TRBJ2-7	CASSQDWNNYAEQFF	0	724	DW
TRBV15	TRBJ1-4	CASSLNSDYTF	0	662	NS
TRBV1	TRBJ1-5	CASSWGGAGDTQYF	0	623	GG
TRBV2	TRBJ2-7	CASSQEGGASGNTLYF	0	584	EG
TRBV31	TRBJ2-4	CASSQEDRGNTEVFF	0	567	ED
TRBV13-2	TRBJ1-1	CASSLAGGSYEQYF	0	565	AG
TRBV30	TRBJ2-3	CASGASDRGSSQNTLYF	0	556	SD
TRBV5	TRBJ2-2	CASSPGGSAETLYF	0	543	GG
TRBV3	TRBJ2-4	CASSLAGGAGEQYF	0	531	AG
TRBV13-3	TRBJ2-7	CTCSADRWGKDEQYF	0	529	DR
TRBV15	TRBJ2-2	CTCSRQGGGGNTLYF	0	527	QG
TRBV15	TRBJ1-1	CASSDAGARGERLFF	0	515	AG
TRBV1	TRBJ2-5	CASGDAMGGRGQNTLYF	0	495	AM
TRBV1	TRBJ2-1	CASGDAGGAYEQYF	0	488	AG
TRBV5	TRBJ2-7	CASGGDKYEQYF	0	482	DK
TRBV13-1	TRBJ1-4	CASGDAGTGGAGYEQYF	0	480	AG
TRBV5	TRBJ1-3	CASSLRDWGAETLYF	0	455	RD
TRBV13-2	TRBJ1-5	CASSWGSSYEQYF	0	454	GS

Appendix Table S1 – Most common Th cells clones by CDR3β TcR sequences read number in SLE-like disease

Memory splenic T cells (TcRb^{+}CD4^{+}CD44^{hi}CD62L^{lo}) were sorted from individual 20 week old BxSB mice either Control or NR4A2 cKO. These cells were subjected to unbiased TCR repertoire analysis using next-generation sequencing. Sequences were categorized based on residues CDR3 position 6&7: hydrophobic P6/7 doublets (promoting self-reactivity) and hydrophilic doublets (limiting self-reactivity). Unique TcR sequences were ordered by most common clones based on read number.
The most numerous 30 CDR3 reads are listed for control T cells (left) and NR4A2 cKO T cells (right) with hydrophobic P6/7 doublets (promoting self-reactivity) coloured red and hydrophilic P6/7 doublets coloured (limiting self-reactivity) blue.

Appendix Table 2 - Characteristics of Human subject Cohorts

	SLE	Healthy controls (HC)
Number	10	18
Male (%)	1(10%)	6 (37%)
Female (%)	9(90%)	12 (67%)
Age±sd<br>(range)	34.40±19.22<br>(15-64)	43.11±13.59 (23-64)
Onset Age±sd<br>(range)	32.10±17.06<br>(15-43)	N/A
Disease duration months ±sd<br>(range)	28.90±48.53<br>(1-144)	N/A
SLEDAI-2K<br>±sd<br>(range)	9.8±3.9<br>(4-16)	N/A
Treament<br>(numbers of subjects)	No treatment - 6<br>MTX -1<br>PSL alone - 1<br>PSL + CyA -1<br>PSL +HCQ - 1	No immunosuppressive drugs - 18

Drug abbreviations: MTX- methotrexate; PSL- prednisone; CyA - Cyclosporin A; HCQ – Hydroxychloroquine

Antibody Name	Specificity	Clone	Supplier
CD11b	Anti-mouse/human	M1/70	Biolegend
CD11c	Anti-mouse	N418	Biolegend
CD127	anti-human	AO19D5	Biolegend
CD16	Anti-mouse	W20015B	Biolegend
CD184/CXCR4	Anti-mouse	L276F12	Biolegend
CD185/CXCR5	Anti-mouse	L138D7	Biolegend
CD2	anti-human	TS1/8	Biolegend
CD21	Anti-mouse	7.00E+09	Biolegend
CD23	Anti-mouse	B3B4	Biolegend
CD25	Anti-mouse	PC61	Biolegend
CD279/PD-1	Anti-mouse	297.1A12	Biolegend
CD279/PD-1	anti-human	EH12.2H7	Biolegend
CD28	Anti-mouse	37.51	Biolegend
CD3	anti-human	OKT3	Biolegend
CD304/NRP1	Anti-mouse	3E12	Biolegend
CD357/GITR	Anti-mouse	DTA-1	Biolegend
CD3e	Anti-mouse	2C11	Biolegend
CD4	Anti-mouse	GK1.5	Biolegend
CD4	anti-human	RPA-T4	Biolegend
CD44	Anti-mouse	IM7	Biolegend
CD45	Anti-mouse	30-F11	Biolegend
CD45R/B220	Anti-mouse	RA3.6B2	Biolegend
CD45RA	anti-human	HI100	Biolegend
CD45RO	anti-human	UCHL1	Biolegend
CD62L	Anti-mouse	W180.21D	Biolegend
CD8	Anti-mouse	53-6.7	Biolegend
CD8	anti-human	SK1	Biolegend
CD95	Anti-mouse	SA367H8	Biolegend
Foxp3	Anti-mouse	MF-14	Biolegend
GL7	Anti-mouse	GL7	Biolegend
ICOS	Anti-mouse/human	C398.4A	Biolegend
Ly6C	Anti-mouse	HK1.4	Biolegend
Ly6G	Anti-mouse	1A1	Biolegend
NK1.1	Anti-mouse	S17016D	Biolegend
NRP1	anti-human	12C2	Biolegend
TcRb	Anti-mouse	H57.397	Biolegend

Appendix Table 3
List of Antibodies used
