# Supplementary material for: Neuropilin‐1 (NRP1) expression distinguishes self‐reactive helper T cells in systemic autoimmune disease
Source: EMBO Mol Med. 2022 Sep 7;14(10):e15864. doi: 10.15252/emmm.202215864 (PMC9549730; doi:10.15252/emmm.202215864)
Supplement: Supplementary file 2 — Expanded View Figures PDF [file EMMM-14-e15864-s004.pdf]

Expanded View Figures

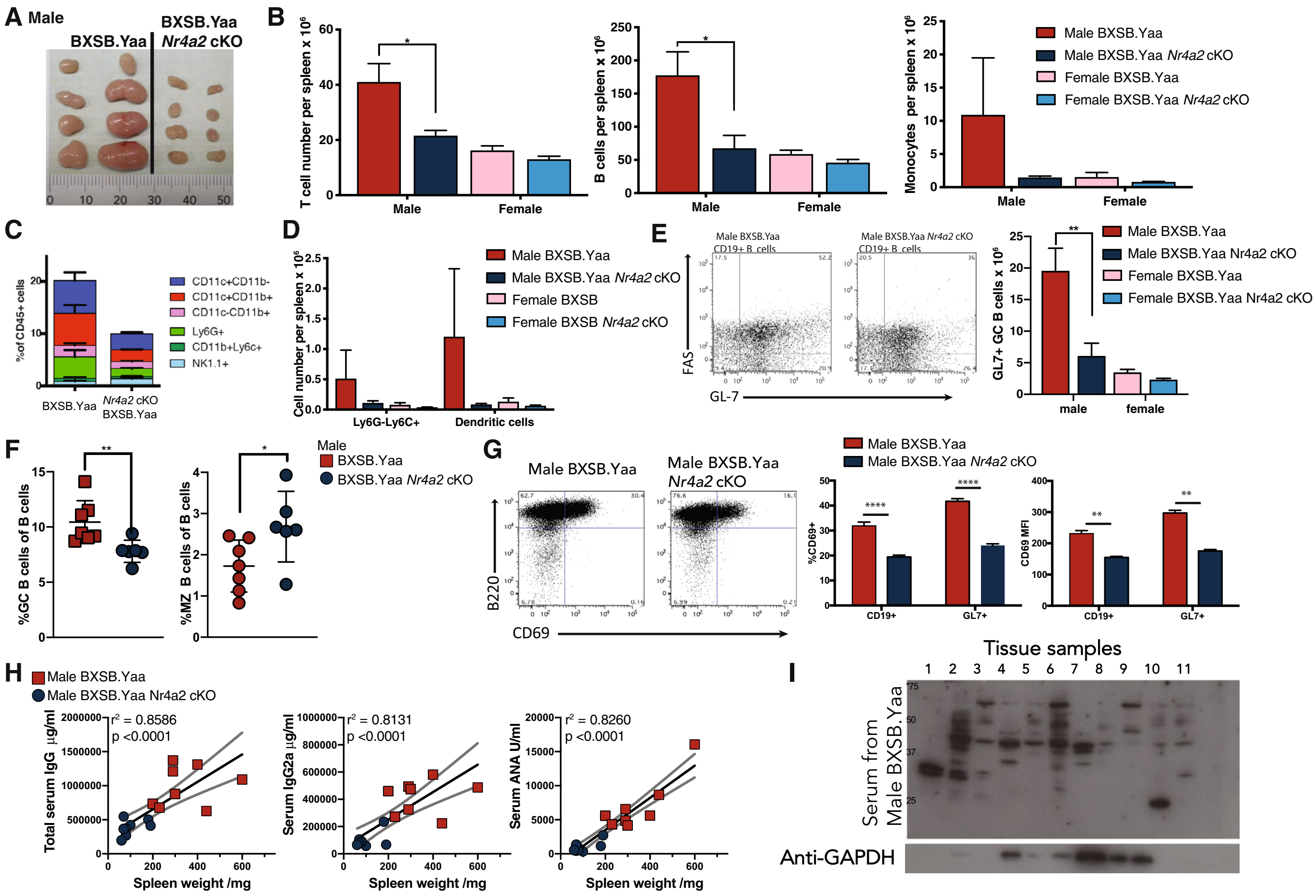

Figure EV1. T-cell-specific deletion of the *Nr4a2* gene abrogates systemic autoimmunity in BXSB mice.

A Inguinal lymph nodes from 16-week-old male BXSB.Yaa or BXSB.Yaa *Nr4a2* cKO mice were photographed.

B–G Spleens from male and female mice were disrupted and cell numbers were counted by flow cytometry: TcRb$^+$ T cells (B, left), CD19$^+$ B cells (B, center), and scatter gated CD45$^+$CD19$^-$TcR$^-$ NK1.1$^-$ monocytes (B, right). Non-T and -B cells from male mice were further fractionated by staining with CD11b, CD11c, Ly6G, Ly6C, as well as NK1.1 (C). CD45$^+$CD11b$^+$Ly6G-Ly6C$^+$ cells (Ly6G-Ly6C$^+$) and CD45$^+$CD11c$^+$ (dendritic cells) were also assessed from male and female mice (D). Germinal center B cell (CD19$^+$B220$^+$ FAS$^+$GL7$^+$, GC B cells) numbers per spleen were also assessed: representative flow cytometry staining is shown (E, left), GC cell numbers for individual mice (E, Right) and GC B cells and CD19$^+$B220$^+$CD21$^-$CD23$^+$ marginal zone (MZ) B cells measured as a proportion of total CD19$^+$ B cells (F). B cell activation as measured by CD69 flow cytometry in total CD45$^+$TcR$^-$CD19$^+$ B cells (CD19$^+$) and CD45$^+$TcR$^-$CD19$^+$GL7$^+$ B cells (GL7$^+$), representative flow cytometry staining is shown (G, left), CD69$^+$ cell populations as a percentage of parent cells (G, center), and CD69 mean fluorescent intensity (MFI) of cell populations (G, right). n = 4–8 mice per group and data represent 3–6 similar experiments. Error bars represent SEM for individual mice; n = 4–7; *P < 0.05, **P < 0.01, ***P < 0.001, ****P < 0.0001 unpaired two-tailed Student's t-test with Welch's correction. Data are representative of at least 10 independent experiments with 4–10 per group.

H Serum concentrations of antibodies (Y-axis) was compared with spleen weight (X-axis) from 16-week-old male BXSB.Yaa and male BXSB.Yaa *Nr4a2* cKO mice; for total IgG (left), IgG2a (center), and ANA (right). n = 8 mice per group; linear Spearman R correlation test; regression analysis (solid line) shown with 95% confidence intervals (dotted lines).

I IgG serum antibody binding to PAGE-resolved proteins isolated from 11 tissues from 16-week-old male BXSB.Yaa mice. Tissues are as follows: 1: salivary glands, 2: liver, 3: lung, 4: heart, 5: stomach, 6: kidney, 7: musele, 8: brain, 9: spinal cord, 10: small intestine, 11: large intestine.

© 2022 The Authors

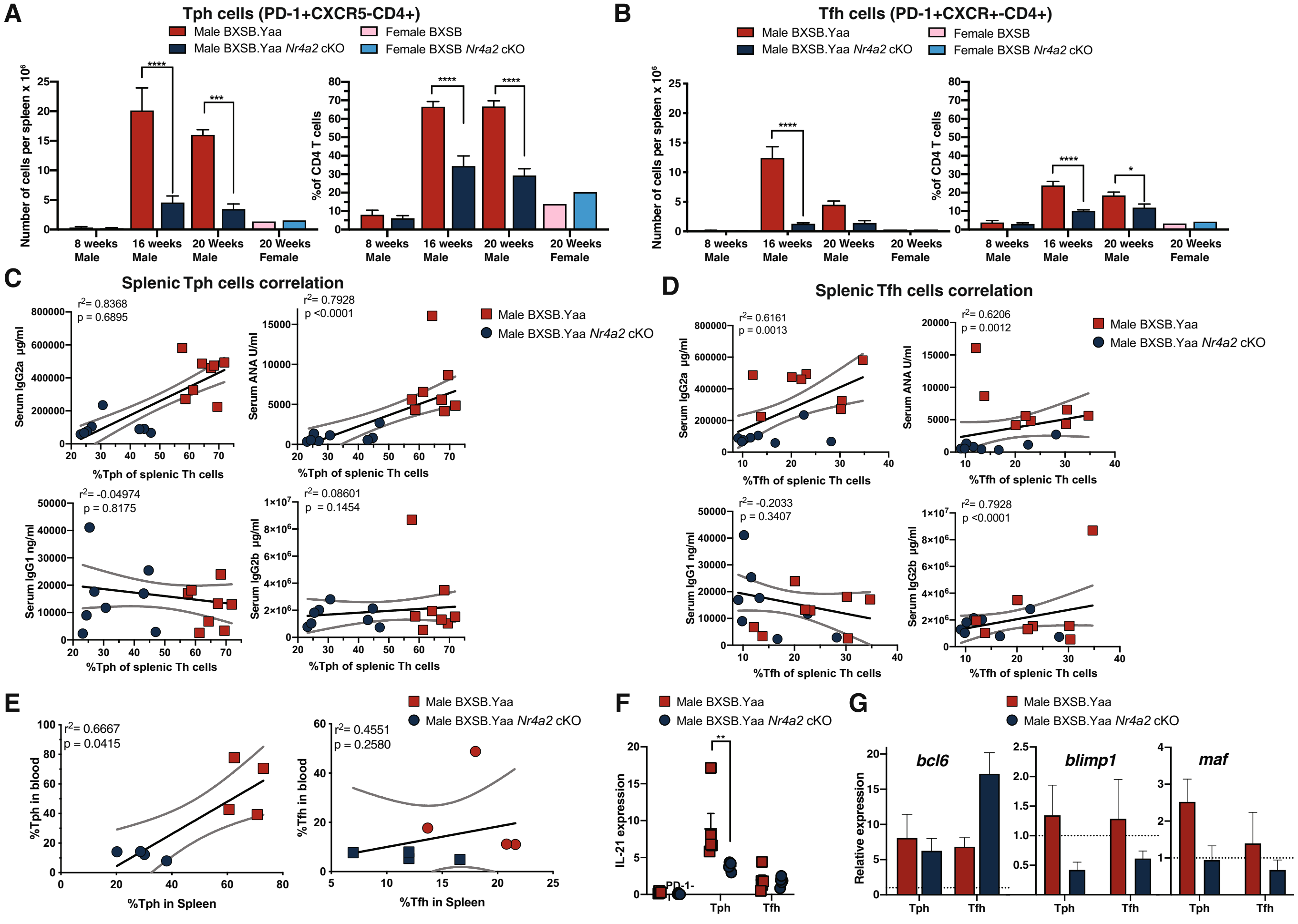

Figure EV2. Rapid expansion of diverse Th cell subsets in BXSB mice is NR4A2 dependent.

A–E Spleen cell suspensions from 8- to 20-week-old male and 16-week-old female BXSB.Yaa and BXSB.Yaa *Nr4a2* cKO mice were evaluated for populations within TcRβ$^+$CD4$^+$ Th cells by cell number per spleen and proportion among Th cells for Tph (PD-1$^+$CXCR5$^-$) cells (A) and Tfh (PD- 1$^+$CXCR5$^+$) cells (B). Data are representative of at least 4–10 independent experiments with 4–10 mice per group; error bars, mean with SEM; **P* < 0.05, ****P* < 0.001, ****P* < 0.001 two-way ANOVA with Bonferroni's multiple-comparison test. For individual mouse, serum IgG2a, ANA, IgG1, and IgG2b level was compared with subset percentage in splenic Th cells for Tph cells (C) and Tfh cells (D). Linear regression lines shown with 95% CI; statistical testing by Spearman *R* test indicated; *n* = 8 per group across 2 independent experiments. For 16-week-old male mice, Th cell subset proportions in spleens (X-axis) were compared with subset proportion in blood (Y-axis) among whole Th cells for Tph cells (E, left) and Tfh cells (E, right). *n* = 4; Spearman *R* correlation test; linear regression analysis lines shown with 95% confidence intervals.

F, G Splenic Th cells from 16-week-old male BXSB.Yaa and BXSB.Yaa *Nr4a2* cKO mice purified by flow cytometric cell sorting into PD-1$^-$, PD-1$^+$CXCR5$^-$ (Tph), and PD-1$^+$CXCR5$^+$ (Tfh) cells and gene transcription for *il21* (F); ***P* < 0.01 two way ANOVA with Bonferroni's multiple-comparison test, and *bcl6*, *blimp1*, and *maf* (G) were assessed by real-time qPCR. Relative transcript levels were normalized to PD-1$^-$ cells (PD-1$^-$ cells were accorded level 1.0, indicated by dotted lines), *n* = 4 mice per group; error bars, mean with SEM; data are representative of at least three similar experiments.

 © 2022 The Authors

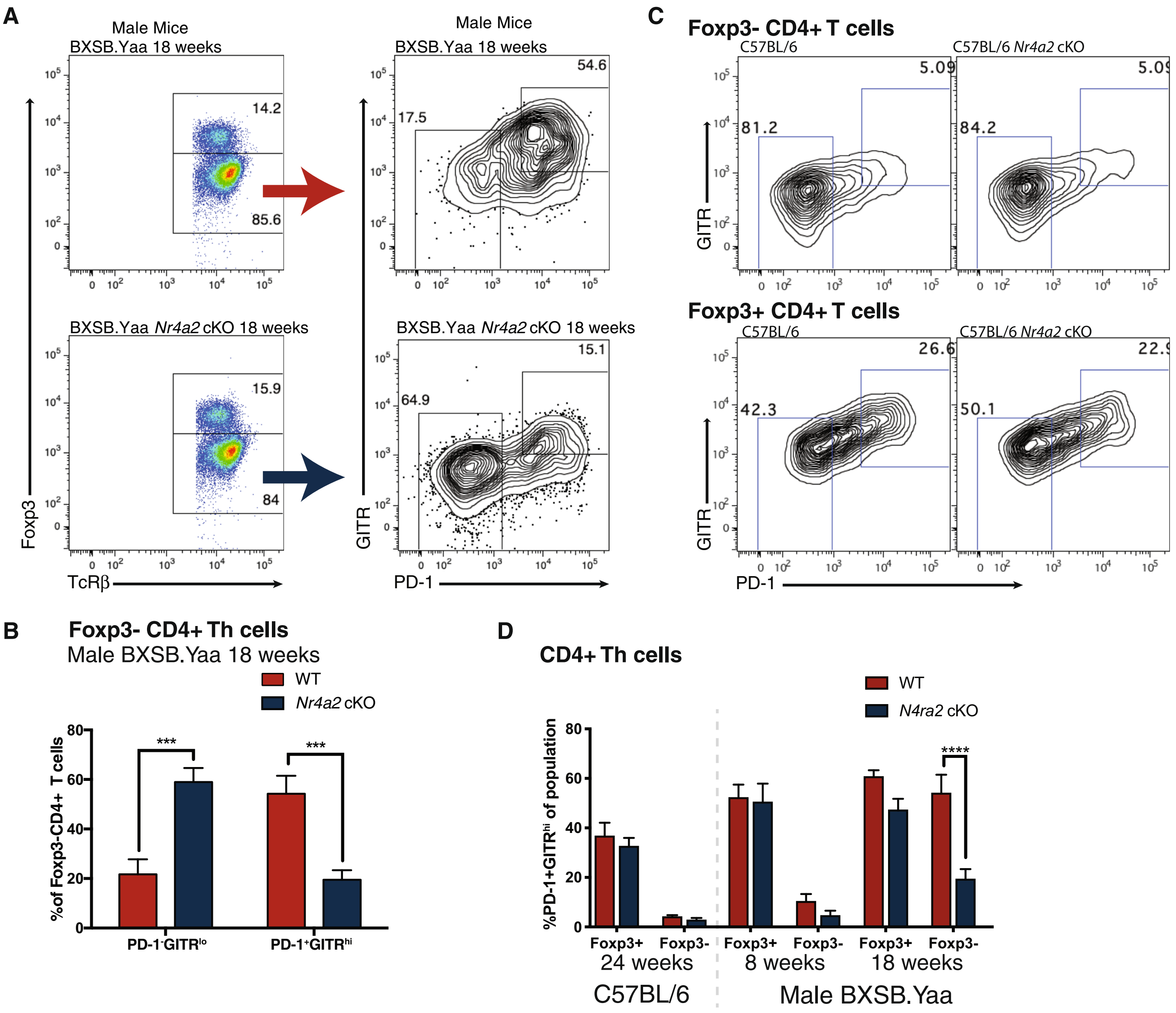

Figure EV3. Foxp3⁻ non-Treg Th cells subset expanded in diseased male BXSB.Yaa mice shares markers with Foxp3⁺ Treg cells.

A, B Single-cell suspensions from spleens of male BXSB.Yaa or male BXSB.Yaa Nr4a2 cKO mice aged 18 weeks were stained with surface antibodies against TcRβ, CD3, PD-1, and GITR before intracellular staining with anti-Foxp3. Foxp3 staining was used to select $Foxp3^-$ conventional Th cells and GITR/PD-1 levels were assessed. Representative staining is shown (A) with the percentage of conventional $PD\text{-}1^-GITR^{lo}$ and $PD\text{-}1^+GITR^{hi}$ Th cells shown for $Foxp3^-$ cells (B).***$P < 0.001$; Two-way ANOVA with Bonferroni's multiple comparisons test; $n = 3$–5 mice per group; error bars are SEM. These data are representative of three independent experiments.

C Single -ell suspensions from spleens of wild-type C57BL/6 or *Nr4a2* cKO C57BL/6 mice aged 24 weeks were stained and examined by flow cytometry and Foxp3 was used to gate T cells into $Foxp3^+$ and $Foxp3^-$. GITR vs. PD-1 expression in $CD4^+TcR\beta^+$ T cells is shown for $Foxp3^-$ and $Foxp3^+$ These data are representative of three independent experiments ($n = 2$–4 mice per group);

D Single-cell suspensions from spleens of wild-type C57BL/6 or *Nr4a2* cKO C57BL/6 aged 24 weeks or male BXSB.Yaa or male BXSB.Yaa Nr4a2 cKO aged either 8 or18 weeks were stained with surface antibodies against, TcRβ, CD3, PD-1, and GITR, before intracellular staining with anti-Foxp3. Foxp3 staining was used to select conventional Th cells and Treg cells. The percentage $PD\text{-}1^+GITR^{hi}$ Th cells is shown for $Foxp3^-$ conventional Th cells and $Foxp3^+$ Treg cells. $n = 4$–6 mice per group error bars, mean with SEM; ****$P < 0.0001$ two-way ANOVA with Bonferroni's multiple-comparison test.

© 2022 The Authors

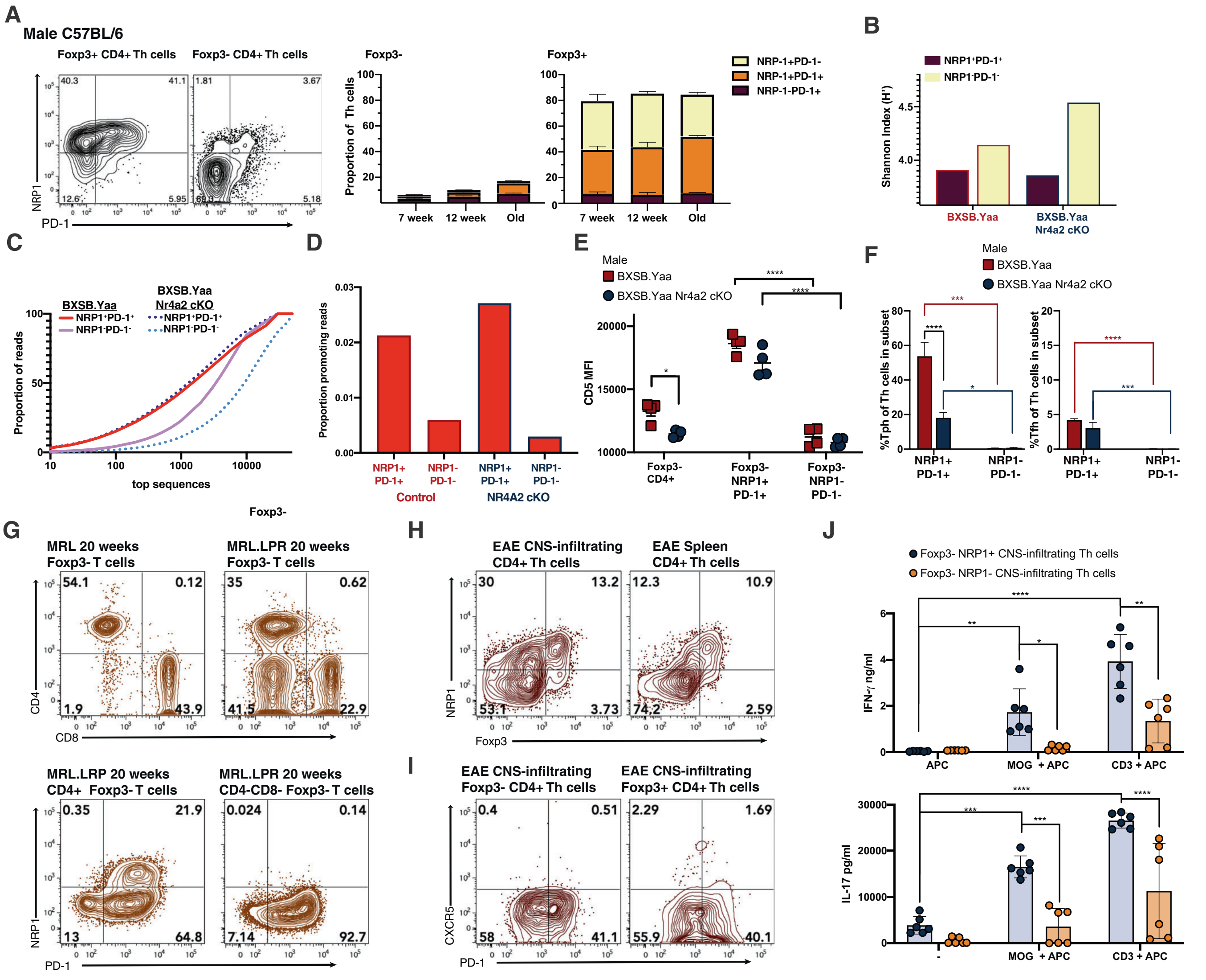

Figure EV4. NRP1 expression identifies a *Nr4a2*-dependent self-reactive Th cell subset expanded in SLE.

A Spleens from wild-type C57BL/6 mice were stained for NRP1 and PD-1 among $TcR\beta^+CD4^+Foxp3^+$ T cells and $TcR\beta^+CD4^+Foxp3^+$ T cells for flow cytometry. Representative staining from 12-week-old mice and proportions of NRP1/PD-1 subsets from mice aged 7, 12, and over 24 weeks old are shown (A), $n = 3$–8 per representative of least two independent litters; error bars, mean with SD.

B–D TcR repertoires were analyzed among $NRP1^+PD1^+$ and $NRP1^-PD\text{-}1^-$ -sorted Th subsets from 16-week-old male BXSB.Yaa and BXSB.Yaa *Nr4a2* cKO mice. Repertoire diversity was indicated by Shannon index (B) and by calculating the cumulative proportion of the total TcR sequence reads against decreasingly common unique sequences to show distribution skewing (C) and the number of reads for clones with self-reactivity promoting TcRs was calculated for the top 1,000 clones (D).

E CD5 expression was measured by flow cytometry among $Foxp3^-$ Th subsets from male BXSB.Yaa and BXSB.Yaa *Nr4a2* cKO mice aged 18 weeks. CD5 MFI is shown for the whole $Foxp3^-CD4^+$ T cells, and $NRP1^+PD1^+$ and $NRP1^-PD\text{-}1^-$ subsets of $Foxp3^-$ Th cells; $n = 5$ mice per group, error bars show SEM, $*P < 0.05$, $****P < 0.0001$ two-way ANOVA with Bonferroni's multiple-comparison test; data are representative of at least three independent experiments.

F $Foxp3^-CD4^+TcR\beta^+$ splenocytes from 18-week-old Male BXSB.Yaa and BXSB.Yaa *Nr4a2* cKO mice divided into $NRP1^+PD\text{-}1^+$ and $NRP1^-PD\text{-}1^-$ subsets were assessed for proportions of Tph (left) and Tfh (right) cells based on CXCR5/PD-1 flow cytometry; $n = 5$ mice per group; data are representative of three independent experiments; error bars show SEM; $****P < 0.0001$, $***P < 0.001$, $*P < 0.05$, two-way ANOVA with Bonferroni's multiple-comparison test.

G Splenocytes from 20-week-old female MRL or MRL.LPR mice were stained for flow cytometry and NRP1 and PD-1 expression was measured for $TcR\beta^+CD4^-CD8^-$ cells. Data are representative of five individual mice per group.

H–J EAE was induced in male C57BL/6 $Foxp3^{hCD2}$ reporter mice. On day 17 peak disease, splenocytes and CNS-infiltrating T cells were isolated and stained for flow cytometry. Representative FACS staining shown for NRP1 versus Foxp3 reporter for $CD45^+CD11b^-TcRb^+$ Th cells (H) and for CXCR5 and PD-1 for $Foxp3^-$ or $Foxp3^+$ $NRP1^+CD45^+CD11b^-TcRb^+$ CNS Th cells (I). Pools of three mice were combined and $NRP1^+$ and $NRP1^-$ $Foxp3^-$ Th cells were purified by FACS sorting. Non-T-cell fractions were irradiated and used as APC. Th cells and APC were co-cultured in the presence or absence of 100 µg/ml MOG or 2 µg/ml anti-CD3. After 96 h, supernatants were collected and cytokine concentrations were measured by ELISA (J). Error bars, SD $****P < 0.0001$, $**P < 0.01$, $*P < 0.05$ two-way ANOVA analysis with Bonferroni's multiple-comparison test. Points show triplicates for two pools of three individual mice. Data are representative of at least two independent experiments with six mice per experiment.

© 2022 The Authors

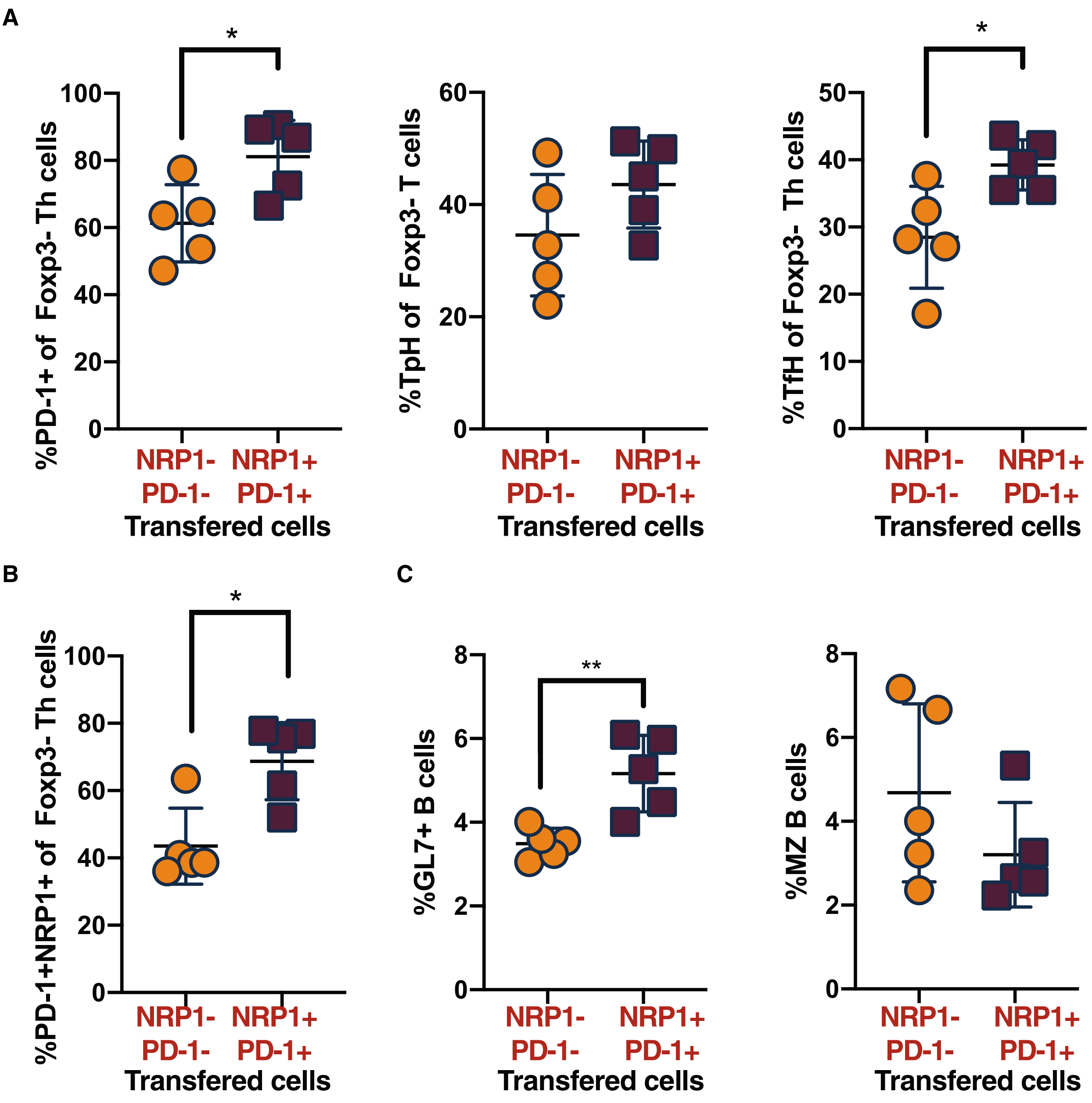

Figure EV5. NRP1/PD-1-expressing Th cells are pathogenic.

A–C Groups of male BXSB.Yaa *Nr4a2* cKO mice aged 18 weeks received either $NRP1^{+}PD\text{-}1^{+}$ $CD4^{+}$ $Foxp3^{-}$ T cells or $NRP1^{-}PD\text{-}1^{-}$ $CD4^{+}$ $Foxp3^{-}$ T cells from 20-week-old donor Male $Foxp3^{hCD2}$ × BXSB.Yaa mice. Four weeks after transfer, spleen cells were analyzed by flow cytometry for $PD\text{-}1^{+}$ T cells among $Foxp3^{-}CD4^{+}$ Th cells (A), $*P < 0.05$ two-tailed unpaired Student's *t*-test. $NRP1^{+}PD\text{-}1^{+}$ cells among $Foxp3^{-}CD4^{+}$ Th cells (B), $*P < 0.05$ two-tailed Mann–Whitney U test, and $GL7^{+}$ GC B cells and $CD21^{+}CD23^{-}$ marginal zone B cells (C), $**P < 0.01$ two-tailed unpaired Student's *t*-test. $n = 5$ mice per group; bars show mean with SD; data are representative of two independent experiments.
