# Supplementary material for: Neuropilin‐1 (NRP1) expression distinguishes self‐reactive helper T cells in systemic autoimmune disease
Source: EMBO Mol Med. 2022 Sep 7;14(10):e15864. doi: 10.15252/emmm.202215864 (PMC9549730; doi:10.15252/emmm.202215864)
Supplement: Supplementary file 3 — PDF+ [file EMMM-14-e15864-s006.pdf]

Article

SOURCE DATA | TRANSPARENT PROCESS | OPEN ACCESS

EMBO Molecular Medicine

Neuropilin-1 (NRP1) expression distinguishes self-reactive helper T cells in systemic autoimmune disease

Ben JE Raveney[1], Yosif El-Darawish[1], Wakiro Sato[1], Yoshiyuki Arinuma[2], Kunihiro Yamaoka[2], Shohei Hori[3], Takashi Yamamura[1] & Shinji Oki[1,*]

Abstract

Pathogenic T helper cells (Th cells) that respond to self-antigen cannot be easily distinguished from beneficial Th cells. These cells can generate systemic autoimmune disease in response to widely expressed self-antigens. In this study, we have identified neuropilin-1 (NRP1) as a cell surface marker of self-reactive Th cells. NRP1$^+$ Th cells, absent in non-regulatory T cell subsets in normal mice, appeared in models of systemic autoimmune disease and strongly correlated with disease symptoms. NRP1$^+$ Th cells were greatly reduced in *Nr4a2* cKO mice, which have reduced self-reactive responses but showed normal responses against exogenous antigens. Transfer of NRP1$^+$ Th cells was sufficient to initiate or accelerate systemic autoimmune disease, and targeting NRP1-expressing Th cells therapeutically ameliorated SLE-like autoimmune symptoms in BXSB-Yaa mice. Peripheral NRP1$^+$ Th cells were significantly increased in human SLE patients. Our data suggest that self-reactive Th cells can be phenotypically distinguished within the Th cell pool. These findings offer a novel approach to identify self-reactive Th cells and target them to treat systemic autoimmune disease.

Keywords neuropilin-1; NR4A2; self-reactive T helper cells; SLE; systemic autoimmune disease

Subject Category Immunology

DOI 10.15252/emmm.202215864 | Received 10 February 2022 | Revised 8 August 2022 | Accepted 15 August 2022 | Published online 7 September 2022

EMBO Mol Med (2022) 14: e15864

Introduction

Systemic lupus erythematosus (SLE), the archetypal systemic autoimmune disorder, is characterized by chronic inflammation accompanied by fever, malaise, joint and muscle pain, and fatigue (Lisnevskaia *et al*, 2014). SLE pathogenesis is associated with autoantibody production against a wide variety of endogenous antigens (Yaniv *et al*, 2015; Tsokos *et al*, 2016). Most autoantibodies in SLE are high-affinity immunoglobulin G class (IgG) indicating production by self-reactive B cells that have undergone somatic hypermutation and class switching driven by self-reactive T helper (Th) cell interactions (Baudino *et al*, 2006; Suurmond & Diamond, 2015). Th cells reacting to self-antigens have been directly implicated in SLE and SLE-like models and thus are a potential target for SLE treatments (Crispin *et al*, 2010; Dong *et al*, 2011). However, therapeutically targeting autoreactive Th cells is challenged by difficulties in discriminating pathogenic self-reactive from non-self-reactive Th cells (Comte *et al*, 2015).

B cell activation and maturation are spurred by interactions with follicular helper T (Tfh) cells in the germinal centers (GC) of lymphoid organs and Tfh cell dysregulation is associated with the development of aberrant B cell responses and systemic autoimmunity (Vinuesa *et al*, 2009; Craft, 2012; Ueno *et al*, 2015; Ueno, 2016). Tfh cells are identified by co-expression of PD-1 and CXCR5; PD-1 plays a key role in Tfh cell activation (Shi *et al*, 2018) and CXCR5 allows Tfh cells to migrate to B cell follicles in lymphoid tissue (Crotty, 2019). Tfh cells secrete interleukin (IL)-21, a critical cytokine controlling B cell maturation and efficient antibody production (Ozaki *et al*, 2002). In addition to Tfh cells, populations of PD-1$^+$ Th cells lacking CXCR5 expression may also contribute to systemic autoimmunity (Rao *et al*, 2017). These cells, termed peripheral helper T (Tph) cells, are expanded in SLE and share features with Tfh cells including *maf* expression and IL-21 production (Bocharnikov *et al*, 2019). Some reports have shown that Tph are more important for driving autoantibody production than classical Tfh cells (Makiyama *et al*, 2019), although their relative contributions to pathogenicity in SLE remain unclear.

Male BXSB.Yaa mice develop a severe spontaneous SLE-like disease characterized by lymphoid hyperplasia, monocytosis, hyper antibody production, including autoantibodies such as anti-nuclear antibodies (ANA), and immune complex-mediated glomerulonephritis. As this disease is enhanced by the presence of the Yaa locus (Y chromosome-associated autoimmune acceleration locus that includes duplication of *tlr7*), pathogenic self-responses predominately

1 Department of Immunology, National Institute of Neuroscience, Tokyo, Japan
2 Department of Rheumatology and Infectious Diseases, Kitasato University School of Medicine, Sagamihara, Japan
3 Laboratory for Immunology and Microbiology, Graduate School of Pharmaceutical Sciences, The University of Tokyo, Tokyo, Japan
*Corresponding author. Tel: +81 042 341 0711 ext. 5243; E-mail: soki@ncnp.go.jp

© 2022 The Authors. Published under the terms of the CC BY 4.0 license.

occur in male mice, although mild disease develops later with age in female mice (Murphy & Roths, 1979). It is known that BXSB.Yaa disease requires IL-21 and Th cells, in particular ICOS-expressing Tfh cells that produce IL-21, and are critical for the development of SLE-like disease in BXSB mice (Chu *et al*, 1996; Bubier *et al*, 2007, 2009; Li *et al*, 2011).

Our previous studies have associated the expression of *Nr4a2* with autoreactive Th cells in patients with the autoimmune disease multiple sclerosis and its mouse model (Doi *et al*, 2008; Raveney *et al*, 2013). Using RNA interference treatment and generating transgenic *Nr4a2* cKO mice with a T-cell-specific *Nr4a2* deficiency, we reported that NR4A2 was required for Th17 cell secretion of IL-21, which in turn promoted autoimmune disease (Raveney *et al*, 2013, 2015). Furthermore, only priming with self-peptide and not with foreign antigens induced aberrant expression of *Nr4a2* in Th cells hinting at a specific association between NR4A2 and autoreactive Th responses (Raveney *et al*, 2013).

In this study, we have shown that male BXSB.Yaa mice lacking *Nr4a2* in T cells were protected from SLE-like disease with reduced self-reactive Th cells. Systemic responses to foreign antigens remained intact in the absence of *Nr4a2*, suggesting that NR4A2 may preferentially control self-reactive immune responses. Research into Th cells in SLE to date focused on Tfh and Tph as pathogenic subsets, which both express PD-1; we now reveal a new cell subset expressing NRP1 as well as PD-1 that transcends these classic subsets. These non-regulatory (conventional) NRP1-expressing Th cells intrinsically bear self-reactive TcRs and act to generate SLE-like disease. Therapeutic targeting of NRP1-expressing cells ameliorated disease indicators, giving future hope for a new approach in treating Th-cell-mediated systemic autoimmune disease, especially as human SLE patients were found to also have increased NRP1-expressing Th cells.

Results

T-cell-specific deletion of the *Nr4a2* gene abrogates systemic autoimmunity in BXSB.Yaa mice

Male BXSB.Yaa mice develop a spontaneous SLE-like disease, resulting from responses against a range of self-antigens. Thus, this model may be more representative of the natural disease course of systemic autoimmunity than many artificial autoimmune models induced against a single antigen. The disease manifests with extreme lymphocyte expansion and enlarged secondary lymphoid organs as well as high levels of pathogenic self-reactive antibodies (Andrews *et al*, 1978; Merino *et al*, 1992). Development of systemic autoimmunity in BXSB.Yaa mice requires IL-21 produced by Th cells to influence self-reactive B cell differentiation (Bubier *et al*, 2007, 2009).

NR4A2 controls IL-21 secretion by Th cells in a model of organ-specific autoimmunity (Raveney *et al*, 2013). To determine whether or not NR4A2 also plays an essential role in IL-21-dependent systemic autoimmune disease, we generated BXSB.Yaa mice with a *Nr4a2* gene deletion in T cells. Backcrossing our previously reported CD4-Cre$^{Tg/+}$ *Nr4a2*$^{fl/fl}$ mice (Raveney *et al*, 2015) to the BXSB.Yaa background provided BXSB.Yaa mice both with a T-specific *Nr4a2* deficiency (Cre-CD4$^{+/-}$ *Nr4a2*$^{fl/fl}$ BXSB.Yaa, BXSB.Yaa *Nr4a2* cKO) and *Nr4a2*-sufficient littermates (*Nr4a2*$^{fl/fl}$ BXSB.Yaa, BXSB.Yaa mice). Male BXSB.Yaa mice developed severe splenomegaly by 16–20 weeks of age similar to wild-type male BXSB.Yaa mice, while BXSB.Yaa *Nr4a2* cKO littermates were significantly protected from lymphoid expansion (Figs 1A–C and EV1A). Male mice lacking NR4A2 were also similar to female BXSB mice that lack the Yaa locus and do not develop the disease (Murphy & Roths, 1979; Merino *et al*, 1992), maintaining normal spleen weight and cellularity (Fig 1B and C).

Splenomegaly in Male BXSB.Yaa mice over 16 weeks of age with intact NR4A2 was associated with increased numbers of a variety of major immune cell populations, including T cells, B cells, and monocyte populations (Fig EV1B–D). This cell expansion was significantly suppressed in Male BXSB.Yaa *Nr4a2* cKO mice, with cell populations remaining similar to pre-disease levels and to female BXSB mice. Not only were B cells generally expanded in Male BXSB.Yaa mice, but GL7^{+} germinal center (GC) B cells were specifically expanded and the proportion of CD21^{+}CD23^{low} marginal zone (MZ) B cells was reciprocally decreased (Fig EV1E and F). These changes that are indicative of disease induction in SLE-like disease were blocked in male BXSB.Yaa *Nr4a2* cKO mice. Instead, male BXSB.Yaa *Nr4a2* cKO mice maintained pre-disease B cell proportions with significantly lower levels of B cell activation than diseased mice as indicated using CD69 level (Fig EV1G). The differences observed in non-T cells, despite the *Nr4a2* deletion being

Figure 1. T-cell-specific deletion of the Nr4a2 gene abrogates systemic autoimmunity in BXSB mice.

Cre-CD4.*Nr4a2*$^{fl/fl}$ mice backcrossed with BXSB.Yaa mice were maintained under SPF conditions until the time point indicated.

A, B Spleens from co-housed littermate male *Nr4a2*$^{fl/fl}$ BXSB.Yaa and Cre-CD4 *Nr4a2*$^{fl/fl}$ BXSB.Yaa (BXSB.Yaa *Nr4a2* cKO) mice were excised, photographed (A), and size measured by weight and total cell number (B). Error bars, SEM for individual mice (n = 4); representative of at least 10 independent experiments, 4–10 per group; ****P < 0.0001, ***P < 0.001. Two-way ANOVA test with Bonferroni's multiple comparison test.

C Representative photographs from 20-week-old female BXSB.Yaa mice are shown.

D–F Serum prepared from 16-week-old male and female BXSB.Yaa or BXSB.Yaa *Nr4a2* cKO mice was assessed by ELISA at the indicated time points for IgG1, IgG2a, and IgG2b (D), comparing total IgG levels from male and female mice aged 16 weeks (E) and IgG2a and IgG anti-nuclear antibody (ANA) for male mice at 16 weeks (F). Time courses in (D) show a representative experiment, n = 4 per group and individual mice from three pooled independent experiments in (E, F), n = 12 per group; (E) ****P < 0.0001, Two-tailed unpaired t-test with Welch's correction; (F) ****P < 0.0001, Two-tailed Mann–Whitney U test. Error bars, SEM.

G Liver proteins were resolved by PAGE and the binding of serum antibodies from four individual mice from each group (numbered 1–4) was detected by anti-IgG secondary antibody.

H Kidney histology of male BXSB.Yaa mice and BXSB.Yaa *Nr4a2* cKO mice aged 24 weeks for H&E, black arrows indicate glomeruli which are more enlarged and inflamed in male BXSB.Yaa mice; periodic acid/Schiff reagent (PAS), arrows indicate pink staining showing glomerular deposition of extracellular matrix components; or Masson's trichrome (MTC), arrows indicate blue staining showing collagen deposition. The black scale bar represents 100 μm, data are representative of at least six individual experiments.

Source data are available online for this figure.

© 2022 The Authors

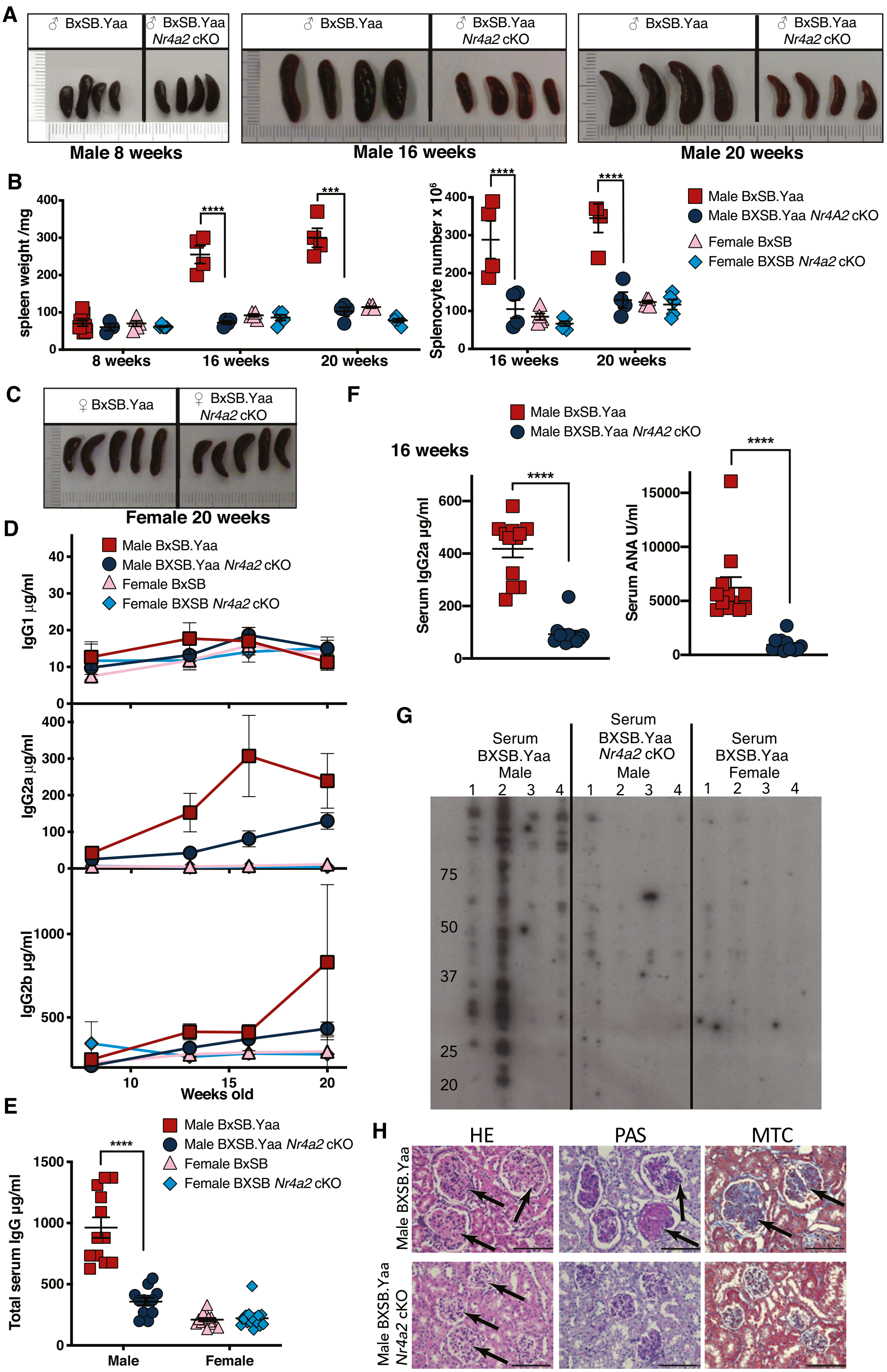
A
♂ BxSB.Yaa
♂ BxSB.Yaa Nr4a2 cKO
Male 8 weeks
Male 16 weeks
Male 20 weeks
B
spleen weight /mg
Splenocyte number x 10^6
8 weeks
16 weeks
20 weeks
Male BxSB.Yaa
Male BXSB.Yaa Nr4A2 cKO
Female BxSB
Female BXSB Nr4a2 cKO
C
♀ BxSB.Yaa
♀ BxSB.Yaa Nr4a2 cKO
Female 20 weeks
D
IgG1 µg/ml
IgG2a µg/ml
IgG2b µg/ml
Weeks old
E
Total serum IgG µg/ml
Male
Female
F
16 weeks
Serum IgG2a µg/ml
Serum ANA U/ml
G
Serum BXSB.Yaa Male
Serum BXSB.Yaa Nr4a2 cKO Male
Serum BXSB.Yaa Female
75
50
37
25
20
H
HE
PAS
MTC
Male BXSB.Yaa
Male BXSB.Yaa Nr4a2 cKO

Figure 1.

T-cell-specific, suggest that the expansion of these populations results from T-cell-related changes.

Autoimmune pathology in male BXSB.Yaa mice is generated by the production of autoantibodies in a similar manner to human SLE (Izui *et al*, 2000; Arbuckle *et al*, 2003). As B cell expansion, activation, and switching to GC B cells were blocked by T-cell-specific deletion of *Nr4a2*, we wished to measure the effects on autoantibody production. Male BXSB.Yaa mice had high IgG serum levels after disease onset, consisting mostly of IgG2a and IgG2b, but no such antibody response developed in Male BXSB.Yaa *Nr4a2* cKO mice or littermate female BXSB mice (Fig 1D and E). In particular, IgG2a and ANA, key pathogenic effector autoantibodies in SLE, were strongly accumulated in Male BXSB.Yaa mice with the disease, but not in Male BXSB.Yaa *Nr4a2* cKO mice (Fig 1F). Interestingly, these serum levels of ANA, IgG2a, and total IgG correlated significantly with spleen size (Fig EV1H).

Although ANA are representative autoantibodies, SLE involves diverse autoantibody responses against a wide variety of self-antigens. To explore this further, we measured the reactivity of serum IgG from 16-week-old male BXSB.Yaa mice by immunoblotting against protein lysates extracted from 11 different male BXSB.Yaa self-organs (Fig EV1I). In particular, serum from male BXSB.Yaa mice bound to multiple liver proteins, while serum derived from male BXSB.Yaa *Nr4a2* cKO mice or female mice was less reactive (Fig 1G). Progressive glomerulonephritis caused by the renal accumulation of immune complexes is a typical feature of the disease in BXSB.Yaa mice (Andrews *et al*, 1978). Multiple pathologies were observed in the kidney of 16-week-old male BXSB.Yaa mice, including glomerular basement membrane hyperplasia, strong perivascular inflammation, focal glomerular fibrosis, and glycoprotein deposition, while these features were milder in male BXSB.Yaa *Nr4a2* cKO littermates (Fig 1H).

Together our data show a T-cell-specific deletion of the *Nr4a2* gene thoroughly blocked onset of SLE-like disease hallmarks in Male BXSB.Yaa mice, suggesting that NR4A2-expressing Th cells are intrinsically involved in the development of autoreactive responses in this disease.

Rapid expansion of diverse Th cell subsets in male BXSB.Yaa mice is NR4A2 dependent

SLE-like disease in male BXSB.Yaa mice has been associated with Tfh cells (Bubier *et al*, 2009), therefore we further scrutinized T cells to determine if NR4A2 had effects on particular subsets. NR4A2-dependent T cell expansion was mainly attributed to $CD4^+$ Th cells, with little increase in $CD8^+$ Tc cells (Fig 2A). Ninety percent of Th cells in 16-week-old male BXSB.Yaa mice expressed PD-1, a surface receptor found on Tfh cells, versus < 50% in male BXSB.Yaa *Nr4a2* cKO mice (Fig 2B). The number of cells in this fraction co-expressing the Tfh-related chemokine receptor CXCR5 was also greater in male BXSB.Yaa mice. However, PD-1^+ Th cells lacking CXCR5 co-expression were also significantly increased in male BXSB.Yaa spleens (Fig 2C). A similar population of PD-1^+CXCR5^- extrafollicular Th cells, termed Tph cells, have been reported as driving autoantibody production in a mouse model of rheumatoid arthritis and in human SLE (Odegard *et al*, 2008; Rao *et al*, 2017; Lin *et al*, 2019; Makiyama *et al*, 2019). In male BXSB.Yaa, we find that such Tph cells predominate over Tfh cells at the onset of systemic autoimmunity and are maintained throughout the disease, yet are virtually absent in young male BXSB.Yaa mice and female BXSB mice (Fig EV2A and B). Interestingly, the splenic Tph cell level correlated with splenomegaly more significantly than Tfh cells (Fig 2D) and a similar finding for Tph correlation with serum levels of ANA and IgG2a, but IgG1 and IgG2b had no correlation with either cell subset (Fig EV2C and D). Th cell subsets in blood had different kinetics with blood Tfh cells increasing at a later time point, whereas the increase in blood Tph cells occurred in early disease and mirrored increases in serum ANA and total IgG2a levels (Fig 2E and F). In male BXSB.Yaa *Nr4a2* cKO mice, circulating Tfh and Tph cell expansion was abrogated and ANA production and increases in IgG2a titer were suppressed at all time points. The blood level of Tph cells correlated well with the level in the spleen (Fig EV2E), but for Tfh cells, correlation was lower between sites, presumably as CXCR5 expression targets Tfh cells to the spleen. Splenic Tph and Tfh cells both produced IL-21 on stimulation, but Tph had a significantly higher proportion of IL-21^+ cells (Fig 2G). IL-21 producers were significantly reduced in male BXSB.Yaa *Nr4a2* cKO mice, particularly in the Tph subset. Similar findings were noted at the mRNA level for *Il21* (Fig EV2F). Transcripts of Tfh-related markers, including the key transcription factor *Maf* and also *Blimp1*, were similar for Tfh and Tph cells, but with high expression compared with PD-1^- cells and reduced level in male BXSB.Yaa *Nr4a2* cKO mice (Fig EV2G).

It is somewhat surprising that disease manifestations were more closely linked to Tph cells rather than Tfh cells, as previous reports highlighted a close link between ICOS/CXCR5 expression in Th cells

Figure 2. Rapid expansion of diverse Th cell subsets in BXSB.Yaa mice is Nr4a2 dependent.

A–C Splenocytes from 16-week-old male and female BXSB.Yaa or BXSB.Yaa *Nr4a2* cKO littermate mice were assessed by flow cytometry for $CD4^+$ Th (TcRβ^+CD4^+CD8^-) and $CD8^+$ Tc (TcRβ^+CD4^-CD8^+) cell number per spleen (A); PD-1/ICOS among TcRβ^+CD4^+ cells (representative staining and PD-1^+ proportion, male mice, B), representative PD-1/CXCR5 staining for TcRβ^+CD4^+ cells (C, left) to determine PD-1^- (PD-1^-CXCR5^-), Tph (PD-1^+CXCR5^-), and Tfh (PD-1^+CXCR5^+) cell subsets and measure mean cell number of each subset per spleen (C, right upper) and proportion among TcRβ^+CD4^+ T cells (C, right lower). Error bars, SEM for individual mice, $n = 4$. Data are representative of at least five independent experiments with 4–10 mice per group; ****$P < 0.0001$, ***$P < 0.001$, and *$P < 0.05$ two-way ANOVA test with Bonferroni's multiple-comparison test comparing *Nr4a2* cKO effect for each subset.

D For individual mice, Tph and Tfh cells' cell percentage in splenic Th cells was compared with spleen weight; linear regression lines shown with 95% CI; $n = 15$ per group across three independent experiments; Spearman R correlation statistics indicated.

E, F Serum antibody levels for male BXSB.Yaa and male BXSB.Yaa *Nr4a2* cKO at the indicated times were compared with Th cell subsets. The left axes indicate Tph or Tfh cell percentage; the right axes indicate serum ANA (E) or IgG (F) level. $n = 8$ per group, representative of three independent experiments; error bars, SEM.

G IL-21 was measured in splenocytes from 16-week-old male mice by intracellular flow cytometry following restimulation PD-1^-, Tph, and Tfh subsets $n = 5$; data are representative of two independent experiments; error bars, SEM, ****$P < 0.0001$, *$P < 0.05$, two-way ANOVA test with Bonferroni's multiple-comparison test.

H CXCR4 expression was measured by flow cytometry for each subset. Representative CXCR4 staining (left) and geometric mean fluorescent intensity (MFI) for each group (right) are shown. $n = 3$ individual mice; data are representative of three similar experiments, error bars, SEM.

© 2022 The Authors

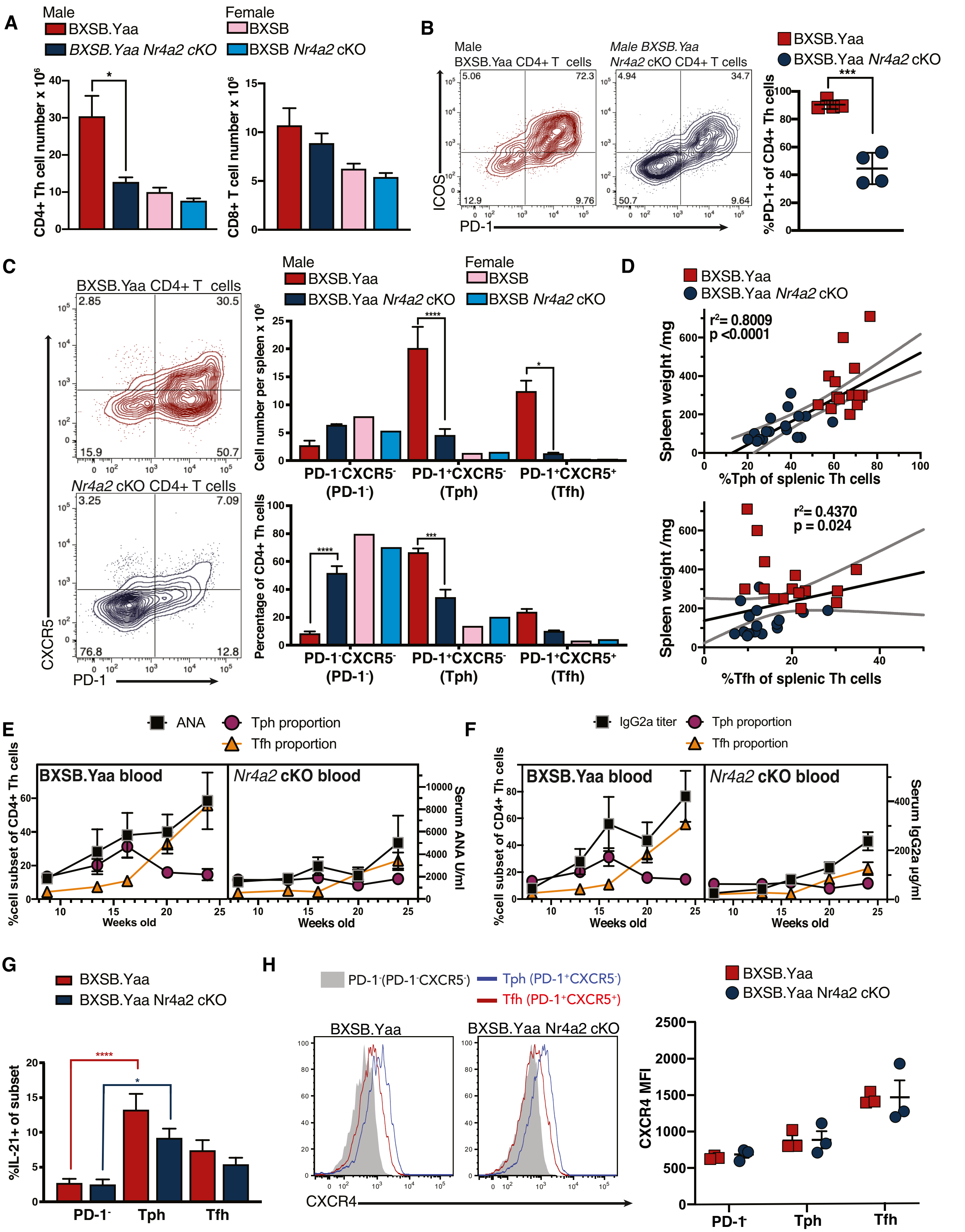
A
Male
BXSB.Yaa
BXSB.Yaa Nr4a2 cKO
Female
BXSB
BXSB Nr4a2 cKO
CD4+ Th cell number x 10^6
CD8+ T cell number x 10^6
B
Male BXSB.Yaa CD4+ T cells
Male BXSB.Yaa Nr4a2 cKO CD4+ T cells
ICOS
PD-1
%PD-1+ of CD4+ Th cells
C
BXSB.Yaa CD4+ T cells
Nr4a2 cKO CD4+ T cells
CXCR5
Cell number per spleen x 10^6
Percentage of CD4+ Th cells
PD-1-CXCR5- (PD-1-)
PD-1+CXCR5- (Tph)
PD-1+CXCR5+ (Tfh)
D
r2= 0.8009
p <0.0001
Spleen weight /mg
%Tph of splenic Th cells
r2= 0.4370
p = 0.024
%Tfh of splenic Th cells
E
ANA
Tph proportion
Tfh proportion
BXSB.Yaa blood
Nr4a2 cKO blood
%cell subset of CD4+ Th cells
Serum ANA U/ml
Weeks old
F
IgG2a titer
Serum IgG2a µg/ml
G
%IL-21+ of subset
PD-1-
Tph
Tfh
H
PD-1-(PD-1-CXCR5-)
Tph (PD-1+CXCR5-)
Tfh (PD-1+CXCR5+)
BXSB.Yaa Nr4a2 cKO
CXCR4
CXCR4 MFI

Figure 2.

and spleen cell number in male BXSB.Yaa mice. It has been reported that high-affinity autoantibody production can be activated extrafollicularly, with B cells interacting with CXCR4⁻-expressing Th cells rather than Tfh cells in GC (Odegard *et al*, 2008). Both Tfh cells and Tph cells expressed CXCR4 to a similar level indicating that CXCR4 expression was independent of NR4A2 (Fig 2H).

Humoral responses against exogenous antigens are independent of NR4A2

Reduced autoantibody production in male BXSB.Yaa *Nr4a2* cKO mice might result from a global defect in Th cell responses which in turn limits B cell help. It has been reported that IgG responses against the exogenous antigen can be stimulated separately to autoantibody production (Niu *et al*, 2008). Therefore, we tested if the defective T-cell-dependent antibody production we observed in spontaneous SLE-like disease was also reconstituted during T-cell-mediated priming of antibody responses to exogenous antigens in the absence of NR4A2. High- and low-affinity NP-specific IgG1and IgG2a responses resulting from NP-KLH immunization were NR4A2 independent (Fig 3A and B). Furthermore, there was no difference in ratio between high- and low-affinity antibodies against NP moiety with/without *Nr4a2* indicating somatic hypermutation occurred to the same extent (Fig 3C). NP-KLH immunization of mice on a C57BL/6 background, which do not develop spontaneous autoimmune disease, led to similar levels of Th cells (red) entering splenic B cell follicles (green, Fig 3D; Appendix Fig S1A) with/without *Nr4a2* in T cells and similar formation of germinal centers within lymphoid follicle structures (Green, Fig 3E; Appendix Fig S1B). Furthermore, anti-NP antibody levels induced by the NP-KHL immunization were not reduced in the absence of *Nr4a2* (Fig 3F). As another measure of Th-dependent humoral responses to exogenous antigen, we measured fecal IgA levels, which are produced in responses to bacterial antigens (Kawamoto *et al*, 2012; Cao *et al*, 2015); these did not differ between C57BL/6 WT and C57BL/6 *Nr4a2* cKO mice (Fig 3G).

These data indicating normal antibody production in the absence of NR4A2 contrasts with our findings showing reduced autoantibody production in male BXSB.Yaa *Nr4a2* cKO mice. Taken together, T-cell-dependent humoral responses against exogenous antigens may be intrinsically distinct from autoantibody production and with NR4A2 exclusively required for T-cell-dependent IgG production against self-antigen.

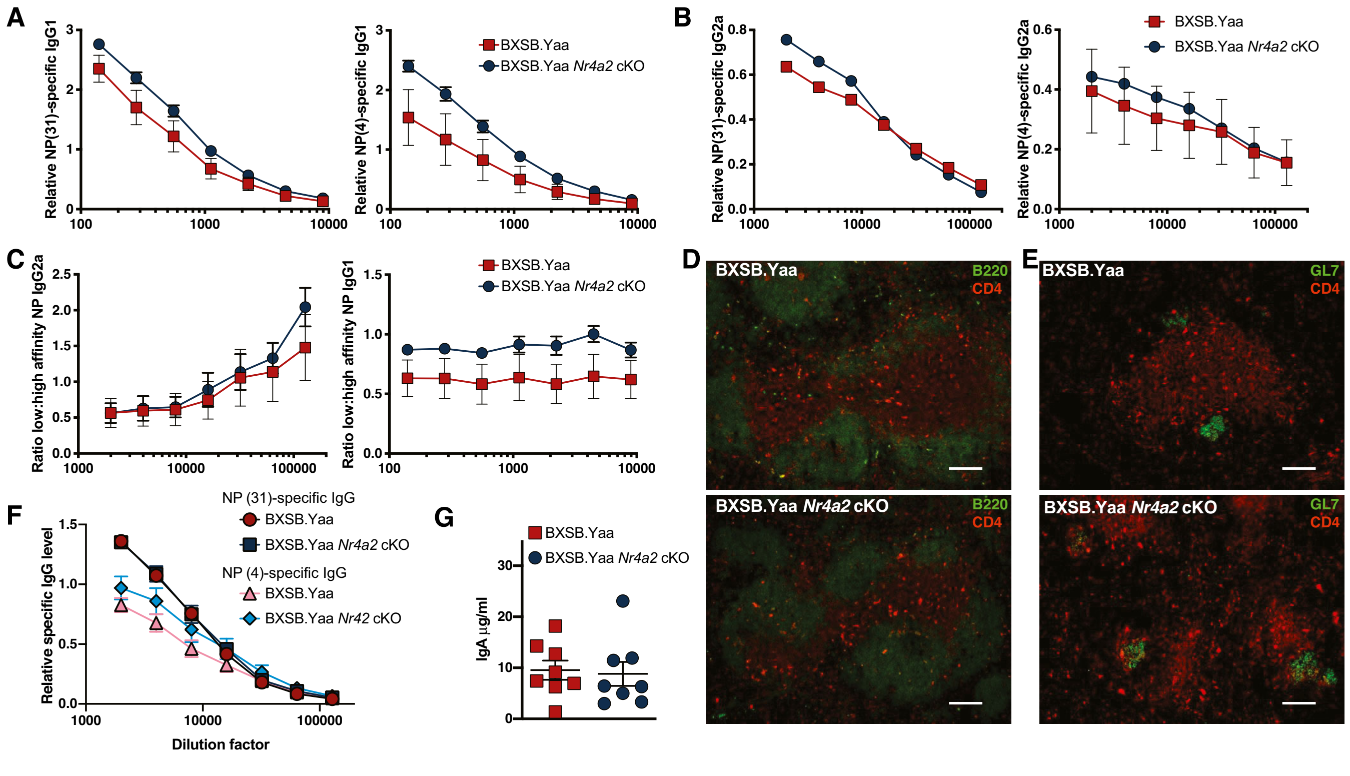

Figure 3. Humoral responses against exogenous antigens are independent of Nr4a2.

A–C Young male BXSB.Yaa and BXSB.Yaa *Nr4a2* cKO mice were immunized twice with NP-KLH emulsified in alum. Serum was assessed for the level of antibodies binding NP-31 (left) and NP-4 (right) epitopes by ELISA across a range of diluted serum samples with IgG1-specific (A) and IgG2a-specific antibodies (B). The ratio for low- to high-affinity (NP-4:NP-31) antibodies was calculated for IgG2a (left) and IgG1 (right) antibodies (C). Error bars, SEM, n = 5 mice per group; $P > 0.1$ two-way ANOVA.

D–F Male C57BL/6 Nr4a2 cKO or wild-type littermate C57BL/6 mice were immunized twice with NP-KLH emulsified in alum. After 2 weeks, 7 μm spleen sections were stained with Red:Alexa-Fluor-594-CD4 and Green: FITC-B220 (D) or FITC-GL7 (E). The scale bar represents 100 nm. The serum was assessed for the level of IgG antibodies binding NP-31 and NP-4 (F).

G Stool samples from young male BXSB.Yaa and BXSB.Yaa *Nr4a2* cKO mice were measured for IgA concentration by ELISA. Points are samples from individual mice, error bars, SEM, n = 8 from two independent experiments; $P > 0.1$ unpaired Student's t-test.

© 2022 The Authors

Self-reactive T cell pool is associated with NR4A2-dependent Th cells

Our data suggest that NR4A2 influences pathogenic responses to self-antigen in male BXSB.Yaa mice by controlling self-reactive Th cells. T cells escaping central tolerance can allow peripheral expression of T cell receptors (TcR) specific for self-antigen that are potentially autoreactive (Yu *et al*, 2015). To date, research into T cells that respond to self-antigens has been hampered by an inability to discern such self-reactive T cells from the useful T cell pool. However, a recent study highlighted a TcR feature linked to self-reactivity: hydrophobic amino acid doublets in positions 6 and 7 (P6-7) in the CDR3 region of a TcR were associated with promoting self-reactivity, while hydrophilic P6-7 doublets limit self-reactivity (Stadinski *et al*, 2016). Hydrophobic TcR CDR3 apexes were also associated with self-reactive TcRs in mouse regulatory cells and TcRs with this attribute were also higher in human subjects with RAG mutations with resulting increase in self-reactive T cells (Daley *et al*, 2019). As we wished to examine potential self-reactive T cells, we performed TcR CDR3 repertoire sequencing of memory Th cells from male BXSB.Yaa mice to identify T cell clones with these hallmarks of self-reactivity.

Male BXSB.Yaa and BXSB.Yaa *Nr4a2* cKO mice had extensive and similar TcR diversity with a similar distribution of TcR clones (Fig 4A). Clones were ranked by read number for each genotype and the CDR3 sequence was assessed for self-reactivity limiting/promoting hallmarks (Appendix Table S1). The most common TcRβ CDR3 sequences with the highest read numbers represent the most expanded Th clones, therefore our investigation focused on such clones as these would represent the most relevant Th cell repertoire for ongoing disease. For example, the top 50 clones, represented at least 25% of the memory cell pool (Fig 4B). Self-reactive promoting TcRs among these 50 most common clones were greatly reduced in male BXSB.Yaa *Nr4a2* cKO mice (Fig 4C). Similar results were observed across the top 25, 100, and 200 clones (covering ~ 15, 40, and 50% of repertoire reads, respectively), with reduced self-reactive promoting clones in BXSB.Yaa *Nr4a2* cKO mice, but similar levels of limiting clones between genotypes (Fig 4D). These data indicate a selective loss of self-reactive T cells from the periphery in the absence of NR4A2.

Disease-associated PD-1$^+$ Th cells share markers with regulatory T cells

The detection of self-reactive cells by cell-intrinsic hallmarks is somewhat of a "holy grail" in autoimmune research and using the properties of TcR CDR3 residues gives tantalizing hints at resolving this issue. To corroborate our finding that self-reactive Th cells were

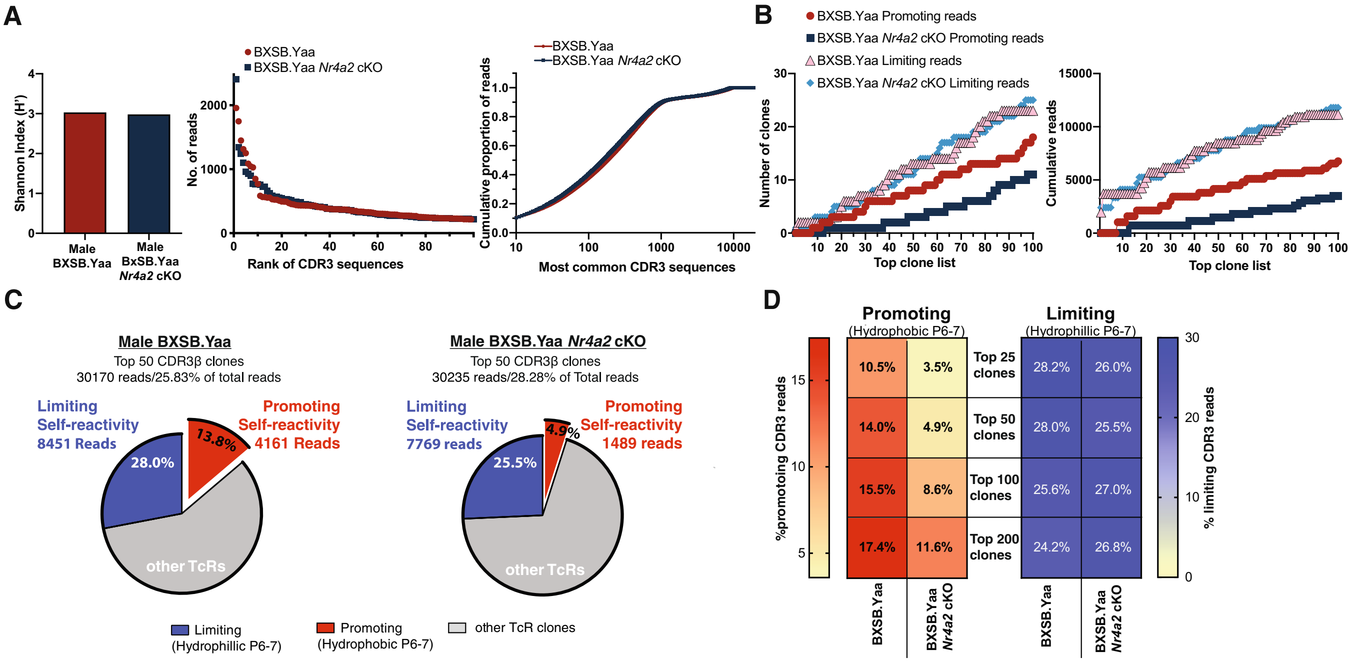

Figure 4. Expanded Th cell clones with hallmarks of TcRs associated with promoting self-reactivity are reduced by *Nr4a2* deletion.

A Splenic memory T cells (TcRb$^+$CD4$^+$CD44^{hi}CD62L^{lo}) sorted from 20-week-old male BXSB.Yaa and BXSB.Yaa *Nr4a2* cKO were sequenced for unbiased TCR repertoire analysis. Unique TcR sequences were ordered by most common clones with summary diversity statistics calculated and the cumulative read proportion across all unique sequences ranked by most common CDR3 is shown for each genotype.

B Sequences were categorized based on residues CDR3 positions 6 and 7: hydrophobic P6/7 doublets (promoting self-reactivity) and hydrophilic doublets (limiting self-reactivity). Unique TcR sequences were ordered by most common clones based on read number, and the proportion of promoting and limiting self-reactivity clones for the top 100 clones of each genotype is shown by the number of clones (left) and cumulative reads (right).

C, D The charts in (C) show the proportion of promoting and limiting self-reactivity clones for each genotype for the top 50 clones, and the proportion of promoting and limiting self-reactivity clones for the top 25, 50, 100, and 200 most common sequences by read number is shown by the heat map in (D).

Source data are available online for this figure.

© 2022 The Authors

reduced in male BXSB.Yaa *Nr4a2* cKO mice, we looked to another T cell population that responds to self-peptides for potential self-reactive T cell hallmarks: regulatory T (Treg) cells (Hsieh *et al*, 2006). Interestingly, Treg cells also upregulate PD-1 (Francisco *et al*, 2009), thus we investigated if other surface markers were also shared between Treg and pathogenic PD-1$^+$ Th cells.

Glucocorticoid-induced TNFR-related protein (GITR, also known as CD357 and TNFRSF18) has previously been reported as a marker of Treg cells (McHugh *et al*, 2002; Shimizu *et al*, 2002) and in particular, PD-1$^+$GITR^{hi} Treg subsets have been reported to include T cell populations with self-reactive TcRs (Wyss *et al*, 2016). We measured GITR expression in Th cells subsets in male BXSB.Yaa mice after disease onset using the hallmark Treg transcription factor Foxp3 to separate non-Treg conventional Th cells from Treg cells. We observed high numbers of PD-1$^+$GITR^{hi} cells among conventional non-Treg cells, as defined by lack of Foxp3 expression (Fig EV3A and B). This expansion of Foxp3$^-$PD-1$^+$GITR^{hi} Th cells was NR4A2 dependent and appeared to be associated with the development of self-reactive responses as few PD-1$^+$GITR^{hi} were observed among conventional non-Treg Th cells in *Nr4a2* cKO littermates (Fig EV3B), or in mice on a background that do not develop spontaneous autoimmune disease (Fig EV3C and D). This expansion of PD-1$^+$GITR^{hi} in male BXSB.Yaa mice was confined to conventional cells as Treg cell numbers and Foxp3$^+$ PD-1$^+$GITR^{hi} Treg subsets were similar between genotypes (Fig EV3D).

NRP1 co-expression with PD-1 identifies a Th cell subset that correlates with SLE-like BXSB.YAA autoimmune disease

While our observation of GITR expression on disease-associated conventional Th cells confirmed our hypothesis that self-reactive pathogenic and Tregs may share markers, GITR was detected as a shift and so not suitable as a marker of potential self-reactive Th cells. Therefore, we sought additional receptors usually expressed by Treg cells that might prove more appropriate. Neuropilin-1 (NRP1; CD304) is a receptor expressed on Treg cells (Bruder *et al*, 2004) that has also been linked with anergic T cells responding to self-peptide (Kalekar *et al*, 2016). However, NRP1 expression on non-Treg conventional Th cells has not been extensively studied, but one report links NRP1 with Tfh cells (Renand *et al*, 2013). We now observe that the onset of disease in male BXSB.Yaa mice led to a massive expansion of NRP1$^+$ PD-1$^+$ Foxp3$^-$ conventional Th cells in the spleen (Fig 5A and B). This expansion of NRP1$^+$PD-1$^+$ Th cells was suppressed in the absence of NR4A2 in male BXSB.Yaa mice (Fig 5B), whereas no changes were seen in NRP1/PD-1 levels among Treg cells in male BXSB.Yaa spleens during disease onset (Fig 5C).

This increase in NRP1 expression in non-Treg Th cells is not observed in standard mice that are not prone to autoimmunity when the steady state is maintained during aging. In C57BL/6 background mice, NRP1$^+$PD-1$^+$ Th cells remained confined to Treg populations with few conventional Th cells expressing NRP1 (Fig EV4A).

Together, these data suggest that the expansion of NRP1$^+$ conventional Th cells in male BXSB.Yaa mice is related to the onset of systemic autoimmunity rather than aging and implicates NRP1$^+$ Th cells as pathogenic. This is further supported by the level of NRP1$^+$PD-1$^+$ conventional Th cells in male BXSB.Yaa, which is strongly correlated with systemic autoimmunity as indicated by splenomegaly and autoantibody production (Fig 5D).

NRP1$^+$PD-1$^+$ conventional Foxp3$^-$ Th cells from male BXSB.Yaa mice had more restricted repertoires than Th cells that did not express PD-1, with lower-sequence diversity and higher coverage ratios of the most frequent TcR CDR3 sequences (Fig EV4B and C). The proportion of self-reactivity promoting P6-7 doublets of TcRβ CDR3 was somewhat increased in NRP1/PD-1$^-$-expressing Th cells over PD-1$^-$ Th cells (Fig EV4D). These differences were apparent in both male BXSB.Yaa and Male BXSB.Yaa *Nr4a2* cKO, thus the reduced self-reactive T cell population observed in the absence of NR4A2 was due to a differential expansion of self-reactive Th cells rather than a skewing of TcR receptor selection away from self-reactive TcRs. The increase in oligoclonality and self-reactivity promoting clones supports the concept that NRP1$^+$PD-1$^+$ cell populations result from expanded self-reactive cells in disease.

In addition, CD5 expression upregulation, which has been associated with T cells expressing TcRs specific for self (Henderson *et al*, 2015; Zinzow-Kramer *et al*, 2019), was increased in diseased male BXSB.Yaa mice. With NRP1$^+$PD-1$^+$ conventional Th cells having significantly higher CD5 levels as compared with PD-1$^-$ Th cells (Fig EV4E). We also noted that most NRP1$^+$ Foxp3$^-$ Th cells in male BXSB.Yaa Mice were CXCR5$^-$ and so found among the Tph fraction, but some Tfh cells also co-expressed NRP1 (Fig EV4F); these proportions were decreased in the absence of *Nr4a2*.

Collectively, these data suggested that NR4A2 in T cells allows the expansion of Th cells bearing potentially self-reactive TcRs, and this expanded subset is linked to NRP1 upregulation.

In addition to NRP1$^+$ PD-1$^+$ Th cells, there was also a minor increase in NRP1$^-$PD-1$^+$ Th cells in the Foxp3$^-$ subset, with slightly higher levels in diseased male BXSB.Yaa mice versus BXSB.Yaa *Nr4a2* cKO mice (Appendix Fig S2A). Whereas NRP1$^+$ Th cells correlate well with measures of SLE-like disease in male BXSB.Yaa mice (Fig 5D), these NRP1$^-$PD-1$^+$ Th cells only weakly correlate (Appendix Fig S2B). Over time, there was little expansion of these cells within the Foxp3$^-$ fraction (Appendix Fig S2A) versus NRP1$^+$PD-1$^+$ conventional Th cells, which were increasing (Fig 5B), supporting the concept that it is NRP1 rather than PD-1 which is the key marker for pathogenic self-reactive Th cell in this disease model.

NRP1-expressing conventional Th cells are expanded in a range of SLE mouse models

The unusual increase in conventional Th cells expressing NRP1 seen in male BXSB.Yaa mice developing SLE-like disease was also apparent in other models that are prone to systemic autoimmune diseases (Fig 5E–G). On the C57BL/6 background, NRP1 expression was mostly confined to Treg cells at 20–24 weeks of age in wild-type mice (Fig 5F). Aire knockout mice (Aire$^{-/-}$), which were developed to study the disease autoimmune polyendocrinopathy-candidiasis-ectodermal dystrophy that results from a mutation in AIRE, are prone to mild systemic autoimmune disease symptoms on the C57BL/6 background (Anderson *et al*, 2002). Aire$^{-/-}$ mice had a significant increase in NRP1 expression in conventional Foxp3$^-$ Th cells compared with age-matched wild-type (WT) C57BL/6 mice (Fig 5F).

Mice on a MRL background develop SLE-like autoimmune disease with splenomegaly, production of autoantibodies, and increased immune complex formation, leading to severe proliferative glomerulonephritis. A mutation in the Fas gene in MRL.LPR

 © 2022 The Authors

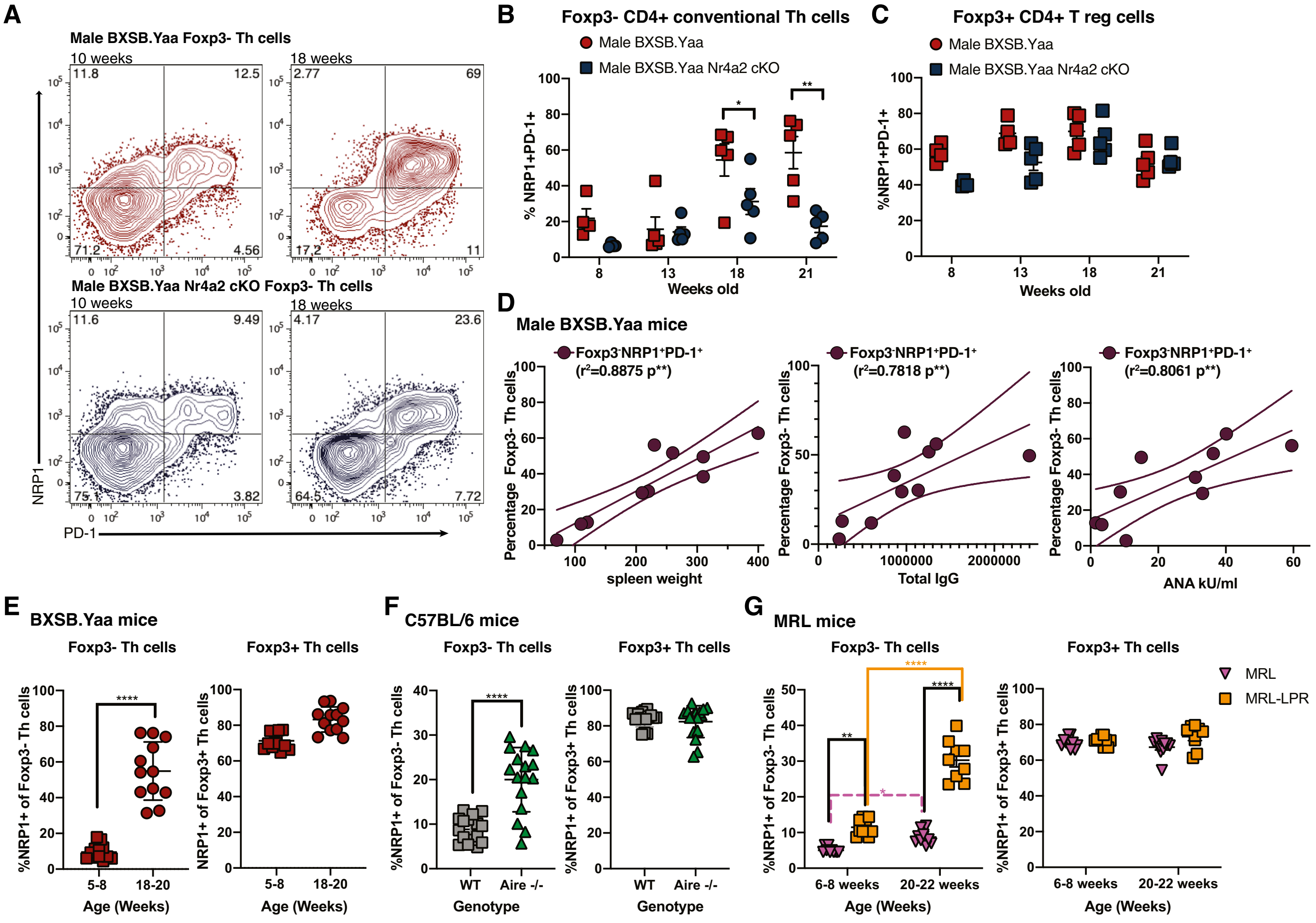

Figure 5. NRP1 expression identifies a Nr4a2-dependent self-reactive Th cell subset expanded in SLE.

A–C Splenocytes from male BXSB.Yaa and BXSB.Yaa *Nr4a2* cKO were assessed by flow cytometry for NRP1 and PD-1 expression in Foxp3$^+$ or Foxp3$^-$ CD4$^+$TcRβ$^+$ subsets: representative staining at 10 and 18 weeks old (A), proportions of NRP1$^+$PD-1$^+$ cells at 8–21 weeks of age in Foxp3$^-$ Th cells (B) or Foxp3$^+$ Th cells (C). n = 5 per group from at least two independent experiments; error bars, SEM; $^{**}P < 0.01$; $^{*}P < 0.05$ two-tailed Mann–Whitney U test.

D The proportion of NRP1$^+$PD-1$^+$ cells (Y-Axis) from splenic TcRb$^+$CD4$^+$Foxp3$^-$ of 18-week-old male BXSB.Yaa and BXSB.Yaa *Nr4a2* cKO was compared with (X-axis) spleen weight (left), and serum levels of IgG (center) and ANA (right). n = 8, linear regression line with 95% CVs shown; data are representative of two independent experiments.

E–G NRP1-expressing proportion was measured by flow cytometry among TcRβ$^+$CD4$^+$Foxp3$^-$ Th cells and TcRβ$^+$CD4$^+$Foxp3$^+$ Th cells in mouse strains prone to systemic autoimmunity. Male BXSB.Yaa mice with SLE-like disease aged 18–20 weeks (n = 12) were compared with pre-disease 5- to 8-week-old male BXSB.Yaa mice (n = 12) (E); Male Aire knock-out mice (Aire$^{-/-}$; n = 16) were compared with male wild-type mice (WT; n = 16), both on a C57BL/6 background and 20–24 weeks (F); Lupus-prone female MRL-LPR mice (n = 10) were compared with female MRL mice (n = 10) at early disease 6–8 weeks and established disease 20- to 22-week-old MRL.LPR (n = 10) with mild disease 20- to 22-week-old MRL (n = 10) (G). For BXSB.Yaa and C57BL/6 background mice, the Foxp3$^-$ subset was determined by surface staining of a transgenic reporter, Foxp3^{hCD2}; for MRL background mice, the Foxp3$^-$ subset was determined by intracellular Foxp3 staining. Error bars, SD $^{****}P < 0.0001$, $^{**}P < 0.01$, $^{*}P < 0.05$, Two-tailed unpaired t-test with Welch's correction for BXSB.Yaa and C57BL/6 background plots, and two-way ANOVA analysis with Bonferroni's multiple-comparison test. Data are pooled from individual mice from at least two independent experiments.

mice leads to lymphoproliferation which greatly accelerates disease, but a slowly developing autoimmune disease also develops in MRL mice without the LRP mutation (Andrews *et al*, 1978; Izui *et al*, 1984; Theofilopoulos & Dixon, 1985). Furthermore, this model has more accelerated disease in females over males (Izui *et al*, 1984) and thus is often used to model human SLE which has a strong female sex bias (Pons-Estel *et al*, 2010). We observed such a disease in MRL.LPR mice was associated with an early increase in NRP1$^+$ Foxp3$^-$ Th cells at 6–8 weeks over MRL mice which was greatly enhanced by 20–22 weeks of age (Fig 5G), whereas NRP1 expression in Tregs remained stable. We also observed a slight increase in NRP1$^+$ cells among Foxp3$^-$ Th cells in MRL mice at 20–22 weeks, which may relate to the onset of the weaker systemic autoimmunity associated with non-LPR MRL mice (Fig 5G). Interestingly, disease in MRL.LPR is also associated with an increase in unusual CD4$^-$CD8$^-$ T cells, a feature which leads to the severe lymphadenopathy in this model, but is not observed in human SLE (Morse *et al*, 1982; Cohen & Eisenberg, 1991); we observed that these cells were mostly PD-1$^+$, but only marginal NRP1 upregulation (Fig EV4G).

Overall, it appears that intrinsically self-reactive Th cells in systemic autoimmune disease may have shared phenotypes with Treg

© 2022 The Authors

cells, with NRP1$^+$ conventional Th cells highly associated with autoimmune disease induction in NR4A2-dependent manner.

Interestingly, we also detected Foxp3$^-$ Th cells that expressed NRP1 in the target organ during experimental autoimmune encephalomyelitis (EAE), an induced mouse model of autoimmune neuroinflammatory disease that is used as an analog of human multiple sclerosis. Here, Th responses are induced by immunization of C57BL/6 mice with the CNS antigen MOG (myelin oligodendrocyte glycoprotein), leading to infiltration of the CNS with pathogenic Th cells that generate local damage. At peak disease, a significant proportion of CNS-infiltrating Foxp3- Th cells had acquired NRP1 expression, making up a third of all CNS-infiltrating Th cells (Fig EV4H). Unlike in systemic autoimmune disease, these NRP1$^+$ Th cells did not express PD-1 (Fig EV4I), suggesting NRP1 expression in autoimmune disease is not only limited to Tph and Tfh cells. Indeed, B cell responses are not required in EAE pathogenesis (Lyons *et al*, 1999), which instead requires pathogenic responses by populations of Th1 and Th17 cells responding to CNS antigens, infiltrating the CNS, and generating damage in this target organ (Jager *et al*, 2009; Loos *et al*, 2020).

When CNS-infiltrating Th cells were restimulated with the immunizing peptide, these CNS Foxp3$^-$ NRP1$^+$ Th cells elaborated IFN-γ and IL-17, the hallmark cytokines produced by Th1 and Th17 cells (Fig EV4J). These data indicate that peptide-specific NRP1$^+$ Th1 and Th17 are induced in EAE and support the concept that self-reactive NRP1$^+$ Th cells may be pathogenic irrelevant to PD-1 expression.

Overall, these data indicate that NRP1 expression by non-Treg Th cells in autoimmunity may extend further than PD-1$^+$ Tfh/Tph cells in systemic autoimmune disease. Furthermore, such data provide indications that these cells are induced and respond to self-peptides. However, an outstanding question remains—are these NRP1$^+$ Th cells that develop in the context of systemic autoimmune disease involved in pathogenesis?

NRP1/PD-1-expressing Th cells are pathogenic

As Foxp3$^-$ Th cells expressing both NRP1 and PD-1 were associated with autoimmune disease, we next tested whether or not NRP1/PD-1-expressing conventional Th cells were directly pathogenic. To discriminate live non-Treg cell subsets from Treg, we backcrossed a reporter mouse that expresses surface human CD2 under the control of Foxp3 to a BXSB.Yaa background; identifying Foxp3$^+$ Tregs more clearly than intracellular Foxp3 staining (Fig 6A). Using this reporter, we flow cytometrically sorted NRP1/PD-1 and PD-1$^-$ conventional (Foxp3$^-$) Th cells and tested them for pathogenicity by transfer into young male BXSB.Yaa mice prior to usual disease onset (Fig 6B and C). Transfer of NRP1$^+$ Th cells generated a rapid increase in serum antibodies (Fig 6D) and increased splenomegaly (Fig 6E), whereas disease markers for recipients of PD-1$^-$ conventional Th cells remained equivalent to littermate mice without cell transfer.

Next, we then assessed if these cells were sufficient to generate autoimmunity where disease onset is blocked by a lack of NR4A2 in T cells. We transferred NRP1$^+$ conventional Th cells or PD-1$^-$ conventional Th cells into 18-week-old male BXSB.Yaa *Nr4a2* cKO mice and measured disease signs induced by transfer of potentially pathogenic cells. Transfer of NRP1$^+$PD-1$^+$ Foxp3$^-$ Th cell elicited disease progression in protected male BXSB.Yaa *Nr4a2* cKO mice, with splenomegaly (Fig 6F and G) and increases in serum antibodies, including ANA (Fig 6H). These disease signs were accompanied by an expansion of NRP1$^+$PD-1$^+$ Th cells in the conventional Th cell pool (Fig EV5A and B). Furthermore, this transfer of T cells also led to increased B cell activation and an increase in GC B cells with a reciprocal decrease in MZ B cells (Fig EV5C).

These data show a surprising expansion of non-regulatory T cells co-expressing NRP1$^+$PD-1$^+$ associated with systemic autoimmune disease. These NRP1$^+$PD-1$^+$ Th cells from diseased donor mice generated disease in both young male BXSB.Yaa and male BXSB.Yaa *Nr4a2* cKO recipient mice upon transfer. Taken together, we conclude that these NRP1$^+$PD-1$^+$ conventional Th cells comprise pathogenic subsets with an implicit self-reactivity that drives systemic autoimmune disease.

Therapeutic intervention targeting NRP1 ameliorates systemic autoimmunity

As we have identified a pathogenic population of Th cells that can induce SLE-like disease, we aimed to explore if targeting these cells could provide a new avenue for the treatment of systemic autoimmunity. Targeting PD-1 has been used in cancer immunotherapy by enhancing anti-tumor immune responses (Zou *et al*, 2016), and ligation of PD-1 with PD-L1Ig inhibits T cell activation (Freeman *et al*, 2000). We also observed that PD-L1Ig treatment inhibited *in vitro* proliferation of PD-1$^+$ Th cells in a dose-dependent manner, but this was only effective on cells from young mice, while similar treatment of Th cells from male BXSB.Yaa mice with established disease enhanced PD-1$^+$ Th cell division (Appendix Fig S3A). As these data indicated that the inhibitory functions of PD-1 were impaired in mice with established disease, we set out to treat the disease in male BXSB.Yaa mice by targeting pathogenic Th cells via their expression of NRP1.

NRP1 is a functional receptor that offers a route to enter cells; for example, interactions between SARS-CoV2 spike proteins with NRP1 have been reported to directly allow the SARS-CoV2 virus to penetrate the cell membrane leading to infection (Cantuti-Castelvetri *et al*, 2020; Daly *et al*, 2020). This route has been exploited artificially to deliver a drug payload to NRP1-expressing cancer cells by attachment of an iRGD peptide to NRP1 and so initiate tumor cell killing (Teesalu *et al*, 2009; Kim *et al*, 2016). These works inspired us to design a NRP1-targeting RPA peptide combined with proapoptotic peptide moiety (Sugahara *et al*, 2010). When this peptide was administered before disease onset, male BXSB.Yaa mice that received RPA treatment (RPA) were protected from splenomegaly (Fig 7A) and lymphadenopathy (Appendix Fig S3B), with a reduced serum secretion of IgG and ANA compared with male BXSB. Yaa mice receiving a control peptide (Control; Fig 7B). RPA treatment reduced NRP1$^+$PD$^-$1$^+$ Th cell expansion (Fig 7C and D) and Tph and Tfh cells were both reduced by RPA peptide treatment (Appendix Fig S3C), while Foxp3$^+$ Treg cells were unaffected by treatment targeting NRP1, including those that express NRP1 (Appendix Fig S3D), implying that different NRP1$^+$-expressing subsets have differential susceptibility to the intervention. To test this possibility, we sorted populations of NRP1$^+$ Th from spleens of diseased male BXSB.Yaa mice, using our Foxp3 reporter to divide them into Foxp3$^+$ Treg cells

 © 2022 The Authors

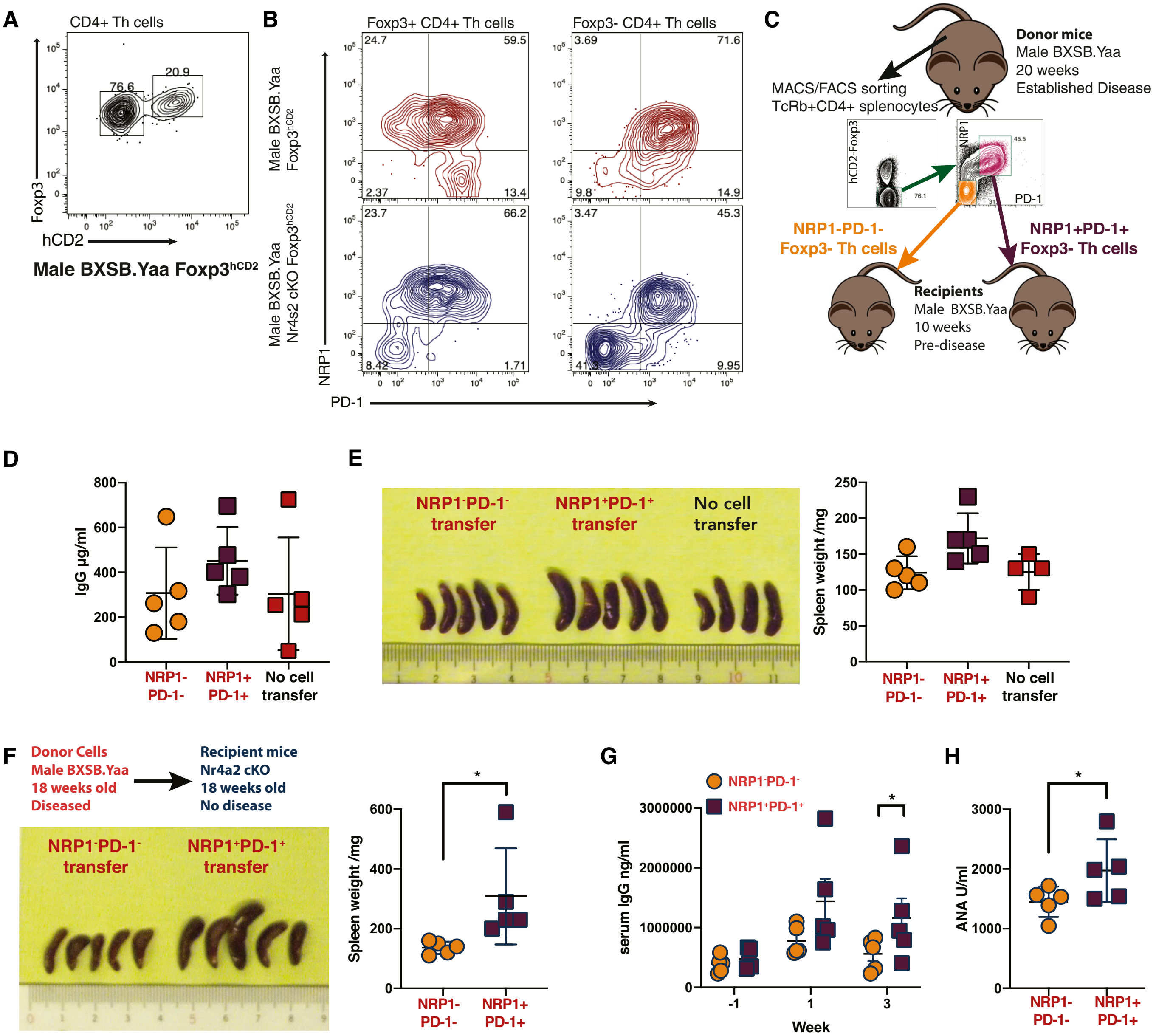

Figure 6. NRP1/PD-1-expressing Th cells are pathogenic.

A, B Spleens from male Foxp3^{hCD2} x BXSB.Yaa mice aged 20 weeks were stained for human CD2 as a Foxp3 surface reporter and intracellularly for Foxp3 among TcRβ$^+$CD4$^+$ T cells (A) and NRP1/PD-1 among hCD2$^+$ (Foxp3$^+$) and hCD2$^-$(Foxp3$^-$) TcRβ$^+$CD4$^+$ T cells (B). Data are representative of three independent experiments.

C–E As indicated in (C) splenic NRP1$^+$PD-1$^+$ and NRP1$^-$PD-1$^-$ Foxp3$^-$CD4$^+$ conventional T cells sorted from 20-week-old male Foxp3^{hCD2} BXSB.Yaa mice and 250,000 cells were transferred into 10-week-old male BXSB.Yaa recipient mice. Four weeks after transfer, serum IgG levels were compared with littermate mice receiving no transfer (D), and spleen sizes were measured (E); $n = 4$ mice per group; error bars, mean with SD; representative of three independent experiments.

F–H A total of 250,000 NRP1$^+$PD-1$^+$ or NRP1$^-$PD-1$^-$ Foxp3$^-$CD4$^+$ T cells were transferred into 18-week-old male BXSB.Yaa *Nr4a2* cKO recipient mice. Four weeks after transfer, spleen sizes were measured (F). Serum IgG (G) and ANA (H) concentrations were assessed 1 week before transfer and 1 and 3 weeks after transfer. $n = 4$ mice per group; error bars, mean with SD; representative of five experiments, (F, H) *$P < 0.05$ unpaired two-tailed Student's *t*-test, (G) *$P < 0.05$ group factor two-way ANOVA analysis with Bonferroni's multiple-comparison test.

and Foxp3$^-$ conventional Th cells and measured apoptosis induced by *in vitro* treatment with RPA peptide. We found that RPA peptide treatment of NRP1$^+$ Th cells led to reduced cells, but NRP1$^-$ cells were unaffected (Appendix Fig S3E). Cell death was greater in Foxp3$^-$NRP1$^+$ Th cells at mid-to-high RPA concentration over Foxp3$^+$ NRP1 Th cells. Taken together with our *in vivo* data, these findings suggest that Treg cells may be more resistant to apoptosis induction via the NRP1-targeting suicide peptide RPA.

In addition to prophylactic treatment, treatment after the disease established was still effective at reducing the key indicators of

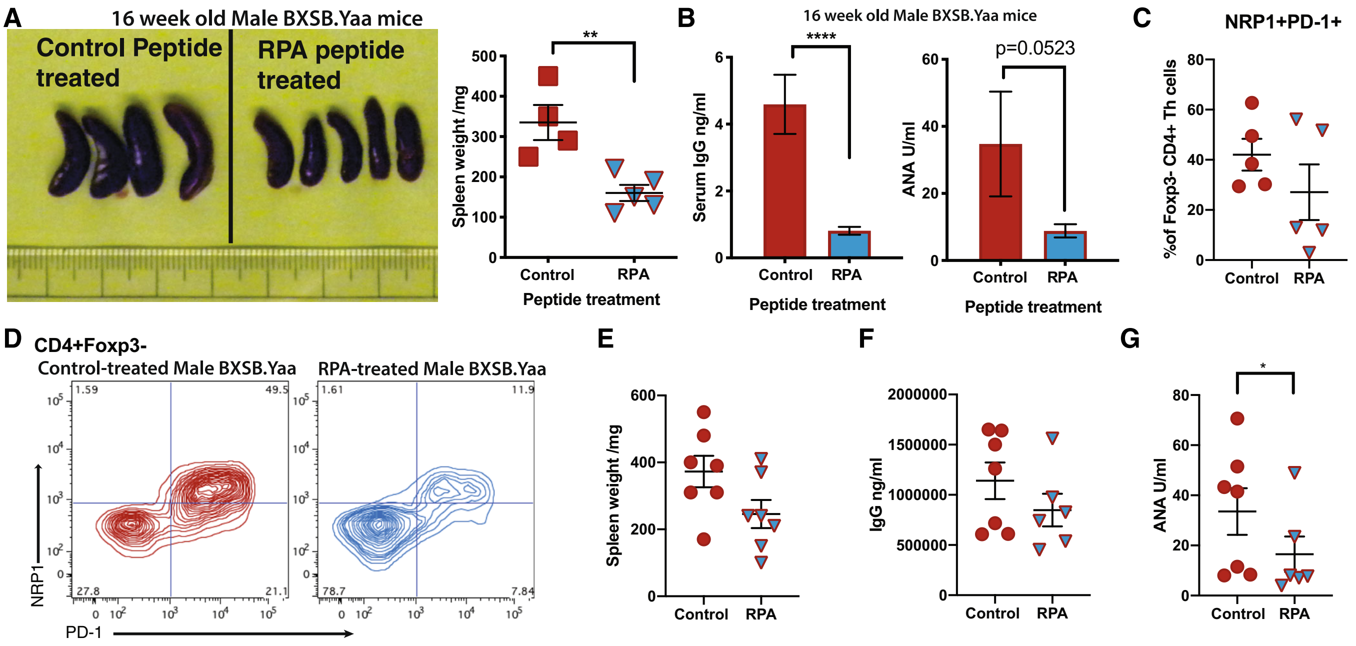

Figure 7. Therapeutic intervention targeting NRP1 ameliorates systemic autoimmunity.

A–D Male BXSB.Yaa mice were treated i.p. with 200 μM of control peptide (Control) or RPA peptide (RPA) in PBS at 10, 12, and 14 weeks of age. At 16 weeks, spleens were excised, photographed, and weighed (A), $^{**}P < 0.01$ unpaired two-tailed Student's *t*-test and total IgG and ANA concentrations were measured in serum (B), $^{***}P < 0.001$, two-tailed Mann–Whitney U test. $Foxp3^{-}$ splenic Th cells were assessed by flow cytometry for NRP1/PD-1 sub-populations, summary data (C) and representative staining (D); $n = 4$ mice per group; error bars, mean with SEM; representative of seven similar independent experiments.

E–G For treatment of established disease, male BXSB.Yaa were injected i.p. with 500 μM control peptide (Control) or RPA peptide (RPA) in PBS at 14, 16, and 18 weeks of age, and mice were sacrificed at 19 weeks old. Spleens were weighed (E) and serum assessed for total IgG (F) and ANA concentration (G), $^{*}P < 0.05$ two-tailed Mann–Whitney U test. $n = 4$ mice per group; error bars, mean with SEM; representative of four independent experiments.

disease (Fig 7E–G), although the reduction in pathogenic T cell subsets was modest (Appendix Fig S3F).

To further test the efficacy of RPA peptide in treating systemic autoimmunity, we also examined the efficacy of NRP1-targeted treatment in the MRL.LPR mouse model of systemic autoimmunity. Mutation in CD95 gene Fas^{lpr} generates spontaneous systemic autoimmune responses leading to massive lymphadenopathy, loss of body weight, glomerulonephritis, and the induction of anti-dsDNA antibodies (Andrews *et al*, 1978; Cohen & Eisenberg, 1991). MRL.LPR mice treated with RPA peptide showed increased body weight and reduced splenomegaly versus control-treated MRL.LPR mice (Appendix Fig S3G and H). Lower levels of serum antibodies including anti-dsDNA autoantibodies (Appendix Fig S3I) also resulted from RPA treatment and splenic $NRP1^{+}PD\text{-}1^{+}$ conventional Th cells were reduced (Appendix Fig S3J) along with the reduction in disease indicators, suggesting a link between this subset and pathogenic mechanisms also in the MRL.LPR mice.

NRP1-expressing Th cells are upregulated in human SLE patients

Our data from mouse models highlight a pivotal role for $NRP1^{+}PD\text{-}1^{+}$ non-Treg Th cells in systemic autoimmune disease. As targeting NRP1 may provide a new direction for the treatment of systemic autoimmune disease, we tested the relevance of these cells in human SLE disease.

Unlike in mice, human regulatory T cells marked by CD127 downregulation or Foxp3 expression do not express high levels of NRP1 (Fig 8A). This finding is in accordance with a previous study (Milpied *et al*, 2009).

NRP1 levels in circulating Th cells from a cohort of 10 patients with active SLE disease (Appendix Table S2) were assessed and compared with a population of 18 healthy controls (HC). We observed a significant increase in $NRP1^{+}$ Th cells in SLE (Fig 8B and C) and this difference was maintained when only conventional non-Treg cells were considered (Fig 8D). To account for the uneven gender distribution in SLE, we compared $NRP1^{+}$ Th cell data between female cases and female controls and found the significant difference remained extant (Appendix Fig S4A). Furthermore, the difference was not related to age or disease duration (Appendix Fig S4B and C) and patients in the SLE cohort were selected to have some level of active disease, regardless of treatment and there was no link between $NRP1^{+}$ Th cell level and the level of disease activity (Appendix Fig S4D). While $NRP1^{+}$ Th cell level was somewhat higher in the untreated SLE cohort as compared with the subjects under treatment at the time of sampling, this difference was not significant (Appendix Fig S4E).

As the level of $NRP1^{+}$ Th cells in the HC cohort was normally distributed (D'Agostino & Pearson normality test alpha = 0.05), we applied a cut-off value of 1.346% (calculated from HC population mean plus 3 standard deviations covering 99.7% of all predicted values in this population) to discern individuals that fall outside the

© 2022 The Authors

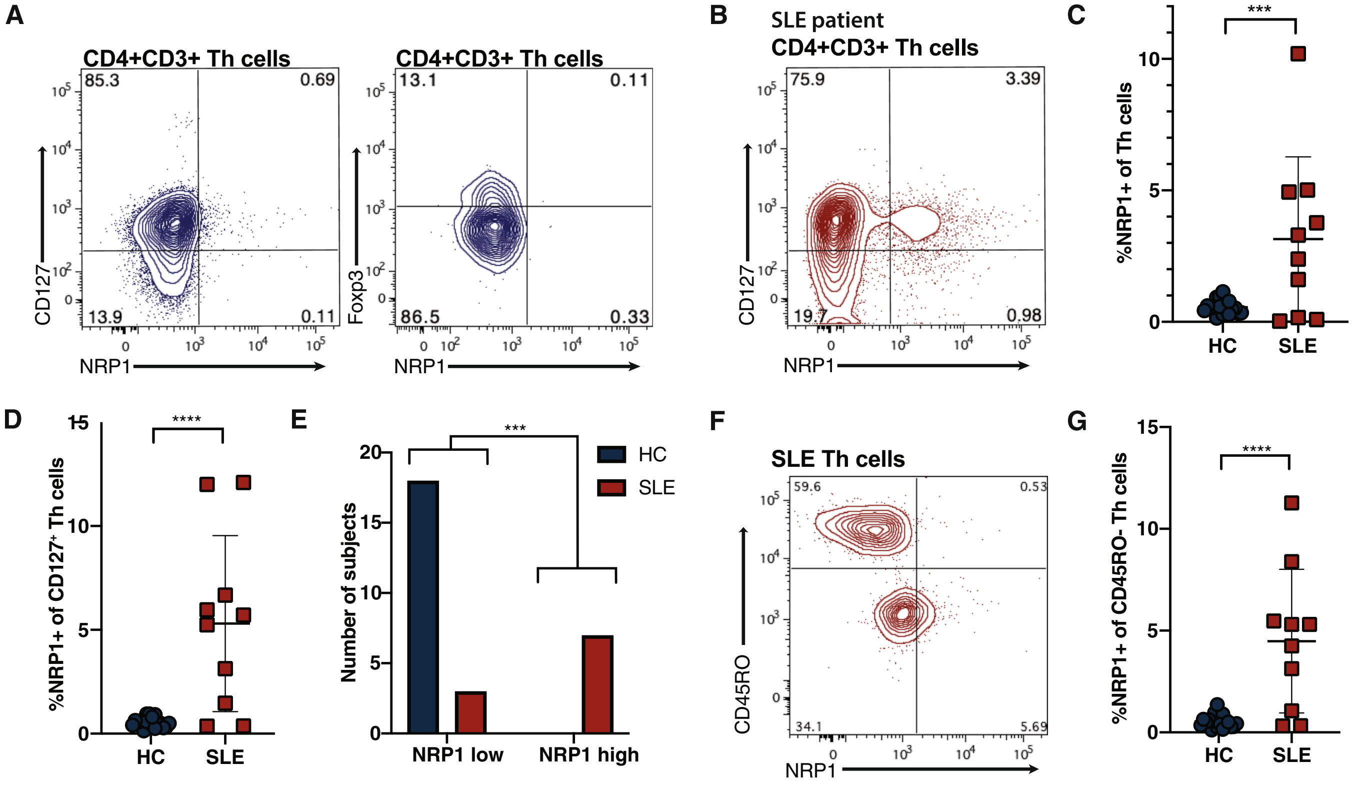

Figure 8. NRP1-expressing Th cells are upregulated in human SLE patients.

A PBMC were isolated from healthy volunteers and stained for flow cytometry to identify NRP1 expression in $CD3^+CD4^+$ Th cells against CD127 (left) and intracellular Foxp3 (right).

B–D NRP1-expressing cells among $CD4^+CD3^+$ Th cells were measured by flow cytometry in PBMC from 18 healthy volunteers (healthy controls, HC) and 10 SLE patients. Representative staining is shown for $CD4^+CD3^+$ Th cells (B), and summary data for $CD3^+CD4^+$ Th cells (C) and $CD3^+CD4^+CD127^{hi}$ conventional cells (D). Plots show individual subjects, HC, $n = 18$; SLE, $n = 10$; with mean ± SD indicated by error bars; ***$P < 0.001$, ****$P < 0.0001$ unpaired Student's *t*-test.

E Individuals were divided into NRP1-high subjects ($NRP1^+$ cells > 1.3% in Th cells) and NRP1-low subjects ($NRP1^+$ cells ≤ 1.35% in Th cells). Numbers of HC and SLE falling into each group are shown. ***$P < 0.001$ Chi-squared test with Yates' correction.

F, G NRP1-expressing cells among $CD3^+CD4^+CD45RO^-$ cells were measured, representative staining (F), and summary data (G). The plot shows individual subjects, HC, $n = 18$; SLE, $n = 10$; with mean ± SD indicated by error bars; ****$P < 0.0001$ unpaired Student's *t*-test.

HC population. At this cut-off level, 7 of 10 SLE patients were defined as NRP1 high with all HC as NRP1 low (Fig 8E); giving this cut-off a positive predictor value (PPV) of 0.8421 and a false discovery rate (FDR) of 0.

We further investigated the phenotype of $NRP1^+$ Th cells in SLE, and observed that the majority were CD45RO- (Fig 8F). Indeed, the increase in $NRP1^+$ Th cells among SLE patients was even more apparent when only CD45RO- Th cells were considered (Fig 8G).

The finding that $NRP1^+$ Th cells were increased in SLE patients confirm the utility of our mouse model experiments. This preliminary human study not only indicates that similar pathogenic Th cell mechanisms are likely in play but also that targeting these cells via NRP1 could form a promising treatment, particularly as the lack of NRP1 on human Tregs limits potential side effects.

Discussion

In this study, we demonstrated that T-cell-specific deletion of the *Nr4a2* gene prevents the induction of autoantibodies and SLE-like systemic disease in male BXSB.Yaa mice. In contrast, antibody production against exogenous antigens was unaltered in the absence of *Nr4a2*, suggesting that NR4A2 expression in Th cells may be an intrinsic requirement for immune responses against self. Furthermore, the NR4A2-dependent Th cell subsets expanded in disease and expressed a surface phenotype reminiscent of self-reactive Treg cells which were pathogenic on transfer, and targeting these cells abrogated disease.

Th cells have a critical role in SLE, driving the development of pathogenic B cells producing autoantibodies and subsequent pathology (Craft, 2012; Tellier & Nutt, 2013; Crotty, 2014; Ueno, 2016) and treatments targeting T cells can reduce SLE-like disease (Chu *et al*, 1996; Yin *et al*, 2015). However, immune targeting approaches without the ability to discriminate self- from non-self-responses carry a potential risk of side effects due to global immune deficits. Therefore, our new findings that *Nr4a2* deficiency compromises autoreactive T cell responses in systemic autoimmunity, while responses against foreign antigens remain intact, yield a tantalizing prospect for the specific identification of self-reactive T cells.

© 2022 The Authors

NR4A2 and other NR4A family members have been previously linked to circulating autoreactive T cells in type 1 diabetes (Durinovic-Bello *et al*, 2014). Furthermore, an intrinsic association between NR4A family members and the quality of T cell activation response has been highlighted: *Nr4a1* reporter mice are widely used to estimate antigenic stimulation in thymocytes (Moran *et al*, 2011; Stadinski *et al*, 2016), and TcR avidity in Treg cells is controlled by the presence/absence of *Nr4a2* (Sekiya *et al*, 2013). Therefore, NR4A family members may be crucially required for high-affinity T cell responses to self-antigens, irrelevant of their effector or regulatory functional properties. It is this apparent NR4A-dependent ability to mount high avidity responses to stimulation by low self-antigen levels or with lower-affinity self-reactive TcRs that may be shared between pathogenic autoreactive subsets and Treg cells (Hirota *et al*, 2007; Martin *et al*, 2013).

While Treg cells can have high affinity for self-antigen, earlier studies suggested that negative selection in the thymus cells permits the release of conventional T cells only with TcRs that have low affinity for self-antigen (Lee *et al*, 2012). As Treg cells can downregulate Foxp3 and become effector cells under certain conditions (Komatsu *et al*, 2014), it is possible that NR4A2-dependent pathogenic Th cells with high-affinity TcR for self-antigens may be converted from common Treg precursor in the peripheral pool or switch directly from Treg cells during autoimmune disease development.

We show that NRP1 expression refines the identification of NR4A2-dependent pathogenic Th cells in diseased male BXSB.Yaa mice and, furthermore, self-reactive NRP1$^+$ Th1/Th17 cells are induced in CNS autoimmune disease and trafficking into the target organ. Indeed, it has been previously reported that NRP1 deficiency reduces EAE symptoms, but the mechanism remains unknown (Solomon *et al*, 2011). The unusual NRP1$^+$ conventional Th cells sharing a surface phenotype with Treg have not been previously reported in systemic autoimmune disease or target organ autoimmunity. Similar NRP1$^+$ Th cells also expand in autoimmune-prone MRL.LPR mice and *Aire*$^{-/-}$ mice, but not in older C57BL/6 mice. Slight increases in NRP1$^+$ conventional Th cells are also observed in MRL mice by 20 weeks that may occur at the same time as early autoantibody increase seen even without the LPR mutation, although full autoimmunity does not become established in this strain until 1–2 years of age (Izui *et al*, 1984). Transfer of conventional NRP1$^+$ Th cells enhances autoimmune disease and NRP1-targeting treatment (RPA peptide) significantly ameliorated autoimmune indicators, thus not only are NRP1-expressing conventional Th cells pathogenic and sufficient to induce disease but also are required for the full establishment of disease.

A number of studies highlighted NRP1 expression on subsets of Treg cells (Delgoffe *et al*, 2013; Overacre-Delgoffe *et al*, 2017), whereas reports indicated NRP1 expression only on a small population of non-Treg conventional cells (Milpied *et al*, 2009; Solomon *et al*, 2011). It was suggested that NRP1 expression was confined to natural Treg cells and thus could be used to distinguish them from induced Treg (Yadav *et al*, 2012), although later studies conflict with this finding (Singh *et al*, 2015). Our data connecting NRP1 with pathogenic Th cells are interesting when considering that both autoreactive T cells and many Treg cells are specific for self-peptides and NRP1 may constitute part of a phenotype associated with self-specific T cells. It is possible that NRP1$^+$PD-1$^+$ pathogenic Th cells develop from Treg cells switching to an autoreactive phenotype or from *de novo* NRP1 expression of lower avidity self-reactive peripheral Th cells. Further investigation into the ontogeny of these cells is required to understand this process.

Tfh and Tph cells have been previously implicated in driving B cell activation in systemic autoimmune disease, but these populations are characterized by markers shared with normal Th cells. Our data indicate that Th cells driving SLE-like disease may be spread between Tph and Tfh cells and instead depends on NR4A2. Thus, T-cell-specific deletion of *Nr4a2* in male BXSB.Yaa mice enabled us to capture self-reactive T cell subsets as a whole, resulting in the identification of NRP1 on conventional Th cells as a potential biomarker for specifying *bona fide* self-reactive Th cells. However, without knowledge of all the self-antigens involved in SLE, it is possible that NRP1$^+$ pathogenic cells may only represent a subset of self-reactive Th cells in SLE-related diseases. A further caveat to our works is that NRP1 expression on human cells appears more controversial. An early study demonstrated a link between NRP1 and Treg cells in humans (Battaglia *et al*, 2008), but this was disputed by work showing that human Treg cells do not express NRP1 (Milpied *et al*, 2009). Therefore, further NRP1 studies in human disease are highly desirable. Furthermore, NRP1 is a complex receptor with a range of possible functions and a number of different binding domains (Pellet-Many *et al*, 2008). It interacts immunologically relevant molecules and influences immune responses in a number of potential ways: semaphorins/plexin (Gu *et al*, 2002; Lu *et al*, 2021) and VEGF (Hansen *et al*, 2012) are ligands for different NRP1 domains, and NRP1 has been linked to T cell–dendritic cell interactions (Mizui & Kikutani, 2008) and cytokine signaling (Glinka *et al*, 2011). Thus, the mechanism of action for NRP1 in pathogenic Th cells driving autoreactive B cell responses remains to be understood.

The key challenge in controlling autoreactive responses is to disrupt Th cells responding inappropriately to self-peptide while leaving beneficial T cell responses intact. However, autoreactive T cell targeting therapy has been limited by the ability to identify and target only self-reactive T cells, while maintaining essential immune responses. Currently, treatments rely on blocking whole types of immune response/cells or through detailed knowledge of autoantigen(s) to induce tolerance. Most animal research into autoimmune disease focuses on models using defined antigens often with peptides/proteins at non-physiologic levels or utilizing TcR transgenic mice with an unrealistic amount of self-specific T cells. Thus, these models may not be suitable for a comprehensive understanding of key hallmarks of autoreactive cells in complex autoimmune diseases such as SLE. Our study approaches the question from a different direction, examining molecules that may be shared by Th cells generally that respond to self-antigens, NR4A2 and NRP1. NRP1$^+$ Th cells are associated with self-reactive responses in autoimmune neuroinflammation and are pathogenic in SLE-like disease, with increased NRP1$^+$ non-Treg Th cells in human SLE patients, and NRP1 can be targeted therapeutically to reduce disease. These findings hint at a wide application of NRP1 to detect and target self-reactive Th cells in a range of autoimmune diseases: however, further study is needed to see how universal this marker is in other diseases and if these cells are the main pathogenic component. These findings potentially open a new approach to treat a wide variety of autoimmune diseases.

© 2022 The Authors

Materials and Methods

Study design

The aim of this study was to explore the role of NR4A2 in T cell activity in systemic autoimmune diseases; as NR4A2 was previously shown to affect IL-21 in a target organ-mediated autoimmune disease, this study focused on an IL-21-dependent standard mouse model of SLE. Mice lacking *Nr4a2* in T cells were generated on a background prone to a spontaneous SLE-like disease, BXSB.Yaa. Breeding was established using a heterozygous system to generate age-matched littermate groups that either lacked *Nr4a2* in T cells or had intact *Nr4a2* as controls. As the disease develops in male BXSB.Yaa mice, using female mice allowed matched controls with no disease. Preliminary experiments conducted during the back-crossing process confirmed key timepoint for disease induction in this well-described disease model and required experimental group size. For treatment and transfer experiments, control or experiment groups were assigned randomly; where possible researchers were blinded to the treatment group, but blinding was not possible for mouse groups due to institutional requirements for labeling LMO animals. The number of experimental replicates and group sizes is indicated in the individual figure legends.

Human subjects

The Ethics Committee of the National Center of Neurology and Psychiatry (NCNP) on human experimentation (A2019-061) and the Kitasato University School of Medicine and Hospital (B19-223) approved the study according to Declaration of Helsinki guidelines and Department of Health and Human Services Belmont Report. Written informed consent was obtained from all participants. The subjects donated blood samples for flow cytometric measurement and cohorts are listed in Appendix Table S2. SLE patients were recruited at Kitasato University Hospital, and diagnosis was confirmed according to the 1997 American College of Rheumatology revised classification criteria for SLE (Hochberg, 1997).

Mice

All mice used were maintained in specific pathogen-free conditions in accordance with institutional guidelines and used at the ages indicated. This study was approved by the Committee for Small Animal Research and Animal Welfare (National Center of Neurology and Psychiatry). All efforts were made to minimize animal suffering in clinical disease experiments where 5–10 mice were used for scoring in each group. BXSB.Yaa were purchased from Japan SLC, Inc. (Shizuoka, Japan) and were backcrossed 10 generations with C57BL/6 CD4-Cre/$Nr4a2^{fl/fl}$ (*Nr4a2* cKO) mice (Raveney *et al*, 2015). $Foxp3^{hCD2}$ mice of C57BL/6 background (Komatsu *et al*, 2009) were backcrossed 10 generations with BXSB-Yaa CD4-Cre/$Nr4a2^{fl/fl}$ (Nr4a2 cKO) mice. $Aire^{tm1/1Doi/J}$ (*Aire* KO) mice on a C57BL/6 background were purchased from Jackson Laboratory (Bar Harbor, ME, USA) and female MRL and MRL.LPR mice were purchased from Japan SLC, Inc. (Shizuoka, Japan).

For serum antibody measurements, blood was withdrawn by retro-orbital bleed, cells were removed by centrifugation, and serum was harvested and stored at –80°C until assayed.

For anti-NP antibody induction in *Nr4a2* cKO mice or control mice with intact *Nr4a2* on BXSB.Yaa or C57BL/6 background, mice were injected subcutaneously with 100 μg NP-KLH (Biosearch Technologies, CA, USA) emulsified in complete Freund's adjuvant (Difco, KS, USA).

For treatment, NRP1-targeting peptide (Sequence RPARPAR) or control peptide (sequence GAGAGAG) conjugated to a cell death-inducing C-GG-$_D$(KLAKLAK)2 domain (synthesized by Toray, Kanagawa, Japan) was administered, either 200 μM or 500 μM was given per mouse in 200 μl PBS.

EAE induction was carried out using our standard protocol (Raveney *et al*, 2015). Briefly, mice were injected subcutaneously with 100 μg $MOG_{35–55}$ peptide (synthesized by Toray Research Center, Chūō-ku, Tokyo, Japan) and 1 mg heat-killed mycobacterium tuberculosis H37RA emulsified in complete Freund's adjuvant (Difco, KS, USA). One hundred nanogram Pertussis toxin (List Biological Laboratories, CA, USA) was injected intraperitoneally (i.p.) on days 0 and 2 after immunization. All peptides were synthesized by Toray Research Center (Chūō-ku, Tokyo, Japan).

Cell isolation, staining, and purification

Spleens were excised after mice were humanely euthanized, connective tissues were removed, and organs dried with an absorbent towel before weighing and photography. Single-cell suspensions were then generated by mechanical disruption of tissues through 70 mm cell strainers (BD Biosciences) and red blood cells were lysed with ACK lysing solution as previously described (Raveney *et al*, 2015). CNS-infiltrating lymphocytes were isolated from spinal cords and brains as previously described (Klemann *et al*, 2009). Briefly, the tissue was cut into small pieces and digested for 40 min at 37°C in RPMI 1640 media (Invitrogen, Tokyo, Japan) supplemented with 1.4 mg/ml collagenase H and 100 μg/ml DNase I (Roche Diagnostics, Tokyo, Japan). The resulting tissue homogenates were forced through a 70 μm cell strainer and leukocytes were enriched using a discontinuous 37%/80% Percoll density gradient centrifugation (GE Healthcare Life Sciences, Tokyo, Japan).

Assessment of cell function

For stimulation experiments, culture media used were DMEM supplemented with 10% FCS, 2 mM L-glutamine, 100 U/ml of penicillin–streptomycin, and 50 μM 2-mercaptoethanol (all Invitrogen). Serum antibody levels were measured using Mouse IgG1, IgG2a, and IgG2b Ready-set-Go! ELISA kits (eBioscience, San Diego, CA, USA) according to manufacturer's instructions or standard ELISA using NP-CGG (with conjugation ratio of either 4 or 31, Biosearch Technologies, CA, USA) and secondary antibodies. Fecal IgA antibodies were measured by ELISA (Affymetrix) according to the manufacturer's instructions. Anti-nuclear antibodies were measured using ANA ELISA kits (USBiological, MA, USA) according to the manufacturer's instructions. Mouse IL-17A and IFN-γ from supernatants were measured using paired antibody kits according to manufacturers' instructions and were supplied by R&D Systems (Minneapolis, USA) and BD Biosciences, respectively.

For intracellular staining, cells were restimulated with 5 ng/ml PMA/ PMA, 500 ng/ml ionomycin (both Sigma-Aldrich) in the presence of Golgi Stop (BD Biosciences) for 5 h, before surface staining

© 2022 The Authors

and fixing/intracellular staining using an eBioscience Foxp3 staining kit according to manufacturer's instructions. For IL-21 measurement, an IL-21-receptor IgG fusion protein (mouse IL-21R Fc Chimera, R&D systems, MN, USA) was used after fixing cells with an eBioscience Foxp3 staining kit. Cells were then stained for surface antigens. Flow cytometry data were acquired using a FACS Canto II or FACS ARIA II (BD cytometry systems) with a FACS DIVA software. Antibodies for flow cytometry were sourced from BioLegend and are listed in Appendix Table S3.

Real-time qPCR

For quantification of mRNA transcripts, total RNA was extracted from flow cytometrically sorted spleen cell populations using a RNeasy Mini Kit (Qiagen, Tokyo, Japan) according to the manufacturer's instructions and cDNA was then generated using a first-strand cDNA Kit (Takara, Shiga, Japan). Quantified real-time PCR using a Power SYBR Green master mix (Applied Biosystems, Warrington, UK) with a LightCycler96 instrument (Roche Diagnostics) was performed using commercial primers (Quantitech primers, Qiagen), and gene expression values were normalized to the expression of the GAPDH, HPRT1, or b2M housekeeping genes.

Histology

Kidney sections obtained from 24-week-old *Nr4a2*(fl/fl) or CD4-Cre/*Nr4a2*(fl/fl) mice at necropsy were fixed with formalin, paraffin embedded, and stained with hematoxylin & eosin, periodic acid/Schiff reagent (PAS), or Masson's trichrome. At least 10 glomeruli were examined for each animal to obtain representative pictures.

Fluorescence microscopy

Spleens were frozen in OCT at −80°C before sectioning in a Microm-HM-525 cryostat. Tissue sections were fixed in acetone for 5 min and blocked with 3% BSA/PBS. Sections were stained overnight with conjugated antibodies: FITC-conjugated anti-B220 or anti-GL7, and biotinylated anti-CD4 (all Biolegend) with a secondary stain of AlexaFluor-594-conjugated streptavidin (Thermo Life Technologies, MA, USA). Sections were mounted with Fluoromount (Southern Biotech, AL, USA) and imaged with a fluorescence BZ-X850 microscope with a BZ-X software (Keyence, Osaka, Japan).

Immunoblotting

Various tissues were dissected from 20-week-old BXSB-Yaa mice and tissue proteins were extracted by using Gentle Macs (Miltenyi Biotech K.K., Tokyo, Japan) according to the manufacturer's instructions.

Unbiased amplification of TCR genes, sequencing, and data analysis

Next-generation sequencing analysis was performed with an unbiased TCR repertoire analysis technology (Repertoire Genesis Inc., Osaka, Japan). Unbiased adaptor-ligation PCR was performed according to the previous reports (Yoshida *et al*, 2000). After PCR amplification, index (barcode) sequences were added by amplification with Nextera XT index kit v2 setA (illumina, San Diego, CA). The indexed amplicon products were mixed in an equal molar concentration and quantified by a Qubit 2.0 Fluorometer (Thermo Fisher Scientific, Waltham, MA). Sequencing was carried out with the Illumina Miseq paired-end platform (2 × 300 bp). All the paired-end reads were classified by index sequences. Assignment of sequences was performed by determining sequences with the highest identity in a data set of reference sequences from the international ImMunoGeneTics information system® (IMGT) database (http://www.imgt.org). Data processing, assignment, and data aggregation were automatically performed using a repertoire analysis software originally developed by Repertoire Genesis Inc. Nucleotide sequences of CDR3 regions ranged from conserved cysteine at position 104 (Cys104) of IMGT nomenclature to conserved phenylalanine at position 118 (Phe118) and the following glycine (Gly119) was translated to deduce amino acid sequences. A unique sequence read (USR) was defined as a sequence read having no identity in TRV, TRJ, and deduced amino acid sequence of CDR3 with the other sequence reads. The copy number of identical USR was automatically counted by RG software (Repertoire Genesis Inc, Japan) in each sample and then ranked in order of the copy number.

Statistical analysis

Data were analyzed using Prism software (GraphPad, CA, USA) and presented as individual points with mean values ± SD. Normality testing was carried out using a Shapiro–Wilk test with a 5% alpha considered normal. Data sets were considered to have an equal variance when standard deviations were within a ratio of 2 and then confirmed with an F test. For bivariate hypothesis testing, a non-parametric Mann–Whitney U test for comparing two sets of non-Gaussian data and a Student's t-test was used for normally distributed data, with Welch's correction when variances between data sets were unequal. For more than two data sets, a one-way ANOVA was used for data with a Gaussian distribution with Bonferroni's *post hoc* test for multiple comparisons; when data set did not have equal variances, a Brown–Forsythe one-way ANOVA test with Dunnett's T3 multiple comparisons test was substituted. For analysis of two independent variables, two-way ANOVA Bonferroni's *post hoc* test for multiple comparisons. P-values and n numbers are stated in the figure legends.

Data availability

The raw TcR sequencing data from this publication (Fig 4) have been deposited to the DNA DataBank of Japan (DDBJ) database and registered with BioProject as BioProject ID PRJNA856453; BioSample IDs: SAMN29553878 and SAMN29553879; data available from SRA http://www.ncbi.nlm.nih.gov/bioproject/856453. Aligned TcR vβ/jβ chains with CDR3β sequences are available in the extended data (Dataset Fig 4) with read counts for each sample.

Expanded View for this article is available online.

© 2022 The Authors

Acknowledgments

The authors would like to thank A. Kimura, C. Tomi, K. Inoue, A. Takeo, and C. Koto for excellent technical assistance and animal care. This work was supported by Health and Labour Sciences Research Grants, Research on rare and intractable diseases, JSPS KAKENHI (number 24590108); the Practical Research Project for Rare/Intractable Diseases from Japan Agency for Medical Research and Development, AMED (20ek0109314) and AMED-CREST (22gm1010011); and Intramural Research Grants (30-9 and 1-5) for Neurological and Psychiatric Disorders of NCNP. BJER is a recipient of JSPS KAKENHI grants (numbers 17K16135 & 20K07775).

Author contributions

Ben JE Raveney: Conceptualization; data curation; formal analysis; investigation; methodology; writing – original draft; writing – review and editing. **Yosif El-Darawish:** Data curation; formal analysis; investigation. **Wakiro Sato:** Resources; data curation; project administration. **Yoshiyuki Arinuma:** Resources; formal analysis. **Kunihiro Yamaoka:** Resources; formal analysis. **Shohei Hori:** Resources. **Takashi Yamamura:** Conceptualization; supervision; funding acquisition; writing – review and editing. **Shinji Oki:** Formal analysis; supervision; writing – original draft; project administration; writing – review and editing.

In addition to the CRediT author contributions listed above, the contributions in detail are:

BR and YMED performed experiments; BR and SO designed experiments; BR, TY, and SO analyzed data; WS, YA, and KY provided human samples and analyzed data; SH provided C57BL/6 Foxp3^{hCD2} mice; BR, TY, and SO wrote the manuscript; and SO conceived the study and supervised the project.

Disclosure and competing interests statement

The authors declare that they have no conflict of interest.

For more information

i OMIM: https://omim.org/
ii GenBank: https://www.ncbi.nlm.nih.gov/gene/
iii Lupus Foundation of America: https://www.lupus.org
iv National Center for Neurology and Psychiatry: https://www.ncnp.go.jp/en/

The paper explained

Problem

Autoimmune disease results from self-attack by one's own immune system. In a systemic autoimmune disease such as Lupus, B cells make antibodies that target one's own tissues and organs and cause chronic damage. These responses are controlled by T helper cells that have developed to be specific for self rather than making protective responses against invading pathogens. As these dangerous cells hijack normally beneficial processes and look like helpful T cells, they are hard to destroy without harming important mechanisms that defend human health.

Results

Using a mutant mouse strain that develops a similar autoimmune disease to SLE, we investigated the properties of these self-reactive T cells. We blocked a gene that was previously associated with dangerous responses and found the self-reactive T cells were decreased and the autoimmune symptoms were decreased but defense against foreign pathogens was unaffected. This allowed us to compare normal and self-reactive T cells directly and we found that they unusually expressed a protein called neuropilin-1 on their cell surface. By targeting this receptor to deliver a payload that kills cells, we could destroy these pathogenic T cells and treat disease. We also found there was an increase in T cells with this neuropilin-1 receptor in the blood of human patients with Lupus disease.

Impact

Testing blood for these neuropilin-1-expressing T cells could provide early information in patients to diagnose an autoimmune disease caused by self-reactive T cells. The ability to identify this type of cell from similar helpful T cells is key to being able to make specific drugs to treat patients. Current treatments rely on blocking the function of part of the immune system or generally reducing the immune system activity, which can reduce the effectiveness of defense against common infectious diseases. If self-reactive T cells causing autoimmune disease can be specifically targeted, it would allow a "silver bullet" therapy to effectively help sufferers whilst reducing potentially dangerous side effects.

References

Anderson MS, Venanzi ES, Klein L, Chen Z, Berzins SP, Turley SJ, von Boehmer H, Bronson R, Dierich A, Benoist C *et al* (2002) Projection of an immunological self shadow within the thymus by the aire protein. *Science* 298: 1395–1401

Andrews BS, Eisenberg RA, Theofilopoulos AN, Izui S, Wilson CB, McConahey PJ, Murphy ED, Roths JB, Dixon FJ (1978) Spontaneous murine lupus-like syndromes. Clinical and immunopathological manifestations in several strains. *J Exp Med* 148: 1198–1215

Arbuckle MR, McClain MT, Rubertone MV, Scofield RH, Dennis GJ, James JA, Harley JB (2003) Development of autoantibodies before the clinical onset of systemic lupus erythematosus. *N Engl J Med* 349: 1526–1533

Battaglia A, Buzzonetti A, Monego G, Peri L, Ferrandina G, Fanfani F, Scambia G, Fattorossi A (2008) Neuropilin-1 expression identifies a subset of regulatory T cells in human lymph nodes that is modulated by preoperative chemoradiation therapy in cervical cancer. *Immunology* 123: 129–138

Baudino L, Azeredo da Silveira S, Nakata M, Izui S (2006) Molecular and cellular basis for pathogenicity of autoantibodies: lessons from murine monoclonal autoantibodies. *Springer Semin Immunopathol* 28: 175–184

Bocharnikov AV, Keegan J, Wacleche VS, Cao Y, Fonseka CY, Wang G, Muise ES, Zhang KX, Arazi A, Keras G *et al* (2019) PD-1hiCXCR5- T peripheral helper cells promote B cell responses in lupus via MAF and IL-21. *JCI Insight* 4: e130062

Bruder D, Probst-Kepper M, Westendorf AM, Geffers R, Beissert S, Loser K, von Boehmer H, Buer J, Hansen W (2004) Neuropilin-1: a surface marker of regulatory T cells. *Eur J Immunol* 34: 623–630

Bubier JA, Bennett SM, Sproule TJ, Lyons BL, Olland S, Young DA, Roopenian DC (2007) Treatment of BXSB-Yaa mice with IL-21R-fc fusion protein minimally attenuates systemic lupus erythematosus. *Ann N Y Acad Sci* 1110: 590–601

Bubier JA, Sproule TJ, Foreman O, Spolski R, Shaffer DJ, Morse HC 3rd, Leonard WJ, Roopenian DC (2009) A critical role for IL-21 receptor signaling in the pathogenesis of systemic lupus erythematosus in BXSB-Yaa mice. *Proc Natl Acad Sci U S A* 106: 1518–1523

Cantuti-Castelvetri L, Ojha R, Pedro LD, Djannatian M, Franz J, Kuivanen S, van der Meer F, Kallio K, Kaya T, Anastasina M *et al* (2020) Neuropilin-1 facilitates SARS-CoV-2 cell entry and infectivity. *Science* 370: 856–860

Cao AT, Yao S, Gong B, Nurieva RI, Elson CO, Cong Y (2015) Interleukin (IL)-21 promotes intestinal IgA response to microbiota. *Mucosal Immunol* 8: 1072–1082

© 2022 The Authors

Chu EB, Hobbs MV, Wilson CB, Romball CG, Linsley PS, Weigle WO (1996) Intervention of CD4+ cell subset shifts and autoimmunity in the BXSB mouse by murine CTLA4Ig. *J Immunol* 156: 1262 – 1268

Cohen PL, Eisenberg RA (1991) Lpr and gld: single gene models of systemic autoimmunity and lymphoproliferative disease. *Annu Rev Immunol* 9: 243 – 269

Comte D, Karampetsou MP, Tsokos GC (2015) T cells as a therapeutic target in SLE. *Lupus* 24: 351 – 363

Craft JE (2012) Follicular helper T cells in immunity and systemic autoimmunity. *Nat Rev Rheumatol* 8: 337 – 347

Crispin JC, Kyttaris VC, Terhorst C, Tsokos GC (2010) T cells as therapeutic targets in SLE. *Nat Rev Rheumatol* 6: 317 – 325

Crotty S (2014) T follicular helper cell differentiation, function, and roles in disease. *Immunity* 41: 529 – 542

Crotty S (2019) T follicular helper cell biology: a decade of discovery and diseases. *Immunity* 50: 1132 – 1148

Daley SR, Koay HF, Dobbs K, Bosticardo M, Wirasinha RC, Pala F, Castagnoli R, Rowe JH, Ott de Bruin LM, Keles S *et al* (2019) Cysteine and hydrophobic residues in CDR3 serve as distinct T-cell self-reactivity indices. *J Allergy Clin Immunol* 144: 333 – 336

Daly JL, Simonetti B, Klein K, Chen KE, Williamson MK, Anton-Plagaro C, Shoemark DK, Simon-Gracia L, Bauer M, Hollandi R *et al* (2020) Neuropilin-1 is a host factor for SARS-CoV-2 infection. *Science* 370: 861 – 865

Delgoffe GM, Woo SR, Turnis ME, Gravano DM, Guy C, Overacre AE, Bettini ML, Vogel P, Finkelstein D, Bonnevier J *et al* (2013) Stability and function of regulatory T cells is maintained by a neuropilin-1-semaphorin-4a axis. *Nature* 501: 252 – 256

Doi Y, Oki S, Ozawa T, Hohjoh H, Miyake S, Yamamura T (2008) Orphan nuclear receptor NR4A2 expressed in T cells from multiple sclerosis mediates production of inflammatory cytokines. *Proc Natl Acad Sci U S A* 105: 8381 – 8386

Dong W, Zhu P, Wang Y, Wang Z (2011) Follicular helper T cells in systemic lupus erythematosus: a potential therapeutic target. *Autoimmun Rev* 10: 299 – 304

Durinovic-Bello I, Gersuk VH, Ni C, Wu R, Thorpe J, Jospe N, Sanda S, Greenbaum CJ, Nepom GT (2014) Avidity-dependent programming of autoreactive T cells in T1D. *PLoS One* 9: e98074

Francisco LM, Salinas VH, Brown KE, Vanguri VK, Freeman GJ, Kuchroo VK, Sharpe AH (2009) PD-L1 regulates the development, maintenance, and function of induced regulatory T cells. *J Exp Med* 206: 3015 – 3029

Freeman GJ, Long AJ, Iwai Y, Bourque K, Chernova T, Nishimura H, Fitz LJ, Malenkovich N, Okazaki T, Byrne MC *et al* (2000) Engagement of the PD-1 immunoinhibitory receptor by a novel B7 family member leads to negative regulation of lymphocyte activation. *J Exp Med* 192: 1027 – 1034

Glinka Y, Stoilova S, Mohammed N, Prud'homme GJ (2011) Neuropilin-1 exerts co-receptor function for TGF-beta-1 on the membrane of cancer cells and enhances responses to both latent and active TGF-beta. *Carcinogenesis* 32: 613 – 621

Gu C, Limberg BJ, Whitaker GB, Perman B, Leahy DJ, Rosenbaum JS, Ginty DD, Kolodkin AL (2002) Characterization of neuropilin-1 structural features that confer binding to semaphorin 3A and vascular endothelial growth factor 165. *J Biol Chem* 277: 18069 – 18076

Hansen W, Hutzler M, Abel S, Alter C, Stockmann C, Kliche S, Albert J, Sparwasser T, Sakaguchi S, Westendorf AM *et al* (2012) Neuropilin 1 deficiency on CD4+Foxp3+ regulatory T cells impairs mouse melanoma growth. *J Exp Med* 209: 2001 – 2016

Henderson JG, Opejin A, Jones A, Gross C, Hawiger D (2015) CD5 instructs extrathymic regulatory T cell development in response to self and tolerizing antigens. *Immunity* 42: 471 – 483

Hirota K, Hashimoto M, Yoshitomi H, Tanaka S, Nomura T, Yamaguchi T, Iwakura Y, Sakaguchi N, Sakaguchi S (2007) T cell self-reactivity forms a cytokine milieu for spontaneous development of IL-17+ Th cells that cause autoimmune arthritis. *J Exp Med* 204: 41 – 47

Hochberg MC (1997) Updating the American College of Rheumatology revised criteria for the classification of systemic lupus erythematosus. *Arthritis Rheum* 40: 1725

Hsieh CS, Zheng Y, Liang Y, Fontenot JD, Rudensky AY (2006) An intersection between the self-reactive regulatory and nonregulatory T cell receptor repertoires. *Nat Immunol* 7: 401 – 410

Izui S, Kelley VE, Masuda K, Yoshida H, Roths JB, Murphy ED (1984) Induction of various autoantibodies by mutant gene lpr in several strains of mice. *J Immunol* 133: 227 – 233

Izui S, Ibnou-Zekri N, Fossati-Jimack L, Iwamoto M (2000) Lessons from BXSB and related mouse models. *Int Rev Immunol* 19: 447 – 472

Jager A, Dardalhon V, Sobel RA, Bettelli E, Kuchroo VK (2009) Th1, Th17, and Th9 effector cells induce experimental autoimmune encephalomyelitis with different pathological phenotypes. *J Immunol* 183: 7169 – 7177

Kalekar LA, Schmiel SE, Nandiwada SL, Lam WY, Barsness LO, Zhang N, Stritesky GL, Malhotra D, Pauken KE, Linehan JL *et al* (2016) CD4(+) T cell anergy prevents autoimmunity and generates regulatory T cell precursors. *Nat Immunol* 17: 304 – 314

Kawamoto S, Tran TH, Maruya M, Suzuki K, Doi Y, Tsutsui Y, Kato LM, Fagarasan S (2012) The inhibitory receptor PD-1 regulates IgA selection and bacterial composition in the gut. *Science* 336: 485 – 489

Kim JY, Han JH, Park G, Seo YW, Yun CW, Lee BC, Bae J, Moon AR, Kim TH (2016) Necrosis-inducing peptide has the beneficial effect on killing tumor cells through neuropilin (NRP-1) targeting. *Oncotarget* 7: 32449 – 32461

Klemann C, Raveney BJ, Klemann AK, Ozawa T, von Horsten S, Shudo K, Oki S, Yamamura T (2009) Synthetic retinoid AM80 inhibits Th17 cells and ameliorates experimental autoimmune encephalomyelitis. *Am J Pathol* 174: 2234 – 2245

Komatsu N, Mariotti-Ferrandiz ME, Wang Y, Malissen B, Waldmann H, Hori S (2009) Heterogeneity of natural Foxp3+ T cells: a committed regulatory T-cell lineage and an uncommitted minor population retaining plasticity. *Proc Natl Acad Sci U S A* 106: 1903 – 1908

Komatsu N, Okamoto K, Sawa S, Nakashima T, Oh-hora M, Kodama T, Tanaka S, Bluestone JA, Takayanagi H (2014) Pathogenic conversion of Foxp3+ T cells into TH17 cells in autoimmune arthritis. *Nat Med* 20: 62 – 68

Lee HM, Bautista JL, Scott-Browne J, Mohan JF, Hsieh CS (2012) A broad range of self-reactivity drives thymic regulatory T cell selection to limit responses to self. *Immunity* 37: 475 – 486

Li J, Pan HF, Cen H, Tian J, Ma Y, Tao JH, Ye DQ (2011) Interleukin-21 as a potential therapeutic target for systemic lupus erythematosus. *Mol Biol Rep* 38: 4077 – 4081

Lin J, Yu Y, Ma J, Ren C, Chen W (2019) PD-1+CXCR5-CD4+T cells are correlated with the severity of systemic lupus erythematosus. *Rheumatology* 58: 2188 – 2192

Lisnevskaia L, Murphy G, Isenberg D (2014) Systemic lupus erythematosus. *Lancet* 384: 1878 – 1888

Loos J, Schmaul S, Noll TM, Paterka M, Schillner M, Loffel JT, Zipp F, Bittner S (2020) Functional characteristics of Th1, Th17, and ex-Th17 cells in EAE revealed by intravital two-photon microscopy. *J Neuroinflammation* 17: 357

Lu D, Shang G, He X, Bai XC, Zhang X (2021) Architecture of the Sema3A/PlexinA4/Neuropilin tripartite complex. *Nat Commun* 12: 3172

 © 2022 The Authors

Lyons JA, San M, Happ MP, Cross AH (1999) B cells are critical to induction of experimental allergic encephalomyelitis by protein but not by a short encephalitogenic peptide. *Eur J Immunol* 29: 3432 – 3439

Makiyama A, Chiba A, Noto D, Murayama G, Yamaji K, Tamura N, Miyake S (2019) Expanded circulating peripheral helper T cells in systemic lupus erythematosus: association with disease activity and B cell differentiation. *Rheumatology* 58: 1861 – 1869

Martin B, Auffray C, Delpoux A, Pommier A, Durand A, Charvet C, Yakonowsky P, de Boysson H, Bonilla N, Audemard A *et al* (2013) Highly self-reactive naive CD4 T cells are prone to differentiate into regulatory T cells. *Nat Commun* 4: 2209

McHugh RS, Whitters MJ, Piccirillo CA, Young DA, Shevach EM, Collins M, Byrne MC (2002) CD4(+)CD25(+) immunoregulatory T cells: gene expression analysis reveals a functional role for the glucocorticoid-induced TNF receptor. *Immunity* 16: 311 – 323

Merino R, Fossati L, Izui S (1992) The lupus-prone BXSB strain: the Yaa gene model of systemic lupus erythematosus. *Springer Semin Immunopathol* 14: 141 – 157

Milpied P, Renand A, Bruneau J, Mendes-da-Cruz DA, Jacquelin S, Asnafi V, Rubio MT, MacIntyre E, Lepelletier Y, Hermine O (2009) Neuropilin-1 is not a marker of human Foxp3+ Treg. *Eur J Immunol* 39: 1466 – 1471

Mizui M, Kikutani H (2008) Neuropilin-1: the glue between regulatory T cells and dendritic cells? *Immunity* 28: 302 – 303

Moran AE, Holzapfel KL, Xing Y, Cunningham NR, Maltzman JS, Punt J, Hogquist KA (2011) T cell receptor signal strength in Treg and iNKT cell development demonstrated by a novel fluorescent reporter mouse. *J Exp Med* 208: 1279 – 1289

Morse HC 3rd, Davidson WF, Yetter RA, Murphy ED, Roths JB, Coffman RL (1982) Abnormalities induced by the mutant gene Ipr: expansion of a unique lymphocyte subset. *J Immunol* 129: 2612 – 2615

Murphy ED, Roths JB (1979) A Y chromosome associated factor in strain BXSB producing accelerated autoimmunity and lymphoproliferation. *Arthritis Rheum* 22: 1188 – 1194

Niu H, Sobel ES, Morel L (2008) Defective B-cell response to T-dependent immunization in lupus-prone mice. *Eur J Immunol* 38: 3028 – 3040

Odegard JM, Marks BR, DiPlacido LD, Poholek AC, Kono DH, Dong C, Flavell RA, Craft J (2008) ICOS-dependent extrafollicular helper T cells elicit IgG production via IL-21 in systemic autoimmunity. *J Exp Med* 205: 2873 – 2886

Overacre-Delgoffe AE, Chikina M, Dadey RE, Yano H, Brunazzi EA, Shayan G, Horne W, Moskovitz JM, Kolls JK, Sander C *et al* (2017) Interferon-gamma drives Treg fragility to promote anti-tumor immunity. *Cell* 169: e11

Ozaki K, Spolski R, Feng CG, Qi CF, Cheng J, Sher A, Morse HC 3rd, Liu C, Schwartzberg PL, Leonard WJ (2002) A critical role for IL-21 in regulating immunoglobulin production. *Science* 298: 1630 – 1634

Pellet-Many C, Frankel P, Jia H, Zachary I (2008) Neuropilins: structure, function and role in disease. *Biochem J* 411: 211 – 226

Pons-Estel GJ, Alarcon GS, Scofield L, Reinlib L, Cooper GS (2010) Understanding the epidemiology and progression of systemic lupus erythematosus. *Semin Arthritis Rheum* 39: 257 – 268

Rao DA, Gurish MF, Marshall JL, Slowikowski K, Fonseka CY, Liu Y, Donlin LT, Henderson LA, Wei K, Mizoguchi F *et al* (2017) Pathologically expanded peripheral T helper cell subset drives B cells in rheumatoid arthritis. *Nature* 542: 110 – 114

Raveney BJ, Oki S, Yamamura T (2013) Nuclear receptor NR4A2 orchestrates Th17 cell-mediated autoimmune inflammation via IL-21 signalling. *PLoS One* 8: e56595

Raveney BJ, Oki S, Hohjoh H, Nakamura M, Sato W, Murata M, Yamamura T (2015) Eomesodermin-expressing T-helper cells are essential for chronic neuroinflammation. *Nat Commun* 6: 8437

Renand A, Milpied P, Rossignol J, Bruneau J, Lemonnier F, Dussiot M, Coulon S, Hermine O (2013) Neuropilin-1 expression characterizes T follicular helper (Tfh) cells activated during B cell differentiation in human secondary lymphoid organs. *PLoS One* 8: e85589

Sekiya T, Kashiwagi I, Yoshida R, Fukaya T, Morita R, Kimura A, Ichinose H, Metzger D, Chambon P, Yoshimura A (2013) Nr4a receptors are essential for thymic regulatory T cell development and immune homeostasis. *Nat Immunol* 14: 230 – 237

Shi J, Hou S, Fang Q, Liu X, Liu X, Qi H (2018) PD-1 controls follicular T helper cell positioning and function. *Immunity* 49: 264 – 274.e4

Shimizu J, Yamazaki S, Takahashi T, Ishida Y, Sakaguchi S (2002) Stimulation of CD25(+)CD4(+) regulatory T cells through GITR breaks immunological self-tolerance. *Nat Immunol* 3: 135 – 142

Singh K, Hjort M, Thorvaldson L, Sandler S (2015) Concomitant analysis of Helios and Neuropilin-1 as a marker to detect thymic derived regulatory T cells in naive mice. *Sci Rep* 5: 7767

Solomon BD, Mueller C, Chae WJ, Alabanza LM, Bynoe MS (2011) Neuropilin-1 attenuates autoreactivity in experimental autoimmune encephalomyelitis. *Proc Natl Acad Sci U S A* 108: 2040 – 2045

Stadinski BD, Shekhar K, Gomez-Tourino I, Jung J, Sasaki K, Sewell AK, Peakman M, Chakraborty AK, Huseby ES (2016) Hydrophobic CDR3 residues promote the development of self-reactive T cells. *Nat Immunol* 17: 946 – 955

Sugahara KN, Teesalu T, Karmali PP, Kotamraju VR, Agemy L, Greenwald DR, Ruoslahti E (2010) Coadministration of a tumor-penetrating peptide enhances the efficacy of cancer drugs. *Science* 328: 1031 – 1035

Suurmond J, Diamond B (2015) Autoantibodies in systemic autoimmune diseases: specificity and pathogenicity. *J Clin Invest* 125: 2194 – 2202

Teesalu T, Sugahara KN, Kotamraju VR, Ruoslahti E (2009) C-end rule peptides mediate neuropilin-1-dependent cell, vascular, and tissue penetration. *Proc Natl Acad Sci U S A* 106: 16157 – 16162

Tellier J, Nutt SL (2013) The unique features of follicular T cell subsets. *Cell Mol Life Sci* 70: 4771 – 4784

Theofilopoulos AN, Dixon FJ (1985) Murine models of systemic lupus erythematosus. *Adv Immunol* 37: 269 – 390

Tsokos GC, Lo MS, Costa Reis P, Sullivan KE (2016) New insights into the immunopathogenesis of systemic lupus erythematosus. *Nat Rev Rheumatol* 12: 716 – 730

Ueno H (2016) T follicular helper cells in human autoimmunity. *Curr Opin Immunol* 43: 24 – 31

Ueno H, Banchereau J, Vinuesa CG (2015) Pathophysiology of T follicular helper cells in humans and mice. *Nat Immunol* 16: 142 – 152

Vinuesa CG, Sanz I, Cook MC (2009) Dysregulation of germinal centres in autoimmune disease. *Nat Rev Immunol* 9: 845 – 857

Wyss L, Stadinski BD, King CG, Schallenberg S, McCarthy NI, Lee JY, Kretschmer K, Terracciano LM, Anderson G, Surh CD *et al* (2016) Affinity for self antigen selects Treg cells with distinct functional properties. *Nat Immunol* 17: 1093 – 1101

Yadav M, Louvet C, Davini D, Gardner JM, Martinez-Llordella M, Bailey-Bucktrout S, Anthony BA, Sverdrup FM, Head R, Kuster DJ *et al* (2012) Neuropilin-1 distinguishes natural and inducible regulatory T cells among regulatory T cell subsets *in vivo*. *J Exp Med* 209: S1 – S19

Yaniv G, Twig G, Shor DB, Furer A, Sherer Y, Mozes O, Komisar O, Slonimsky E, Klang E, Lotan E *et al* (2015) A volcanic explosion of autoantibodies in

systemic lupus erythematosus: a diversity of 180 different antibodies found in SLE patients. *Autoimmun Rev* 14: 75 – 79

Yin Y, Choi SC, Xu Z, Perry DJ, Seay H, Croker BP, Sobel ES, Brusko TM, Morel L (2015) Normalization of CD4+ T cell metabolism reverses lupus. *Sci Transl Med* 7: 274ra18

Yoshida R, Yoshioka T, Yamane S, Matsutani T, Toyosaki-Maeda T, Tsuruta Y, Suzuki R (2000) A new method for quantitative analysis of the mouse T-cell receptor V region repertoires: comparison of repertoires among strains. *Immunogenetics* 52: 35 – 45

Yu W, Jiang N, Ebert PJ, Kidd BA, Muller S, Lund PJ, Juang J, Adachi K, Tse T, Birnbaum ME *et al* (2015) Clonal deletion prunes but does not eliminate self-specific alphabeta CD8(+) T lymphocytes. *Immunity* 42: 929 – 941

Zinzow-Kramer WM, Weiss A, Au-Yeung BB (2019) Adaptation by naive CD4 (+) T cells to self-antigen-dependent TCR signaling induces functional heterogeneity and tolerance. *Proc Natl Acad Sci U S A* 116: 15160 – 15169

Zou W, Wolchok JD, Chen L (2016) PD-L1 (B7-H1) and PD-1 pathway blockade for cancer therapy: mechanisms, response biomarkers, and combinations. *Sci Transl Med* 8: 328rv4

License: This is an open access article under the terms of the Creative Commons Attribution License, which permits use, distribution and reproduction in any medium, provided the original work is properly cited.

 © 2022 The Authors

Expanded View Figures

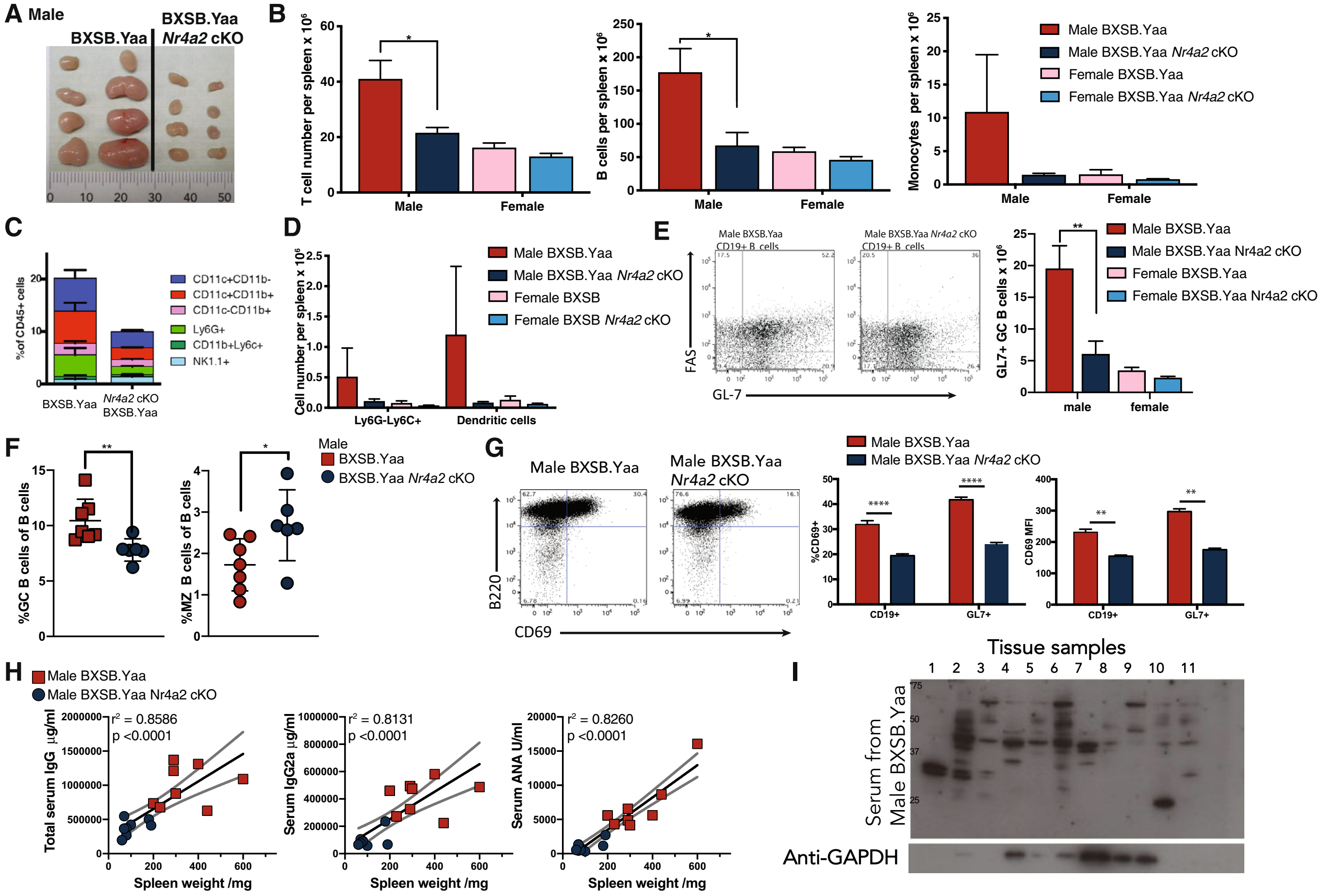

Figure EV1. T-cell-specific deletion of the *Nr4a2* gene abrogates systemic autoimmunity in BXSB mice.

A Inguinal lymph nodes from 16-week-old male BXSB.Yaa or BXSB.Yaa *Nr4a2* cKO mice were photographed.

B–G Spleens from male and female mice were disrupted and cell numbers were counted by flow cytometry: TcRb^{+} T cells (B, left), CD19^{+} B cells (B, center), and scatter gated CD45^{+}CD19^{-}TcR^{-} NK1.1^{-} monocytes (B, right). Non-T and -B cells from male mice were further fractionated by staining with CD11b, CD11c, Ly6G, Ly6C, as well as NK1.1 (C). CD45^{+}CD11b^{+}Ly6G-Ly6C^{+} cells (Ly6G-Ly6C^{+}) and CD45^{+}CD11c^{+} (dendritic cells) were also assessed from male and female mice (D). Germinal center B cell (CD19^{+}B220^{+} FAS^{+}GL7^{+}, GC B cells) numbers per spleen were also assessed: representative flow cytometry staining is shown (E, left), GC cell numbers for individual mice (E, Right) and GC B cells and CD19^{+}B220^{+}CD21^{-}CD23^{+} marginal zone (MZ) B cells measured as a proportion of total CD19^{+} B cells (F). B cell activation as measured by CD69 flow cytometry in total CD45^{+}TcR^{-}CD19^{+} B cells (CD19^{+}) and CD45^{+}TcR^{-}CD19^{+}GL7^{+} B cells (GL7^{+}), representative flow cytometry staining is shown (G, left), CD69^{+} cell populations as a percentage of parent cells (G, center), and CD69 mean fluorescent intensity (MFI) of cell populations (G, right). n = 4–8 mice per group and data represent 3–6 similar experiments. Error bars represent SEM for individual mice; n = 4–7; *P < 0.05, **P < 0.01, ***P < 0.001, ****P < 0.0001 unpaired two-tailed Student's t-test with Welch's correction. Data are representative of at least 10 independent experiments with 4–10 per group.

H Serum concentrations of antibodies (Y-axis) was compared with spleen weight (X-axis) from 16-week-old male BXSB.Yaa and male BXSB.Yaa *Nr4a2* cKO mice; for total IgG (left), IgG2a (center), and ANA (right). n = 8 mice per group; linear Spearman R correlation test; regression analysis (solid line) shown with 95% confidence intervals (dotted lines).

I IgG serum antibody binding to PAGE-resolved proteins isolated from 11 tissues from 16-week-old male BXSB.Yaa mice. Tissues are as follows: 1: salivary glands, 2: liver, 3: lung, 4: heart, 5: stomach, 6: kidney, 7: musele, 8: brain, 9: spinal cord, 10: small intestine, 11: large intestine.

© 2022 The Authors

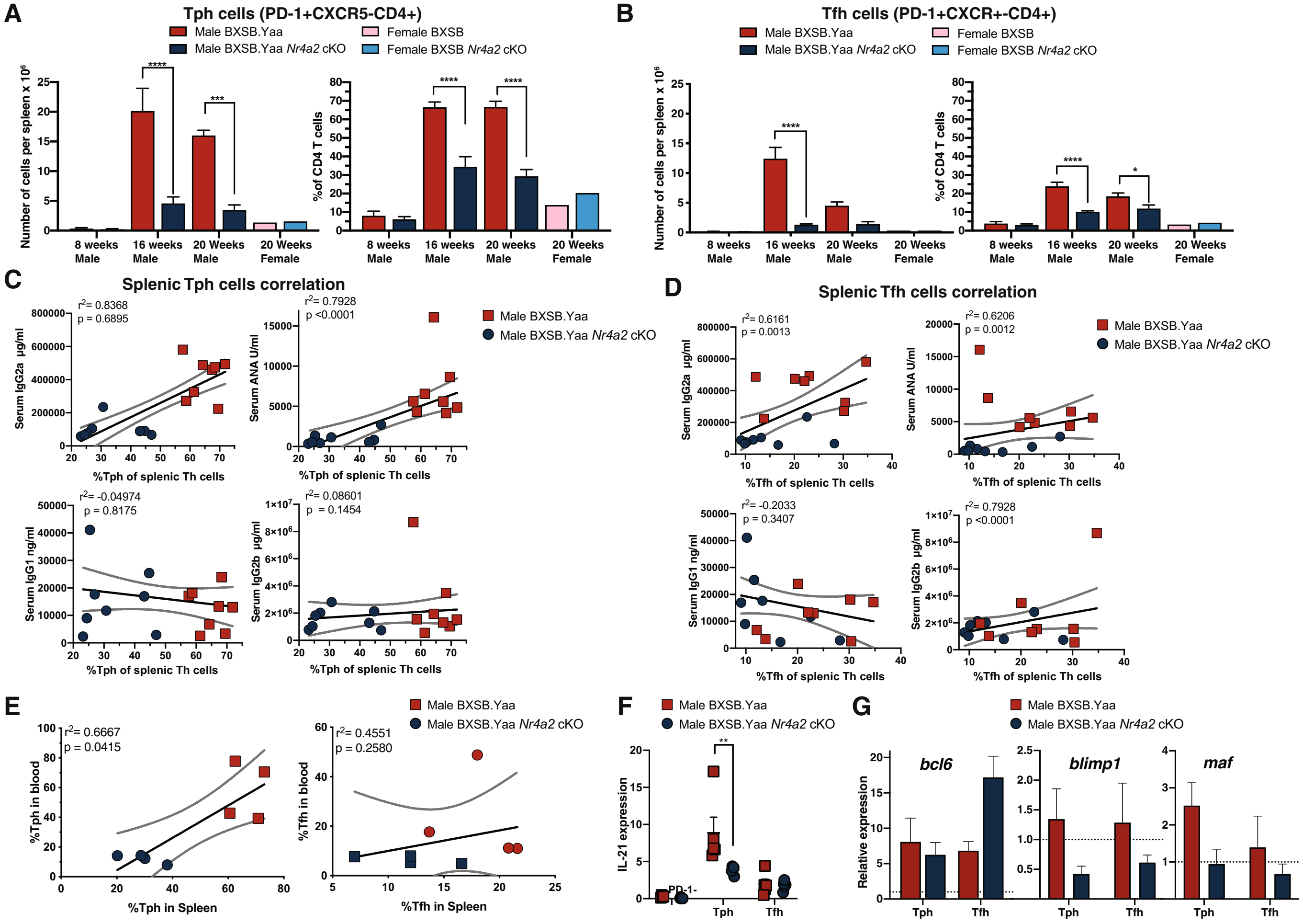

Figure EV2. Rapid expansion of diverse Th cell subsets in BXSB mice is NR4A2 dependent.

A–E Spleen cell suspensions from 8- to 20-week-old male and 16-week-old female BXSB.Yaa and BXSB.Yaa *Nr4a2* cKO mice were evaluated for populations within TcRβ$^+$CD4$^+$ Th cells by cell number per spleen and proportion among Th cells for Tph (PD-1$^+$CXCR5$^-$) cells (A) and Tfh (PD- 1$^+$CXCR5$^+$) cells (B). Data are representative of at least 4–10 independent experiments with 4–10 mice per group; error bars, mean with SEM; *$P < 0.05$, ***$P < 0.001$, ***$P < 0.001$ two-way ANOVA with Bonferroni's multiple-comparison test. For individual mouse, serum IgG2a, ANA, IgG1, and IgG2b level was compared with subset percentage in splenic Th cells for Tph cells (C) and Tfh cells (D). Linear regression lines shown with 95% CI; statistical testing by Spearman *R* test indicated; $n = 8$ per group across 2 independent experiments. For 16-week-old male mice, Th cell subset proportions in spleens (X-axis) were compared with subset proportion in blood (Y-axis) among whole Th cells for Tph cells (E, left) and Tfh cells (E, right). $n = 4$; Spearman *R* correlation test; linear regression analysis lines shown with 95% confidence intervals.

F, G Splenic Th cells from 16-week-old male BXSB.Yaa and BXSB.Yaa *Nr4a2* cKO mice purified by flow cytometric cell sorting into PD-1$^-$, PD-1$^+$CXCR5$^-$ (Tph), and PD-1$^+$CXCR5$^+$ (Tfh) cells and gene transcription for *il21* (F); **$P < 0.01$ two way ANOVA with Bonferroni's multiple-comparison test, and *bcl6*, *blimp1*, and *maf* (G) were assessed by real-time qPCR. Relative transcript levels were normalized to PD-1$^-$ cells (PD-1$^-$ cells were accorded level 1.0, indicated by dotted lines), $n = 4$ mice per group; error bars, mean with SEM; data are representative of at least three similar experiments.

 © 2022 The Authors

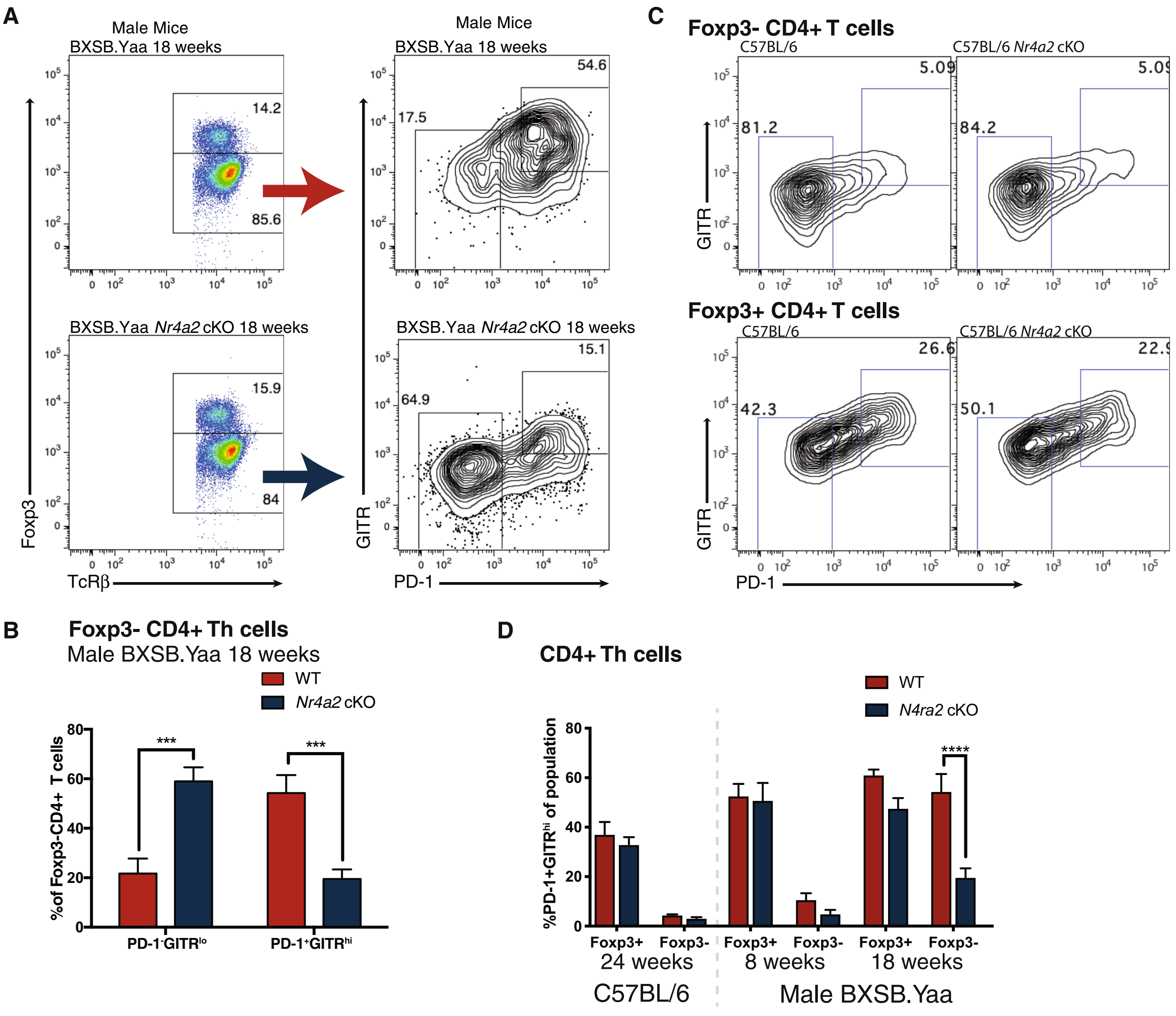

Figure EV3. Foxp3^{-} non-Treg Th cells subset expanded in diseased male BXSB.Yaa mice shares markers with Foxp3^{+} Treg cells.

A, B Single-cell suspensions from spleens of male BXSB.Yaa or male BXSB.Yaa Nr4a2 cKO mice aged 18 weeks were stained with surface antibodies against TcRβ, CD3, PD-1, and GITR before intracellular staining with anti-Foxp3. Foxp3 staining was used to select Foxp3^{-} conventional Th cells and GITR/PD-1 levels were assessed. Representative staining is shown (A) with the percentage of conventional PD-1^{-}GITR^{lo} and PD-1^{+}GITR^{hi} Th cells shown for Foxp3^{-} cells (B).***$P < 0.001$; Two-way ANOVA with Bonferroni's multiple comparisons test; n = 3–5 mice per group; error bars are SEM. These data are representative of three independent experiments.

C Single -ell suspensions from spleens of wild-type C57BL/6 or *Nr4a2* cKO C57BL/6 mice aged 24 weeks were stained and examined by flow cytometry and Foxp3 was used to gate T cells into Foxp3^{+} and Foxp3^{-}. GITR vs. PD-1 expression in CD4^{+}TcRβ^{+} T cells is shown for Foxp3^{-} and Foxp3^{+} These data are representative of three independent experiments (n = 2–4 mice per group);

D Single-cell suspensions from spleens of wild-type C57BL/6 or *Nr4a2* cKO C57BL/6 aged 24 weeks or male BXSB.Yaa or male BXSB.Yaa Nr4a2 cKO aged either 8 or18 weeks were stained with surface antibodies against, TcRβ, CD3, PD-1, and GITR, before intracellular staining with anti-Foxp3. Foxp3 staining was used to select conventional Th cells and Treg cells. The percentage PD-1^{+}GITR^{hi} Th cells is shown for Foxp3^{-} conventional Th cells and Foxp3^{+} Treg cells. n = 4–6 mice per group error bars, mean with SEM; ****$P < 0.0001$ two-way ANOVA with Bonferroni's multiple-comparison test.

© 2022 The Authors

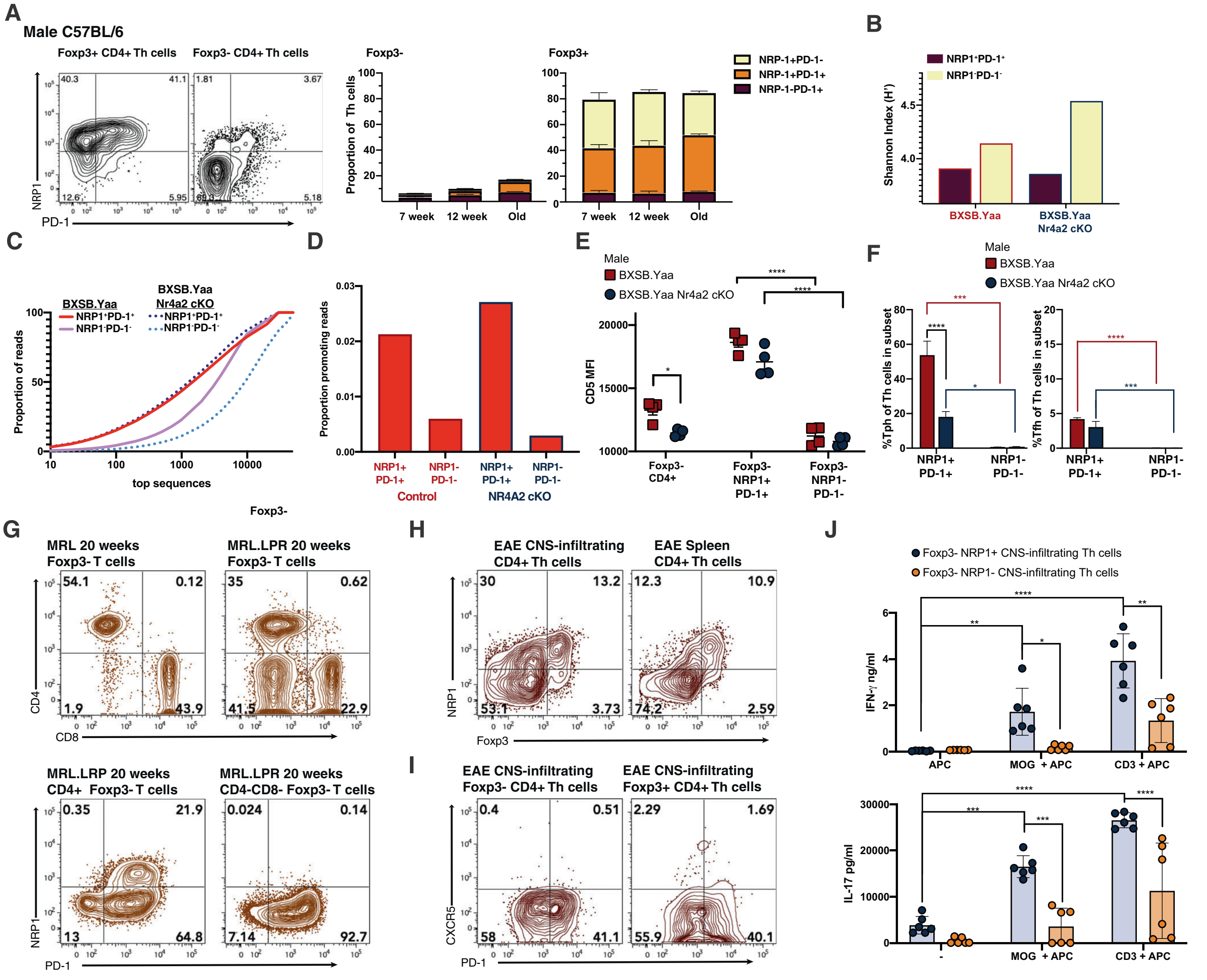

Figure EV4. NRP1 expression identifies a *Nr4a2*-dependent self-reactive Th cell subset expanded in SLE.

A Spleens from wild-type C57BL/6 mice were stained for NRP1 and PD-1 among TcRβ^+CD4$^+$Foxp3$^+$ T cells and TcRβ^+CD4$^+$Foxp3$^+$ T cells for flow cytometry. Representative staining from 12-week-old mice and proportions of NRP1/PD-1 subsets from mice aged 7, 12, and over 24 weeks old are shown (A), n = 3–8 per representative of least two independent litters; error bars, mean with SD.

B–D TcR repertoires were analyzed among NRP1$^+$PD1$^+$ and NRP1$^-$PD-1$^-$ -sorted Th subsets from 16-week-old male BXSB.Yaa and BXSB.Yaa *Nr4a2* cKO mice. Repertoire diversity was indicated by Shannon index (B) and by calculating the cumulative proportion of the total TcR sequence reads against decreasingly common unique sequences to show distribution skewing (C) and the number of reads for clones with self-reactivity promoting TcRs was calculated for the top 1,000 clones (D).

E CD5 expression was measured by flow cytometry among Foxp3$^-$ Th subsets from male BXSB.Yaa and BXSB.Yaa *Nr4a2* cKO mice aged 18 weeks. CD5 MFI is shown for the whole Foxp3$^-$CD4$^+$ T cells, and NRP1$^+$PD1$^+$ and NRP1$^-$PD-1$^-$ subsets of Foxp3$^-$ Th cells; n = 5 mice per group, error bars show SEM, *$P < 0.05$, ****$P < 0.0001$ two-way ANOVA with Bonferroni's multiple-comparison test; data are representative of at least three independent experiments.

F Foxp3$^-$CD4$^+$TcRβ^+ splenocytes from 18-week-old Male BXSB.Yaa and BXSB.Yaa *Nr4a2* cKO mice divided into NRP1$^+$PD-1$^+$ and NRP1$^-$PD-1$^-$ subsets were assessed for proportions of Tph (left) and Tfh (right) cells based on CXCR5/PD-1 flow cytometry; n = 5 mice per group; data are representative of three independent experiments; error bars show SEM; ****$P < 0.0001$, ***$P < 0.001$, *$P < 0.05$, two-way ANOVA with Bonferroni's multiple-comparison test.

G Splenocytes from 20-week-old female MRL or MRL.LPR mice were stained for flow cytometry and NRP1 and PD-1 expression was measured for TcRβ^+CD4$^-$CD8$^-$ cells. Data are representative of five individual mice per group.

H–J EAE was induced in male C57BL/6 Foxp3^{hCD2} reporter mice. On day 17 peak disease, splenocytes and CNS-infiltrating T cells were isolated and stained for flow cytometry. Representative FACS staining shown for NRP1 versus Foxp3 reporter for CD45$^+$CD11b$^-$TcRb$^+$ Th cells (H) and for CXCR5 and PD-1 for Foxp3$^-$ or Foxp3$^+$ NRP1$^+$CD45$^+$CD11b$^-$TcRb$^+$ CNS Th cells (I). Pools of three mice were combined and NRP1$^+$ and NRP1$^-$ Foxp3$^-$ Th cells were purified by FACS sorting. Non-T-cell fractions were irradiated and used as APC. Th cells and APC were co-cultured in the presence or absence of 100 μg/ml MOG or 2 μg/ml anti-CD3. After 96 h, supernatants were collected and cytokine concentrations were measured by ELISA (J). Error bars, SD ****$P < 0.0001$, **$P < 0.01$, *$P < 0.05$ two-way ANOVA analysis with Bonferroni's multiple-comparison test. Points show triplicates for two pools of three individual mice. Data are representative of at least two independent experiments with six mice per experiment.

© 2022 The Authors

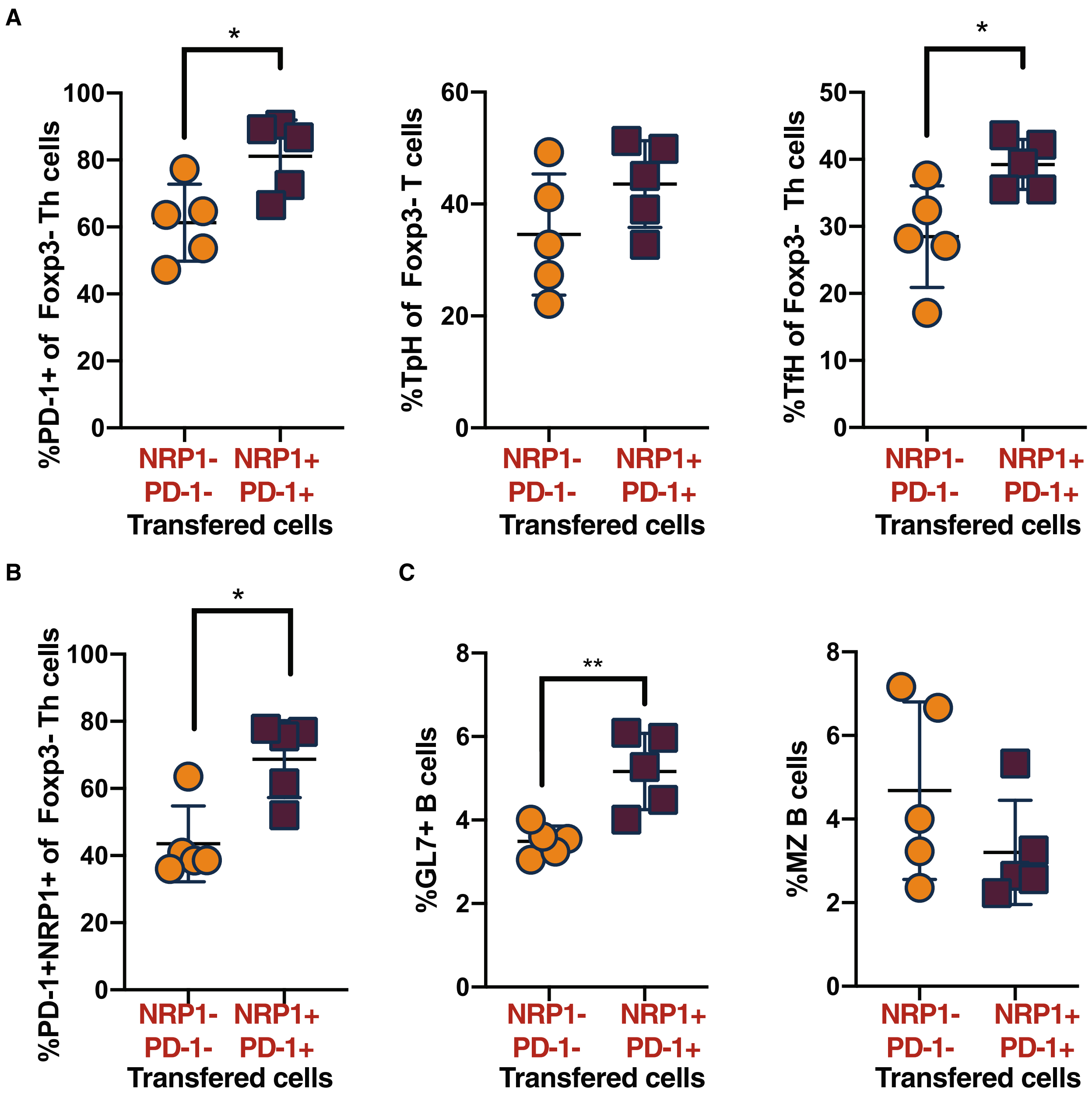

Figure EV5. NRP1/PD-1-expressing Th cells are pathogenic.

A–C Groups of male BXSB.Yaa *Nr4a2* cKO mice aged 18 weeks received either NRP1$^+$PD-1$^+$ CD4$^+$ Foxp3$^-$ T cells or NRP1$^-$PD-1$^-$ CD4$^+$ Foxp3$^-$ T cells from 20-week-old donor Male Foxp3^{hCD2} × BXSB.Yaa mice. Four weeks after transfer, spleen cells were analyzed by flow cytometry for PD-1$^+$ T cells among Foxp3$^-$CD4$^+$ Th cells (A), $^*P < 0.05$ two-tailed unpaired Student's *t*-test. NRP1$^+$PD-1$^+$ cells among Foxp3$^-$CD4$^+$ Th cells (B), $^*P < 0.05$ two-tailed Mann–Whitney U test, and GL7$^+$ GC B cells and CD21$^+$CD23$^-$ marginal zone B cells (C), $^{**}P < 0.01$ two-tailed unpaired Student's *t*-test. $n = 5$ mice per group; bars show mean with SD; data are representative of two independent experiments.

© 2022 The Authors

Appendix

Table of contents

Appendix Fig. S1
Appendix Fig. S2
Appendix Fig. S3
Appendix Fig. S4
Appendix Table S1 Most Common TcR Clones
Appendix Table S2. Characteristics of Human subjects
Appendix Table S3 List of antibodies

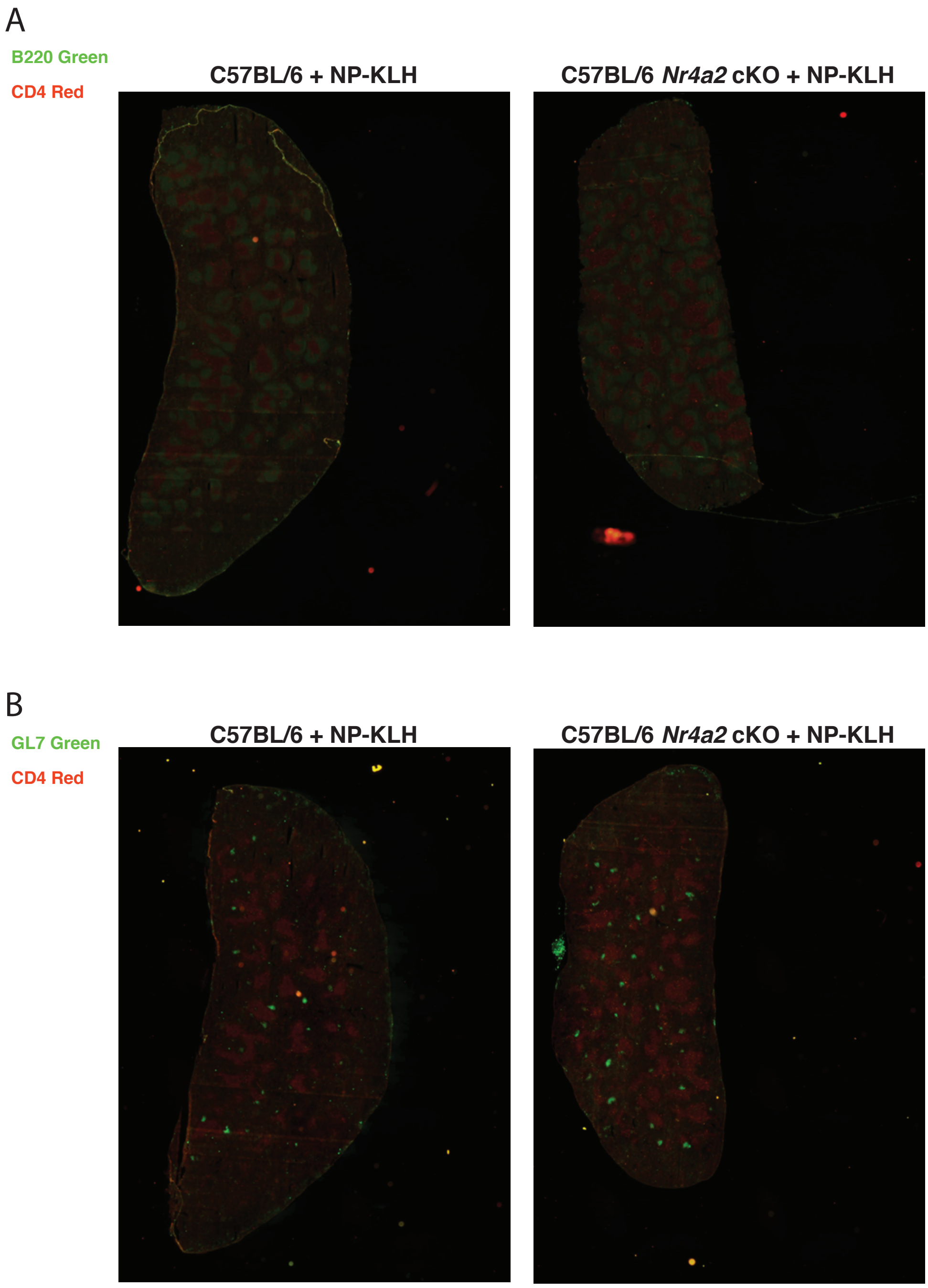

Appendix Fig. S1 – Normal germinal center formation induced by foreign antigen in absence of Nr4a2

Male C57BL/6 *Nr4a2* cKO mice (Right) or wildtype littermate C57BL/6 mice (left) were immunised twice with NP-KLH emulsified in alum. After 2 weeks, 7μm spleen sections were stained with Red:Alexa-Fluor-594-CD4 and Green: FITC-B220 (**A**) to visualise Th cells (red) entering B cell zones (green) or FITC-GL7 (**B**) to visualise Germinal Center formation (green). Representative photomicrographs show whole spleens at 2x magnification.

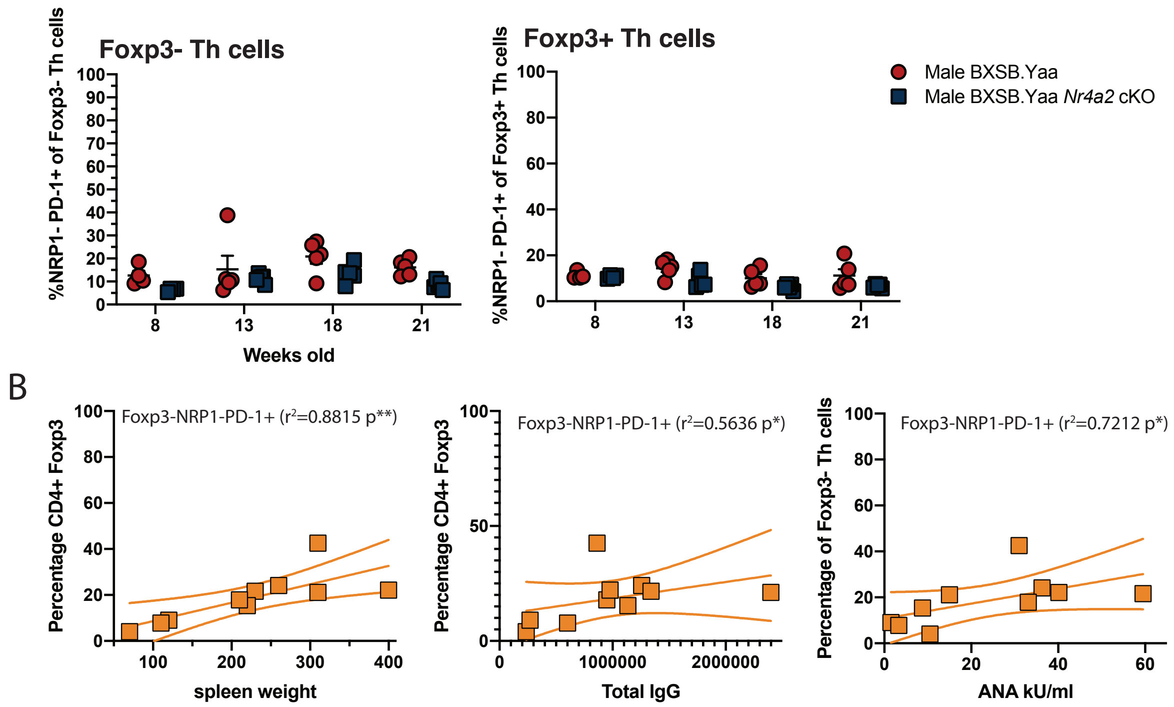

Appendix Fig. S2 NRP1$^-$ PD-1$^+$ Foxp3$^-$ Th cells can upregulate NRP1

Groups of Male BXSB.Yaa and Male BXSB.Yaa *Nr4a2* cKO mice were sacrificed at the indicated ages and splenic TcRβ$^+$CD4$^+$Foxp3- (**A**, left) and TcRβ$^+$CD4$^+$Foxp3$^+$ (**A**, right) were assessed for proportion of NRP1-PD-1$^+$ cells. n=4, Data is representative of at least 2 independent experiments, error bars are SD.

Proportion of NRP1-PD-1$^+$ and NRP1$^+$PD-1$^+$ cells (Y-Axis) from splenic TcRβ$^+$CD4$^+$Foxp3- of 18 week old Male BXSB.Yaa and Male BXSB.Yaa *Nr4a2* cKO was compared with (X-axis) spleen weight (**B**, left), and serum levels of IgG (**B**, center) and ANA (**B**, right). n=8, linear regression line with 95% CVs shown; data is representative of 2 independent experiments.

Appendix Figure S3

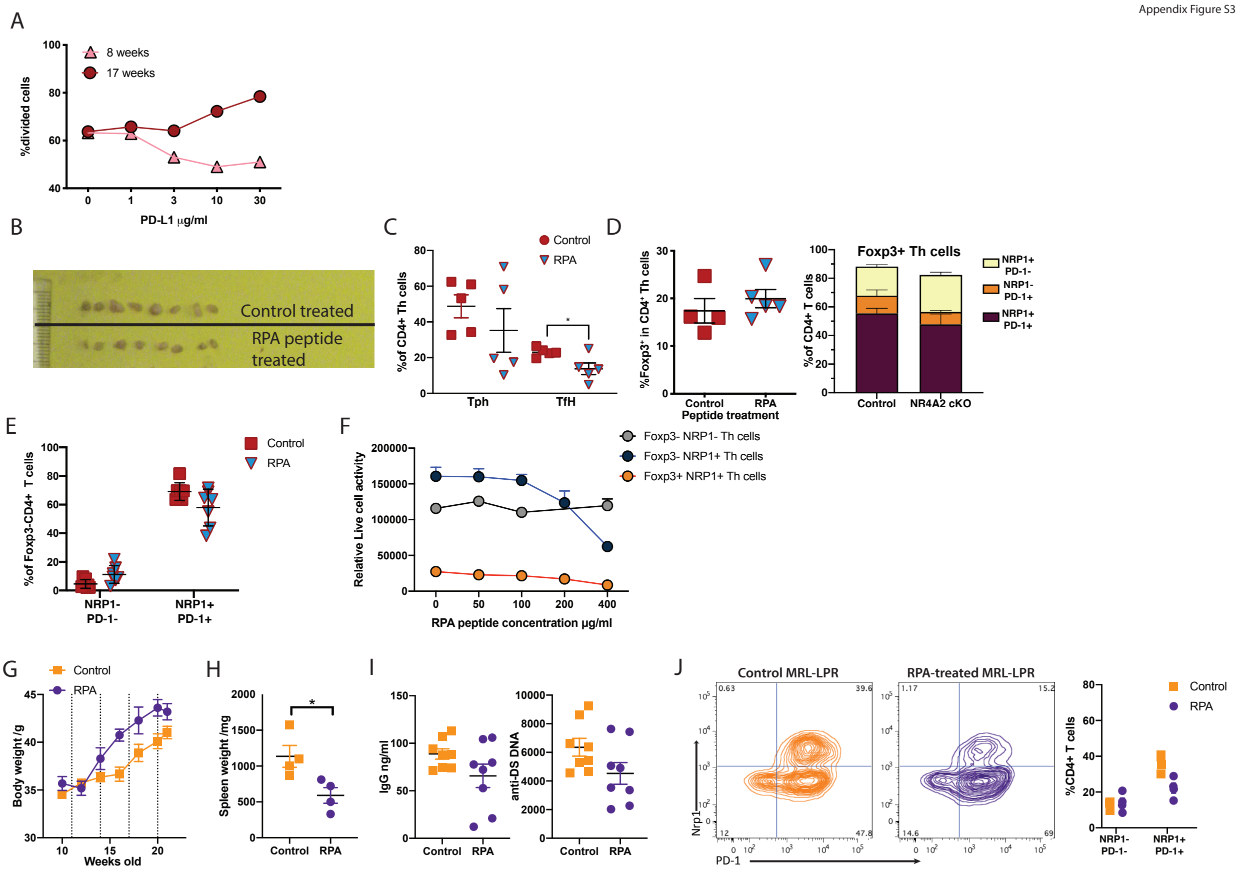

Appendix Fig. S3 Therapeutic intervention targeting NRP1 ameliorates systemic autoimmunity

Spleen cells from Male BxSB.Yaa mice at 8 weeks or 17 weeks of age were sorted by flow cytometry into TcRb$^+$CD4$^+$PD-1$^+$ populations and labelled with Cell trace Violet. Cells were restimulated *in vitro* using plate-bound anti-CD3 (10μg/ml) in the presence of recombinant PD-L1 at the indicated concentrations for 72 hours. Proliferation was calculated by Cell trace violet dilution to indicate the proportion of divided cells for PD-1$^+$ Th cells (**A**). n=4 mice pooled from 1 of 2 independent experiments.

Male BXSB.Yaa mice were treated with an i.p. administration of 200 μM of RPA peptide in PBS (RPA) or PBS alone (Control) at 10, 12, and 14 weeks of age. Mice were sacrificed at 16 weeks' old and inguinal lymph nodes were photographed (**B**). Spleen cells were stained for surface and intracellular markers and analysed by flow cytometry. Proportion of Tph (CXCR5-PD-1$^+$) and Tfh (CXCR5$^+$PD-1$^+$) of TcRβ$^+$CD4$^+$ cells was measured (**C**), and Foxp3$^+$ of TcRβ$^+$CD4$^+$ (**D**, left), and NRP1$^+$PD-1$^-$ and NRP1$^+$PD-1$^+$ of TcRβ$^+$CD4$^+$Foxp3$^+$ was calculated (**D**, right). n=4-5; data representative of 5 independent experiments.

Proportion of sub-populations in splenic TcRb$^+$CD4$^+$ conventional Th cells was measured by NRP1/PD-1 staining (**E**). * P<0.05 Two tailed unpaired t-test with Welch's correction.

Populations of Foxp3- Th cells were sorted by flow cytometry from spleens of 18 week old Male BXSB.Yaa mice, using the Foxp3^{hCD2} reporter system based on NRP1 and PD-1 expression to yield 3 subsets: TcRb$^+$CD4$^+$Foxp3-NRP1-PD-1-, TcRb$^+$CD4$^+$Foxp3-NRP1-PD-1$^+$, and TcRb$^+$CD4$^+$Foxp3-NRP1$^+$PD-1$^+$. They were then stimulated *in vitro* with pb-anti-CD3 and s-anti-CD28 antibodies with RPA peptide added at the indicated concentrations. On day 6, relative live cell numbers were measured by Cell Titer Glo 2 assay (**F**). These data are representative of 4 independent experiments.

MRL.LPR mice were treated with 200µM of RPA peptide in PBS (RPA) or PBS alone (Control), 11, 14, 17, and 20 weeks of age and mice were weighted fortnightly from week 10. Body weight is shown for each group by age with dotted lines indicating treatments (**G**). Mice were sacrificed at 21 weeks old and spleens weighed (**H**) and serum assessed for total IgG (**I, left**) and anti-DS DNA (**I, right**) concentration. n=10, statistical testing by Two-tailed unpaired t-test with Welch's correction * $P<0.05$. Data is representative of 4 independent experiments.
MRL.LPR mice were treated with 500 µM of RPA peptide in PBS (RPA) or PBS alone (Control), 11, 14, 17, and 20 weeks of age and mice were sacrificed at 21 weeks old. Spleen cells were stained for flow cytometric analysis for NRP1/PD-1 amongst $TcR\beta^{+}CD4^{+}Foxp3^{-}$ Th cells; representative staining shown and collective data (**J**). n =5; data is representative of 4 independent experiments.

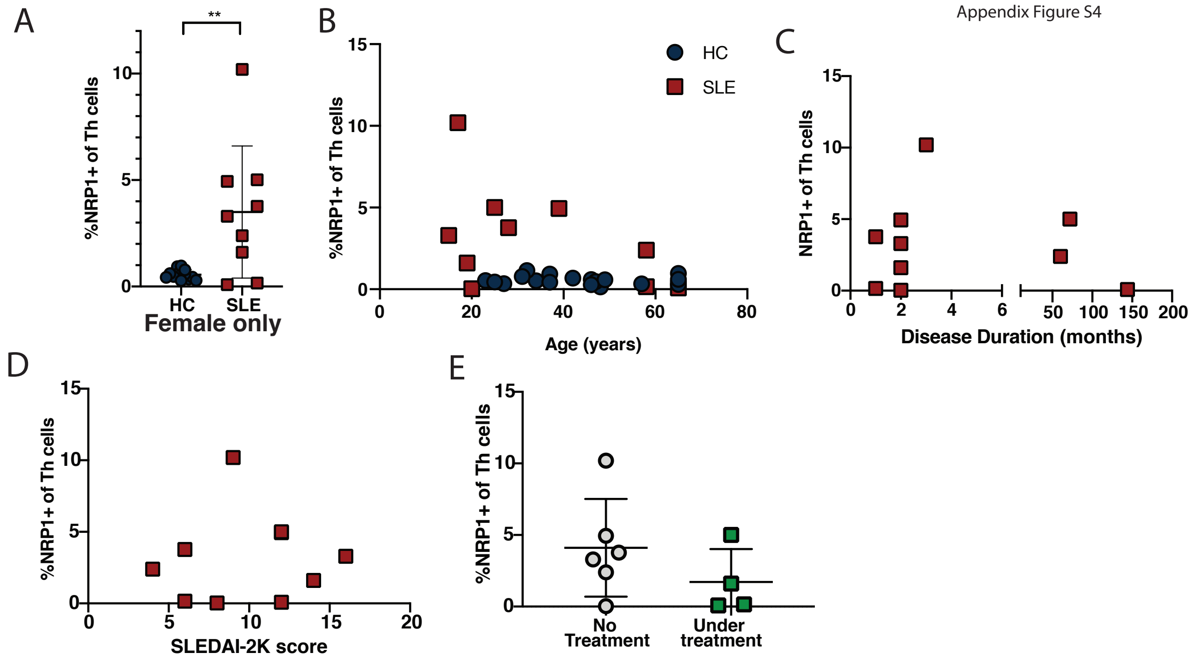

Appendix Fig. S4 NRP1-expressing Th cells are upregulated in human SLE patients

NRP1 expression in $CD3^+CD4^+$ Th cells was measured in 12 female healthy control (HC) and 9 female SLE subjects amongst PBMC (**A**) **p<0.001Unpaired Student's T test.
The level of NRP1-expressing cells amongst $CD4^+CD3^+$ Th cells was compared with subject age for 18 HC and 10 SLE subjects (**B**) and disease duration for 10 SLE subjects (**C**) No correlation with age for HC group $r^2 = 0.005474$, p =0.7894; weak negative correlation for SLE group $r^2 = 0.2184$, p =0.173 , Two-tailed Pearson correlation coefficient tests

The level of NRP1-expressing cells amongst $CD4^+CD3^+$ Th cells was compared with SLE disease activity (SLE Disease Activity Index 2000; SLE-DAI-2K score)for 10 SLE subjects (**D**) Not significant, $r^2 = 0.00695$, p =0.8189, Two-tailed Pearson correlation coefficient test
10 SLE patients were divided into 2 groups based on no current treatment (n=6) or currently under treatment (either MTX or PSL; n=4) and $NRP1^+$ Th cell level was compared between groups (**E**). Not significant, >0.05 Unpaired Student's T test.

Hydrophilic residue 6-7 (Limiting doublet)

Hydrophobic residue 6-7 (Promoting doublet)

Top 30 reads of control $CD4^+$ T cells

TRBV	TRBJ	CDR3β sequence	No.of reads		P6-P7 doublet
			Control	NR4A2cKO	
TRBV29	TRBJ1-1	CASSLGQNTEVFF	1957	13	GQ
TRBV13-3	TRBJ1-6	CASSFRDSYNSPLYF	1748	0	RD
TRBV3	TRBJ2-2	CASSLGLSNTGQLYF	1449	0	GL
TRBV12-2	TRBJ2-5	CASSLELGGQDTQYF	1313	0	EL
TRBV13-3	TRBJ2-2	CASGGGLTGQLYF	1252	0	GL
TRBV13-3	TRBJ2-1	CASSDTGGAYNYAEQFF	1087	0	TG
TRBV12-2	TRBJ2-5	CASSTTGNQDTQYF	1044	0	TG
TRBV13-3	TRBJ2-5	CASSYWGGKDTQYF	1028	0	WG
TRBV13-3	TRBJ1-1	CASSGGSNTEVFF	849	0	GS
TRBV3	TRBJ1-3	CASSSTGDGNTLYF	769	0	TG
TRBV5	TRBJ2-2	CASSQPGTANTGQLYF	585	0	PG
TRBV13-2	TRBJ2-1	CASRGNYAEQFF	566	0	NY
TRBV15	TRBJ1-1	CASSFAGAEVFF	564	0	AG
TRBV5	TRBJ1-1	CASSDGAEVFF	555	0	GA
TRBV13-3	TRBJ1-1	CASSQPGQTEVFF	555	0	PG
TRBV12-2	TRBJ2-5	CASSLDWGDTQYF	531	10	DW
TRBV20	TRBJ2-3	CGARAQSAETLYF	523	0	QS
TRBV3	TRBJ1-2	CASSLVANSDYTF	522	0	VA
TRBV5	TRBJ2-1	CASSQKGGNYAEQFF	507	0	KG
TRBV5	TRBJ2-7	CASSQEDWGGYEQYF	494	0	ED
TRBV16	TRBJ2-7	CASSLETGGYEQYF	492	0	ET
TRBV5	TRBJ1-4	CASSQPNERLFF	490	0	PN
TRBV1	TRBJ1-5	CTCSADNGHNNQAPLF	469	0	DN
TRBV29	TRBJ2-2	CASSRQGASGQLYF	456	0	QG
TRBV5	TRBJ2-5	CASSQDGGADTQYF	449	0	DG
TRBV5	TRBJ1-5	CASSQDYNNQAPLF	438	0	DY
TRBV5	TRBJ1-6	CASSGTTSYNSPLYF	435	0	TT
TRBV5	TRBJ1-1	CASSPHRDAEQFF	432	0	HR
TRBV2	TRBJ2-1	CASSQPGQNTEVFF	432	0	PG
TRBV13-3	TRBJ2-1	CASGGLGGSALYF	431	0	LG

Top 30 reads of NR4A2 cKO $CD4^+$ T cells

TRBV	TRBJ	CDR3β sequence	No.of reads		P6-P7 doublet
			Control	NR4A2cKO	
TRBV15	TRBJ1-1	CTCSGGQNSDYTF	0	2407	GQ
TRBV13-2	TRBJ2-5	CASSLRGDQDTQYF	0	1341	RG
TRBV12-2	TRBJ2-7	CASSGGGKNAEQFF	0	1242	GG
TRBV1	TRBJ2-7	CASGDPGGNERLFF	0	1102	PG
TRBV31	TRBJ1-3	CSSRLGGRDAETLYF	0	960	GG
TRBV15	TRBJ2-5	CASGDEGGNTLYF	0	933	EG
TRBV13-3	TRBJ2-2	CTCSAGPRDRAAEQFF	0	896	GP
TRBV13-2	TRBJ2-5	CASSYRRDPYSGNTLYF	0	777	RR
TRBV15	TRBJ2-2	CASSLETGGYAEQFF	0	766	ET
TRBV13-2	TRBJ2-4	CATRDRGHERLFF	0	764	RG
TRBV1	TRBJ2-7	CTCSGQIQDTQYF	0	759	QI
TRBV13-2	TRBJ2-4	CASSHDRNQDTQYF	0	727	DR
TRBV13-2	TRBJ2-7	CASSQDWNNYAEQFF	0	724	DW
TRBV15	TRBJ1-4	CASSLNSDYTF	0	662	NS
TRBV1	TRBJ1-5	CASSWGGAGDTQYF	0	623	GG
TRBV2	TRBJ2-7	CASSQEGGASGNTLYF	0	584	EG
TRBV31	TRBJ2-4	CASSQEDRGNTEVFF	0	567	ED
TRBV13-2	TRBJ1-1	CASSLAGGSYEQYF	0	565	AG
TRBV30	TRBJ2-3	CASGASDRGSSQNTLYF	0	556	SD
TRBV5	TRBJ2-2	CASSPGGSAETLYF	0	543	GG
TRBV3	TRBJ2-4	CASSLAGGAGEQYF	0	531	AG
TRBV13-3	TRBJ2-7	CTCSADRWGKDEQYF	0	529	DR
TRBV15	TRBJ2-2	CTCSRQGGGGNTLYF	0	527	QG
TRBV15	TRBJ1-1	CASSDAGARGERLFF	0	515	AG
TRBV1	TRBJ2-5	CASGDAMGGRGQNTLYF	0	495	AM
TRBV1	TRBJ2-1	CASGDAGGAYEQYF	0	488	AG
TRBV5	TRBJ2-7	CASGGDKYEQYF	0	482	DK
TRBV13-1	TRBJ1-4	CASGDAGTGGAGYEQYF	0	480	AG
TRBV5	TRBJ1-3	CASSLRDWGAETLYF	0	455	RD
TRBV13-2	TRBJ1-5	CASSWGSSYEQYF	0	454	GS

Appendix Table S1 – Most common Th cells clones by CDR3β TcR sequences read number in SLE-like disease

Memory splenic T cells ($TcRb^+CD4^+CD44^{hi}CD62L^{lo}$) were sorted from individual 20 week old BxSB mice either Control or NR4A2 cKO. These cells were subjected to unbiased TCR repertoire analysis using next-generation sequencing. Sequences were categorized based on residues CDR3 position 6&7: hydrophobic P6/7 doublets (promoting self-reactivity) and hydrophilic doublets (limiting self-reactivity). Unique TcR sequences were ordered by most common clones based on read number.

The most numerous 30 CDR3 reads are listed for control T cells (left) and NR4A2 cKO T cells (right) with hydrophobic P6/7 doublets (promoting self-reactivity) coloured red and hydrophilic P6/7 doublets coloured (limiting self-reactivity) blue.

Appendix Table 2 - Characteristics of Human subject Cohorts

	SLE	Healthy controls (HC)
Number	10	18
Male (%)	1(10%)	6 (37%)
Female (%)	9(90%)	12 (67%)
Age±sd<br>(range)	34.40±19.22<br>(15-64)	43.11±13.59 (23-64)
Onset Age±sd<br>(range)	32.10±17.06<br>(15-43)	N/A
Disease duration months ±sd<br>(range)	28.90±48.53<br>(1-144)	N/A
SLEDAI-2K<br>±sd<br>(range)	9.8±3.9<br>(4-16)	N/A
Treament<br>(numbers of subjects)	No treatment - 6<br>MTX -1<br>PSL alone - 1<br>PSL + CyA -1<br>PSL +HCQ - 1	No immunosuppressive drugs - 18

Drug abbreviations: MTX- methotrexate; PSL- prednisone; CyA - Cyclosporin A; HCQ – Hydroxychloroquine

Antibody Name	Specificity	Clone	Supplier
CD11b	Anti-mouse/human	M1/70	Biolegend
CD11c	Anti-mouse	N418	Biolegend
CD127	anti-human	AO19D5	Biolegend
CD16	Anti-mouse	W20015B	Biolegend
CD184/CXCR4	Anti-mouse	L276F12	Biolegend
CD185/CXCR5	Anti-mouse	L138D7	Biolegend
CD2	anti-human	TS1/8	Biolegend
CD21	Anti-mouse	7.00E+09	Biolegend
CD23	Anti-mouse	B3B4	Biolegend
CD25	Anti-mouse	PC61	Biolegend
CD279/PD-1	Anti-mouse	297.1A12	Biolegend
CD279/PD-1	anti-human	EH12.2H7	Biolegend
CD28	Anti-mouse	37.51	Biolegend
CD3	anti-human	OKT3	Biolegend
CD304/NRP1	Anti-mouse	3E12	Biolegend
CD357/GITR	Anti-mouse	DTA-1	Biolegend
CD3e	Anti-mouse	2C11	Biolegend
CD4	Anti-mouse	GK1.5	Biolegend
CD4	anti-human	RPA-T4	Biolegend
CD44	Anti-mouse	IM7	Biolegend
CD45	Anti-mouse	30-F11	Biolegend
CD45R/B220	Anti-mouse	RA3.6B2	Biolegend
CD45RA	anti-human	HI100	Biolegend
CD45RO	anti-human	UCHL1	Biolegend
CD62L	Anti-mouse	W180.21D	Biolegend
CD8	Anti-mouse	53-6.7	Biolegend
CD8	anti-human	SK1	Biolegend
CD95	Anti-mouse	SA367H8	Biolegend
Foxp3	Anti-mouse	MF-14	Biolegend
GL7	Anti-mouse	GL7	Biolegend
ICOS	Anti-mouse/human	C398.4A	Biolegend
Ly6C	Anti-mouse	HK1.4	Biolegend
Ly6G	Anti-mouse	1A1	Biolegend
NK1.1	Anti-mouse	S17016D	Biolegend
NRP1	anti-human	12C2	Biolegend
TcRb	Anti-mouse	H57.397	Biolegend

Appendix Table 3
List of Antibodies used
