# Supplementary figures and images for: Neuropilin‐1 (NRP1) expression distinguishes self‐reactive helper T cells in systemic autoimmune disease
Source: EMBO Mol Med. 2022 Sep 7;14(10):e15864. doi: 10.15252/emmm.202215864 (PMC9549730; doi:10.15252/emmm.202215864)

## Slide 1
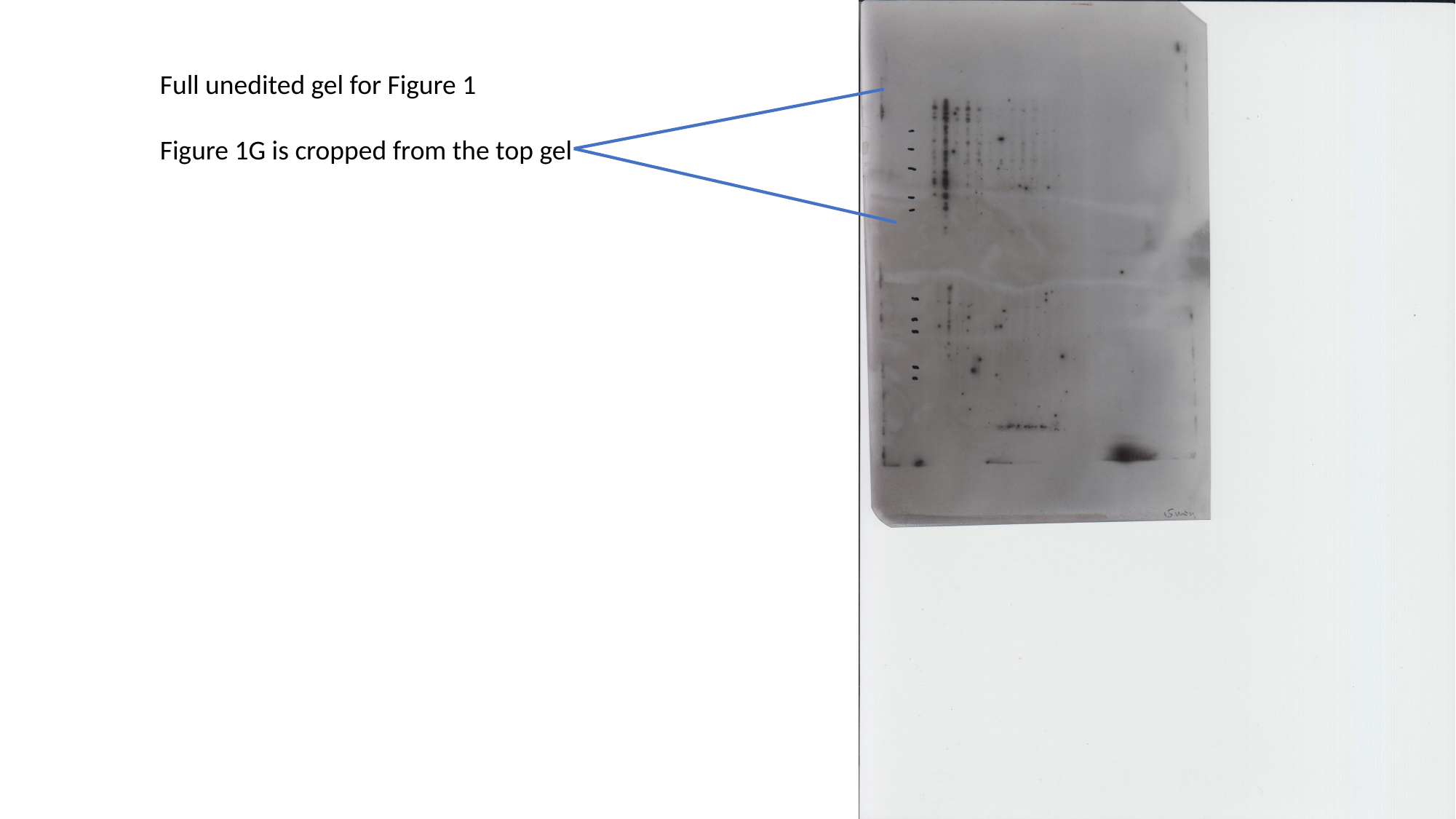

Full unedited gel for Figure 1
Figure 1G is cropped from the top gel

Supplement: Supplementary file 4 — Source Data for Figure 1 [file EMMM-14-e15864-s003.pptx]
