# Supplementary material for: Neuropilin‐1 (NRP1) expression distinguishes self‐reactive helper T cells in systemic autoimmune disease
Source: EMBO Mol Med. 2022 Sep 7;14(10):e15864. doi: 10.15252/emmm.202215864 (PMC9549730; doi:10.15252/emmm.202215864)
Supplement: Supplementary file 6 [file EMMM-14-e15864-s002.pdf]

EMBO Molecular Medicine

Neuropilin-1 expression distinguishes self-reactive helper T cells in systemic autoimmune disease

Ben Raveney, Yosif El-Darawish, Wakiro Sato, Yoshiyuki Arinuma, Kunihiro Yamaoka, Shohei Hori, Takashi Yamamura, and Shinji Oki
DOI: 10.15252/emmm.202215864

Corresponding author: Shinji Oki (soki@ncnp.go.jp)

Review Timeline:

Submission Date:	10th Feb 22
Editorial Decision:	2nd Mar 22
Revision Received:	14th Jul 22
Editorial Decision:	5th Aug 22
Revision Received:	8th Aug 22
Accepted:	15th Aug 22

Editor: Zeljko Durdevic

Transaction Report:

(Note: With the exception of the correction of typographical or spelling errors that could be a source of ambiguity, letters and reports are not edited. Depending on transfer agreements, referee reports obtained elsewhere may or may not be included in this compilation. Referee reports are anonymous unless the Referee chooses to sign their reports.)

2nd Mar 2022

Dear Dr. Raveney,

Thank you for the submission of your manuscript to EMBO Molecular Medicine. We have now received feedback from the three reviewers who agreed to evaluate your manuscript. As you will see from the reports below, the referees acknowledge the interest of the study but also raise important concerns that should be addressed in a major revision.

Further consideration of a revision that addresses reviewers' concerns in full will entail a second round of review. EMBO Molecular Medicine encourages a single round of revision only and therefore, acceptance or rejection of the manuscript will depend on the completeness of your responses included in the next, final version of the manuscript. For this reason, and to save you from any frustrations in the end, I would strongly advise against returning an incomplete revision.

We would welcome the submission of a revised version within three months for further consideration. Please let us know if you require longer to complete the revision.

Please use this link to login to the manuscript system and submit your revision: https://embomolmed.msubmit.net/cgi-bin/main.plex

I look forward to receiving your revised manuscript.

Yours sincerely,

Zeljko Durdevic

Zeljko Durdevic
Editor
EMBO Molecular Medicine

***** Reviewer's comments *****

Referee #1 (Remarks for Author):

In this work, Raveney et al. used animal models (BXSB, MRL lpr/lpr) of systemic autoimmune diseases to identify a subset of CD4 helper T cells that seems crucial in driving B cells to produce characteristic autoantibodies. These CD4 T cells are dependent on the transcription factor NR4A2 and express at their cell surface both PD-1 and Neuropilin-1. The autoreactivity of these CD4 T cells is suspected based on molecular and functional features. Importantly, targeting Neuropilin-1 in vivo prevented the development of systemic autoimmune diseases in mice. Importantly, the authors went on to assess whether expression of Neuropilin-1 was increased on CD4 T cells from patients with systemic lupus erythematosus, and this turned out indeed to be the case.
The study is well designed, and carefully executed. The results are fully novel and may well lead to the identification of a therapeutic target for patients with systemic autoimmune diseases. The manuscript is clearly written. There are, however, several important issues that need to be clarified by both text changes and complementary data.

Important issues to be addressed:

1. From the data presented it is not fully clear that Neuropilin-1, rather than PD-1 or the combination of both, characterizes the autoreactive CD4 T cells. So far, the conclusion is based on indirect evidence such as TCR CDR3β sequences. To further explore this question the authors should perform adoptive transfer experiments with PD-1+ Neuropilin-1-negative vs. PD-1+ Neuropilin-1-positive CD4 T cells. Both populations can be clearly separated in MRL-Lpr mice (Suppl Fig 6I). Alternatively, the authors could sort the CD4 T cells based on PD-1 and Neuropilin-1 expression and assess ex vivo their self-reactive potential.
2. The RPA peptide is said to target Neuropilin-1-expressing cells and promote an apoptotic process. It is unclear why the frequency of Neuropilin-1-expressing conventional CD4 T cells is only partly reduced and why Neuropilin-1-expressing Foxp3 regulatory CD4 T cells are fully spared. A discussion on the cell types expressing Neuropilin-1 and the consequences of their targeting by the RPA peptide in warranted.
3. The statistical analyses need to be reassessed, possibly with the help of statistical expertise. Specifically, it is unclear why one-tailed tests were performed; whether corrections for multiple comparisons were systematically applied, why Mann-Whitney test was used when comparing more than 2 groups, and has a correction for unequal variance been applied to the Student t test when appropriate.

Other comments:
1. The use of the words 'aged' and 'old' should be changed throughout since they usually refer to mice above the age of 18 months.
2. In Fig 2H, the CXCR4 data could be presented as gMFI data since there is a global shift in staining of the Tfh and Tph populations.
3. Data from Fig 3D should be quantified.
4. How can a two-tailed Mann-Whitney U test provide significant results for Suppl Fig 1F with 4 mice per group.
5. Fig 4G: the proportion of reads rather than the actual number of reads may convey more insightful information.
6. Suppl Fig 4 legend: (B) and (D) are mislabeled.
7. Fig 5E and F: is it not 'NRP1+PD-1+' rather than 'NRP1+PD-1-' ?
8. Spelling mistake on the label of the Y axis of Suppl Fig 5A.
9. Fig 6G legend is under Fig 6F.
10. Page 13 line 8: the 'loss of body weight' is not evident from Suppl Fig 6F.
11. In the blood of SLE patients what is the expression level of Neuropilin-1 on TFH CD4 T cell? On CD8 T cells?
12. Page 15 line 26: the wording 'necessary' seems a bit strong given the presented evidence. Please tone down.

Referee #2 (Comments on Novelty/Model System for Author):

Considering that SLE is an autoimmune disease with a strong female bias (female to male ratio of 9:1), I am not sure it is appropriate to use a mouse model where pathogenic self responses predominantly occur in male mice.

Referee #2 (Remarks for Author):

The manuscript by Raveney el at uses a mouse model of SLE with a T cell specific deletion of neuropilin to demonstrate the resistance of these mice to SLE. They examine the phenotype of Tfh, autoantibody secretion, CDR3 sequences and other functional experiments to demonstrate that NRP1 is a marker of autoantigen-specific T cells. The manuscript in general is well written and contains a wealth of data examining the role of NRP1 on T cells in autoimmunity pathogenesis. However, in this reviewer's opinion, the main claim of the paper - NRP1 distinguishes self-reactive T helper cells- is not demonstrated, neither in the mouse model nor using human samples.
1. What is the specific reactivity of NRP1+ T helper cells in their model? Can the authors find a model where, knowing the autoantigen, they can demonstrate that only NRP1+ T helper cells are autoreactive?
2. Interestingly enough, NRP deletion on T cells (which includes Tregs) does lead to less autoimmunity. However, NRP1 expression on Tregs has been shown to be important for Treg stability in other diseases (cancer mouse models). What is the phenotype and function of these NRP1 KO Tregs in the model? How do the authors reconcile these two observations?
3. The authors focus on the role of NRP1 on Tfh, Tph, with a strong underlying rationale. But what about other T helper populations? If NRP really marks autoantigen-specific T helper cells, we would expect to see other Th that are NRP+ and reactive to self-antigens.
4. What is the role of NRP1 on these self-antigen specific T cells? What is the mechanism by which NRP silencing leads to resistance to autoimmunity? Is NRP involved in Tfh-B cell interactions? Do B cells express any of NRP1 ligands? Is NRP1 involved in self-reactive T cell viability/proliferation/survival? Inclusion of mechanistic data on this regard would clearly improve the current manuscript.

Referee #3 (Comments on Novelty/Model System for Author):

Technical quality: there is a lack of information regarding animal gender. Statistical tests should be justified

Novelty is high as it is the description of of potential specific marker of autoreactive Th cells

Medical impact could be high as it could provide a therapeutical target, or at least a very meaningfull biomarker to monitor a therapeutic effects of current treatments

Referee #3 (Remarks for Author):

Raveney et al describe the identification of autoreactive-Th specific markers in mouse models as well as in SLE patients. Using the BXSB strain (as well as Mrl-Lpr strain), whose males develop an SLE-like pathology, the authors showed that animals deficient for the Nr4a2 gene specifically in Th lymphocytes are protected from the pathology. In particular, the authors show that male BXSB mice display an expansion of various Th subgroups, and that this expansion is Nr4a2-dependent. On the other hand, the ablation of the Nr4a2 gene does not affect the response to exogenous antigens (NP-KLH). The co-expression of Nrp1 and Pd1 identifies a self-reactive population of Th cells that is dependent on Nr4a2. The transfer of this population into young BSXB mice (pre-disease) or into NR4A2-Th-deficient animals is necessary and sufficient to induce the pathology. A therapeutic intervention targeting Nrp1 improves systemic autoimmunity in BXSB and MRL-lpr mice. Finally, the authors show that Th expressing NRP1 are increased in lupus patients in the active phase of their disease.

This is an nice piece of work identifying a potentially usefull markers of autoreactive Th cells.

General comments:
It is quite disturbing to name the male BSXB mice: "Control". Control usually refers to healthy animals. Using BXSB and BXSB-Nr4a2cKO, would be easier to follow.

Genes names should appear in italic. Since they are mouse genes, only the first letter should be capital i.e Nr4a2. It is particularly important to respect these rules as there are both mice and humans studies in this work.

Specific Comments:

Figure 1A: Although it is probably spleens from male mice that are shown, it is not explicitely written neither in the figure legend nor in the text. It could be of interest to show both male and female spleens. Adding a scale on the y axis would be also usefull.

Figure 1G: It is too small and it would deserve arrows to point the abnormal structures. This would be of great help for non-specialist readers.

Figure 2 D-E-F: To explain the lack of correlation regarding the proportion of Tfh cells in the blood and the spleen, the authors argued that the Cxcr5 expression might targets the Tfh cells to the spleen. However, there is a huge increase of Tfh cells in the blood in 20 and 25weeks old animals. How do the authors explain this apparent discrepancy ?

Figure 4E: The authors compared the proportion of Nrp1+ CD4+ T cells in several autoimune backgrounds such as Aire or Fas deficient animals. Did the authors compared male animals in all groups? MRL-lpr mice develop severe early-onset autoimmune lymphorproliferation. Comparing with MRM-wt animals would be important as these animals also develop autoimmunity though at later age. Also, MRL-lpr mice exhibit high proportion of CD4-CD8- T cells (called double negative). It would be particularly important to assess the Nrp1 expression on this subset as it is believed to be autoreactive T cells accumulating as the consequence of the Fas deficiency. Lastly, the animal numbers are quite variable between the groups. Is the statistical test used really appropriate ?

In the transfer experiments (Figure 5) the donor cells are probaby coming from male mice. Are the recipient male mice as well? Globally there is a lack of information regarding the animal gender in most figures.

Figure 7 (SLE patients): the proportion of NRP1+ cells is quite variable in the patients. As 6 patients are not treated at the time of sampling , is it possible that the treated patients exhibit a lower proportion of NRP1+ cells? It could be probably more accurate to split the patients group in treated and untreated subgroups.

Referee #1

In this work, Raveney et al. used animal models (BXSB, MRL lpr/lpr) of systemic autoimmune diseases to identify a subset of CD4 helper T cells that seems crucial in driving B cells to produce characteristic autoantibodies. These CD4 T cells are dependent on the transcription factor NR4A2 and express at their cell surface both PD-1 and Neuropilin-1. The autoreactivity of these CD4 T cells is suspected based on molecular and functional features. Importantly, targeting Neuropilin-1 in vivo prevented the development of systemic autoimmune diseases in mice. Importantly, the authors went on to assess whether expression of Neuropilin-1 was increased on CD4 T cells from patients with systemic lupus erythematosus, and this turned out indeed to be the case

The study is well designed, and carefully executed. The results are fully novel and may well lead to the identification of a therapeutic target for patients with systemic autoimmune diseases. The manuscript is clearly written. There are, however, several important issues that need to be clarified by both text changes and complementary data

Important issues to be addressed:

1. From the data presented it is not fully clear that Neuropilin-1, rather than PD-1 or the combination of both, characterizes the autoreactive CD4 T cells. So far, the conclusion is based on indirect evidence such as TCR CDR3β sequences. To further explore this question the authors should perform adoptive transfer experiments with PD-1+ Neuropilin-1-negative vs. PD-1+ Neuropilin-1-positive CD4 T cells. Both populations can be clearly separated in MRL-Lpr mice (Suppl Fig 6I). Alternatively, the authors could sort the CD4 T cells based on PD-1 and Neuropilin-1 expression and assess ex vivo their self-reactive potential.

The reviewer asks an important question about PD-1 as a marker of pathogenic Th cells versus NRP1. Previously, PD1+ Th cells, either TfH or Tph, have been linked to pathogenesis in SLE and its animal models. As NRP1 in non-Treg cells is expressed by PD-1+ Th cells, it is conceivable that our finding merely underlines the previous concept of the crucial importance of PD-1+ T cells. Although there are few NRP1-PD-1+ Th cells in BXSB.Yaa mice, indicating a role for them is less likely, the reviewer suggests analysis of NRP1-PD-1+ Th cells in MRL-LPR mice to confirm this.

Unfortunately, we are unable to conduct the experiments suggested by the reviewer directly. The MRL.LPR mouse is not available with a Foxp3 reporter and so we are unable to sort populations of Foxp3-NRP1-PD-1+ Th cells for transfer. Additionally, direct self-reactive *in vitro* experiments are complex in these SLE models, where the exact self-antigens are unknown.

We must emphasize that it is targeting of NRP1 that led to reduced disease whereas targeting PD-1 directly to treat disease in BXSB is not necessarily successful (delivering inhibitory signal via PD-1 receptor alone was not sufficient to prevent disease [1] and we also were unsuccessful in treating disease in BXSB.Yaa mice with anti-PD-1 antibodies).

We have added new data that indicates that in another model of autoimmune disease, EAE, NRP1 expression occurs independently of PD-1 (**New data: Fig. EV4I**). As the antigen-specificity is well-understood in EAE, we were able to show that these Th1/Th17 NRP1+PD-1- Th cells infiltrating the CNS are specific for self-antigen. These

new data provide additional indirect evidence that NRP1 may be more important than PD-1 in identifying self-reactive pathogenic Th cells.
In addition, we have added more information regarding the NRP1-PD-1+ Th cell subset using BXSB.Yaa mice (NRP1-PD-1+ Th cells ca be found in BXSB.Yaa diseased mice as well as NRP1+PD-1+ Th cells, albeit at lower levels than MRL.LPR). NRP1-PD-1+ Th cells do not correlate with disease measures to the same extent as NRP1+ Th cells (**New data Fig. S2B**) and these cells remain constant or decrease in later stages, whilst NRP1+ Th cells continue to accumulate (**New data Fig. S1A**). Together, these data suggest the importance of NRP1+ Th cells over NRP1-PD-1+ Th cells in systemic autoimmune disease.

2. The RPA peptide is said to target Neuropilin-1-expressing cells and promote an apoptotic process. It is unclear why the frequency of Neuropilin-1-expressing conventional CD4 T cells is only partly reduced and why Neuropilin-1-expressing Foxp3 regulatory CD4 T cells are fully spared. A discussion on the cell types expressing Neuropilin-1 and the consequences of their targeting by the RPA peptide in warranted/
Although less relevant for human studies, due to the lack of NRP1 expression on Treg cells, the reviewer raises an important point about why our NRP1—targeting treatment mostly affects only non-Treg and not NRP1+ T regs cells. We have conducted further experiments to understand this serendipitous finding which we have added (**New data Appendix Fig. S3E**).
We did not observe a difference in NRP1 expression level between NRP1+ conventional Th cells and Tregs. Therefore we, investigated if these cells had different sensitivity to apoptosis induction. It has been previous reported for Tregs have differential susceptibility to particular apoptosis pathways, for example via BIM/BCL-2 [2].
In our system, we use the NRP1 receptor to target the pro-apoptotic KLAKLAK-KLAKLAK peptide sequence via a NRP1-binding sequence that delivers the payload into the cell. There, this peptide interferes with mitochondrial membrane potential leading to apoptosis [3, 4]. To test this hypothesis, we investigated the effect of our RPA peptide in vitro on populations of NRP1+ Th cells, both Treg and Foxp3-. Preliminary findings suggest that Foxp3+ Treg cells are somewhat resistant to this apoptosis induction compared with Foxp3-NRP1+ (**New data Appendix Fig. S3E**), but future work will be required to understand this mechanism more deeply.

3. The statistical analyses need to be reassessed, possibly with the help of statistical expertise.
We thank the reviewer for his/her careful reading of the manuscript and highlighting these issues. We have added missing descriptions and details to legends, and revised testing where it could be argued that univariate testing was misapplied. We have added more information to the statistics section in the methods, specifically explain our approach to determining distribution.

Specifically, it is unclear why one-tailed tests were performed;
Much of the early part of the manuscript results from our hypothesis that NR4A2 deletion (a nuclear receptor previously associated with IL-21-dependent autoimmune responses) would reduce systemic autoimmune disease in BXSB.Yaa mice. Therefore,

when testing this hypothesis against measures clearly associated with autoimmunity e.g. splenomegaly, we used one tailed tests using the hypothesis Nr4a2 deletion reduces disease compared with the null hypothesis that there is no disease reduction. In cases where the relationship between the measure and autoimmune disease was ambiguous, e.g. MZ B cells, two tailed tests were used. Similarly, as NRP1+ Th cells were associated with disease, for the treatment experiments targeting NRP1, the hypothesis was that this would reduce disease, thus we argue that one tailed testing is the right approach.

whether corrections for multiple comparisons were systematically applied, why Mann-Whitney test was used when comparing more than 2 groups,

Mann-Whitney U tests were used to compare 2 groups with data sets that failed normality testing. We applied such tests to make independent comparisons only, but within plot or within experiments. Correcting for multiple comparisons can be a thorny issue in statistical testing. Some experts statisticians argue that it is never useful [5], or even that commonly used hypothesis testing isn't useful for stating confidence intervals. However, in medical science studies, addressing the multiple comparison problem leading to type I errors is usually expected. Although attempting to correct complementary dataset with MCT will in fact likely to lead to type II errors and grouping datasets into one plot or panel doesn't necessarily imply that the datasets constitute multiple comparisons.

For many of the multiple timepoint measures in this study, it is questionable whether such data sets should be regarded as repeated measures (these are terminal datapoints using separate individual animals) requiring MCT or if they should be treated as complimentary independent data sets using a univariate analysis. For example, comparing pairs of genotypes at each timepoint where correcting for type I errors by MCT could instead introduce type II errors, as these data are supportive of each other. However, we agree that using a conventional approach is likely to be more helpful to allow translational scientists an appreciation the confidence in any differences in data.

Although multiple comparisons using false discovery rate possibly have the most power [6], generally manuscripts such as ours are published using ANOVA testing with post hoc multiple comparisons tests [7]. Thus, in light of the reviewer's comments, I have revisited the statistical testing and extended multiple comparison tests where appropriate. In some cases, p values increased or decreased and the statistical testing information has been updated in legends and figures to reflect this.

and has a correction for unequal variance been applied to the Student t test when appropriate.

We used Welch's correction to t-tests where data were normally distributed, but initial testing indicated unequal variance between the two data sets (by comparing if SDs ratios were close and confirmed by an F test). We have corrected the legends where this information was missing.

We thank the reviewer for his/her detailed suggestions and discussion of our statistical shortcomings and we hope that her/his concerns are satisfied by our new analysis and additional details.

Other comments:
1. The use of the words 'aged' and 'old' should be changed throughout since they usually refer to mice above the age of 18 months.
In light of this reviewer's comments and also comments from reviewer 3, we have amended the text to use more precise naming in all cases and have avoided the use of aged.

2. In Fig 2H, the CXCR4 data could be presented as gMFI data since there is a global shift in staining of the Tfh and Tph populations.
We agree that MFI is a fairer measure for this shift and have changed this figure to this. We thank the reviewer for his/her careful analysis.

3. Data from Fig 3D should be quantified
We thank the reviewer for indicating that this figure is lacking clarity. Numeration of germinal centers is unlikely to be helpful for these enlarged sections, therefore we have added additional plots as Appendix Fig. S1 showing these staining from the whole spleen. We have added text to clarify of the structures to be compared by this assay and we hope that the reviewer finds this solution to be more meaningful and help the reader to understand this comparison.

4. How can a two-tailed Mann-Whitney U test provide significant results for Suppl Fig 1F with 4 mice per group.
We thank the reviewer for his/her careful reading. This typographic error mislabelled a two tailed test together with this one figure as a one tailed test. As above we have clarified all statistical methods and revised where appropriate, but in this case owing to the reviewer's concerns, we have swapped in new data using a larger group of mice. These new data now tested as normally distributed, so a t-test was substituted.

5. Fig 4G: the proportion of reads rather than the actual number of reads may convey more insightful information.
Fig. EV4D (previously Fig. 4G) describing self-reactivity promoting TcR sequences in NRP1 subsets is now given as proportion of total reads.

6. Suppl Fig 4 legend: (B) and (D) are mislabeled.
Figure legends have been revised

7. Fig 5E and F: is it not 'NRP1+PD-1+' rather than 'NRP1+PD-1-' ?
This typo has been corrected (Now Fig. 6E&F)

8. Spelling mistake on the label of the Y axis of Suppl Fig 5A.
This typo has been corrected (Now Fig. EV5A)

9. Fig 6G legend is under Fig 6F
This has been corrected (Now Fig. 7G).

10. Page 13 line 8: the 'loss of body weight' is not evident from Suppl Fig 6F
Loss of body weight or reduced weight gain over development time is a standard feature of MRL.LRP mice as compared with MRL background mice that only get very

mild disease. For our purposes, we ended the experiments at early timepoint to reduce potential animal suffering and so didn't reach significant weight loss stage, so it is more correctly expressed as “less weight gain than would occur if healthy” with these large mice, thus preventing this process leads to increased body weight in the RPA-treated mice versus the control mice. We have corrected this sentence in the text and added a reference to make this clearer.

11. In the blood of SLE patients what is the expression level of Neuropilin-1 on TFH CD4 T cell? On CD8 T cells?

In human CD8 T cells, there were few NRP1+ cells with the exception of 1 SLE patient (Letter Figure 1)

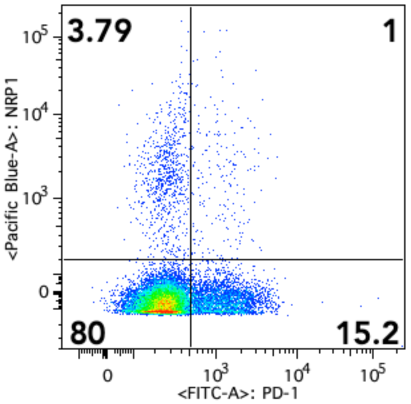

Letter Figure 1
Left: staining of CD8 T cells in PBMC from a SLE subject with high NRP1+ CD8 T cells stained for PD-1 and NRP1 by flow cytometry
Right, summary percentages for SLE and healthy control CD8 T cell NRP1 expression

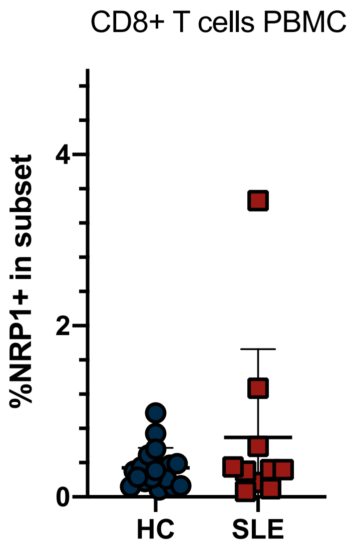

Unfortunately, we do not have staining data available for Tfh markers in human PBMC for NRP1+ Th cells as only limited sample was available and sample collection took many months.

12. Page 15 line 26: the wording 'necessary' seems a bit strong given the presented evidence. Please tone down.

We have corrected this.

Referee #2 (Comments on Novelty/Model System for Author):
Considering that SLE is an autoimmune disease with a strong female bias (female to male ratio of 9:1), I am not sure it is appropriate to use a mouse model where pathogenic self responses predominantly occur in male mice.

We understand the reviewers concerns that although SLE does occur in males, female patients predominate, therefore it might seem counter intuitive to use BXSB mice where SLE-like disease occurs more aggressively in male mice. The reason that male mice have accelerated disease on this autoimmune prone background is not due to sex-related differences in immune responses, rather that the autoimmune accelerator mutation (aa) that includes duplication of immune genes from other chromosomes happens to incorporate into the Y chromosome (Yaa). Female mice also develop SLE-like disease, but much more slowly. Thus we do not regard BXSB.Yaa SLE-like disease as a male autoimmune disease *per se* [8, 9], but an autoimmune diseases that happens to occur in male mice independent of other sex factors. No mouse model is perfect, but BXSB.Yaa mice do recapitulate a number of features of human SLE and so despite the sex flaw, they can still be useful: BXSB.Yaa mice have been successfully used over many years to provide insight into human SLE and are regarded in the field as a standard model [10-12]. It is also worth pointing out that we also confirmed some of our main findings in another SLE-like mouse model, MRL-LPR, and these studies used female mice. We have added additional discussion to the introduction to help readers appreciate the apparent conflict in gender for this model.

Referee #2 (Remarks for Author):
The manuscript by Raveney el at uses a mouse model of SLE with a T cell specific deletion of neuropilin to demonstrate the resistance of these mice to SLE. They examine the phenotype of Tfh, autoantibody secretion, CDR3 sequences and other functional experiments to demonstrate that NRP1 is a marker of autoantigen-specific T cells. The manuscript in general is well written and contains a wealth of data examining the role of NRP1 on T cells in autoimmunity pathogenesis. However, in this reviewer's opinion, the main claim of the paper - NRP1 distinguishes self-reactive T helper cells- is not demonstrated, neither in the mouse model nor using human samples.

We thank the reviewer for his/her kind praise of our works. However, we feel that there has been a misunderstanding regarding our title - NRP1 distinguishes self-reactive T helper cells. We do not mean to imply that our works show that this is a universal marker of ALL self-reactive cells. We will revise the title for clarity and add further discussion to indicate that more work is required to link these cells in other disease contexts and that the results do not rule out other non-NRP1+ Th cells populations in SLE and other autoimmune diseases. We understand that the reviewer has concerns about the specificity of these cells to fully demonstrate this claim. However, the main thrust of the manuscript is that we have shown that NRP1+ Th cells increase in correlation with disease serving as indicators, NRP1 can be used to isolate self-reactive pathogenic Th cells, and that targeting NRP1+ Th cells reduces autoimmune responses, all together these data certainly support of claim that NRP1 expression can be used to indicate population of pathogenic self-reactive Th cells.

We appreciate the reviewer's position that the addition of information about the reactivity of this cell subset is desirable to more fully demonstrate the claim. We have conducted additional experiments and added new data (Fig. EV4H-J) in response to the reviewer's specific points below, which we believe greatly enhances the manuscript, further supporting our claim and extending the relevance of NRP1 expression to other autoimmune contexts. We hope these revisions satisfy the reviewer's concerns and will allow publication, which will seed future research into this potentially important marker in other autoimmune contexts with clearer antigen-specificity.

1. What is the specific reactivity of NRP1+ T helper cells in their model? Can the authors find a model where, knowing the autoantigen, they can demonstrate that only NRP1+ T helper cells are autoreactive?

Whilst we consider the wide range of antigens involved in BXSB.Yaa mouse model of SLE to be an advantage to study more physiologic conditions by which self-specific Th cells generate autoimmune disease, we recognise the lack of knowledge of a specific antigen in each mouse to limit the extent of this study. We appreciate that to fully support a claim that these cells are self-reactive, indirect measures such as TcR CDR3 contact residues and anergy markers, as well as that these cells cause increased pathology on transfer are not sufficient.
Therefore, as suggested we have turned to a mouse model that we are familiar with and has a known driving autoantigen – EAE induced by MOG35-55 immunisation of C57BL/6 mice.
We find induction of EAE, the mouse model of autoimmune neuroinflammatory disease, led to infiltration of CNS tissues with Th cells expressing NRP1. These NRP1+Foxp3- Th cells were also self-antigen specific as they responded to stimulation by the self-antigen to drive production of the cytokines IL-17 and IFN-g, which are associated with pathogenic mechanisms in EAE (**New data Fig. EV4H-J**). These cells were PD-1- and in this disease does not involve B cell:Tfh cell interactions, suggesting NRP1 association with self-reactive Th cells may apply more widely than PD-1+ Th cells in systemic autoimmune disease.
We thank the reviewer for this suggestion and although these preliminary data are supportive of the findings in the current study that NRP1+ Th cells are self reactive, we hope that building on this will generate future interesting findings.

2. Interestingly enough, NRP deletion on T cells (which includes Tregs) does lead to less autoimmunity. However, NRP1 expression on Tregs has been shown to be important for Treg stability in other diseases (cancer mouse models). What is the phenotype and function of these NRP1 KO Tregs in the model? How do the authors reconcile these two observations?

The biology of NRP1 is potentially complex and heterogenous. NRP1 acts as a receptor for a number of different ligands and could have differential functions in different contexts. These different pathways can explain how NRP1 has seemingly conflicting roles.
In the cases of Treg, it has been suggested that NRP1 binding of VEGF could allow entry of Tregs into the tumor region and suppress anti-tumor immune responses[13].

Thus, blocking NRP1 decreased Treg activity and enhances anti-tumor immunity. For its role in maintenance and stability of mouse Treg, it has been suggested that a different NRP1 binding partner, SEMA 4a is involved[14]. Another suggested function for NRP1 on Tregs is enhancing binding and cell-cell contact with other immune cells, for example Tregs with DCs [15].
The NRP1 KO is not available on the BXSB.Yaa background, so it is unclear of how NRP1 KO Tregs operate in this model. The lack of NRP1 expression on human Tregs demonstrates that these Tregs at least can function normally in the absence of NRP1 and it is an intriguing question how the biology of these Tregs in humans differ from mouse. However, we hope the publication of our works will inspire future studies to understand NRP1 biology in the context of self-reactive pathogenic Th cells as well as self-reactive Tregs.

3. The authors focus on the role of NRP1 on Tfh, Tph, with a strong underlying rationale. But what about other T helper populations? If NRP really marks autoantigen-specific T helper cells, we would expect to see other Th that are NRP+ and reactive to self-antigens.

The reviewer makes an important point about a potential of more universal nature of NRP1 expression on other populations of Th cells. Indeed, we do observe some non-Treg Th cells expressing NRP1 outside of autoimmune contexts (B6 mice Fig. EV4A (previously SFig. 4) and also, the small populations to NRP1+ Th cells in human subjects – including healthy controls that are PD-1 negative (Letter Figure 2).

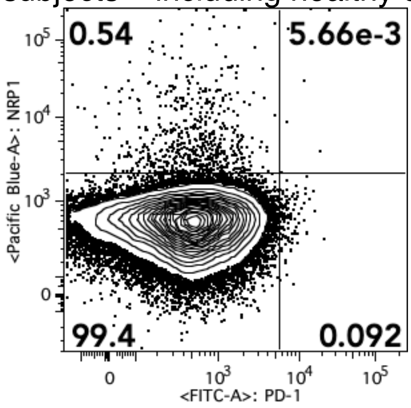

Letter Figure 2
Representative staining of CD3+CD4+ PBMC from a healthy subject stained for PD-1 and NRP1 by flow cytometry

However, these are relatively small numbers as might be expected of autoreactive Th cells in the steady state. But it is conceivable that these are also self-reactive cells of unknown specificity that accumulate with age. Furthermore, from our answer to point 2 above, we have now investigated NRP1 expression in other, non-systemic, autoimmune models that do not rely on Tfh/Tph supplying B cells help. In these cases, NRP1+ Th cells can elaborate IFN-g/IL-17 in response to stimulation with self-antigen (**New Data Fig. 5H&J**) and do not express PD-1 (**New data Fig. 5G**). So the reviewer's prediction is correct that NRP1+ is not tied to TfH/TpH in systemic autoimmune disease, but it also can be linked to self-reactive Th1/Th17 cells.

4. What is the role of NRP1 on these self-antigen specific T cells? What is the mechanism by which NRP silencing leads to resistance to autoimmunity? Is NRP involved in Tfh-B cell interactions? Do B cells express any of NRP1 ligands? Is NRP1 involved in self-reactive T cell viability/proliferation/survival? Inclusion of mechanistic data on this regard would clearly improve the current manuscript.

NRP1 has a wide range of possible ligands and mechanisms [16]. It is possible that NRP1 on Th cells could function to assist T:B interactions. NRP1 does function in T:DC interactions via the MAM domain [17]. Other ligands for NRP1 could also be involved – the CUB domains interact with Semaphorins and Plexin [18, 19]. For example, the semaphorin SEMA 3A is widely expressed on both B cells and Th cells in BXSB.Yaa mice (Letter Figure 3).

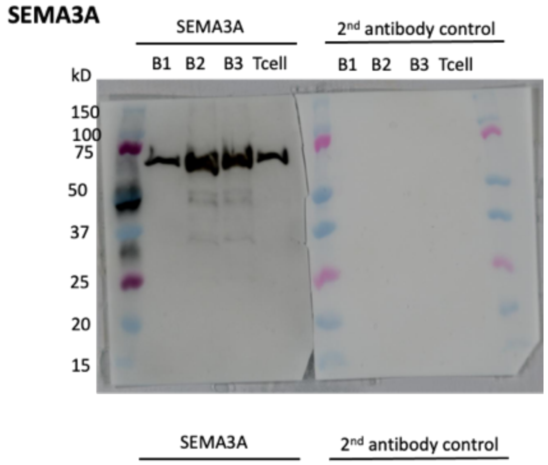

Letter Figure 3
CD19+ Splenic B cells were sorted from male BXSB.Yaa mice after disease onset in 3 different experiments (B1,2, 3) as well as Th cells from experiment 3. Proteins were purified from these cells and SEM3A was measured by Western Blotting

NRP1 binds to VEGF influencing T cell trafficking [13] and it can act as a co-receptor for TGFβ [20] and NRP1 can also initiate direct downstream signalling [21].
We agree that the mechanism by which NRP1 on non-Tregs is an exciting subject and have added additional discussion on the possible mechanisms of NRP1 to the manuscript. With such potential wide roles for NRP1 in immunity in different contexts, we hope that the function of NRP1 on pathogenic Th cells can be further addressed by future studies. Additionally, the recent interest in NRP1 that has been prompted by its potential role in SARS-CoV2 entry to cells [22] , which has sparked many new works investigating new potential functions of this molecule in immune interactions.

Referee #3 (Comments on Novelty/Model System for Author):
Technical quality: there is a lack of information regarding animal gender. Statistical tests should be justified
Novelty is high as it is the description of of potential specific marker of autoreactive Th cells
Medical impact could be high as it could provide a therapeutical target, or at least a very meaningfull biomarker to monitor a therapeutic effects of current treatments
We understand the reviewer's comments about gender here and below in specific questions, as well as suggestions for the naming of strains/control samples in improving clarity. To resolve this, we have renamed samples in full with strain name and sex. Please see our answer to reviewer 1 for statistical test justification. We thank the reviewer for her/his helpful suggestions to improve the manuscript quality and readability.

Referee #3 (Remarks for Author):
Raveney et al describe the identification of autoreactive-Th specific markers in mouse models as well as in SLE patients. Using the BXSB strain (as well as Mrl-Lpr strain), whose males develop an SLE-like pathology, the authors showed that animals deficient for the Nr4a2 gene specifically in Th lymphocytes are protected from the pathology. In particular, the authors show that male BXSB mice display an expansion of various Th subgroups, and that this expansion is Nr4a2-dependent. On the other hand, the ablation of the Nr4a2 gene does not affect the response to exogenous antigens (NP-KLH). The co-expression of Nrp1 and Pd1 identifies a self-reactive population of Th cells that is dependent on Nr4a2. The transfer of this population into young BSXB mice (pre-disease) or into NR4A2-Th-deficient animals is necessary and sufficient to induce the pathology. A therapeutic intervention targeting Nrp1 improves systemic autoimmunity in BXSB and MRL-lpr mice. Finally, the authors show that Th expressing NRP1 are increased in lupus patients in the active phase of their disease. This is an nice piece of work identifying a potentially usefull markers of autoreactive Th cells.

General comments:
It is quite disturbing to name the male BSXB mice: "Control". Control usually refers to healthy animals. Using BXSB and BXSB-Nr4a2cKO, would be easier to follow.
We appreciate the problem here and accept that our attempts at simplification have led to a reduction of clarity. We have changed our naming system throughout the manuscript to cover this, now only using the full strain/sex names.

Genes names should appear in italic. Since they are mouse genes, only the first letter should be capital i.e Nr4a2. It is particularly important to respect these rules as there are both mice and humans studies in this work.
We have made these changes to the text as requested.

Specific Comments:
Figure 1A: Although it is probably spleens from male mice that are shown, it is not explicitely written neither in the figure legend nor in the text. It could be of interest to show both male and female spleens. Adding a scale on the y axis would be also usefull.

Female spleens have now been added (**New data, Fig. 1C**) and a y-axis scale has been added to Fig. 1A and 1C.

Figure 1G: It is too small and it would deserve arrows to point the abnormal structures. This would be of great help for non-specialist readers.
We have made these changes and added explanation of the arrows for each stain to the legend.

Figure 2 D-E-F: To explain the lack of correlation regarding the proportion of Tfh cells in the blood and the spleen, the authors argued that the Cxcr5 expression might targets the Tfh cells to the spleen. However, there is a huge increase of Tfh cells in the blood in 20 and 25weeks old animals. How do the authors explain this apparent discrepancy ?
$CXCR5^{+}$ Th cells in blood have been termed Tfh-like cells or circulating Tfh (cTfh), these have attracted study in recent years. Differences between these cells and splenic classical Tfh have been proposed in terms of phenotype and activity.
In human SLE disease, expansion of cTfh is observed as a late feature in established disease correlating with high activity or related to severe disease [23-25]. A study suggests that these cells are generated in local lymphoid compartments before release into the blood due to immune activation [26]. Another study indicates that cTfh originates in lymph node germinal centers [27]. It is possible in our model that these cells have different source to Tph and represent more advance disease associated with tissue damage leading to increased antigen shedding to draining lymph nodes and further T cell activation and differentiation. Another possibility is that these cells could escape from the spleen during splenomegaly process with splenic inflammation leading to more disorganisation and potentially immune cell leakage to vasculature changes, suggesting that these convert in the spleen following splenic hyperplasia. Although, we have no direct data to confirm this on our model.

Figure 4E: The authors compared the proportion of Nrp1+ CD4+ T cells in several autoimune backgrounds such as Aire or Fas deficient animals. Did the authors compared male animals in all groups? MRL-lpr mice develop severe early-onset autoimmune lymphorproliferation. Comparing with MRM-wt animals would be important as these animals also develop autoimmunity though at later age. Also, MRL-lpr mice exhibit high proportion of CD4-CD8- T cells (called double negative). It would be particularly important to assess the Nrp1 expression on this subset as it is believed to be autoreactive T cells accumulating as the consequence of the Fas deficiency. Lastly, the animal numbers are quite variable between the groups. Is the statistical test used really appropriate ?
The reviewer raises important points about controls for the MRL.LPR mice and potentially extending the data by investigating older MRL mice which develop mild autoimmune disease. We have now added new data for MRL mice and extended the previous Fig. 4E. MRL mice had considerably lower proportions of NRP1 conventional Th cells and we did indeed find slight increases in NRP1+ Th cells in MRL mice 20-20 weeks versus 6-8 weeks. In reformatting the figures for the Journal EV and Appendix requirements, we have restructured this part of the figure (Now Fig 5G) making these data clearer and given them greater prominence in the text.

We thank the reviewer for the suggestion to consider CD4^{-}CD8^{-} T cells in MRL.LPR mice. We have now analysed the flow cytometry data and added these data (**New data: Fig. EV4G**). Interestingly, whilst we do observe increased numbers of CD4^{-}CD8^{-} T cells and these do mostly express PD-1, only a little NRP1 increase is seen in this population.
We have conducted further experiments and added additional samples to groups to giving increased n numbers and enable more similar numbers per group in the extended figure (**New data: Fig. 5E-G**).

In the transfer experiments (Figure 5) the donor cells are probaby coming from male mice. Are the recipient male mice as well? Globally there is a lack of information regarding the animal gender in most figures.
In case of disease in BXSB.Yaa mice, males were used. But we understand the likely confusion of gender issues in this study as we also used non-diseased females as controls and, in other models, female mice were used. In view of this reviewer's and reviewer 2's comments, we have tried to be explicit about gender in all cases and have added more comprehensive labelling of mouse gender in the text and legends through the manuscript.

Figure 7 (SLE patients): the proportion of NRP1+ cells is quite variable in the patients. As 6 patients are not treated at the time of sampling , is it possible that the treated patients exhibit a lower proportion of NRP1+ cells? It could be probably more accurate to split the patients group in treated and untreated subgroups.
As shown Fig. S3E (previously Supp, Fig. 7E), the proportion of NRP+ cells is somewhat lower in treated patients, but this difference was not statistically significant. We have made this finding clearer in the text. Unfortunately, we do not have staining data available for Tfh markers in human PBMC for NRP1+ Th cells.

References:

1. Ding, H., et al., *Delivering PD-1 inhibitory signal concomitant with blocking ICOS co-stimulation suppresses lupus-like syndrome in autoimmune BXSB mice.* Clin Immunol, 2006. **118**(2-3): p. 258-67.
2. Tischner, D., et al., *Defective cell death signalling along the Bcl-2 regulated apoptosis pathway compromises Treg cell development and limits their functionality in mice.* J Autoimmun, 2012. **38**(1): p. 59-69.
3. Ellerby, H.M., et al., *Anti-cancer activity of targeted pro-apoptotic peptides.* Nat Med, 1999. **5**(9): p. 1032-8.
4. Sugahara, K.N., et al., *Coadministration of a tumor-penetrating peptide enhances the efficacy of cancer drugs.* Science, 2010. **328**(5981): p. 1031-5.
5. Rothman, K.J., *No Adjustments Are Needed for Multiple Comparisons.* Epidemiology, 1990. **1**(1): p. 43-46.
6. Benjamini, Y. and Y. Hochberg, *Controlling the False Discovery Rate: A Practical and Powerful Approach to Multiple Testing.* Journal of the Royal Statistical Society: Series B (Methodological), 1995. **57**(1): p. 289-300.
7. McDonald, J.H., *Multiple Comparisons*, in *Handbook of Biological Statistics*. 2014, Sparky House Publishing, Baltimore, Maryland.

8. Eisenberg, R.A., et al., *Male determined accelerated autoimmune disease in BXSB mice: transfer by bone marrow and spleen cells.* J Immunol, 1980. **125**(3): p. 1032-6.
9. Eisenberg, R.A. and F.J. Dixon, *Effect of castration on male-determined acceleration of autoimmune disease in BXSB mice.* J Immunol, 1980. **125**(5): p. 1959-61.
10. Izui, S., et al., *Lessons from BXSB and related mouse models.* Int Rev Immunol, 2000. **19**(4-5): p. 447-72.
11. Kimura, J., et al., *BXSB-type genome causes murine autoimmune glomerulonephritis: pathological correlation between telomeric region of chromosome 1 and Yaa.* Genes Immun, 2014. **15**(3): p. 182-9.
12. McPhee, C.G., et al., *IL-21 is a double-edged sword in the systemic lupus erythematosus-like disease of BXSB.Yaa mice.* J Immunol, 2013. **191**(9): p. 4581-8.
13. Hansen, W., et al., *Neuropilin 1 deficiency on CD4+Foxp3+ regulatory T cells impairs mouse melanoma growth.* J Exp Med, 2012. **209**(11): p. 2001-16.
14. Delgoffe, G.M., et al., *Stability and function of regulatory T cells is maintained by a neuropilin-1-semaphorin-4a axis.* Nature, 2013. **501**(7466): p. 252-6.
15. Sarris, M., et al., *Neuropilin-1 expression on regulatory T cells enhances their interactions with dendritic cells during antigen recognition.* Immunity, 2008. **28**(3): p. 402-13.
16. Pellet-Many, C., et al., *Neuropilins: structure, function and role in disease.* Biochem J, 2008. **411**(2): p. 211-26.
17. Mizui, M. and H. Kikutani, *Neuropilin-1: the glue between regulatory T cells and dendritic cells?* Immunity, 2008. **28**(3): p. 302-3.
18. Lu, D., et al., *Architecture of the Sema3A/PlexinA4/Neuropilin tripartite complex.* Nat Commun, 2021. **12**(1): p. 3172.
19. Gu, C., et al., *Characterization of neuropilin-1 structural features that confer binding to semaphorin 3A and vascular endothelial growth factor 165.* J Biol Chem, 2002. **277**(20): p. 18069-76.
20. Glinka, Y., et al., *Neuropilin-1 exerts co-receptor function for TGF-beta-1 on the membrane of cancer cells and enhances responses to both latent and active TGF-beta.* Carcinogenesis, 2011. **32**(4): p. 613-21.
21. Valdembri, D., et al., *Neuropilin-1/GIPC1 signaling regulates alpha5beta1 integrin traffic and function in endothelial cells.* PLoS Biol, 2009. **7**(1): p. e25.
22. Daly, J.L., et al., *Neuropilin-1 is a host factor for SARS-CoV-2 infection.* Science, 2020.
23. Simpson, N., et al., *Expansion of circulating T cells resembling follicular helper T cells is a fixed phenotype that identifies a subset of severe systemic lupus erythematosus.* Arthritis Rheum, 2010. **62**(1): p. 234-44.
24. Kim, S.J., K. Lee, and B. Diamond, *Follicular Helper T Cells in Systemic Lupus Erythematosus.* Front Immunol, 2018. **9**: p. 1793.
25. Choi, J.Y., et al., *Circulating follicular helper-like T cells in systemic lupus erythematosus: association with disease activity.* Arthritis Rheumatol, 2015. **67**(4): p. 988-99.
26. Asrir, A., et al., *Interconnected subsets of memory follicular helper T cells have different effector functions.* Nat Commun, 2017. **8**(1): p. 847.
27. Vella, L.A., et al., *T follicular helper cells in human efferent lymph retain lymphoid characteristics.* J Clin Invest, 2019. **129**(8): p. 3185-3200.

5th Aug 2022

Dear Dr. Raveney,

Thank you for the submission of your revised manuscript to EMBO Molecular Medicine. I am pleased to inform you that we will be able to accept your manuscript pending the following final amendments:

1) Please address the referee #1 minor concerns.
2) Authorship: In our submission system Ben Raveney and Shinji Oki are indicated as corresponding authors, while in the manuscript only Shinji Oki is the corresponding author. Please clarify and correct either in the submission system or in the manuscript.
3) Figures: We noticed that Figure 8 seems to be distorted and the right side of the file seems cut. Please check and provide the correct figure?
4) In the main manuscript file, please do the following:
- Remove text highlight colour, also in the "Appendix".
- In M&M, please include statement that in addition to WMA Declaration of Helsinki the experiments also conformed the Department of Health and Human Services Belmont Report.
5) Author Contributions: CRediT has replaced the traditional author contributions section because it offers a systematic machine readable author contributions format that allows for more effective research assessment. You are encouraged to use the free text boxes beneath each contributing author's name to add specific details on the author's contribution. More information is available in our guide to authors.
6) Synopsis: Please submit the synopsis text as a separate .doc file. Check your synopsis text and image before submission with your revised manuscript. Please be aware that in the proof stage minor corrections only are allowed (e.g., typos).
7) For more information: There is space at the end of each article to list relevant web links for further consultation by our readers. Could you identify some relevant ones and provide such information as well? Some examples are patient associations, relevant databases, OMIM/proteins/genes links, author's websites, etc...
8) Source data: We encourage you to include the source data for figure panels that show essential data. Numerical data should be provided as individual .xls or .csv files (including a tab describing the data). For blots or microscopy, uncropped images should be submitted (using a zip archive if multiple images need to be supplied for one panel). For the main figures please upload one file per figure, while all EV figures should be zipped and uploaded as one file. Please check "Author Guidelines" for more information.
9) As part of the EMBO Publications transparent editorial process initiative (see our Editorial at http://embomolmed.embopress.org/content/2/9/329), EMBO Molecular Medicine will publish online a Review Process File (RPF) to accompany accepted manuscripts. This file will be published in conjunction with your paper and will include the anonymous referee reports, your point-by-point response and all pertinent correspondence relating to the manuscript. Let us know whether you agree with the publication of the RPF and as here, if you want to remove or not any figures from it prior to publication. Please note that the Authors checklist will be published at the end of the RPF.
10) Please provide a point-by-point letter INCLUDING my comments as well as the reviewer's reports and your detailed responses (as Word file).

I look forward to reading a new revised version of your manuscript as soon as possible.

Yours sincerely,

Zeljko Durdevic

.

***** Reviewer's comments *****

Referee #1 (Remarks for Author):

My earlier comments have been dealt with appropriately and I wish to congratulate the authors for their very nice work.

I would just raise two minor points that can be corrected on the very last version of the paper:

- MRL-LPR is sometimes mispealled (PRL) in the text and on Figures
- I would only use two-tailed statistical tests throughout the manuscript

Referee #2 (Comments on Novelty/Model System for Author):

No concerns on the above points

Referee #2 (Remarks for Author):

The authors have addressed all my comments.

CD8$^-$ T cells and these do mostly express PD-1, only a little NRP1 increase is seen in this population.
We have conducted further experiments and added additional samples to groups to giving increased n numbers and enable more similar numbers per group in the extended figure (**New data: Fig. 5E-G**).

In the transfer experiments (Figure 5) the donor cells are probaby coming from male mice. Are the recipient male mice as well? Globally there is a lack of information regarding the animal gender in most figures.
In case of disease in BXSB.Yaa mice, males were used. But we understand the likely confusion of gender issues in this study as we also used non-diseased females as controls and, in other models, female mice were used. In view of this reviewer's and reviewer 2's comments, we have tried to be explicit about gender in all cases and have added more comprehensive labelling of mouse gender in the text and legends through the manuscript.

Figure 7 (SLE patients): the proportion of NRP1+ cells is quite variable in the patients. As 6 patients are not treated at the time of sampling , is it possible that the treated patients exhibit a lower proportion of NRP1+ cells? It could be probably more accurate to split the patients group in treated and untreated subgroups.
As shown Fig. S3E (previously Supp, Fig. 7E), the proportion of NRP+ cells is somewhat lower in treated patients, but this difference was not statistically significant. We have made this finding clearer in the text. Unfortunately, we do not have staining data available for Tfh markers in human PBMC for NRP1+ Th cells.

References:

1. Ding, H., et al., *Delivering PD-1 inhibitory signal concomitant with blocking ICOS co-stimulation suppresses lupus-like syndrome in autoimmune BXSB mice.* Clin Immunol, 2006. **118**(2-3): p. 258-67.
2. Tischner, D., et al., *Defective cell death signalling along the Bcl-2 regulated apoptosis pathway compromises Treg cell development and limits their functionality in mice.* J Autoimmun, 2012. **38**(1): p. 59-69.
3. Ellerby, H.M., et al., *Anti-cancer activity of targeted pro-apoptotic peptides.* Nat Med, 1999. **5**(9): p. 1032-8.
4. Sugahara, K.N., et al., *Coadministration of a tumor-penetrating peptide enhances the efficacy of cancer drugs.* Science, 2010. **328**(5981): p. 1031-5.
5. Rothman, K.J., *No Adjustments Are Needed for Multiple Comparisons.* Epidemiology, 1990. **1**(1): p. 43-46.
6. Benjamini, Y. and Y. Hochberg, *Controlling the False Discovery Rate: A Practical and Powerful Approach to Multiple Testing.* Journal of the Royal Statistical Society: Series B (Methodological), 1995. **57**(1): p. 289-300.
7. McDonald, J.H., *Multiple Comparisons*, in *Handbook of Biological Statistics*. 2014, Sparky House Publishing, Baltimore, Maryland.
8. Eisenberg, R.A., et al., *Male determined accelerated autoimmune disease in BXSB mice: transfer by bone marrow and spleen cells.* J Immunol, 1980. **125**(3): p. 1032-6.
9. Eisenberg, R.A. and F.J. Dixon, *Effect of castration on male-determined acceleration of autoimmune disease in BXSB mice.* J Immunol, 1980. **125**(5): p. 1959-61.

10. Izui, S., et al., *Lessons from BXSB and related mouse models.* Int Rev Immunol, 2000. **19**(4-5): p. 447-72.
11. Kimura, J., et al., *BXSB-type genome causes murine autoimmune glomerulonephritis: pathological correlation between telomeric region of chromosome 1 and Yaa.* Genes Immun, 2014. **15**(3): p. 182-9.
12. McPhee, C.G., et al., *IL-21 is a double-edged sword in the systemic lupus erythematosus-like disease of BXSB.Yaa mice.* J Immunol, 2013. **191**(9): p. 4581-8.
13. Hansen, W., et al., *Neuropilin 1 deficiency on CD4+Foxp3+ regulatory T cells impairs mouse melanoma growth.* J Exp Med, 2012. **209**(11): p. 2001-16.
14. Delgoffe, G.M., et al., *Stability and function of regulatory T cells is maintained by a neuropilin-1-semaphorin-4a axis.* Nature, 2013. **501**(7466): p. 252-6.
15. Sarris, M., et al., *Neuropilin-1 expression on regulatory T cells enhances their interactions with dendritic cells during antigen recognition.* Immunity, 2008. **28**(3): p. 402-13.
16. Pellet-Many, C., et al., *Neuropilins: structure, function and role in disease.* Biochem J, 2008. **411**(2): p. 211-26.
17. Mizui, M. and H. Kikutani, *Neuropilin-1: the glue between regulatory T cells and dendritic cells?* Immunity, 2008. **28**(3): p. 302-3.
18. Lu, D., et al., *Architecture of the Sema3A/PlexinA4/Neuropilin tripartite complex.* Nat Commun, 2021. **12**(1): p. 3172.
19. Gu, C., et al., *Characterization of neuropilin-1 structural features that confer binding to semaphorin 3A and vascular endothelial growth factor 165.* J Biol Chem, 2002. **277**(20): p. 18069-76.
20. Glinka, Y., et al., *Neuropilin-1 exerts co-receptor function for TGF-beta-1 on the membrane of cancer cells and enhances responses to both latent and active TGF-beta.* Carcinogenesis, 2011. **32**(4): p. 613-21.
21. Valdembri, D., et al., *Neuropilin-1/GIPC1 signaling regulates alpha5beta1 integrin traffic and function in endothelial cells.* PLoS Biol, 2009. **7**(1): p. e25.
22. Daly, J.L., et al., *Neuropilin-1 is a host factor for SARS-CoV-2 infection.* Science, 2020.
23. Simpson, N., et al., *Expansion of circulating T cells resembling follicular helper T cells is a fixed phenotype that identifies a subset of severe systemic lupus erythematosus.* Arthritis Rheum, 2010. **62**(1): p. 234-44.
24. Kim, S.J., K. Lee, and B. Diamond, *Follicular Helper T Cells in Systemic Lupus Erythematosus.* Front Immunol, 2018. **9**: p. 1793.
25. Choi, J.Y., et al., *Circulating follicular helper-like T cells in systemic lupus erythematosus: association with disease activity.* Arthritis Rheumatol, 2015. **67**(4): p. 988-99.
26. Asrir, A., et al., *Interconnected subsets of memory follicular helper T cells have different effector functions.* Nat Commun, 2017. **8**(1): p. 847.
27. Vella, L.A., et al., *T follicular helper cells in human efferent lymph retain lymphoid characteristics.* J Clin Invest, 2019. **129**(8): p. 3185-3200.

***** Reviewer's comments ***** (Reviewer in red, author response in black)

Referee #1 (Remarks for Author):

My earlier comments have been dealt with appropriately and I wish to congratulate the authors for their very nice work.
We thank the reviewer for his/her kind comments. We greatly appreciate the reviewer's time in this process and genuinely believe that his/her comments and questions have improved this manuscript greatly.

I would just raise two minor points that can be corrected on the very last version of the paper:

- MRL-LPR is sometimes mispealled (PRL) in the text and on Figures
We apologize for these errors. Unfortunately, we have been unable to locate these typos – hopefully, if a mistake remains, it can be picked up by final proof-readers.

- I would only use two-tailed statistical tests throughout the manuscript
We have made these changes to Figs. 1B, 6F,7ABEF

Referee #2 (Comments on Novelty/Model System for Author):

No concerns on the above points

Referee #2 (Remarks for Author):

The authors have addressed all my comments.
We thank the reviewer for his/her time in assessing our manuscript and the helpful comments that have substantially improved this manuscript.

We are pleased to inform you that your manuscript is accepted for publication and is now being sent to our publisher to be included in the next available issue of EMBO Molecular Medicine.

EMBO Press Author Checklist

Corresponding Author Name:	
Journal Submitted to:	
Manuscript Number:	

USEFUL LINKS FOR COMPLETING THIS FORM
The EMBO Journal - Author Guidelines
EMBO Reports - Author Guidelines
Molecular Systems Biology - Author Guidelines
EMBO Molecular Medicine - Author Guidelines

Reporting Checklist for Life Science Articles (updated January 2022)
This checklist is adapted from Materials Design Analysis Reporting (MDAR) Checklist for Authors. MDAR establishes a minimum set of requirements in transparent reporting in the life sciences (see Statement of Task: 10.31222/osf.io/9sm4x). Please follow the journal's guidelines in preparing your manuscript.
Please note that a copy of this checklist will be published alongside your article.

Abridged guidelines for figures
1. Data
The data shown in figures should satisfy the following conditions:
- the data were obtained and processed according to the field's best practice and are presented to reflect the results of the experiments in an accurate and unbiased manner.
- ideally, figure panels should include only measurements that are directly comparable to each other and obtained with the same assay.
- plots include clearly labeled error bars for independent experiments and sample sizes. Unless justified, error bars should not be shown for technical replicates.
- if n<5, the individual data points from each experiment should be plotted. Any statistical test employed should be justified.
- Source Data should be included to report the data underlying figures according to the guidelines set out in the authorship guidelines on Data Presentation.

2. Captions
Each figure caption should contain the following information, for each panel where they are relevant:
- a specification of the experimental system investigated (eg cell line, species name).
- the assay(s) and method(s) used to carry out the reported observations and measurements.
- an explicit mention of the biological and chemical entity(ies) that are being measured.
- an explicit mention of the biological and chemical entity(ies) that are altered/varied/perturbed in a controlled manner.
- the exact sample size (n) for each experimental group/condition, given as a number, not a range;
- a description of the sample collection allowing the reader to understand whether the samples represent technical or biological replicates (including how many animals, litters, cultures, etc.).
- a statement of how many times the experiment shown was independently replicated in the laboratory.
- definitions of statistical methods and measures:
  - common tests, such as t-test (please specify whether paired vs. unpaired), simple χ^2 tests, Wilcoxon and Mann-Whitney tests, can be unambiguously identified by name only, but more complex techniques should be described in the methods section;
  - are tests one-sided or two-sided?
  - are there adjustments for multiple comparisons?
  - exact statistical test results, e.g., P values = x but not P values < x;
  - definition of 'center values' as median or average;
  - definition of error bars as s.d. or s.e.m.

Please complete ALL of the questions below.
Select "Not Applicable" only when the requested information is not relevant for your study.

Materials

Newly Created Materials	Information included in the manuscript?	In which section is the information available? (Reagents and Tools Table, Materials and Methods, Figures, Data Availability Section)
New materials and reagents need to be available; do any restrictions apply?	Not Applicable	

Antibodies	Information included in the manuscript?	In which section is the information available? (Reagents and Tools Table, Materials and Methods, Figures, Data Availability Section)
For **antibodies** provide the following information:<br>- Commercial antibodies: RRID (if possible) or supplier name, catalogue number and or/clone number<br>- Non-commercial: RRID or citation	Yes	Appendix Table 3

DNA and RNA sequences	Information included in the manuscript?	In which section is the information available? (Reagents and Tools Table, Materials and Methods, Figures, Data Availability Section)
**Short novel DNA or RNA including primers, probes**: provide the sequences.	Yes	Materials and methods

Cell materials	Information included in the manuscript?	In which section is the information available? (Reagents and Tools Table, Materials and Methods, Figures, Data Availability Section)
**Cell lines:** Provide species information, strain. Provide accession number in repository **OR** supplier name, catalog number, clone number, and/**OR** RRID.	Not Applicable	
**Primary cultures:** Provide species, strain, sex of origin, genetic modification status.	Not Applicable	
Report if the cell lines were recently **authenticated** (e.g., by STR profiling) and tested for mycoplasma contamination.	Not Applicable	

Experimental animals	Information included in the manuscript?	In which section is the information available? (Reagents and Tools Table, Materials and Methods, Figures, Data Availability Section)
**Laboratory animals or Model organisms:** Provide species, strain, sex, age, genetic modification status. Provide accession number in repository **OR** supplier name, catalog number, clone number, **OR** RRID.	Yes	Materials and Methods
**Animal observed in or captured from the field:** Provide species, sex, and age where possible.	Not Applicable	
Please detail **housing and husbandry conditions**.	Yes	Materials and Methods

Plants and microbes	Information included in the manuscript?	In which section is the information available? (Reagents and Tools Table, Materials and Methods, Figures, Data Availability Section)
**Plants:** provide species and strain, ecotype and cultivar where relevant, unique accession number if available, and source (including location for collected wild specimens).	Not Applicable	
**Microbes:** provide species and strain, unique accession number if available, and source.	Not Applicable	

Human research participants	Information included in the manuscript?	In which section is the information available? (Reagents and Tools Table, Materials and Methods, Figures, Data Availability Section)
If collected and within the bounds of privacy constraints report on age, sex and gender or ethnicity for all study participants.	Yes	Appendix Table 2

Core facilities	Information included in the manuscript?	In which section is the information available? (Reagents and Tools Table, Materials and Methods, Figures, Data Availability Section)
If your work benefited from core facilities, was their service mentioned in the acknowledgments section?	Not Applicable	

Design

Study protocol	Information included in the manuscript?	In which section is the information available? (Reagents and Tools Table, Materials and Methods, Figures, Data Availability Section)
If study protocol has been **pre-registered, provide DOI in the manuscript**. For clinical trials, provide the trial registration number **OR** cite DOI.	Not Applicable	
Report the **clinical trial registration number** (at ClinicalTrials.gov or equivalent), where applicable.	Not Applicable	

Laboratory protocol	Information included in the manuscript?	In which section is the information available? (Reagents and Tools Table, Materials and Methods, Figures, Data Availability Section)
Provide DOI OR other citation details if **external detailed step-by-step protocols** are available.	Not Applicable	

Experimental study design and statistics	Information included in the manuscript?	In which section is the information available? (Reagents and Tools Table, Materials and Methods, Figures, Data Availability Section)
Include a statement about **sample size** estimate even if no statistical methods were used.	Yes	Materials and Methods
Were any steps taken to minimize the effects of subjective bias when allocating animals/samples to treatment (e.g. **randomization procedure**)? If yes, have they been described?	Yes	Materials and Methods
Include a statement about **blinding** even if no blinding was done.	Yes	Materials and Methods
Describe **inclusion/exclusion criteria** if samples or animals were excluded from the analysis. Were the criteria pre-established?<br><br>If sample or data points were omitted from analysis, report if this was due to attrition or intentional exclusion and provide justification.	Not Applicable	
For every figure, are **statistical tests** justified as appropriate? Do the data meet the assumptions of the tests (e.g., normal distribution)? Describe any methods used to assess it. Is there an estimate of variation within each group of data? Is the variance similar between the groups that are being statistically compared?	Yes	Normality was tested where required using a Shapiro-Wilk test

Sample definition and in-laboratory replication	Information included in the manuscript?	In which section is the information available? (Reagents and Tools Table, Materials and Methods, Figures, Data Availability Section)
In the figure legends: state number of times the experiment was **replicated** in laboratory.	Yes	
In the figure legends: define whether data describe **technical or biological replicates**.	Yes	

Ethics

Ethics	Information included in the manuscript?	In which section is the information available? (Reagents and Tools Table, Materials and Methods, Figures, Data Availability Section)
Studies involving **human participants**: State details of **authority granting ethics approval** (IRB or equivalent committee(s), provide reference number for approval.	Yes	Materials and Methods
Studies involving **human participants**: Include a statement confirming that **informed consent** was obtained from all subjects and that the experiments conformed to the principles set out in the WMA Declaration of Helsinki and the Department of Health and Human Services Belmont Report.	Yes	Materials and Methods
Studies involving **human participants:** For publication of **patient photos**, include a statement confirming that consent to publish was obtained.	Not Applicable	
Studies involving experimental **animals**: State details of **authority granting ethics approval** (IRB or equivalent committee(s), provide reference number for approval. Include a statement of compliance with ethical regulations.	Yes	Materials and Methods
Studies involving **specimen and field samples:** State if relevant **permits** obtained, provide details of authority approving study; if none were required, explain why.	Not Applicable	

Dual Use Research of Concern (DURC)	Information included in the manuscript?	In which section is the information available? (Reagents and Tools Table, Materials and Methods, Figures, Data Availability Section)
Could your study fall under dual use research restrictions? Please check biosecurity documents and list of **select agents and toxins** (CDC): https://www.selectagents.gov/sat/list.htm	Not Applicable	
If you used a select agent, is the security level of the lab appropriate and reported in the manuscript?	Not Applicable	
If a study is subject to dual use research of concern regulations, is the name of the **authority granting approval and reference number** for the regulatory approval provided in the manuscript?	Not Applicable	

Reporting

The MDAR framework recommends adoption of discipline-specific guidelines, established and endorsed through community initiatives. Journals have their own policy about requiring specific guidelines and recommendations to complement MDAR.

Adherence to community standards	Information included in the manuscript?	In which section is the information available? (Reagents and Tools Table, Materials and Methods, Figures, Data Availability Section)
State if relevant guidelines or checklists (e.g., **ICMJE, MIBBI, ARRIVE, PRISMA**) have been followed or provided.	Not Applicable	
For **tumor marker prognostic studies,** we recommend that you follow the **REMARK** reporting guidelines (see link list at top right). See author guidelines, under 'Reporting Guidelines'. Please confirm you have followed these guidelines.	Not Applicable	
For **phase II and III randomized controlled trials**, please refer to the **CONSORT** flow diagram (see link list at top right) and submit the CONSORT checklist (see link list at top right) with your submission. See author guidelines, under 'Reporting Guidelines'. Please confirm you have submitted this list.	Not Applicable	

Data Availability

Data availability	Information included in the manuscript?	In which section is the information available? (Reagents and Tools Table, Materials and Methods, Figures, Data Availability Section)
Have **primary datasets** been deposited according to the journal's guidelines (see 'Data Deposition' section) and the respective accession numbers provided in the Data Availability Section?	Not Applicable	
Were **human clinical and genomic datasets** deposited in a public access-controlled repository in accordance to ethical obligations to the patients and to the applicable consent agreement?	Not Applicable	
Are **computational models** that are central and integral to a study available without restrictions in a machine-readable form? Were the relevant accession numbers or links provided?	Not Applicable	
If publicly available data were reused, provide the respective **data citations in the reference list**.	Not Applicable	
